# Benzophenone Derivatives as a New Class of Anti‐Infectives Against *Acanthamoeba spp*

**DOI:** 10.1002/ardp.70321

**Published:** 2026-08-20

**Authors:** Tomas Rimkus, Hannes Geisler, Taha B. El‐Jourani, Aurélien F. A. Moumbock, Matthias Schiedel, Stephan Reichl

**Affiliations:** ^1^ Institute of Pharmaceutical Technology and Biopharmaceutics Technische Universität Braunschweig Braunschweig Germany; ^2^ Institute of Medicinal and Pharmaceutical Chemistry Technische Universität Braunschweig Braunschweig Germany; ^3^ Institute of Pharmaceutical Sciences Albert‐Ludwigs‐Universität Freiburg Freiburg Germany; ^4^ Universität Münster Institut für Pharmazeutische und Medizinische Chemie Münster Germany; ^5^ Center of Pharmaceutical Engineering Technische Universität Braunschweig Braunschweig Germany

**Keywords:** *Acanthamoeba*, anti‐infectives, cycloartenol synthase, keratitis, Ro 48‐8071

## Abstract

The rare but sight‐threatening ocular disease *Acanthamoeba* keratitis represents a therapeutic challenge due to its causative agents, *Acanthamoeba spp*., being able to penetrate the corneal epithelium and persist in the corneal stroma as dormant and highly resistant cysts. To combat the scarcity and limitations of current treatment options, we developed a new class of anti‐infective agents against *Acanthamoeba spp*. based on Ro 48‐8071 (**3**), a known inhibitor of the human oxidosqualene cyclase (hOSC). The compound design was guided by docking studies using a homology model of the cycloartenol synthase (CAS), which is the homolog of hOSC found in *Acanthamoeba spp*. and essential for the parasite's steroid biosynthesis. Among the synthesized Ro 48‐8071 analogues, MSHG19 (**4f**) evoked the most potent activity against *Acanthamoeba hatchetti* leading to total eradication of trophozoites and cysts at single‐digit micromolar concentrations, whereas having no effects within this concentration range on the viability of human corneal epithelial cells. With our initial set of synthesized compounds, we established a first structure–activity relationship model, which nicely aligns with the predicted binding mode of **4f** to CAS. Overall, the presented findings highlight the potential of benzophenones as a new class of anti‐infectives for the treatment of *Acanthamoeba* keratitis.

## Graphical abstract

Analogues of the human oxidosqualene cyclase inhibitor Ro 48‐8071 were designed, synthesized, and tested against *Acanthamoeba hatchetti*, one of the pathogens causing *Acanthamoeba* keratitis. The *N*‐propargyl derivative **4f** showed strong amoebicidal activity with complete eradication of trophozoites and cysts, while exhibiting low toxicity toward human corneal epithelial cells, highlighting its potential to combat *Acanthamoeba*‐related infections.

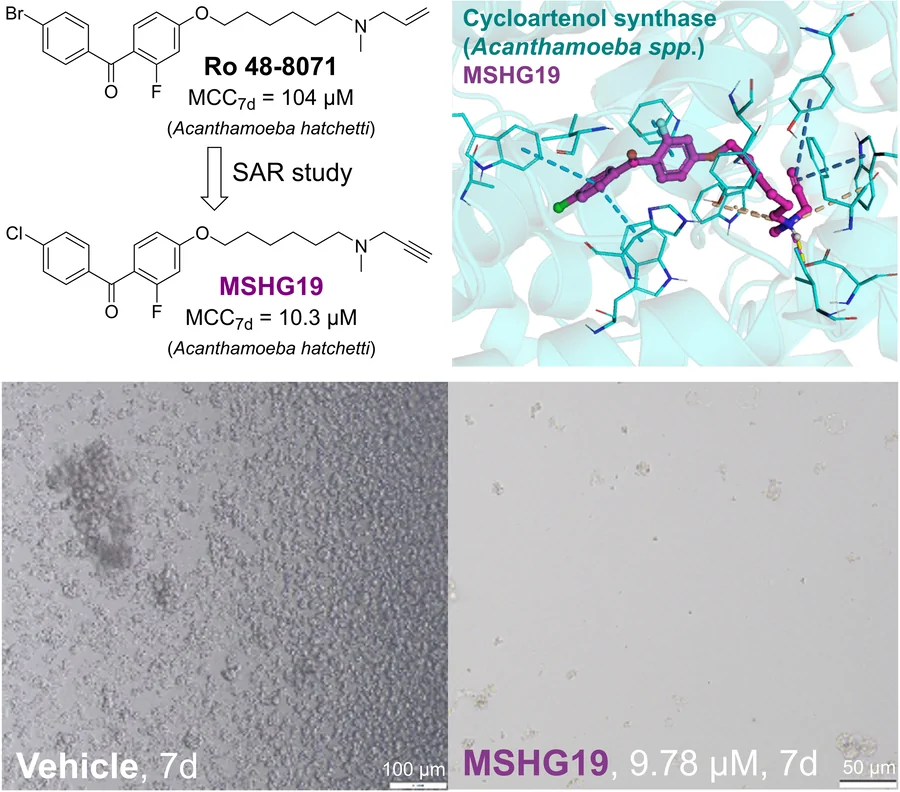

## 1 Introduction

**Figure 1:**
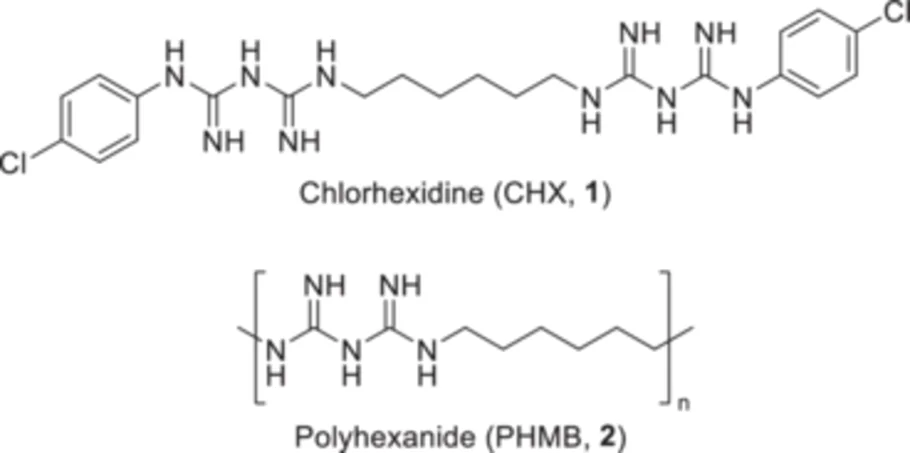
Chemical structures of the antiseptic compounds chlorhexidine (CHX) and polyhexanide (PHMB) that represent the current gold standards for the treatment of Acanthamoeba keratitis (AK).

*Acanthamoeba* keratitis (AK) is a rare, often unilaterally occurring infection of the human eye, predominantly affecting contact lens wearers around the world. Its causative agents—trophozoites and cysts of *Acanthamoeba species pluralis (spp.)*—are a type of ubiquitous, free‐living amoeba and represent a substantial challenge regarding their eradication from infected tissues. After penetration of vegetative trophozoites through the epithelium and Bowman's layer into the human corneal stroma, the parasites can form dormant, double‐walled and highly resistant cysts [1]. This contributes to the difficulty of treating *Acanthamoeba*‐related infections, as cysts can withstand frequent and prolonged exposure to anti‐infective agents and hatch upon survival, thus leading to recurrence of active infection and inflammation [2]. Thus, with the available therapeutic agents, a complex multi‐month regimen is necessary to ensure complete parasite eradication. The current gold‐standard compounds for the treatment of AK are the biguanides chlorhexidine (CHX) and polyhexanide (PHMB) [3, 4, 5, 6], which have been known for decades and act as antiseptic compounds *via* a non‐specific mechanism [7] (Figure 1).

Due to their high basicity, these compounds exhibit a polycationic nature under physiological pH conditions, which is concomitant with pharmacokinetic disadvantages regarding their penetration into the corneal stroma. Thus, a so‐called *abrasio corneae*—the debridement of the human corneal epithelium—is the first step of standard AK therapy with CHX [8, 9] or PHMB [9], facilitating an enhanced penetration of these anti‐infectives into the corneal stroma. In the first days of treatment, this is followed by hourly instillations of the drug, which represents a significant burden for the patient. These circumstances highlight the urgent need for new, highly effective, tolerable, and specific agents for AK treatment.

Recent studies have been investigating a plethora of structurally diverse compounds, including miltefosine [10], amphotericin B [11], oxasqualenoids [12], quaternary ammonium compounds [13, 14], alkylphosphocholines [13], diamidines [15], derivatives of ursolic acid [16], and voriconazole [10] as well as other azole‐antimycotics [17, 18] for their activity against *Acanthamoeba spp*. Despite these efforts, only a few compounds, including miltefosine, the two diamidines hexamidine and propamidine isethionate, the two biguanides CHX and PHMB [3], and voriconazole [19], have been tested in animals or humans in a clinical setting. To date, the only completed phase 3 clinical trials addressing AK have evaluated a combination therapy with propamidine isethionate and PHMB [20, 21]. Among the preclinically and clinically evaluated compounds, voriconazole was reported to have a particularly high activity against *Acanthamoeba spp*. trophozoites and cysts in vitro [10, 11, 22] and significantly contributed to a positive curative outcome in a clinical setting [19, 23, 24]. Given that (i) voriconazole belongs to the azole antimycotics inhibiting the cytochrome P450 51 (CYP51), also referred to as sterol 14α‐demethylase, in the fungal sterol biosynthesis pathway [25, 26]; and (ii) *Acanthamoeba spp*. cannot—or only in a limited manner—make use of exogenous sterols required for the synthesis of ergosterol [27], targeting the sterol biosynthesis of *Acanthamoeba spp*. represents a promising strategy for the discovery of new anti‐infectives against *Acanthamoeba spp*. Within this context, the cycloartenol synthase (CAS), which is an essential enzyme in the sterol biosynthesis of *Acanthamoeba spp*. catalyzing the conversion of 2,3‐oxidosqualene to cycloartenol [28] and a homolog of the human lanosterol synthase (LAS, also referred to as human oxidosqualene cyclase (hOSC)), should be discussed as a potential anti‐amoebicidal drug target, although the literature is not yet fully conclusive [28, 29].

## 2 Results and Discussion

### 2.1 Identification of Ro 48‐8071 as an Anti‐Amoebic Drug

**Figure 2:**
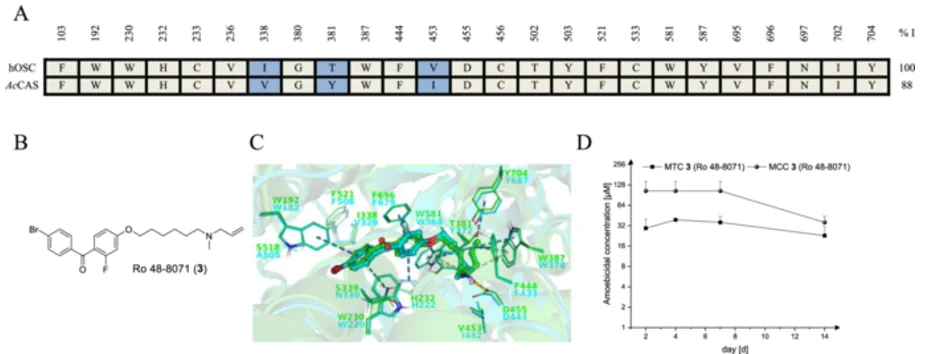
Identification of Ro 48‐8071 (**3**) as an anti‐amoebic drug. (A) Sequence alignment of human OSC (hOSC) and CAS found in *Acanthamoeba spp*., with a focus on amino acids forming the active sites of the respective enzymes. Since the genome of *Acanthamoeba hatchetti* has not been fully sequenced, we used the available CAS sequence from the closely related *Acanthamoeba hatchetti castellanii* for our alignment; differences are marked in blue, with % I marking the percentual sequence identity of both active sites calculated based on hOSC. (B) Chemical structure of **3**, a known inhibitor of hOSC [30]. C) Crystal structure of hOSC in complex with **3** (PDB: pdb_00001w6j, green) overlaid with the predicted binding pose of **3** docked to a homology model of CAS from *Acanthamoeba spp* (cyan). For building our homology model, we used the available CAS sequence from *A. castellanii*, which is closely related to *A. hatchetti*. Salt bridges are labelled in magenta, cation–π–interactions in beige, hydrogen bonds in yellow, and π–π–interactions in blue. (D) Amoebicidal activity of **3** determined in an eradication assay with *A. hatchetti* (2HH strain) trophozoites and cysts. Minimal trophicidal concentration (MTC) and minimal cysticidal concentration (MCC) were determined over a period of 14 days, mean ± SD, *n *≥ 6).

Based on the aforementioned reports that highlight CAS as a potential drug target for the treatment of Acanthamoeba infections and the high sequence similarity between CAS in *Acanthamoeba spp*. and hOSC (Figure 2A (sequence identity within the ligand binding site: 88%), Supporting Information S1: Figure S1 (overall sequence identity: 50%)), we first aimed for investigating Ro 48‐8071 (**3**, Figure 2B), a known inhibitor of human OSC [30], regarding its activity against *Acanthamoeba spp*. trophozoites and cysts. Our interest in **3** as a potential anti‐amoebic agent was further stimulated by its highly similar predicted binding mode in an *Acanthamoeba spp*. homology model compared to its reported binding mode in hOSC (Figure 2C) [30]. Although the model is derived from the reported CAS of Acanthamoeba castellanii [28], the A. castellanii strain available in our laboratory (NEFF) did not reliably form cysts under our experimental conditions. Thus, for our biological evaluation, Acanthamoeba hatchetti (2HH strain) [31, 32] was used as a model organism, since this pathogenic strain is among the causative agents of AK [31] and cyst formation is a key determinant of both pathogenicity and drug resistance in *Acanthamoeba spp* [33]. As a readout for our activity assays, which were performed according to a previously published protocol [14], we chose to report minimal trophicidal concentrations (MTCs) and minimal cysticidal concentrations (MCCs), respectively, rather than EC_50_ values. We consider MTCs and MCCs to be more relevant for future therapeutic applications, since the primary goal is a complete eradication of the pathogen, whereas partial eradication might lead to a recurrence of the infection. Gratifyingly, our investigations indicated that **3** evokes a total eradication of both trophozoites and cysts with amoebicidal activities in a similar range as reported for CHX and PHMB, the current gold standards for AK treatment [34, 35]. In detail, a 2‐h treatment with **3** and a subsequent compound removal resulted on Day 2 in a full eradication of trophozoites with an MTC of 29.3 ± 10.7 µM. Even 14 dayspost‐exposure, similar effects were observed (MTC = 22.8 ± 7.97 µM). For the more drug‐resistant cysts, we detected MCC values of 104.2 ± 39.1 µM (2 days) and 35.8 ± 7.97 µM (14 days). These results indicate a prolonged amoebicidal activity of **3**. As aforementioned, this is especially relevant with respect to the reported phenomenon of *Acanthamoeba spp*. recovery after drug exposure [14, 36, 37].

### 2.2 Structure–Activity Relationship (SAR) of Ro 48‐8071 Analogues

Based on the encouraging amoebicidal activity of **3**, we aimed for establishing an initial SAR model. These studies were intended to identify key structural moieties of **3** involved in protein–ligand interactions, validate the predicted binding mode to *Ac*CAS, and ultimately guide the development of analogues with enhanced amoebicidal activity. Based on the known binding mode of **3** to hOSC and its predicted binding pose to *Ac*CAS (Figure 2C), we first wanted to study the *N*‐allyl moiety. To shed light on the importance of the predicted π–π–interaction of the allyl group with Trp378, Phe433, Trp568, and Tyr687, we synthesized the *N*‐propyl (**4a**) and *N*‐propargyl (**4b**) analogues. In the case of the *N*‐propyl analogue **4a**, the lacking double bond should lead to a loss of the π–π interactions and to a reduced activity, whereas the additional π‐electrons of the triple bond of the *N*‐propargyl derivative **4b** might result in increased π–π interactions. To study whether or not the ionic interaction between the catalytic Asp444 and the protonated and thus positively charged tertiary amine is essential for biological activity, we synthesized the *O*‐allyl analogue **5**, which should not be able to form this interaction. Since it was reported for hOSC that the length of the linker between the amine's nitrogen and the electron‐deficient benzophenone has a pronounced effect on inhibitor binding affinity and that longer linkers lead to steric clashes or constrained inhibitor conformations [30], we aimed for synthesizing compound **4c** featuring a heptyl instead of a hexyl linker to study if this is also true for *Acanthamoeba spp*. CAS. SAR studies for hOSC indicated that electron‐withdrawing substituents in position 4 of the terminal phenyl increase potency compared with the unsubstituted analogues, which might be attributed to increased interactions with the electron‐rich tryptophan residues (Trp192 and Trp230) within this subpocket [30]. Thus, we synthesized the chloro analogue **4d** and the unsubstituted compound **4e** as a control. For the design of compound **4f** (MSHG_019), we combined the 4‐chloro substituent at the terminal phenyl with the *N*‐propargyl moiety that was envisaged to result in an increased biological activity.

**Scheme 1:**
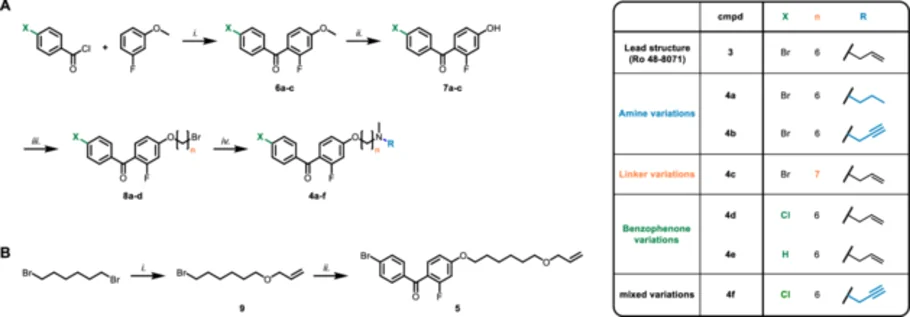
Synthesis of new Ro 48‐8071 (**3**) analogues. Reagents and conditions: (A) *i*. AlCl_3_, dry DCM, 0°C to rt, 24 h, 37%–47% yield. *ii*. BBr_3_, dry DCM, 0°C to rt, overnight, 83%–99% yield. *iii*. Respective α,ω‐dibromoalkane, K_2_CO_3_, acetone, reflux, 5 h, 69%–75% yield. *iv*. Respective amine, DMA, 0°C–70°C, overnight, 65%–89% yield. (B) *i*. allyl alcohol, NaH, dry THF, N_2_ atmosphere, rt, 19 h, 51% yield. *ii*. **7a**, K_2_CO_3_, acetone, reflux, 2.5 h, 96% yield.

For the synthesis of the envisaged compounds **4a–f**, we adapted a synthesis procedure reported by Dehmlow et al. [38](Scheme 1A). Starting with a Friedel–Crafts acylation of 3‐fluoroanisole with benzoyl chlorides, we obtained the 4‐methoxybenzophenones analogues **6a–c** that were subsequently *O*‐demethylated with boron tribromide to yield the 4‐hydroxybenzophenones **7a–c**. These 4‐hydroxybenzophenones were alkylated with an excess of the respective α,ω‐dibromoalkane to give the intermediates **8a–d**, which were transformed to the final tertiary amines (**4a–f**) *via* an S_N_2 reaction with the respective secondary amines.

For the synthesis of *O*‐allyl analogue **5** (Scheme 1B), we started with 1,6‐dibromohexane and allyl alcohol to furnish the allyl 6‐bromohexyl ether (**9**) by means of a Williamson ether synthesis. A second Williamson ether synthesis with **9** and the intermediate **7a** gave the final *O*‐allyl analogue **5** of the parent compound **3**.

Next, we evaluated the anti‐amoebic activities of the synthesized analogues of **3**. To this end, we applied the aforementioned approach for the biological characterization of the amoebicidal activity of **3**. The corresponding MTCs and MCCs for all tested compounds are summarized in Table 1.

**Table 1:** MTCs and MCCs for all investigated compounds over the course of 14 days, asterisk—1 out of 3 samples exhibited values ≥ 156.3 and fell out of the tested range, mean ± SD, *n* = 3–9.

Day [d]	MTC [µM]							
	**3**	**4a**	**4b**	**4c**	**4 d**	**4e**	**4 f**	**5**
2	29.3 ± 10.7	39.1	9.77	39.1	78.1 ± 67.7	156*	6.51 ± 2.52	≥ 156
4	39.1	39.1	7.32 ± 2.67	58.6 ± 21.4	117 ± 68	156*	4.88	≥ 156
7	35.8 ± 8.0	58.6 ± 21.4	10.6 ± 4.8	39.1	78.1 ± 67.7	156*	4.88	≥ 156
14	22.8 ± 8.0	58.6 ± 21.4	11.4 ± 4.0	39.1	117 ± 68	≥ 156	5.70 ± 1.99	≥ 156

**Day [d]**	**MCC [µM]**							
	**3**	**4a**	**4b**	**4c**	**4 d**	**4e**	**4 f**	**5**
2	104 ± 39	104 ± 39	11.4 ± 6.5	65.1 ± 19.5	n.d.	n.d.	11.4 ± 6.5	n.d.
4	104 ± 39	104 ± 39	13.6 ± 7.2	73.8 ± 13.0	n.d.	n.d.	16.3 ± 23.3	n.d.
7	104 ± 39	148 ± 26	14.1 ± 5.2	73.8 ± 13.0	n.d.	n.d.	10.3 ± 5.7	n.d.
14	35.8 ± 8.0	143 ± 32	17.1 ± 12.3	78.1 ± 42.8	n.d.	n.d.	8.14 ± 2.5	n.d.

Abbreviation: n.d., not determined.

**Figure 3:**
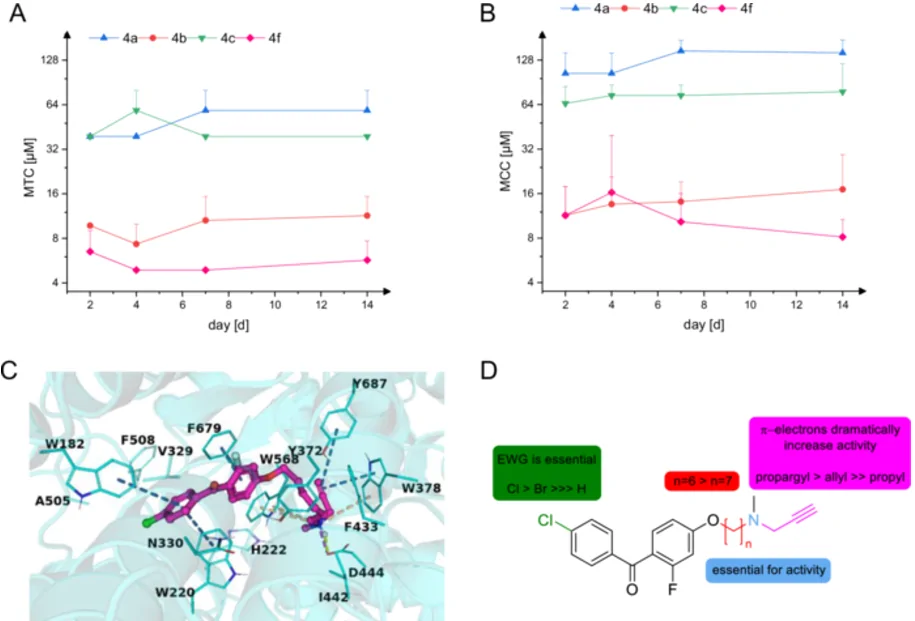
Amoebicidal activities of selected **3** analogues **4a–c** and **4f**. (A) MTCs determined over a period of 14 days, mean ± SD, *n* = 6. (B) MCCs determined over a period of 14 days, mean ± SD, *n* = 6–9. (C) Predicted binding pose of the most active benzophenone derivative **4f** (violet) docked to a homology model of *Ac*CAS (cyan). Salt bridges are labelled in magenta, cation‐π interactions in beige, hydrogen bonds in yellow, and π–π interactions in blue. (D) Initial structure–activity relationship (SAR) model for the anti‐amoebic effects of the benzophenone **4f** and its derivatives.

Furthermore, the amoebicidal activities of the compounds that showed sufficient effects are illustrated in Figure 3A, B, respectively. In brief, compared with **3** (MTC_2d_ = 29.3 ± 10.7 µM, MTC_14d_ = 22.8 ± 8.0 µM, MCC_2d_ = 104 ± 39 µM, and MCC_14d_ = 35.8 ± 8.0 µM), **4a** with its *N*‐propyl group showed reduced amoebicidal activities (MTC_2d_ = 39.1 ± 0.0 µM, MTC_14d_ = 58.6 ± 21.4 µM, MCC_2d_ = 104 ± 39 µM, and MCC_14d_ = 143 ± 32 µM), whereas the *N*‐propargyl analogue **4b** evoked improved anti‐amoebic effects (MTC_2d_ = 9.77 ± 0.0 µM, MTC_14d_ = 11.4 ± 4.0 µM, MCC_2d_ = 11.4 ± 6.5 µM, and MCC_14d_ = 17.1 ± 12.3 µM). These results indicate that π–π interactions between the *N*‐allyl moiety of **3** and Trp378, Phe433, Trp568, and Tyr687 contribute to ligand binding, in a similar manner as reported for hOSC (see Figure 1C), and that these interactions can be strengthened by adding two further π‐electrons when moving from a double to a triple bond. In full agreement with the reported binding mode of **3** to hOSC [30], the disruption of the ionic interaction with Asp444 resulted in a complete loss of activity, as shown by the *O*‐allyl analogue **5** (MTC_2d_ ≥ 156 µM). Thus, highlighting the utmost importance of this ionic interaction for target binding. Due to the complete inactivity of **5**, we refrained from further biological characterization of this compound. A longer heptyl linker was tolerated but did not result in improved amoebicidal activities (**4c**: MTC_2d_ = 39.1 ± 0.0 µM, MTC_14d_ = 39.1 ± 0.00 µM, MCC_2d_ = 65.1 ± 19.5 µM, and MCC_14d_ = 78.1 ± 42.8 µM). Removal of the 4‐bromo substituent at the terminal phenyl of the benzophenone moiety led to a complete loss of activity (**4e**: MTC_2d_ ≥ 156 µM, and MTC_14d_ ≥ 156 µM), whereas a 4‐chloro substituent partially restored the amoebicidal activity (**4d**: MTC_2d_ = 78.1 ± 67.7 µM, and MTC_14d_ ≥ 117 ± 68 µM), thereby highlighting the importance of an electron withdrawing group at this position. This fully aligns with both the previously described observations for hOSC [30, 38] and our proposed binding mode for **3** to *Acanthamoeba spp*. CAS (Figure 2C), since an electronegative substituent at this position should lead to enhanced binding interactions of the electron‐deficient phenyl moiety with the electron‐dense pocket of the active site by facilitating π–π interactions with the surrounding Trp182 and Trp220 (corresponding to Trp192 and Trp230 in hOSC). Finally, by combining the *p*‐chloro substituent at the terminal phenyl of the benzophenone moiety with the propargyl group of our most active compound **4b**, we were able to further increase the amoebicidal activities to low two‐digit or even single‐digit MTCs and MCCs (**4f**, MTC_2d_ = 6.51 ± 2.52 µM, MTC_14d_ = 5.70 ± 1.99 µM, MCC_2d_ = 11.4 ± 6.5 µM, and MCC_14d_ = 8.14 ± 2.5 µM). Molecular dynamics (MD) simulations indicated a high stability of the complex of **4f** bound to *Ac*CAS (Figure 3C) over a time period of 100 ns (Supporting Information S1: Figure S2) and further highlighted the interactions with specific amino acids (*e.g*., Trp182, Trp220, Trp378, Phe433, Asp444, Trp568, and Phe679; see Supporting Information S1: Figure S3A,B) that were already suggested by the docking studies (Figure 3C), thereby further supporting the proposed binding mode of **4f**. The initial SAR model for the anti‐amoebic effects of **4f** and its derivatives is illustrated in Figure 3D.

**Figure 4:**
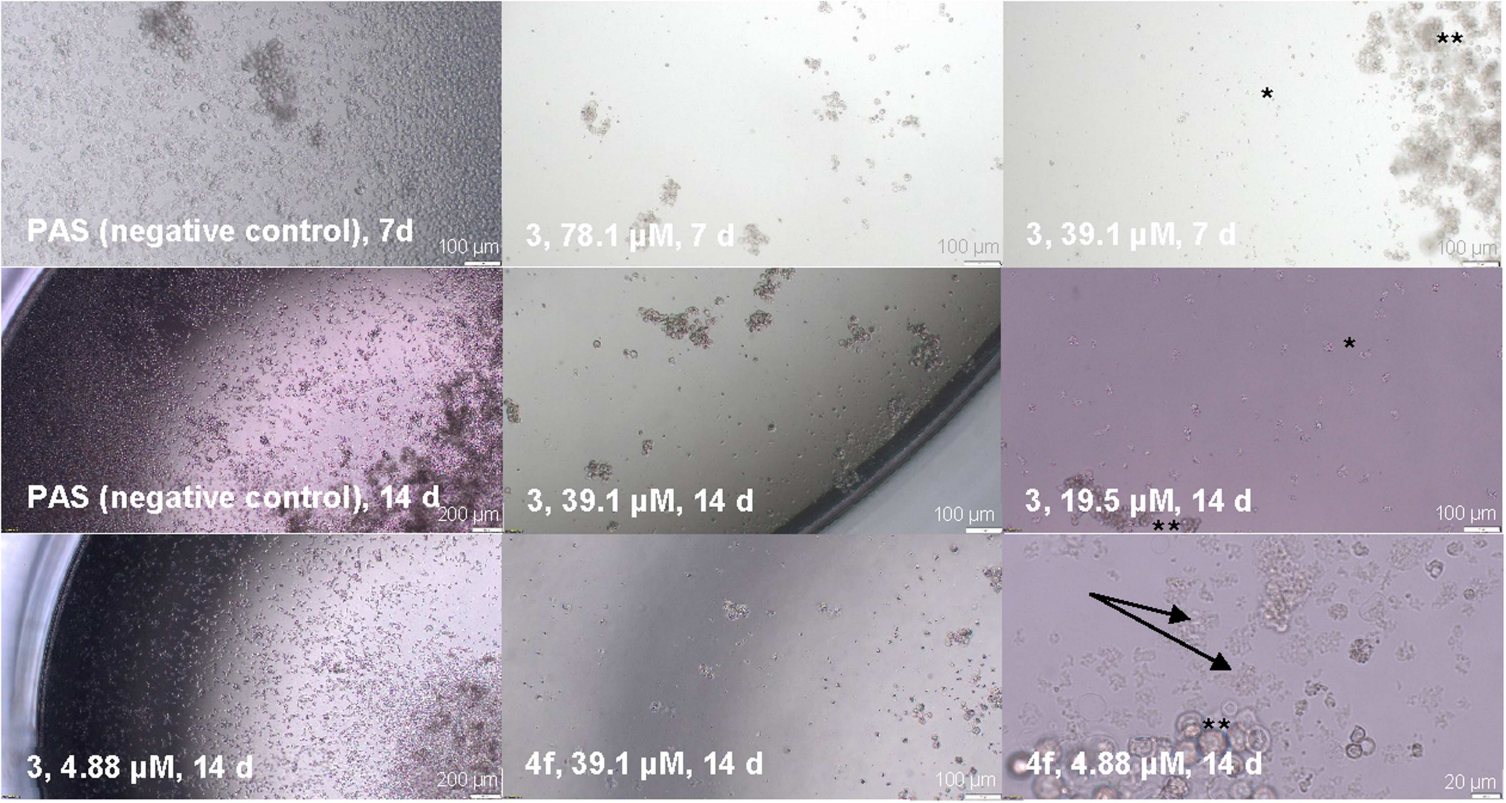
Representative microscopic images of *Acanthamoeba hatchetti* (2HH strain) after 7 and 14 days of MCC testing under given conditions with the respective size of the scale bar indicated on the bottom right; * ‐ cluster of live trophozoites, ** ‐ cluster of cysts, arrows ‐ dead trophozoites; enlarged and additional images can be accessed in the supporting information.

Additionally, representative microscopic images are shown in Figure 4. The irregular morphology of treated trophozoites compared with the negative control supports the proposed mechanism of action involving small‐molecule‐mediated CAS inhibition and disruption of parasite steroid biosynthesis.

For reference, the negative control at 7 days illustrates viable trophozoites and cysts, whereas after 14 days, a slight change in morphology is observable due to overgrowth. The long‐term effect after initial application of 3 is illustrated at 39.1 µM, 7 or 14 days after exposure, where previously living but visibly affected amoeba (**7d**) could not recuperate after 14 days. The superior efficacy of our lead compound **4f** compared with **3** is evidenced by its visible impact on cell viability at 4.88 µM after 14 days. At these conditions, trophozoites appear malformed or crumbled without a clearly defined membrane, indicating dead cells. Shrinkage of most endocysts with the absence of overall proliferation illustrates the specific impact on cysts, although both stages are considered when determining the MCC, as absence of growth after 14 days is the deciding factor. As mentioned before, our method for determining the amoebicidal activities identifies only those conditions as efficacious, at which no viable *Acanthamoeba* are detectable. Consequently, these results likely represent conservative estimates of efficacy under real‐world conditions, since *Acanthamoeba spp*. can recover if complete eradication is not achieved [2]. Nevertheless, pronounced inhibitory effects on trophozoite and cyst proliferation are evident even at concentrations well below the determined MTCs/MCCs.

### 2.3 Effects on the Viability of Human Corneal Epithelial Cells

**Figure 5:**
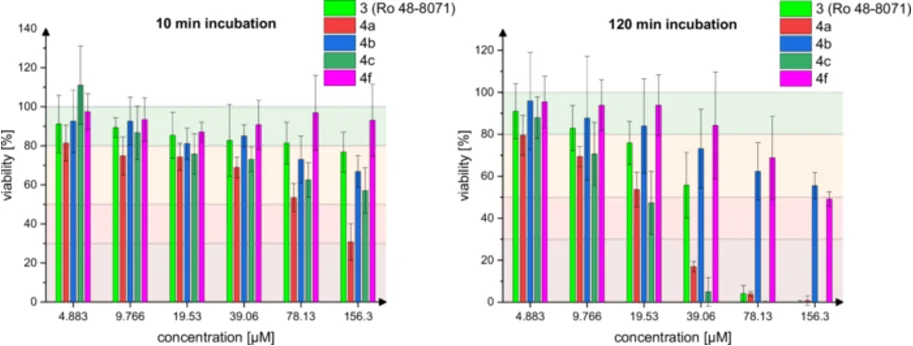
Viability studies with human corneal epithelial cells. Bar diagram represents relative viabilities of HCE‐T cells after 10 min (A) or 120 min (B) of incubation with the investigated compounds, mean ± SD, *n* = 6.

Compounds intended for AK treatment should achieve complete eradication of *Acanthamoeba spp*. trophozoites and cysts, while exerting minimal or no detrimental effects on relevant host cells. To study the effects of **3** and its most promising analogues (*i.e*., **4a–c** and **4f**) on host cells, we performed MTT‐based viability assays. The human corneal epithelial cell line HCE‐T was chosen as a model cell line, since these cells represent the human corneal epithelium. For our viability assay, HCE‐T cells were exposed to our test compounds for 10 and 120 min, respectively. These incubation times simulate realistic short‐term exposure upon application of eye drops (10 min) and hypothetical longer term exposure (120 min), respectively. The observed reduction in viability was normalized to Krebs–Ringer bicarbonate (KRB) buffer as a control and is illustrated in Figure 5. To facilitate a clearer interpretation of the results, we defined “no toxicity” as 100%–80%, “mild toxicity” as 80%–50%, “medium toxicity” as 50%–30%, and “high toxicity” as 30%–0% remaining viability. After 10 min of incubation, “mild toxicity” on HCE‐T cells was observed for **3** at 156 µM, **4a** at 9.77 µM, **4b** at 78.1 µM, and **4c** at 19.5 µM, whereas **4f** showed no toxicity, even at the highest tested concentration of 156 µM. Only **4a** led to a stronger reduction in viability (30.8%) at 156 µM indicating “medium toxicity” under these conditions. These results indicate that **3**, **4b**, and **4f** exert little detrimental impact on host cells at amoebicidal concentrations (see Table 1) after short‐term exposure.

After 120 min of incubation, the impact on HCE‐T cell viability of the investigated compounds varies considerably. “Mild toxicity” was evoked by **3** at 19.5 µM, **4a** at 4.88 µM, **4b** at 39.1 µM, **4c** at 9.77 µM, and **4f** at 78.1 µM. “Medium toxicity” was first detected for following conditions: **4a** at 39.1 µM, **4c** at 19.5 µM. and **4f** at 156 µM. **4b** did not lead to values below 50% at any given concentration. Finally, the threshold for “high toxicity” (≤ 30%) was met for **3** at 78.1 µM, **4a** at 39.1 µM, **4c** at 39.1 µM. Fortunately, the two *N*‐propargyl analogues **4b** and **4f**, which featured the highest amoebicidal activities, did not evoke “high toxicity,” even at the highest test concentration (156 µM). Thus, these compounds show by far the most favorable efficacy versus toxicity profiles among the evaluated compounds, which is very promising, considering the reported high cytotoxicity of PHMB [39, 40] and—to a lesser extent—CHX [39, 41] on host cells.

## 3 Conclusion

Our investigations highlight a series of benzophenones as highly efficacious agents against *Acanthamoeba spp*. and thus as novel potential anti‐infectives for the treatment of *Acanthamoeba*‐related diseases, such as AK. First, we identified the benzophenone Ro 48‐8071 (**3**), a known inhibitor of the human oxidosqualene cyclase (hOSC) that is closely related to the CAS of *Acanthamoeba spp*., as a highly active anti‐amoebic agent with prolonged amoebicidal effects both on trophozoites and cysts. Intrigued by these encouraging findings, we designed, synthesized, and tested several analogues of **3** in order to establish a first SAR model for benzophenones as anti‐amoebic drugs and eventually improve their amoebicidal activity while reducing toxicity on host cells. In the course of this SAR study, we identified three key structural motifs that are essential for anti‐amoebic activity: (i) an electron‐withdrawing substituent (*e.g*., chloro and bromo) in *the para* position of the terminal phenyl ring of the benzophenone, (ii) a tertiary amine with either an *N*‐allyl or *N*‐propargyl moiety, and (iii) an alkoxy linker, ideally hexyloxy, connecting the tertiary amine with the benzophenone at the *para* position of its non‐terminal phenyl ring. A removal of one of these structural motifs led to a drastic loss of activity. This steep and very clear SAR model fully aligns with the reported SAR for **3** and its analogues as hOSC inhibitors, thus suggesting that **3** and its derivatives exert their anti‐amoebic effects by inhibiting the ortholog of hOSC in *Acanthamoeba spp*., which is closely related to CAS. Among the synthesized benzophenones, we identified the *N*‐propargyl derivatives **4b** and **4f** as the most active agents against *Acanthamoeba hachettii*. Compared with our starting point **3**, these two compounds showed improved amoebicidal activities and, which might be even more important with respect to their future application for AK treatment, substantially reduced effects on the viability of HCE‐T cells—a surrogate for human corneal epithelial cells. Another advantage of **4b** and **4f**, compared with the polycationic CHX and PHMB that represent the current standard of care for the treatment of AK, is their druglike structure, which should allow them to permeate through the corneal epithelium and the Bowman's layer more easily to reach the stroma, where the resistant cysts are located. Considering recent reports indicating that dual inhibition of *Acanthmoeba's* steroid biosynthesis, for example by pitavastatin and isavuconazole, evokes synergistic effects against trophozoites of *A. castellanii* [42], we will evaluate our novel benzophenones in combination with other compounds that are known to interfere with the parasite's steroid biosynthesis. Further follow‐up studies will be focused on more detailed mechanistic studies and the determination of EC_50_ values to enable better comparability with previously reported anti‐amoebic compounds. Further evaluation using additional pathogenic *Acanthamoeba* strains will be necessary to provide a more comprehensive characterization of our lead compounds and to assess their suitability for clinical development. Moreover, hOSC inhibition has been associated with adverse effects in rats following prolonged administration of high doses over several weeks [43, 44]. Based on the concentrations of **4b** and **4f** reported in the present study, we anticipate that the risk of such adverse effects in the treatment of AK will be substantially reduced, owing to the short treatment duration and the markedly lower concentrations required for efficacy. Ultimately, subsequent in vivo studies will be required to further evaluate these aspects and may advance this compound class toward potential clinical application for the treatment of AK.

## 4 Experimental

### 4.1 Chemistry

#### 4.1.1 General Remarks

Starting materials were purchased from commercial suppliers (abcr, Alfa Aesar, BLDpharm, TCI, Thermo scientific, Sigma Aldrich) and used without further purification. Solvents were used in p.a. quality and dried according to common procedures, if necessary. All reactions that required dry conditions were carried out in flame‐dried flasks covered with a drying tube filled with calcium chloride. Literature‐known compounds were synthesized according to previously published procedures (**3** (Ro 48‐8071) [45], **6a** [46], **8a** [45]). Thin‐layer chromatography (TLC) for reaction monitoring was performed with polyester sheets coated with Macherey–Nagel silica gel 60 F_254_ (layer thickness: 0.2 mm) and analysed under UV‐light (254 nm). UV‐inactive compounds were analysed using KMnO_4_ staining. Low resolution mass spectrometry (LRMS) was performed with an Advion expression compact mass spectrometer (CMS) coupled with a Plate Express (Advion) TLC plate reader using electrospray ionization (ESI) and atmospheric pressure chemical ionization (APCI). High‐resolution mass spectrometry (HRMS) was performed with an LTQ‐Orbitrap Velos spectrometer using electrospray ionization (ESI). Flash column chromatography was performed with hand‐packed silica columns 60 M (0.040–0.063 mm, 230–400 mesh) as a stationary phase on a Biotage Selekt automated flash purification system equipped with a diode array detector (DAD). Yields were not optimized. NMR‐spectra were recorded with either a Bruker Avance 500 (^1^H: 500 MHz, ^13^C: 126 MHz) or a Bruker Avance 600 (^1^H: 600 MHz, ^13^C: 151 MHz) instrument. The spectra are referenced against the NMR solvent or tetramethylsilane (TMS) and are reported as follows: ^1^H: chemical shift δ (ppm), multiplicity (s = singlet, d = doublet, dd = doublet of doublets, t = triplet, q = quartet, p = quintet, m = multiplet), integration, coupling constant (*J* in Hz). ^13^C: chemical shift δ (ppm), multiplicity (s = singlet, d = doublet), coupling constant (*J* in Hz), abbreviations: carbons that could not be found in ^13^C spectra but in HMBC or HSQC are additionally marked with an asterisk (*). The assignment resulted from HMBC and HSQC experiments. Purity was determined by HPLC and UV detection and was ≥ 95% for all tested compounds. HPLC analyses were performed using a VWR Hitachi Chromaster HPLC system utilizing a DAD (detection at 220 and 254 nm) and a Merck LiChrospher 100 RP‐18 (5 µm) column with a flow rate of 0.5 mL·min^‐1^. The indicated purity was determined at a wavelength of 254 nm. All tested compounds were dissolved in methanol containing 1% (v/v) TFA (M1) before injection. As the solvent system, the following binary solvent system was used. M1: Elution was performed at room temperature under gradient conditions. Eluent A was water containing 0.5% (v/v) TFA; eluent B was acetonitrile containing 0.5% (v/v) TFA. Linear gradient conditions were as follows: 0–3.0 min: A = 90%, B = 10%; 3.0–18.0 min: linear increase to A = 5%, B = 95%; 18.0–24.0 min: A = 5%, B = 95%, 24.0–27.0 min: linear decrease to A = 90%, B = 10%; 27.0–30.0 min: A = 90%, B = 10%.

#### 4.1.2 Synthesis of [(4‐{[6‐(allylmethyl)amino]hexyl}oxy)‐2‐fluorophenyl](4‐bromophenyl)methanone (**3**, Ro 48‐8071) [45]

{4‐[(6‐Bromohexyl)oxy]‐2‐fluorophenyl}(4‐bromophenyl)methanone (**8a**, 150 mg, 0.327 mmol, 1.0 eq.) was dissolved in dimethylacetamide (2.0 mL). The solution was cooled in an ice bath, and *N*‐methylprop‐2‐en‐1‐amine (60 µL, 0.66 mmol, 2.0 eq.) was added. The reaction was stirred for 20 h at ambient temperature. After complete conversion of starting materials as monitored by TLC, the mixture was diluted with saturated sodium bicarbonate solution (20 mL) and the aqueous layer was extracted with dichloromethane (3 × 20 mL). The combined organic layer was dried over Na_2_SO_4_, filtered, and concentrated under reduced pressure to obtain the crude product as a yellow oil. The crude product was purified by flash column chromatography (petroleum ether/ethyl acetate: gradient 20%–100%) to obtain the product as a yellow resin (95.7 mg, 0.211 mmol, 65%).

*R*_f_ = 0.17 (dichloromethane/methanol: 19:1); ^1^H NMR (500 MHz, DMSO‐*d*_6_ δ [ppm]): 7.79–7.72 (m, 2H, 4‐bromophenyl, H‐2,6), 7.67–7.62 (m, 2H, 4‐bromophenyl, H‐3,5), 7.55 (dd, 1H, J = 8.6, 8.6 Hz, 2‐fluorophenyl, H‐6), 6.97 (dd, 1H, *J* = 12.7, 2.4 Hz, 2‐fluorophenyl, H‐3), 6.93 (dd, 1H, *J* = 8.6, 2.4 Hz, 2‐fluorophenyl, H‐5), 5.85–5.74 (m, 1H, –CH═CH_2_), 5.20–5.04 (m, 2H, –HC═CH_2_), 4.08 (t, 2H, *J* = 6.6 Hz, –O–CH_2_–(CH_2_)_5_–N(CH_3_)–), 2.92 (d, *J* = 6.3 Hz, –N(CH_3_)–CH_2_–HC═CH_2_), 2.27 (t, 2H, *J* = 7.2 Hz, –O–(CH_2_)_5_–CH_2_–N(CH_3_)–), 2.10 (s, 3H, –N(CH_3_)–CH_2_–HC═CH_2_), 1.74 (p, 2H, *J* = 6.6 Hz, –O–CH_2_–CH_2_–(CH_2_)_4_–N(CH_3_)–), 1.47–1.36 (m, 4H, –O–(CH_2_)_2_–CH_2_–CH_2_–CH_2_–CH_2_–N(CH_3_)–), 1.37–1.27 (m, 2H, –O–(CH_2_)_3_–CH_2_–(CH_2_)_2_–N(CH_3_)–); ^13^C NMR (126 MHz, DMSO‐*d*_*6*_, *δ* [ppm]): 190.91 q (C═O), 163.38 q (d, *J* = 11.6 Hz, 2‐fluorophenyl, C‐4), 161.28 q (d, *J* = 252 Hz, 2‐fluorophenyl, C‐2), 136.88 q (4‐bromophenyl, C‐1), 136.21 (–N(CH_3_)–CH_2_–HC═CH_2_), 132.45 (d, *J* = 4.3 Hz, 2‐fluorophenyl, C‐6), 131.70 (4‐bromophenyl, C‐2,6), 131.06 (4‐bromophenyl, C‐3,5), 127.13 q (4‐bromophenyl, C‐4), 117.85 q (d, *J* = 13.6 Hz, 2‐fluorophenyl, C‐1), 116.90 (–N(CH_3_)–CH_2_–HC═CH_2_), 111.39 (d, *J* = 2.6 Hz, 2‐fluorophenyl, C‐5), 102.35 (d, *J* = 25.5 Hz, 2‐fluorophenyl, C‐3), 68.50 (–O–CH_2_–(CH_2_)_5_–N(CH_3_)–), 60.33 (–N(CH_3_)–CH_2_–HC═CH_2_), 56.47 (–O‐(CH_2_)_5_–CH_2_–N(CH_3_)–), 41.67 (–N(CH_3_)–CH_2_–HC═CH_2_), 28.37 (–O–CH_2_–CH_2_–(CH_2_)_4_–N(CH_3_)–), 26.67, 26.53 (–O–(CH_2_)_3_–(CH_2_)_2_–CH_2_–N(CH_3_)–), 25.50 (–O–(CH_2_)_2_–CH_2_–(CH_2_)_3_–N(CH_3_)‐); LRMS (APCI^+^): *m/z* 448 [M + H]^+^; HRMS (ESI^+^): *m/z* calcd for C_23_H_28_BrFNO_2_^+^: 448.12887 [M + H]^+^, found: 448.12879; HPLC retention time: 19.44 min, 98% (M1).

#### 4.1.3 Synthesis of (4‐bromophenyl)[2‐fluoro‐4‐({6‐[methyl(propyl)amino]hexyl}oxy)phenyl]methanone (**4a**)

{4‐[(6‐Bromohexyl)oxy]‐2‐fluorophenyl}(4‐bromophenyl)methanone (**8a**, 133 mg, 0.291 mmol, 1.0 eq.) was dissolved in dimethylacetamide (2.0 mL). The solution was cooled in an ice bath, and *N*‐methylpropan‐1‐amine (60 µL, 0.58 mmol, 2.0 eq.) was added. The reaction was stirred for 18 h at ambient temperature. After complete conversion of starting materials as monitored by TLC, the mixture was diluted with saturated sodium bicarbonate solution (20 mL) and the aqueous layer was extracted with dichloromethane (3 × 20 mL). The combined organic layer was dried over Na_2_SO_4_, filtered, and concentrated under reduced pressure to obtain the crude product as a yellow oil. The crude product was purified by flash column chromatography (dichloromethane/methanol: gradient 5%–10%) to obtain the product as a yellow resin (101 mg, 0.225 mmol, 77%).

*R*_f_ = 0.31 (dichloromethane/methanol: 10:1); ^1^H NMR (600 MHz, CD_3_OD‐*d*_4_ δ [ppm]): 7.70–7.67 (m, 2H, 4‐bromophenyl, H‐2,6), 7.67–7.65 (m, 2H, 4‐bromophenyl, H‐3,5), 7.56 (dd, 1H, *J* = 8.6, 8.6 Hz, 2‐fluorophenyl, H‐6), 6.88 (dd, 1H, *J* = 8.6, 2.4 Hz, 2‐fluorophenyl, H‐5), 6.80 (dd, 1H, *J* = 12.4, 2.4 Hz, 2‐fluorophenyl, H‐3), 4.09 (t, 2H, *J* = 6.4 Hz, –O–CH_2_–(CH_2_)_5_–N(CH_3_)–), 2.61 (t, 2H, *J* = 8.0 Hz, –N(CH_3_)–CH_2_–CH_2_–CH_3_), 2.56 (t, 2H, *J *= 8.0 Hz, –O–(CH_2_)_5_–CH_2_–N(CH_3_)–), 2.41 (s, 3H, –N(CH_3_)–CH_2_–CH_2_–CH_3_), 1.88–1.80 (m, 2H, –O–CH_2_–CH_2_–(CH_2_)_4_–N(CH_3_)–), 1.66–1.51 (m, 6H, –O–(CH_2_)_3_–(CH_2_ )_2_–CH_2_–N(CH_3_)–CH_2_–CH_2_–CH_3_), 1.46–1.38 (m, 2H, –O–(CH_2_)_2_–CH_2_–(CH_2_)_3_–N(CH_3_)–), 0.94 (t, 3H, *J* = 7.4 Hz, –N(CH_3_)–CH_2_–CH_2_–CH_3_); ^13^C NMR (151 MHz, CD_3_OD‐*d*_*4*_, *δ* [ppm]): 193.38 q (C═O), 165.53 q (d, *J* = 11.5 Hz, 2‐fluorophenyl, C‐4), 163.40 q (d, *J* = 253 Hz, 2‐fluorophenyl, C‐2), 138.67 q (4‐bromophenyl, C‐1), 133.73 (d, *J* = 4.2 Hz, 2‐fluorophenyl, C‐6), 132.84 (4‐bromophenyl, C‐2,6), 132.17 (4‐bromophenyl, C‐3,5), 128.89 q (4‐bromophenyl, C‐4), 119.63 q (d, *J* = 13.6 Hz, 2‐fluorophenyl, C‐1) 112.19 (d, *J* = 2.8 Hz, 2‐fluorophenyl, C‐5), 103.29 (d, *J* = 25.5 Hz, 2‐fluorophenyl, C‐3), 69.82 (–O–CH_2_–(CH_2_)_5_–N(CH_3_)–), 60.25 (–N(CH_3_)–CH_2_–CH_2_–CH_3_), 58.29 (–O–(CH_2_)_5_–CH_2_–N(CH_3_)–), 41.83 (–N(CH_3_)–CH_2_–CH_2_–CH_3_), 29.98 (–O–CH_2_–CH_2_–(CH_2_)_4_–N(CH_3_)–), 28.07 (–O–(CH_2_)_4_–CH_2_–CH_2_–N(CH_3_)–), 26.97, 26.88 (–O–(CH_2_)_2_–(CH_2_)_2‐_(CH_2_)_2_–N(CH_3_)–), 20.26 (–N(CH_3_)–CH_2_–CH_2_–CH_3_) 11.90 (–N(CH_3_)–CH_2_–CH_2_–CH_3_); LRMS (APCI^+^): *m/z* 450 [M + H]^+^; HRMS (ESI^+^): *m/z* calcd for C_23_H_30_BrFNO_2_^+^: 450.14453 [M + H]^+^, found: 450.14450; HPLC retention time: 19.54 min, 95% (M1).

#### 4.1.4 Synthesis of (4‐bromophenyl)[2‐fluoro‐4‐({6‐[methyl(prop‐2‐yn‐1‐yl)amino]hexyl}oxy)phenyl]methanone (**4b**)

{4‐[(6‐Bromohexyl)oxy]‐2‐fluorophenyl}(4‐bromophenyl)methanone (**8a**, 150 mg, 0.327 mmol, 1.0 eq.) was dissolved in dimethylacetamide (2.0 mL). The solution was cooled in an ice bath, and *N*‐methylprop‐2‐yn‐1‐amine (60 µL, 0.66 mmol, 2.0 eq.) was added. The reaction was stirred for 20 h at ambient temperature. After complete conversion of starting materials as monitored by TLC, the mixture was diluted with saturated sodium bicarbonate solution (20 mL) and the aqueous layer was extracted with dichloromethane (3 × 20 mL). The combined organic layer was dried over Na_2_SO_4_, filtered, and concentrated under reduced pressure to obtain the crude product as a yellow oil. The crude product was purified by flash column chromatography (petroleum ether/ethyl acetate: gradient 20%–60%) to obtain the product as a pale‐yellow resin (121 mg, 0.270 mmol, 83%).

*R*_f_ = 0.25 (petroleum ether/ethyl acetate: 3:2); ^1^H NMR (500 MHz, DMSO‐*d*_6_ δ [ppm]): 7.79–7.72 (m, 2H, 4‐bromophenyl, H‐2,6), 7.68–7.62 (m, 2H, 4‐bromophenyl, H‐3,5), 7.55 (dd, 1H, *J* = 8.6, 8.6 Hz, 2‐fluorophenyl, H‐6), 6.97 (dd, 1H, *J* = 12.7, 2.4 Hz, 2‐fluorophenyl, H‐3), 6.93 (dd, 1H, *J* = 8.6, 2.4 Hz, 2‐fluorophenyl, H‐5), 4.08 (t, 2H, *J* = 6.5 Hz, –O–CH_2_–(CH_2_)_5_–N(CH_3_)–), 3.27 (d, 2H, *J* = 2.4 Hz, –N(CH_3_)–CH_2_–C ≡ CH) 3.08 (t, 1H, *J* = 2.4 Hz, –C ≡ CH) 2.32 (t, 2H, *J* = 7.1 Hz, –O–(CH_2_)_5_–CH_2_–N(CH_3_)–), 2.17 (s, 3H, –N(CH_3_)–CH_2_–C ≡ CH), 1.78–1.69 (m, 2H, –O–CH_2_–CH_2_–(CH_2_)_4_–N(CH_3_)–), 1.49–1.37 (m, 4H, –O–(CH_2_)_2_–CH_2_–CH_2_–CH_2_–CH_2_–N(CH_3_)–), 1.37–1.29 (m, 2H, –O–(CH_2_)_3_–CH_2_–(CH_2_)_2_–N(CH_3_)–); ^13^C NMR (126 MHz, DMSO‐*d*_*6*_, δ [ppm]): 190.91 q (C═O), 163.38 q (d, *J* = 11.5 Hz, 2‐fluorophenyl, C‐4), 161.27 q (d, *J* = 252 Hz, 2‐fluorophenyl, C‐2), 136.88 q (4‐bromophenyl, C‐1), 132.45 (d, *J* = 4.2 Hz, 2‐fluorophenyl, C‐6), 131.69 (4‐bromophenyl, C‐2,6), 131.06 (4‐bromophenyl, C‐3,5), 127.12 q (4‐bromophenyl, C‐4), 117.85 q (d, *J* = 13.6 Hz, 2‐fluorophenyl, C‐1), 111.39 (d, *J* = 2.6 Hz, 2‐fluorophenyl, C‐5), 102.35 (d, *J* = 25.5 Hz, 2‐fluorophenyl, C‐3), 79.07 q (–N(CH_3_)–CH_2_–C ≡ CH), 75.54 (–N(CH_3_)–CH_2_–C ≡ CH), 68.50 (–O–CH_2_–(CH_2_)_5_–N(CH_3_)–), 54.84 (–O–(CH_2_)_5_–CH_2_–N(CH_3_)–), 44.88 (–N(CH_3_)–CH_2_–C ≡ CH), 41.27 (–N(CH_3_)–CH_2_–C ≡ CH), 28.37 (–O–CH_2_–CH_2_–(CH_2_)_4_–N(CH_3_)–), 26.80, 26.48 (–O–(CH_2_)_3_–(CH_2_)_2_–CH_2_–N(CH_3_)–), 25.27 (–O–(CH_2_)_2_–CH_2_–(CH_2_)_3_–N(CH_3_)‐); LRMS (APCI^+^): *m/z* 446 [M + H]^+^; HRMS (ESI^+^): *m/z* calcd for C_23_H_26_BrFNO_2_^+^: 446.11321 [M + H]^+^, found: 446.11305; HPLC retention time: 19.13 min, 99% (M1).

#### 4.1.5 Synthesis of [4‐({7‐[allyl(methyl)amino]heptyl}oxy)‐2‐fluorophenyl](4‐bromophenyl)methanone (**4c**)

{4‐[(7‐Bromoheptyl)oxy]‐2‐fluorophenyl}(4‐bromophenyl)methanone (**8d**, 84 mg, 0.18 mmol, 1.0 eq.) was dissolved in dimethylacetamide (2.0 mL). The solution was cooled in an ice bath, and *N*‐methylprop‐2‐en‐1‐amine (100 µL, 2.08 mmol, 5.8 eq.) was added. The reaction was stirred for 18 h at room temperature, then heated up to 70°C and stirred for an additional 2 h. After complete conversion of starting materials as monitored by TLC, the mixture was neutralized by the addition of a saturated sodium bicarbonate solution and the aqueous layer was extracted with dichloromethane (3 × 50 mL). The combined organic layer was dried over Na_2_SO_4_, filtered, and concentrated under reduced pressure to obtain the crude product as a yellow oil. The crude product was purified by flash column chromatography (dichloromethane/methanol: gradient 0%–10%) to obtain the product as a yellow resin (73 mg, 0.16 mmol, 89%).

*R*_f_ = 0.33 (dichloromethane/methanol: 20:1); ^1^H NMR (500 MHz, DMSO‐*d*_*6*_, δ [ppm]): 7.79–7.73 (m, 2H, 4‐bromophenyl, H‐2,6), 7.68–7.62 (m, 2H, 4‐bromophenyl, H‐3,5), 7.55 (dd, *J* = 8.6, 8.6 Hz, 1H, 2‐fluorophenyl, H‐6), 6.97 (dd, *J* = 12.7, 2.4 Hz, 1H, 2‐fluorophenyl, H‐3), 6.93 (dd, *J* = 8.7, 2.4 Hz, 1H, 2‐fluorophenyl, H‐5), 5.88–5.77 (m, 1H, –HC═CH_2_), 5.29–5.13 (m, 2H, –HC═CH_2_), 4.09 (t, *J* = 6.4 Hz, 2H, –O–CH_2_–(CH_2_)_6_–N(CH_3_)–), 3.09 (s, 2H, –N(CH_3_)–CH_2_–HC═CH_2_), 2.42 (s, 2H, –O–(CH_2_)_6_–CH_2_–N(CH_3_)–), 2.23 (s, 3H, –N(CH_3_)–CH_2_–HC═CH_2_), 1.74 (p, *J* = 6.7 Hz, 2H, –O–CH_2_–CH_2_–(CH_2_)_5_–N(CH_3_)–), 1.49–1.27 (m, 8H, –O–(CH_2_)_2_–(CH_2_)_4_–CH_2_–N(CH_3_)–); ^13^C NMR^a)^ (126 MHz, DMSO‐*d*_*6*_, δ [ppm]): 190.82 q (C═O), 163.29 q (d, *J* = 11.5 Hz, 2‐fluorophenyl, C‐4), 161.19 q (d, *J* = 252 Hz, 2‐fluorophenyl, C‐2), 136.79 q (4‐bromophenyl, C‐1), 132.38 (d, *J* = 4.2 Hz, 2‐fluorophenyl, C‐6), 131.62 (4‐bromophenyl, C‐2,6), 130.98 (4‐bromophenyl, C‐3,5), 127.05 q (4‐bromophenyl, C‐4), 117.78 q (d, *J* = 13.7 Hz, 2‐fluorophenyl, C‐1), 111.30 (d, *J* = 2.5 Hz, 2‐fluorophenyl, C‐5), 102.27 (d, *J* = 25.5 Hz, 2‐fluorophenyl, C‐3), 68.41 (–O–CH_2_–(CH_2_)_6_–N(CH_3_)–), 59.53 (–N(CH_3_)–CH_2_–HC═CH_2_), 55.98 (–O–(CH_2_)_6_–CH_2_–N(CH_3_)–), 40.96 (–N(CH_3_)–CH_2_–HC═CH_2_), 28.38 (–O–CH_2_–CH_2_–(CH_2_)_5_–N(CH_3_)–), 28.22, 26.48, 25.82, 25.22 (–O–(CH_2_)_2_–(CH_2_)_4_–CH_2_–N(CH_3_)–); LRMS (APCI^+^): *m/*z 462 [M + H]^+^; HRMS (ESI^+^): *m/z* calcd for C_24_H_30_BrFNO_2_^+^: 462.14385 [M + H]^+^, found: 462.14470;

HPLC retention time: 19.87 min, 95% (M1). ^a)^ the ^13^C NMR signal for –HC═CH_2_ could not be detected.

#### 4.1.6 Synthesis of [4‐({6‐[allyl(methyl)amino]hexyl}oxy)‐2‐fluorophenyl](4‐chlorophenyl)methanone (**4d**)

{4‐[(6‐Bromohexyl)oxy]‐2‐fluorophenyl}(4‐chlorophenyl)methanone (**8b**, 222 mg, 0.537 mmol, 1.0 eq.) was dissolved in dimethylacetamide (2.0 mL). The solution was cooled in an ice bath, and *N*‐methylprop‐2‐en‐1‐amine (90 µL, 1.1 mmol, 2.0 eq.) was added. The reaction was stirred for 5 h at 70°C, then cooled to ambient temperature and stirred overnight. After complete conversion of starting materials as monitored by TLC, the mixture was neutralized by the addition of a saturated sodium bicarbonate solution and the aqueous layer was extracted with dichloromethane (3 × 30 mL). The combined organic layer was dried over Na_2_SO_4_, filtered, and concentrated under reduced pressure to obtain the crude product as a yellow oil. The crude product was purified by flash column chromatography (dichloromethane/methanol: gradient 0%–5%) to obtain the product as a yellow resin (140 mg, 0.347 mmol, 65%).

*R*_f_ = 0.25 (dichloromethane/methanol: 19:1); ^1^H NMR (500 MHz, DMSO‐*d*_6_ δ [ppm]): 7.76–7.69 (m, 2H, 4‐chlorophenyl, H‐2,6), 7.64–7.58 (m, 2H, 4‐chlorophenyl, H‐3,5), 7.55 (dd, 1H, *J* = 8.6, 8.6 Hz, 2‐fluorophenyl, H‐6), 6.96 (dd, 1H, *J* = 12.7, 2.4 Hz, 2‐fluorophenyl, H‐3), 6.93 (dd, 1H, *J* = 8.6, 2.4 Hz, 2‐fluorophenyl, H‐5), 5.85–5.75 (m, 1H, –HC═CH_2_), 5.22–5.10 (m, 2H, –HC═CH_2_), 4.08 (t, 2H, *J* = 6.5 Hz, –O–CH_2_–(CH_2_)_5_–N(CH_3_)–), 2.99 (d, 2H, *J* = 6.2 Hz, –N(CH_3_)–CH_2_–HC═CH_2_), 2.32 (t, 2H, *J* = 7.4 Hz, –O–(CH_2_)_5_–CH_2_–N(CH_3_)–), 2.15 (s, 3H, –N(CH_3_)–CH_2_–HC═CH_2_), 1.73 (p, 2H, *J* = 6.7, –O–CH_2_–CH_2_–(CH_2_)_4_–N(CH_3_)–), 1.50– 1.37 (m, 4H, –O–(CH_2_)_2_–CH_2_–CH_2_–CH_2_–CH_2_–N(CH_3_)–), 1.37–1.25 (m, 2H, –O–(CH_2_)_3_–CH_2_–(CH_2_)_2_–N(CH_3_)–); ^13^C NMR (126 MHz, DMSO‐*d*_*6*_, δ [ppm]): 190.72 q (C═O), 163.36 q (d, *J* = 11.5 Hz, 2‐fluorophenyl, C‐4), 161.26 q (d, *J* = 252 Hz, 2‐fluorophenyl, C‐2), 137.99 q (4‐chlorophenyl, C‐4), 136.54 q (4‐chlorophenyl, C‐1), 135.41* (–N(CH_3_)–CH_2_–HC═CH_2_), 132.44 (d, *J* = 4.3 Hz, 2‐fluorophenyl, C‐6), 130.97 (4‐chlorophenyl, C‐2,6), 128.76 (4‐chlorophenyl, C‐3,5), 117.90 q (d, *J* = 13.5 Hz, 2‐fluorophenyl, C‐1), 117.51 (–N(CH_3_)–CH_2_–HC═CH_2_), 111.39 (d, *J* = 2.6 Hz, 2‐fluorophenyl, C‐5), 102.35 (d, *J* = 25.5 Hz, 2‐fluorophenyl, C‐3), 68.48 (–O–CH_2_–(CH_2_)_5_–N(CH_3_)–), 60.05 (–N(CH_3_)–CH_2_–HC═CH_2_), 56.29 (–O–(CH_2_)_5_–CH_2_–N(CH_3_)–), 41.43 (–N(CH_3_)–CH_2_–HC═CH_2_), 28.35 (–O–CH_2_–CH_2_–(CH_2_)_4_–N(CH_3_)–), 26.44, 26.38 (–O–(CH_2_)_3_–(CH_2_)_2_–CH_2_–N(CH_3_)–), 25.26 (–O–(CH_2_)_2_–CH_2_–(CH_2_)_3_–N(CH_3_)–); LRMS (APCI^+^): *m/z* 404 [M + H]^+^; HRMS (ESI^+^): *m/z* calcd for C_23_H_28_ClFNO_2_^+^: 404.17938 [M + H]^+^, found: 404.17897; HPLC retention time: 18.81 min, 98% (M1).

#### 4.1.7 Synthesis of [4‐({6‐[allyl(methyl)amino]hexyl}oxy)‐2‐fluorophenyl](phenyl)methanone (**4e**)

{4‐[(6‐Bromohexyl)oxy]‐2‐fluorophenyl}(phenyl)methanone (**8c**, 150 mg, 0.396 mmol, 1.0 eq.) was dissolved in dimethylacetamide (2.0 mL). The solution was cooled in an ice bath, and *N*‐methylprop‐2‐en‐1‐amine (100 µL, 1.04 mmol, 2.6 eq.) was added. The reaction was stirred for 21 h at room temperature. After complete conversion of starting materials as monitored by TLC, the mixture was neutralized by the addition of a saturated sodium bicarbonate solution and the aqueous layer was extracted with dichloromethane (3 × 50 mL). The combined organic layer was dried over Na_2_SO_4_, filtered, and concentrated under reduced pressure to obtain the crude product as a yellow oil. The crude product was purified by flash column chromatography (dichloromethane/methanol: gradient 0%–10%) to obtain the product as a pale‐yellow oil (115 mg, 0.311 mmol, 79%).

*R*_f_ = 0.14 (dichloromethane/methanol: 20:1); ^1^H NMR (500 MHz, DMSO‐*d*_*6*_, δ [ppm]): 7.75–7.70 (m, 2H, phenyl, H‐2,6), 7.69– 7.64 (m, 1H, phenyl, H‐4), 7.58–7.50 (m, 3H, phenyl, H‐3,5, 2‐fluorophenyl, H‐6), 6.96 (dd, *J* = 12.5, 2.4 Hz, 1H, 2‐fluorophenyl, H‐3), 6.93 (dd, *J* = 8.5, 2.4 Hz, 1H, 2‐fluorophenyl, H‐5), 5.87–5.75 (m, 1H, –HC═CH_2_), 5.26–5.09 (m, 2H, –HC═CH_2_), 4.08 (t, *J* = 6.5 Hz, 2H, –O–CH_2_–(CH_2_)_5_–N(CH_3_)–), 3.01 (d, *J* = 6.4 Hz, 2H, –N(CH_3_)–CH_2_–HC═CH_2_), 2.35 (t, *J* = 7.1 Hz, 2H, –O–(CH_2_)_5_–CH_2_–N(CH_3_)–), 2.17 (s, 3H, –N(CH_3_)–CH_2_–HC═CH_2_), 1.74 (p, *J* = 6.7 Hz, 2H, –O–CH_2_–CH_2_–(CH_2_)_4_–N(CH_3_)–), 1.50–1.38 (m, 4H, –O–(CH_2_)_2_–CH_2_–CH_2_–CH_2_–CH_2_–N(CH_3_)–), 1.38–1.28 (m, 2H, –O–(CH_2_)_3_–CH_2_–(CH_2_)_2_–N(CH_3_)‐); ^13^C NMR (126 MHz, DMSO‐*d*_*6*_, δ [ppm]): 191.83 q (C═O), 163.02 q (d, *J* = 11.6 Hz, 2‐fluorophenyl, C‐4), 161.12 q (d, *J* = 251 Hz, 2‐fluorophenyl, C‐2), 137.70 q (phenyl, C‐1), 135.26 (–N(CH_3_)–CH_2_–HC═CH_2_), 133.06 (phenyl, C‐2,6), 132.26 (d, *J* = 4.6 Hz, 2‐fluorophenyl, C‐6), 129.04 (phenyl, C‐4), 128.52 (phenyl, C‐3,5), 118.26 q (d, *J* = 13.8 Hz, 2‐fluorophenyl, C‐1), 117.64 (–N(CH_3_)–CH_2_–HC═CH_2_), 111.18 (d, *J* = 2.7 Hz, 2‐fluorophenyl, C‐5), 102.23 (d, *J* = 25.6 Hz, 2‐fluorophenyl, C‐3), 68.35 (–O–CH_2_–(CH_2_)_5_–N(CH_3_)–), 59.92 (–N(CH_3_)–CH_2_–HC═CH_2_), 56.18 (–O–(CH_2_)_5_–CH_2_‐N(CH_3_)–), 41.30 (–N(CH_3_)–CH_2_–HC═CH_2_), 28.28 (–O–CH_2_–CH_2_–(CH_2_)_4_–N(CH_3_)–), 26.35, 26.24 (–O–(CH_2_)_3_–(CH_2_)_2_–CH_2_–N(CH_3_)–), 25.19 (–O–(CH_2_)_2_–CH_2_–(CH_2_)_3_–N(CH_3_)–); LRMS (APCI^+^): *m/*z 370 [M + H]^+^; HRMS (ESI^+^): *m/z* calcd for C_23_H_29_FNO_2_^+^: 370.21768 [M + H]^+^, found: 370.21780; HPLC retention time: 17.59 min, 98% (M1).

#### 4.1.8 Synthesis of (4‐chlorophenyl)[2‐fluoro‐4‐({6‐[methyl(prop‐2‐yn‐1‐yl)amino]hexyl}oxy)phenyl]methanone (**4 f**, MSHG_019)

{4‐[(6‐Bromohexyl)oxy]‐2‐fluorophenyl}(4‐chlorophenyl)methanone (**8b**, 222 mg, 0.537 mmol, 1.0 eq.) was dissolved in dimethylacetamide (2.0 mL). The solution was cooled in an ice bath, and *N*‐methylprop‐2‐yn‐1‐amine (90 µL, 1.1 mmol, 2.0 eq.) was added. The reaction was stirred for 5 h at 70°C, then cooled to ambient temperature and stirred overnight. After complete conversion of starting materials as monitored by TLC, the mixture was neutralized by the addition of a saturated sodium bicarbonate solution and the aqueous layer was extracted with dichloromethane (3 × 30 mL). The combined organic layer was dried over Na_2_SO_4_, filtered, and concentrated under reduced pressure to obtain the crude product as a yellow oil. The crude product was purified by flash column chromatography (petroleum ether/ethyl acetate: gradient 20%–60%) to obtain the product as a pale‐yellow resin (162 mg, 0.537 mmol, 75%).

*R*_f_ = 0.25 (petroleum ether/ethyl acetate: 7:3); ^1^H NMR (500 MHz, DMSO‐*d*_6_ δ [ppm]): 7.77–7.70 (m, 2H, 4‐chlorophenyl, H‐2,6), 7.64–7.58 (m, 2H, 4‐chlorophenyl, H‐3,5), 7.55 (dd, 1H, *J* = 8.6, 8.6 Hz, 2‐fluorophenyl, H‐6), 6.97 (dd, 1H, *J* = 12.7, 2.4 Hz, 2‐fluorophenyl, H‐3), 6.93 (dd, 1H, *J* = 8.6, 2.4 Hz, 2‐fluorophenyl, H‐5), 4.08 (t, 2H, *J* = 6.5 Hz, –O–CH_2_–(CH_2_)_5_–N(CH_3_)–), 3.27 (d, 2H, *J* = 2.4 Hz, –N(CH_3_)–CH_2_–C ≡ CH) 3.08 (t, 1H, *J* = 2.4 Hz, –C ≡ CH), 2.32 (t, 2H, *J* = 7.1 Hz, –O‐(CH_2_)_5_–CH_2_–N(CH_3_)–), 2.17 (s, 3H, –N(CH_3_)‐CH_2_–C ≡ CH), 1.78–1.69 (m, 2H, –O–CH_2_–CH_2_–(CH_2_)_4_‐N(CH_3_)–), 1.48–1.36 (m, 4H, –O–(CH_2_)_2_–CH_2_–CH_2_–CH_2_–CH_2_–N(CH_3_)–), 1.36–1.29 (m, 2H, –O–(CH_2_)_3_–CH_2_–(CH_2_)_2_–N(CH_3_)–); ^13^C NMR (126 MHz, DMSO‐*d*_*6*_, δ [ppm]): 190.72 q (C=O), 163.36 q (d, *J* = 11.6 Hz, 2‐fluorophenyl, C‐4), 161.26 q (d, *J* = 252 Hz, 2‐fluorophenyl, C‐2), 137.98 q (4‐chlorophenyl, C‐4), 136.54 q (4‐chlorophenyl, C‐1), 132.44 (d, *J* = 4.2 Hz, 2‐fluorophenyl, C‐6), 130.97 (4‐chlorophenyl, C‐2,6), 128.75 (4‐chlorophenyl, C‐3,5), 117.89 q (d, *J* = 13.6 Hz, 2‐fluorophenyl, C‐1), 111.39 (d, *J* = 2.6 Hz, 2‐fluorophenyl, C‐5), 102.35 (d, *J* = 25.5 Hz, 2‐fluorophenyl, C‐3), 79.07 q (–N(CH_3_)–CH_2_–C ≡ CH), 75.54 (–N(CH_3_)–CH_2_–C ≡ CH), 68.50 (–O–CH_2_–(CH_2_)_5_–N(CH_3_)–), 54.84 (–O–(CH_2_)_5_–CH_2_–N(CH_3_)–) 44.88 (–N(CH_3_)–CH_2_–C ≡ CH), 41.27 (–N(CH_3_)–CH_2_–C ≡ CH) 28.37 (–O–CH_2_‐CH_2_–(CH_2_)_4_–N(CH_3_)–), 26.80, 26.48 (–O–(CH_2_)_3_–(CH_2_)_2_–CH_2_–N(CH_3_)–), 25.27 (–O–(CH_2_)_2_–CH_2_–(CH_2_)_3_–N(CH_3_)–); LRMS (APCI^+^): *m/z* 402 [M + H]^+^; HRMS (ESI^+^): *m/z* calcd for C_23_H_26_ClFNO_2_^+^: 402.16372 [M + H]^+^, found: 402.16330; HPLC retention time: 18.72 min, 99% (M1).

#### 4.1.9 Synthesis of (4‐{[6‐(allyloxy)hexyl]oxy}‐2‐fluorophenyl)(4‐bromophenyl)methanone (5)

To a mixture of (4‐bromophenyl)(2‐fluoro‐4‐hydroxyphenyl)methanone (**7a**, 72 mg, 0.24 mmol, 1.0 eq.) and potassium carbonate (101 mg, 0.732 mmol, 3.0 eq.) in acetone (3.0 mL), 1‐(allyloxy)‐6‐bromohexane (**9**, 162 mg, 0.732 mmol, 3.0 eq.) was added. The reaction mixture was stirred for 2.5 h at 75°C. After complete conversion of starting materials as monitored by TLC, the reaction mixture was filtered and concentrated under reduced pressure to obtain the crude product. The crude product was purified *by* flash column chromatography (petroleum ether/ethyl acetate: gradient 0%–10%) to obtain the product as a white oil (102 mg, 0.234 mmol, 96%).

*R*_f_ = 0.35 (petroleum ether/ethyl acetate: 10:1); ^1^H NMR (500 MHz, DMSO‐*d*_*6*_, δ [ppm]): 7.78–7.73 (m, 2H, 4‐bromophenyl, H‐2,6), 7.67–7.63 (m, 2H, 4‐bromophenyl, H‐3,5), 7.55 (dd, *J* = 8.6, 8.6 Hz, 1H, 2‐fluorophenyl, H‐6), 6.97 (dd, *J* = 12.6, 2.3 Hz, 1H, 2‐fluorophenyl, H‐3), 6.93 (dd, *J* = 8.6, 2.3 Hz, 1H, 2‐fluorophenyl, H‐5), 5.92–5.82 (m, 1H, –HC═CH_2_), 5.26–5.09 (m, 2H, –HC═CH_2_), 4.08 (t, *J* = 6.7 Hz, 2H, aryl–O–CH_2_–(CH_2_)_5_–O–), 3.91 (dt, *J* = 5.4, 1.5 Hz, 2H, –O–CH_2_–HC═CH_2_), 3.38 (t, *J* = 6.5 Hz, 2H, aryl–O–(CH_2_)_5_–CH_2_–O–), 1.79–1.69 (m, 2H, aryl–O–CH_2_–CH_2_–(CH_2_)_4_–O–), 1.58–1.49 (m, 2H, aryl–O–(CH_2_)_4_–CH_2_–CH_2_–O–), 1.47–1.33 (m, 4H, aryl–O–(CH_2_)_2_–(CH_2_)_2_–(CH_2_)_2_–O–); ^13^C NMR (126 MHz, DMSO‐*d*_*6*_, δ [ppm]): 190.83 q (C═O), 163.29 q (d, *J* = 11.6 Hz, 2‐fluorophenyl, C‐4), 161.19 q (d, *J* = 252 Hz, 2‐fluorophenyl, C‐2), 136.80 q (4‐bromophenyl, C‐1), 135.40 (–O–CH_2_–HC=CH_2_), 132.37 (d, *J* = 4.2 Hz, 2‐fluorophenyl, C‐6), 131.62 (4‐bromophenyl, C‐2,6), 130.98 (4‐bromophenyl, C‐3,5), 127.05 q (4‐bromophenyl, C‐4), 117.78 q (d, *J* = 13.4 Hz, 2‐fluorophenyl, C‐1), 115.95 (–O–CH_2_–HC═CH_2_), 111.31 (d, *J* = 2.7 Hz, 2‐fluorophenyl, C‐5), 102.27 (d, *J* = 25.7 Hz, 2‐fluorophenyl, C‐3), 70.68 (–O–CH_2_–HC═CH_2_), 69.34 (aryl–O–CH_2_–(CH_2_)_5_–O–), 68.39 (aryl–O–(CH_2_)_5_–CH_2_–O–), 29.04, 28.26, 25.32, 25.14 (aryl–O–CH_2_–(CH_2_)_4_–CH_2_–O–); LRMS (APCI^+^): *m/z* 435 [M + H]^+^; HRMS (ESI^+^): *m/z* calcd for C_22_H_24_BrFO_3_^+^: 435.09656 [M + H]^+^, found: 435.09675; HPLC retention time: 24.16 min, 98% (M1).

#### 4.1.10 Synthesis of (4‐bromophenyl)(2‐fluoro‐4‐methoxyphenyl)methanone (**6a**) [45, 46]

1‐Fluoro‐3‐methoxybenzene (1.13 mL, 9.91 mmol, 1.0 eq.) and 4‐bromobenzoyl chloride (1.70 mL, 12.9 mmol, 1.3 eq.) were dissolved in dry dichloromethane (40 mL) in a round‐bottom flask. The solution was cooled in an ice bath, and aluminium chloride (1.99 g, 14.9 mmol, 1.5 eq.) was added portion wise. The reaction mixture was stirred for 24 h at ambient temperature under dry conditions. After complete conversion of starting materials as monitored by TLC, the reaction was quenched by the addition of water (80 mL). The reaction mixture was extracted with ethyl acetate (3 × 50 mL), then dried over Na_2_SO_4_, filtered, and concentrated under reduced pressure to obtain the crude product. The crude product was purified by flash column chromatography (petroleum ether/ethyl acetate: gradient 0%–18%) to obtain the product as a white solid. (1.16 g, 3.74 mmol, 47%).

*R*_f_ = 0.44 (petroleum ether/ethyl acetate: 9:1); ^1^H NMR (500 MHz, DMSO‐*d*_6_ *δ* [ppm]): 7.78–7.74 (m, 2H, 4‐bromophenyl, H‐2,6), 7.68–7.63 (m, 2H, 4‐bromophenyl, H‐3,5), 7.57 (dd, 1H, *J* = 8.5, 8.5 Hz, 2‐fluoro‐4‐methoxyphenyl, H‐6), 6.99 (dd, 1H, *J* = 12.6, 2.4 Hz, 2‐fluoro‐4‐methoxyphenyl, H‐3), 6.95 (dd, 1H, *J* = 8.6, 2.4 Hz, 2‐fluoro‐4‐methoxyphenyl, H‐5), 3.87 (s, 3H, –O–CH_3_); ^13^C NMR (126 MHz, DMSO‐*d*_*6*_, δ [ppm]): 190.92 q (C═O), 163.93 q (d, *J* = 11.7 Hz, 2‐fluoro‐4‐methoxyphenyl, C‐4), 161.26 q (d, *J* = 252 Hz, 2‐fluoro‐4‐methoxyphenyl, C‐2), 136.84 q (4‐bromophenyl, C‐1), 132.45 (d, *J* = 4.3 Hz, 2 fluoro‐4‐methoxyphenyl, C‐6), 131.71 (4‐bromophenyl, C‐2,6), 131.08 (4‐bromophenyl, C‐3,5), 127.17 q (4‐bromophenyl, C‐4) 118.02 q (d, *J* = 13.7 Hz, 2‐fluoro‐4‐methoxyphenyl, C‐1), 111.05 (d, *J* = 2.6 Hz, 2‐fluoro‐4‐methoxyphenyl, C‐5), 102.02 (d, *J* = 25.5 Hz, 2‐fluoro‐4‐methoxyphenyl, C‐3), 56.13 (–O–CH_3_); LRMS (APCI^+^): *m/z* 309 [M + H]^+^. The obtained analytical data are in a good agreement with literature values. [45, 46].

#### 4.1.11 Synthesis of (4‐chlorophenyl)(2‐fluoro‐4‐methoxyphenyl)methanone (**6b**) [47]

1‐Fluoro‐3‐methoxybenzene (0.906 mL, 7.93 mmol, 1.0 eq.) and 4‐chlorobenzoyl chloride (1.22 mL, 9.51 mmol, 1.2 eq.) were dissolved in dry dichloromethane (20 mL) in a round‐bottom flask. The solution was cooled in an ice bath, and aluminium chloride (1.59 g, 11.9 mmol, 1.5 eq.) was added portion wise. The reaction mixture was stirred for 24 h at ambient temperature under dry conditions. After complete conversion of starting materials as monitored by TLC, the reaction was quenched with water (40 mL). The reaction mixture was extracted with ethyl acetate (1 × 40 mL). The organic layer was washed with saturated sodium bicarbonate solution (20 mL) and brine (20 mL), then dried over Na_2_SO_4_, filtered, and concentrated under reduced pressure to obtain the crude product. The crude product was purified by flash column chromatography (petroleum ether/ethyl acetate: gradient 0%–18%) to obtain the product as a white solid. (985 mg, 3.72 mmol, 47%).

*R*_f_ = 0.40 (petroleum ether/ethyl acetate: 9:1); ^1^H NMR (500 MHz, DMSO‐*d*_6_ δ [ppm]): 7.75–7.71 (m, 2H, 4‐chlorophenyl, H‐2,6), 7.64 – 7.60 (m, 2H, 4‐chlorophenyl, H‐3,5), 7.57 (dd, 1H, *J* = 8.6, 8.6 Hz, 2‐fluoro‐4‐methoxyphenyl, H‐6), 6.99 (dd, 1H, *J* = 12.6, 2.4 Hz, 2‐fluoro‐4‐methoxyphenyl, H‐3), 6.95 (dd, 1H, *J* = 8.6, 2.4 Hz, 2‐fluorophenyl, H‐5), 3.87 (s, 3H, –O–CH_3_); ^13^C NMR (126 MHz, DMSO‐*d*_*6*_, δ [ppm]): 190.74 q (C═O), 163.91 q (d, *J* = 11.6 Hz, 2‐fluoro‐4‐methoxyphenyl, C‐4), 161.24 q (d, *J* = 252 Hz, 2‐fluoro‐4‐methoxyphenyl, C‐2), 138.03 q (4‐chlorophenyl, C‐4), 136.50 q (4‐chlorophenyl, C‐1), 132.44 (d, *J* = 4.3 Hz, 2‐fluoro‐4‐methoxyphenyl, C‐6), 130.99 (4‐chlorophenyl, C‐2,6), 128.77 (4‐chlorophenyl, C‐3,5), 118.06 q (d, *J* = 13.6 Hz, 2‐fluorophenyl, C‐1), 111.04 (d, *J* = 2.7 Hz, 2‐fluoro‐4‐methoxyphenyl, C‐5), 102.02 (d, *J* = 25.6 Hz, 2‐fluoro‐2‐methoxyphenyl, C‐3), 56.13 (‐O‐CH_3_); LRMS (APCI^+^): *m/z* 265 [M + H]^+^. The obtained analytical data are in a good agreement with literature values [47].

#### 4.1.12 Synthesis of (2‐fluoro‐4‐methoxyphenyl)(phenyl)methanone (**6c**) [48]

1‐Fluoro‐3‐methoxybenzene (0.906 mL, 7.93 mmol, 1.0 eq.) and benzoyl chloride (1.11 mL, 9.51 mmol, 1.2 eq.) were dissolved in dry dichloromethane (20 mL) in a round‐bottom flask. The solution was cooled in an ice bath, and aluminium chloride (1.59 g, 11.9 mmol, 1.5 eq.) was added portion wise. The reaction mixture was stirred for 2 h at ambient temperature under dry conditions. After complete conversion of starting materials as monitored by TLC, the reaction was quenched with water (30 mL). The reaction mixture was extracted with ethyl acetate (3 × 40 mL). The organic layer was washed with saturated sodium bicarbonate solution (20 mL) and brine (20 mL), then dried over Na_2_SO_4_, filtered, and concentrated under reduced pressure to obtain the crude product. The crude product was purified by flash column chromatography (petroleum ether/ethyl acetate: gradient 0%–10%) to obtain the product as a white solid (985 mg, 3.72 mmol, 47%).

*R*_f_ = 0.61 (petroleum ether/ethyl acetate: 4:1); ^1^H NMR (600 MHz, DMSO‐*d*_*6*_, δ [ppm]): 7.74–7.72 (m, 2H), 7.69–7.66 (m, 2H), 7.56–7.54 (m, 2H), 6.98 (dd, *J* = 12.5, 2.4 Hz, 1H), 6.95 (dd, *J* = 6.1, 2.5 Hz, 1H), 3.88 (s, 3H). LRMS (APCI^+^): *m/z* 231 [M + H]^+^. The obtained analytical data are in good agreement with literature values [48].

#### 4.1.13 Synthesis of (4‐bromophenyl)(2‐fluoro‐4‐hydroxyphenyl)methanone (**7a**) [45, 46]

(4‐Bromophenyl)(2‐fluoro‐4‐methoxyphenyl)methanone (**6a**, 1.10 g, 3.56 mmol, 1.0 eq.) was dissolved in dry dichloromethane (20 mL) in a round‐bottom flask. The solution was cooled in an ice bath, and boron tribromide (1 M solution in dichloromethane, 10.7 mL, 10.7 mmol, 3.0 eq.) was added portion wise. The reaction mixture was stirred for 18 h at ambient temperature under dry conditions. The reaction mixture was then cooled in an ice bath and boron tribromide (1 M solution in dichloromethane, 3.56 mL, 3.56 mmol, 1.0 eq.) was added. After complete conversion of starting materials as monitored by TLC, the reaction mixture was cooled on an ice bath and water (50 mL) was carefully added. The reaction mixture was extracted with ethyl acetate (1 × 50 mL). The organic layer was washed with water (50 mL) and brine (50 mL), then dried over Na_2_SO_4_, filtered and concentrated under reduced pressure to obtain the crude product. The crude product was purified by flash column chromatography (petroleum ether/ethyl acetate: gradient 10%–30%) to obtain the product as a white solid (872 mg, 2.96 mmol, 83%).

*R*_f_ = 0.32 (petroleum ether/ethyl acetate: 4:1); ^1^H NMR (500 MHz, DMSO‐*d*_6_ *δ* [ppm]): 10.80 (s, 1H, –OH), 7.77–7.71 (m, 2H, 4‐bromophenyl, H‐2,6), 7.67–7.60 (m, 2H, 4‐bromophenyl, H‐3,5), 7.48 (dd, 1H, *J* = 8.6, 8.6 Hz, 2‐fluoro‐4‐hydroxyphenyl, H‐6), 6.76 (dd, 1H, *J* = 8.6, 2.3 Hz, 2‐fluoro‐4‐hydroxyphenyl, H‐5), 6.67 (dd, 1H, *J* = 12.5, 2.3 Hz, 2‐fluoro‐4‐hydroxyphenyl, H‐3); ^13^C NMR (126 MHz, DMSO‐*d*_*6*_, δ [ppm]): 190.88 q (C═O), 163.03 q (d, *J* = 12.2 Hz, 2‐fluoro‐4‐hydroxyphenyl, C‐4), 161.51 q (d, *J* = 252 Hz, 2‐fluoro‐4‐hydroxyphenyl, C‐2), 137.20 q (4‐bromophenyl, C‐1), 132.87 (d, *J* = 4.3 Hz, 2 fluoro‐4‐hydroxyphenyl, C‐6), 131.61 (4‐bromophenyl, C‐2,6), 131.00 (4‐bromophenyl, C‐3,5), 126.84 q (4‐bromophenyl, C‐4) 116.61 q (d, *J* = 13.0 Hz, 2‐fluoro‐4‐hydroxyphenyl, C‐1), 112.10 (d, *J* = 2.4 Hz, 2‐fluoro‐4‐hydroxyphenyl, C‐5), 103.01 (d, *J* = 24.1 Hz, 2‐fluoro‐4‐hydroxyphenyl, C 3); LRMS (ESI^‐^): *m/z* 294 [M‐H]^‐^. The obtained analytical data are in a good agreement with literature values [45, 46].

#### 4.1.14 Synthesis of (4‐chlorophenyl)(2‐fluoro‐4‐hydroxyphenyl)methanone (**7b**) [49, 50]

(4‐Chlorophenyl)(2‐fluoro‐4‐methoxyphenyl)methanone (**6b**, 800 mg, 3.02 mmol, 1.0 eq.) was dissolved in dry dichloromethane (13 mL) in a round‐bottom flask. The solution was cooled in an ice bath, and boron tribromide (1 M solution in dichloromethane, 9.07 mL, 9.07 mmol, 3.0 eq.) was added portion wise. The reaction mixture was stirred for 22 h at ambient temperature under dry conditions. After complete conversion of starting materials as monitored by TLC, the reaction mixture was cooled in an ice bath and water (25 mL) was carefully added. The reaction mixture was extracted with ethyl acetate (1 × 40 mL). The organic layer was washed with water (40 mL) and brine (40 mL), then dried over Na_2_SO_4_, filtered, and concentrated under reduced pressure to obtain the crude product. The crude product was purified by flash column chromatography (petroleum ether/ethyl acetate: gradient 10%–60%) to obtain the product as a white solid (667 mg, 2.66 mmol, 88%).

*R*_f_ = 0.40 (petroleum ether/ethyl acetate: 3:1); ^1^H NMR (500 MHz, DMSO‐*d*_6_ *δ* [ppm]): 10.80 (s, 1H, 2‐fluoro‐4‐hydroxyphenyl, –OH), 7.73–7.69 (m, 2H, 4‐chlorophenyl, H‐2,6), 7.61–7.58 (m, 2H, 4‐chlorophenyl, H‐3,5), 7.48 (dd, 1H, *J* = 8.6, 8.6 Hz, 2‐fluoro‐4‐hydroxyphenyl, H‐6), 6.76 (dd, 1H, *J* = 8.6, 2.2 Hz, 2‐fluoro‐4‐hydroxyphenyl, H‐5), 6.67 (dd, 1H, *J* = 12.5, 2.2 Hz, 2‐fluoro‐4‐hydroxyphenyl, H‐3); ^13^C NMR (126 MHz, DMSO‐*d*_*6*_, *δ* [ppm]): 190.71 q (C═O), 163.02 q (d, *J* = 12.4 Hz, 2‐fluoro‐4‐hydroxyphenyl, C‐4), 161.50 q (d, *J* = 252 Hz, 2‐fluoro‐4‐hydroxyphenyl, C‐2), 137.73 q (4‐chlorophenyl, C‐4), 136.86 q (4‐chlorophenyl, C‐1), 132.86 (d, *J* = 4.4 Hz, 2‐fluoro‐4‐hydroxyphenyl, C‐6), 130.90 (4‐chlorophenyl, C‐2,6), 128.68 (4‐chlorophenyl, C‐3,5), 116.65 q (d, *J* = 13.1 Hz, 2‐fluoro‐4‐hydroxyphenyl, C‐1), 112.10 (d, *J* = 2.5 Hz, 2‐fluoro‐4‐hydroxyphenyl, C‐5), 103.01 (d, *J* = 24.1 Hz, 2‐fluoro‐4‐hydroxyphenyl, C‐3); LRMS (APCI^+^): *m/z* 251 [M + H]^+^.

The compound is described in patent literature. However, only LRMS data were reported, which is in good agreement with our experimental data [49, 50]. To provide further experimental data on compound characterization, we attached ^1^H and ^13^C NMR spectra to the Supporting Information.

#### 4.1.15 Synthesis of (2‐fluoro‐4‐hydroxyphenyl)(phenyl)methanone (**7c**)

(2‐Fluoro‐4‐methoxyphenyl)(phenyl)methanone (**6c**, 600 mg, 2.61 mmol, 1.0 eq.) was dissolved in dry dichloromethane (7.5 mL) in a round‐bottom flask. The solution was cooled in an ice bath, and boron tribromide (1 M solution in dichloromethane, 10.4 mL, 10.4 mmol, 4.0 eq.) was added portion wise. The reaction mixture was stirred for 22 h at ambient temperature under dry conditions. After complete conversion of starting materials as monitored by TLC, the reaction mixture was cooled on an ice bath and water (25 mL) was carefully added. The reaction mixture was extracted with ethyl acetate (1 × 40 mL). The organic layer was washed with water (40 mL) and brine (40 mL), then dried over Na_2_SO_4_, filtered, and concentrated under reduced pressure to obtain the crude product. The crude product was purified by flash column chromatography (petroleum ether/ethyl acetate: gradient 0%–25%) to obtain the product as a white solid (735 mg, 3.40 mmol, quantitative yield).

*R*_f_ = 0.62 (petroleum ether/ethyl acetate: 3:2); ^1^H NMR (600 MHz, DMSO‐*d*_6_ δ [ppm]): 10.75 (s, 1H, 2‐fluoro‐4‐hydroxyphenyl, –OH), 7.72–7.69 (m, 2H, phenyl, H‐2,6), 7.67–7.63 (m, 1H, phenyl, H‐4), 7.55–7.51 (m, 2H, phenyl, H‐3,5), 7.46 (dd, *J* = 8.5, 8.5 Hz, 1H, 2‐fluoro‐4‐hydroxyphenyl, H‐6), 6.76 (dd, *J* = 8.5, 2.3 Hz, 1H, 2‐fluoro‐4‐hydroxyphenyl, H‐5), 6.67 (dd, *J* = 12.5, 2.3 Hz, 1H, 2‐fluoro‐4‐hydroxyphenyl, H‐3); ^13^C NMR (151 MHz, DMSO‐*d*_*6*_, δ [ppm]): 191.84 q (C═O), 162.64 q (d, *J* = 12.1 Hz, 2‐fluoro‐4‐hydroxyphenyl, C‐4), 161.35 q (d, *J* = 252 Hz, 2‐fluoro‐4‐hydroxyphenyl, C‐2), 138.01 q (phenyl, C‐1), 132.82 (phenyl, C‐2,6), 132.68 (d, *J* = 4.5 Hz, 2‐fluoro‐4‐hydroxyphenyl, C‐6), 128.97 (phenyl, C‐4), 128.44 (phenyl, C‐3,5), 116.98 q (d, *J* = 13.0 Hz, 2‐fluoro‐4‐hydroxyphenyl, C‐1), 111.89 (d, *J* = 2.5 Hz, 2‐fluoro‐4‐hydroxyphenyl, C‐5), 102.88 (d, *J* = 24.2 Hz, 2‐fluoro‐4‐hydroxyphenyl, C‐3); LRMS (APCI^+^): *m/z* 217 [M + H]^+^.

#### 4.1.16 Synthesis of {4‐[(6‐bromohexyl)oxy]‐2‐fluorophenyl}(4‐bromophenyl)methanone (8a) [45]

For the synthesis of **8a** we referred to a protocol reported by Morand et al. To a mixture of 1,6‐dibromohexane (0.924 mL, 6.10 mmol, 3.0 eq.) and potassium carbonate (843 mg, 6.10 mmol, 3.0 eq.) in acetone (12 mL), (4‐bromophenyl)(2‐fluoro‐4‐hydroxyphenyl)methanone (**7a**, 600 mg, 2.03 mmol, 1.0 eq.) in acetone (8.0 mL) was added dropwise over a period of 30 min. The reaction mixture was stirred for 5 h at 75°C. After complete conversion of starting materials as monitored by TLC, the reaction mixture was filtered and concentrated under reduced pressure. The residue was dissolved in dichloromethane, treated with Na_2_SO_4_, filtered again, and concentrated under reduced pressure to obtain the crude product. The crude product was purified by flash column chromatography (petroleum ether/ethyl acetate: gradient 0%–20%) to obtain the product as a white resin (700 mg, 1.53 mmol, 75%).

*R*_f_ = 0.59 (petroleum ether/ethyl acetate: 9:1);

^1^H NMR (600 MHz, DMSO‐*d*_6_ δ [ppm]): 7.80–7.73 (m, 2H, 4‐bromophenyl, H‐2,6), 7.68–7.62 (m, 2H, 4‐bromophenyl, H‐3,5), 7.55 (dd, 1H, *J* = 8.6, 8.6 Hz, 2‐fluorophenyl, H‐6), 6.97 (dd, 1H, *J* = 12.6, 2.4 Hz, 2‐fluorophenyl, H‐3), 6.93 (dd, 1H, *J* = 8.6, 2.4 Hz, 2‐fluorophenyl, H‐5), 4.09 (t, 2H, *J* = 6.5 Hz, –O–CH_2_–(CH_2_)_5_–Br), 3.54 (t, 2H, *J* = 6.7 Hz, –O–(CH_2_)_5_–CH_2_–Br), 1.87–1.79 (m, 2H, –O–CH_2_–CH_2_–(CH_2_)_4_–Br), 1.79–1.70 (m, 2H, –O–(CH_2_)_4_–CH_2_–CH_2_–Br), 1.50–1.40 (m, 4H, –O–(CH_2_)_2_–(CH_2_)_2_–(CH_2_)_2_–Br; ^13^C NMR (151 MHz, DMSO‐*d*_*6*_, *δ* [ppm]): 190.92 q (C═O), 163.36 q (d, *J* = 11.5 Hz, 2‐fluorophenyl, C‐4), 161.28 q (d, *J* = 252 Hz, 2‐fluorophenyl, C‐2), 136.88 q (4‐bromophenyl, C‐1), 132.48 (d, *J* = 4.2 Hz, 2‐fluorophenyl, C‐6), 131.71 (4‐bromophenyl, C‐2,6), 131.08 (4‐bromophenyl, C‐3,5), 127.15 q (4‐bromophenyl, C‐4) 117.88 q (d, *J* = 13.6 Hz, 2‐fluorophenyl, C‐1), 111.40 (d, *J* = 2.5 Hz, 2‐fluorophenyl, C‐5), 102.37 (d, *J* = 25.5 Hz, 2‐fluorophenyl, C‐3), 68.42 (–O–CH_2_–(CH_2_)_5_–Br), 35.10 (–O–(CH_2_)_5_–CH_2_–Br), 32.14 (–O–CH_2_–CH_2_–(CH_2_)_4_–Br), 28.21, 27.22 (–O–(CH_2_)_3_–CH_2_–CH_2_–CH_2_–Br), 24.54 (–O–(CH_2_)_2_–CH_2_–(CH_2_)_3_–Br); LRMS (APCI^+^): *m/z* 459 [M + H]^+^.

#### 4.1.17 Synthesis of {4‐[(6‐bromohexyl)oxy]‐2‐fluorophenyl}(4‐chlorophenyl)methanone (**8b**)

To a mixture of (4‐chlorophenyl)(2‐fluoro‐4‐hydroxyphenyl)methanone (**7b**, 600 mg, 2.39 mmol, 1.0 eq.) and potassium carbonate (993 mg, 7.18 mmol, 3.0 eq.) in acetone (20 mL), 1,6‐dibromohexane (1.09 mL, 7.18 mmol, 3.0 eq.) was added. The reaction mixture was stirred for 5 h at 75°C. After complete conversion of starting materials as monitored by TLC, the reaction mixture was filtered and concentrated under reduced pressure. The residue was dissolved in dichloromethane, treated with Na_2_SO_4_, filtered again, and concentrated under reduced pressure to obtain the crude product. The crude product was purified by flash column chromatography (petroleum ether/ethyl acetate: gradient 0%–60%) to obtain the product as a white resin (682 mg, 1.65 mmol, 69%).

*R*_f_ = 0.75 (petroleum ether/ethyl acetate: 9:1); ^1^H NMR (500 MHz, DMSO‐*d*_6_ δ [ppm]): 7.76–7.70 (m, 2H, 4‐chlorophenyl, H‐2,6), 7.64–7.59 (m, 2H, 4‐chlorophenyl, H‐3,5), 7.55 (dd, 1H, *J* = 8.6, 8.6 Hz, 2‐fluorophenyl, H‐6), 6.97 (dd, 1H, *J* = 12.7, 2.3 Hz, 2‐fluorophenyl, H‐3), 6.93 (dd, 1H, *J* = 8.6, 2.3 Hz, 2‐fluorophenyl, H‐5), 4.09 (t, 2H, *J* = 6.4 Hz, –O–CH_2_–(CH_2_)_5_–Br), 3.54 (t, 2H, *J* = 6.7 Hz, –O–(CH_2_)_5_–CH_2_–Br), 1.88–1.79 (m, 2H, –O–CH_2_–CH_2_–(CH_2_)_4_‐Br), 1.78–1.64 (m, 2H, –O–(CH_2_)_4_–CH_2_–CH_2_–Br) 1.51– 1.40 (m, 4H, –O–(CH_2_)_2_–(CH_2_ )_2_–(CH_2_)_2_–Br); ^13^C NMR (126 MHz, DMSO‐*d*_*6*_, *δ* [ppm]): 190.72 q (C═O), 163.33 q (d, *J* = 11.6 Hz, 2‐fluorophenyl, C‐4), 161.25 q (d, *J* = 252 Hz, 2‐fluorophenyl, C‐2), 138.01 q (4‐chlorophenyl, C‐4), 136.52 q (4‐chlorophenyl, C‐1), 132.45 (d, *J* = 4.1 Hz, 2‐fluorophenyl, C‐6), 130.98 (4‐chlorophenyl, C‐2,6), 128.76 (4‐chlorophenyl, C‐3,5), 117.92 q (d, *J* = 13.6 Hz, 2‐fluorophenyl, C‐1), 111.39 (d, *J* = 2.6 Hz, 2‐fluorophenyl, C‐5), 102.37 (d, *J* = 25.4 Hz, 2‐fluorophenyl, C‐3), 68.41 (–O–CH_2_–(CH_2_)_5_–Br), 35.08 (–O–(CH_2_)_5_–CH_2_–Br), 32.13 (–O–CH_2_–CH_2_–(CH_2_)_4_‐Br), 28.19, 27.20 (–O–(CH_2_)_3_–(CH_2_)_2_–CH_2_–Br), 24.53 (–O–(CH_2_)_2_–CH_2_–(CH_2_)_3_‐Br); LRMS (APCI^+^): *m/z* 413 [M + H]^+^.

#### 4.1.18 Synthesis of {4‐[(6‐bromohexyl)oxy]‐2‐fluorophenyl}(phenyl)methanone (**8c**)

To a mixture of (2‐fluoro‐4‐hydroxyphenyl)(phenyl)methanone (**7c**, 400 mg, 1.85 mmol, 1.0 eq.) and potassium carbonate (767 mg, 5.55 mmol, 3.0 eq.) in acetone (13 mL), 1,6‐dibromohexane (840 µL, 5.55 mmol, 3.0 eq.) was added. The reaction mixture was stirred for 5 h at 75°C. After complete conversion of starting materials as monitored by TLC, the reaction mixture was filtered and concentrated under reduced pressure. The residue was dissolved in dichloromethane, treated with Na_2_SO_4_, filtered again, and concentrated under reduced pressure to obtain the crude product. The crude product was purified by flash column chromatography (petroleum ether/ethyl acetate: gradient 0%–20%) to obtain the product as a colorless oil (522 mg, 1.37 mmol, 74%).

*R*_f_ = 0.42 (petroleum ether/ethyl acetate: 4:1); ^1^H NMR (500 MHz, DMSO‐*d*_*6*_, δ [ppm]): 7.75–7.70 (m, 2H, phenyl, H‐2,6), 7.70–7.65 (m, 1H, phenyl, H‐4), 7.58–7.50 (m, 3H, phenyl, H‐3,5, 2‐fluorophenyl, H‐6), 6.96 (dd, *J* = 12.5, 2.4 Hz, 1H, 2‐fluorophenyl, H‐3), 6.93 (dd, *J* = 8.5, 2.4 Hz, 1H, 2‐fluorophenyl, H‐5), 4.09 (t, *J* = 6.5 Hz, 2H, –O–CH_2_–(CH_2_)_5_–Br), 3.55 (t, *J* = 6.7 Hz, 2H, –O–(CH_2_)_5_–CH_2_–Br), 1.88–1.79 (m, 2H, –O–CH_2_–CH_2_–(CH_2_)_4_–Br), 1.79–1.68 (m, 2H, –O–(CH_2_)_4_–CH_2_–CH_2_–Br), 1.51–1.37 (m, 4H, –O–(CH_2_)_2_–(CH_2_)_2_–(CH_2_)_2_–Br); ^13^C NMR (126 MHz, DMSO‐*d*_*6*_, *δ* [ppm]): 191.82 q (C═O), 162.98 q (d, *J* = 11.6 Hz, 2‐fluorophenyl, C‐4), 161.09 q (d, *J* = 251 Hz, 2‐fluorophenyl, C‐2), 137.68 q (phenyl, C‐1), 133.04 (phenyl, C‐2,6), 132.24 (d, *J* = 4.6 Hz, 2‐fluorophenyl, C‐6), 129.02 (phenyl, C‐4), 128.50 (phenyl, C‐3,5), 118.26 q (d, *J* = 13.8 Hz, 2‐fluorophenyl, C‐1), 111.16 (d, *J* = 2.7 Hz, 2‐fluorophenyl, C‐5), 102.23 (d, *J* = 25.5 Hz, 2‐fluorophenyl, C‐3), 68.26 (–O–CH_2_–(CH_2_)_5_–Br), 34.98 (–O–(CH_2_)_5_–CH_2_–Br), 32.04, 28.12, 27.11, 24.44 (–O–CH_2_–(CH_2_)_4_–CH_2_–Br); LRMS (APCI^+^): *m/*z 379 [M + H]^+^. The attached ^1^H and ^13^C NMR contain residual solvent signals (DMA).

#### 4.1.19 Synthesis of {4‐[(7‐bromoheptyl)oxy]‐2‐fluorophenyl}(4‐bromophenyl)methanone (**8 d**)

To a mixture of (2‐fluoro‐4‐hydroxyphenyl)(phenyl)methanone (**7a**, 150 mg, 0.508 mmol, 1.0 eq.) and potassium carbonate (211 mg, 1.53 mmol, 3.0 eq.) in acetone (5.0 mL), 1,7‐dibromoheptane (261 µL, 1.53 mmol, 3.0 eq.) was added. The reaction mixture was stirred for 4 h at 75°C. After complete conversion of starting materials as monitored by TLC, the reaction mixture was filtered and concentrated under reduced pressure. The residue was dissolved in dichloromethane, treated with Na_2_SO_4_, filtered again, and concentrated under reduced pressure to obtain the crude product. The crude product was purified *by* flash column chromatography (petroleum ether/ethyl acetate: gradient 0%–15%) to obtain the product as a white solid (172 mg, 0.364 mmol, 72%).

*R*_f_ = 0.52 (petroleum ether/ethyl acetate: 4:1); ^1^H NMR (500 MHz, DMSO‐*d*_*6*_, δ [ppm]): 7.79–7.73 (m, 2H, 4‐bromophenyl, H‐2,6), 7.68–7.62 (m, 2H, 4‐bromophenyl, H‐3,5), 7.55 (dd, *J* = 8.5, 8.5 Hz, 1H, 2‐fluorophenyl, H‐6), 6.97 (dd, *J* = 12.7, 2.4 Hz, 1H, 2‐fluorophenyl, H‐3), 6.93 (dd, *J* = 8.6, 2.4 Hz, 1H, 2‐fluorophenyl, H‐5), 4.09 (t, *J* = 6.5 Hz, 2H, –O–CH_2_–(CH_2_)_6_–Br), 3.54 (t, *J* = 6.7 Hz, 2H, –O–(CH_2_)_6_–CH_2_–Br), 1.81 (p, *J* = 6.8 Hz, 2H, –O–CH_2_–CH_2_–(CH_2_)_5_–Br), 1.78–1.71 (m, 2H, –O–(CH_2_)_5_–CH_2_–CH_2_–Br), 1.47–1.31 (m, 6H, –O–(CH_2_)_2_–‐(CH_2_)_3_–(CH_2_)_2_–Br); ^13^C NMR (126 MHz, DMSO‐*d*_*6*_, δ [ppm]): 190.81 q (C═O), 163.27 q (d, *J* = 11.5 Hz, 2‐fluorophenyl, C‐4), 161.18 q (d, *J* = 252 Hz, 2‐fluorophenyl, C‐2), 136.78 q (4‐bromophenyl, C‐1), 132.36 (d, *J* = 4.2 Hz, 2‐fluorophenyl, C‐6), 131.60 (4‐bromophenyl, C‐2,6), 130.97 (4‐bromophenyl, C‐3,5), 127.03 q (4‐bromophenyl, C‐4), 117.76 q (d, *J* = 13.5 Hz, 2‐fluorophenyl, C‐1), 111.29 (d, *J* = 2.6 Hz, 2‐fluorophenyl, C‐5), 102.26 (d, *J* = 25.5 Hz, 2‐fluorophenyl, C‐3), 68.36 (–O–CH_2_–(CH_2_)_6_–Br), 35.06 (–O–(CH_2_)_6_–CH_2_–Br), 32.05, 28.15, 27.61, 27.32, 25.10 (–O–CH_2_–(CH_2_)_5_–CH_2_–Br); LRMS (APCI^+^): *m/*z 471 [M + H]^+^. The attached ^1^H and ^13^C NMR contain residual solvent signals (DMA).

#### 4.1.20 Synthesis of 1‐(allyloxy)‐6‐bromohexane (**9**) [51]

In a 50 mL flame‐dried, nitrogen‐flushed two‐neck round‐bottom flask sodium hydride (207 mg, 8.61 mmol, 1.25 eq.) (60% dispersion in mineral oil) was dissolved in dry THF (11 mL). Prop‐2‐en‐1‐ol (470 µL, 6.89 mmol, 1.0 eq.) was added dropwise, and the mixture was stirred for 20 min at ambient temperature. 1,6‐Dibromohexane (3.13 mL, 20.7 mmol, 3.0 eq.) was added, and the reaction was stirred for 19 h at ambient temperature under a nitrogen atmosphere. After complete conversion of starting materials as monitored by TLC, water (5 mL) was carefully added. The reaction mixture was extracted with ethyl acetate (4 × 20 mL). The organic layer was dried over Na_2_SO_4_, filtered, and concentrated under reduced pressure to obtain the crude product. The crude product was purified with flash column chromatography (petroleum ether/ethyl acetate: gradient 0‐8%) to obtain the product as a colorless liquid (777 mg, 3.51 mmol, 51%).

*R*_f_ = 0.57 (petroleum ether/ethyl acetate: 10:1, KMnO_4_ staining); ^1^H NMR (500 MHz, DMSO‐*d*_*6*_, δ [ppm]): 5.92–5.82 (m, 1H, –O–CH_2_–HC═CH_2_), 5.27–5.09 (m, 2H, –O–CH_2_–HC═CH_2_), 3.90 (dt, *J* = 5.3, 1.5 Hz, 2H, –O–CH_2_–HC═CH_2_), 3.52 (t, *J* = 6.7 Hz, 2H, Br–CH_2_–(CH_2_)_5_–O–), 3.36 (t, *J* = 6.5 Hz, 2H, Br–(CH_2_)_5_–CH_2_–O–), 1.83–1.75 (m, 2H, Br–CH_2_–CH_2_–(CH_2_)_4_–O–), 1.55–1.46 (m, 2H, Br–(CH_2_)_4_–CH_2_–CH_2_–O–), 1.43–1.28 (m, 4H, Br–(CH_2_)_2_–(CH_2_)_2_–(CH_2_)_2_–O–); ^13^C NMR (126 MHz, DMSO‐*d*_*6*_, δ [ppm]): 135.39 (–O–CH_2_–HC═CH_2_), 115.96 (‐O‐CH_2_‐HC = CH_2_), 70.67 (‐O‐CH_2_‐HC = CH_2_), 69.28 (Br‐(CH_2_)_5_‐CH_2_‐O‐), 35.03 (Br‐CH_2_‐(CH_2_)_5_‐O‐), 32.10, 28.94, 27.26, 24.75 (Br‐CH_2_‐(CH_2_)_4_‐CH_2_‐O‐); LRMS (APCI^+^): *m/z* 221 [M + H]^+^. The obtained analytical data are in good agreement with literature values [51].

### 4.2 Compound Preparation for Testing

The solid compounds were dissolved and diluted in pure DMSO to a concentration of 50 mM. Then, a 1/10 dilution was made by adding 180 µL Page's Amoeba Saline (PAS, ATCC medium no. 1323, ATCC, USA) to 20 µL of the previous dilution, resulting in a concentration of 5 mM. All following concentrations were prepared by adding a solution composed of 90% (v/v) PAS and 10% (v/v) DMSO accordingly.

### 4.3 Sequence Alignment, Homology Modeling, Molecular Docking, and MD Simulations

*Sequence alignment:* Pairwise sequence alignment of human lanosterol synthase (LAS, also referred to as hOSC (Uniprot: P48449)) and CAS from *Acanthamoeba castellanii* (UniProt: L8GD76) was performed using Clustal Omega [52] and annotated with Jalview [53], while structural alignment was performed with PyMOL (Schrödinger LLC, NY, USA).

*Homology modeling:* A BLAST P [54] search using the CAS found in *A. castellanii* (NCBI accession ID: XP_004332827.1) as a template was conducted, yielding high percentage identity scores of 50.56% and 50.42% for the co‐crystallized structures of human OSC with bound lanosterol (PDB [55]: pdb_00001w6k [30]) and Ro 48‐8071 (**3**, PDB: pdb_00001w6j [30]), respectively. This process was repeated with the Molecular Operating Environment (MOE) [56], Software using its integrated PDB‐search feature, identifying the same two potential templates. Intending to use the homology model for docking studies with potential inhibitors derived from Ro 48 8071 (**3**), the co‐crystal structure of human OSC with bound Ro 48‐8071 (**3**, PDB: pdb_00001w6j) was taken as a template to provide accurate spatial orientation of the corresponding residues in the active site. 3D model generation was performed following optimization of alignment via MOE, using its integrated homology builder at default configuration, only employing the additional induced fit around the bound inhibitor Ro 48‐8071 (**3**), yielding a model based on the sequence of XP_004332827.1 with corresponding RMSD values of 0.519 Å for the spatial pocket residue alignment and an RMSD of 0.831 Å for the entire structure encompassing all residues with regards to the template structure pdb_00001w6j. As the model lacks bound water found in the template structure, docking results were only considered in the form of poses for visualization and guidance during elucidation of the SAR.

*Molecular docking:* For docking studies, we used the aforementioned homology model of *A. castellanii* CAS. The structures of the ligands were prepared with LigPrep [57] (Schrödinger LLC, NY, USA), employing the OPLS4 force field [58]. The geometric center of the ligand was treated as the grid centroid for flexible docking, which was executed with Glide's [59] Standard Precision (SP) scoring function. Binding pose depictions were generated using PyMOL (Schrödinger LLC, NY, USA).

*MD:* Simulations were conducted using Desmond [60], also applying the OPLS4 force field. The docked complex was solvated with TIP3P water molecules, and the system's charge was neutralized using counterions within an orthorhombic periodic box that featured 10 Å side barriers. Following an equilibration protocol, production MD simulations were run for 100 ns in an NPT ensemble at 300 K, which was regulated by a Nosé–Hoover thermostat and a Martyna–Tobias–Klein barostat. Atomic coordinates were recorded every 400 ps, resulting in a total of 250 frames.

### 4.4 Acanthamoeba Culture

#### 4.4.1 Trophozoites

Trophozoites were cultivated as described previously [14]. Briefly, trophozoites belonging to the pathogenic *A. hatchetti* strain (2 HH) were obtained from the research group of Prof. Julia Walochnik (Vienna, Austria) and cultured in a medium composed of (20.0 g proteose peptone (20.0 g/L), yeast extract (20.0 g/L), glucose (18.0 g/L), CaCl_2_ (8.0 mL of 0.05 M), MgSO_4_·7H_2_O (10 mL of 0.4 M), Na_2_HPO_4_·7H_2_O (10 mL of 0.25 M), KH_2_PO_4_ (10 mL of 0.25 M), sodium citrate (1.0 g/L), and Fe(NH_4_)_2_(SO_4_)_2_ (10 mL of 0.005 M) (all from Carl Roth, Karlsruhe, Germany) in double‐distilled water, also known as peptose yeast‐extract glucose medium (PYG). Cells were maintained in an incubator at 30.0°C and passaged weekly.

#### 4.4.2 Cysts

Trophozoites obtained as described above were cultivated as described previously [14]. After centrifugation, the culture medium was removed, and the cell pellet was resuspended in Neff's Encystation Medium (NEM) according to Kilvington et al. [61]. NEM consisted of KCl (0.1 M), NaHCO_3_ (0.001 M), MgSO_4_·7H_2_O (0.008 M), Tris (0.02 M), CaCl_2_ (0.0004 M), and phenol red–sodium solution (25 µL of a 1.5% w/w solution in H_2_O), prepared in double‐distilled water (Carl Roth, Karlsruhe, Germany) and adjusted to pH 8.9 ± 0.1. Cultures were incubated at room temperature for 10–14 days until near‐complete encystation was confirmed. The resulting cysts were subsequently washed and stored at 4°C–8°C in Page's Amoeba Saline (PAS composed of Na_2_HPO_4_ (0.142 g/L), KH_2_PO_4_ (0.136 g/L), MgSO_4_·7H_2_O (0.004 g/L), CaCl_2_·2H_2_O (0.004 g/L), and NaCl (0.120 g/L) in distilled water (Carl Roth).

### 4.5 Amoebicidal Assays

#### 4.5.1 Determination of the Minimal Trophicidal Concentration (MTC)

Upon reaching confluence, trophozoites were detached on ice, followed by removal of PYG via centrifugation and its replacement by PAS. The resulting suspension was adjusted to a cell density of 5 × 10^5^ cells/mL employing a hemocytometer of the Neubauer type.

A volume of 50 µL of the cell suspension were added per well of a 96‐well plate and incubated at 30°C for 30 min to allow attachment. Subsequently, 50 µL of the test compound diluted in PAS containing 10% (v/v) DMSO (controls: 90% PAS, 10% DMSO) were added, yielding final concentrations of 4.88 to 156 µM. After 2 h at 30°C, the medium was gently replaced with PYG. Plates were then incubated for 14 days and monitored on Days 2, 4, 7, and 14 for growth, trophozoite morphology, or disintegration. The MTC was defined as the lowest concentration showing no growth, intact morphology, or complete cell disintegration.

#### 4.5.2 Determination of the Minimal Cysticidal Concentration (MCC)

After adjusting the obtained cyst suspension (see above) to a concentration 5 × 10^5^ cells/mL in PAS, the same procedure as for the determination of the MTC was applied. The MCC was defined as the concentration at which no proliferation or excystation could be observed microscopically.

### 4.6 Cell Culture

#### 4.6.1 HCE‐T

Human corneal epithelial cells (HCE‐T, SV‐40 immortalized, RCB Cat# RCB2280, RRID:CVCL_1272) [62] were purchased from RIKEN Cell Bank (Tsukuba, Japan). Keratinocyte growth medium KGM (Lonza Bioscience, Basel, Switzerland) spiked with 700 µL of CaCl_2_ (27.75 mg/mL, Acros Organics, Geel, Belgium) was used for cultivation in an incubator at 37°C, 5% CO_2_, and 100% RH. The cells were fed three times per week and passaged weekly.

### 4.7 Viability Testing (MTT)

The cytotoxicity of the tested compounds on HCE‐T cells was assessed via MTT assay, as described previously [14], with the incorporation of DMSO to enhance solubility of the investigated compounds. Briefly, HCE‐T cells were seeded at 2.5 × 10^4^ cells/well in 96‐well plates containing KGM medium and cultured for 48 h at 37°C, 5% CO_2_, and 100% RH until confluence. The medium was replaced with 50 µL of Krebs–Ringer buffer (KRB; CaCl_2_·2H_2_O 0.13 g, d‐glucose monohydrate 0.55 g, HEPES PUFFERAN 1.79 g, NaCl 3.40 g, NaHCO_3_ 1.05 g, NaH_2_PO_4_·H_2_O 0.07 g (all from Carl Roth), KCl 0.20 g, MgSO_4_·7H_2_O 0.10 g (both from Acros Organics) in 500 mL double‐distilled water, pH adjusted to 7.40 with 3 M NaOH (Carl Roth)) followed by 50 µL of compounds in KRB containing 10% (v/v) DMSO (positive controls: final concentration of 5% (v/v) DMSO) at final concentrations of 9.77 to 313 µM. For negative controls, 50 µL of 1% (v/v) Triton X‐100 (Carl Roth) in KRB containing 10% DMSO (v/v) were added instead. Plates were incubated for 10–120 min at 37°C, 5% CO_2_. After exposure, wells were washed once with KRB and incubated with 100 µL MTT solution (0.5 g MTT (Merck, Germany) in 10 mL double‐distilled water, further diluted 1:100 in KGM) for 2 h. The medium was then removed and replaced with 100 µL lysis solution (sodium dodecyl sulfate 1.365 g, isopropanol 452.7 g, 37% HCl 1.7%, water 44.1 g). Plates were shielded from light and shaken at 150 rpm for 2 h before measuring absorbance at 570 nm using a Tecan Infinite 200 Pro.

### 4.8 Statistical Analysis

A one‐way ANOVA test using OriginPro 2019b (Northampton, USA) was used for the statistical analysis. The tested level of significance was *p* < 0.05. The Post‐Hoc test according to Bonferroni was employed. The results are illustrated in Supporting Information S1: Tables S1‐5.

## Funding

The authors have nothing to report.

## Conflicts of Interest

The authors declare no conflicts of interest.

## Supporting information


Supporting File

## Data Availability

The data that support the findings of this study are available from the corresponding author upon reasonable request.
