# Supplementary material for: Benzophenone Derivatives as a New Class of Anti‐Infectives Against Acanthamoeba spp
Source: Arch Pharm (Weinheim). 2026 Aug 20;359(8):e70321. doi: 10.1002/ardp.70321 (PMC13492222; doi:10.1002/ardp.70321)
Supplement: Supplementary file 1 [file ARDP-359-e70321-s001.pdf]

Supporting Information
©Wiley-VCH 2025
69451 Weinheim, Germany

Benzophenone derivatives as a new class of anti-infectives against *Acanthamoeba spp.*

Tomas Rimkus[1], Hannes Geisler[2], Taha B. El-Jourani[2], Aurélien F. A. Moumbock[2,3,4], Matthias Schiedel[2,4,5], Stephan Reichl*[1,5]

1 Institute of Pharmaceutical Technology and Biopharmaceutics, Technische Universität Braunschweig, Braunschweig, Germany
2 Institute of Medicinal and Pharmaceutical Chemistry, Technische Universität Braunschweig, Braunschweig, Germany
3 Institute of Pharmaceutical Sciences, Albert-Ludwigs-Universität Freiburg, Freiburg, Germany
4 Universität Münster, Institut für Pharmazeutische und Medizinische Chemie, Corrensstraße 48, D-48149, Münster, Germany
5 Center of Pharmaceutical Engineering, Technische Universität Braunschweig, Braunschweig, Germany
*Correspondence:
Prof. Dr. Stephan Reichl, Institute of Pharmaceutical Technology and Biopharmaceutics, Mendelssohnstraße 1, 38106 Braunschweig
Email: s.reichl@tu-braunschweig.de

Abstract: The rare but sight-threatening ocular disease *Acanthamoeba* keratitis represents a therapeutic challenge due to its causative agents – *Acanthamoeba spp.* – being able to penetrate the corneal epithelium and persist in the corneal stroma as dormant and highly resistant cysts. To combat the scarcity and limitations of current treatment options, we developed a new class of anti-infective agents against *Acanthamoeba spp*. based on Ro 48-8071 (**3**), a known inhibitor of the human oxidosqualene cyclase (hOSC). The compound design was guided by docking studies using a homology model of the cycloartenol synthase (CAS), which is the homolog of hOSC found in *Acanthamoeba spp.* and essential for the parasite's steroid biosynthesis. Among the synthesized Ro 48-8071 analogues, MSHG19 (**4f**) evoked the most potent activity against *Acanthamoeba hatchetti* leading to total eradication of trophozoites and cysts at single-digit micromolar concentrations, whereas having no effects within this concentration range on the viability of human corneal epithelial cells. With our initial set of synthesized compounds, we established a first structure-activity relationship model, which nicely aligns with the predicted binding mode of **4f** to CAS. Overall, the presented findings highlight the potential of benzophenones as a new class of anti-infectives for the treatment of *Acanthamoeba* keratitis.

Table of Contents

Page	Contents
S3	Supplementary Figures
S6	Supplementary Microscopic Images
S13	Statistical Evaluation
S18	Supplementary NMR Spectra
S91	Supplementary HPLC Chromatograms
S99	Supplementary Compound Table (InChI format)
S100	Supplementary References

Supplementary Figures

Figure S1. Sequence alignment of human lanosterol synthase (LAS, also referred to as hOSC (Uniprot: P48449)) and CAS from *Acanthamoeba castellanii* (UniProt: L8GD76), revealed a sequence identity of 50%. Identical amino acids are highlighted in color. Identical amino acids outside the active site are highlighted in blue. Identical amino acids interacting with Ro 48-8071 (**3**)[1] are marked with a yellow frame and highlighted according to the Clustal Omega color coding. Non-identical residues within the active sites are marked with a red frame. Sequence alignment was performed with Clustal Omega[2,3].

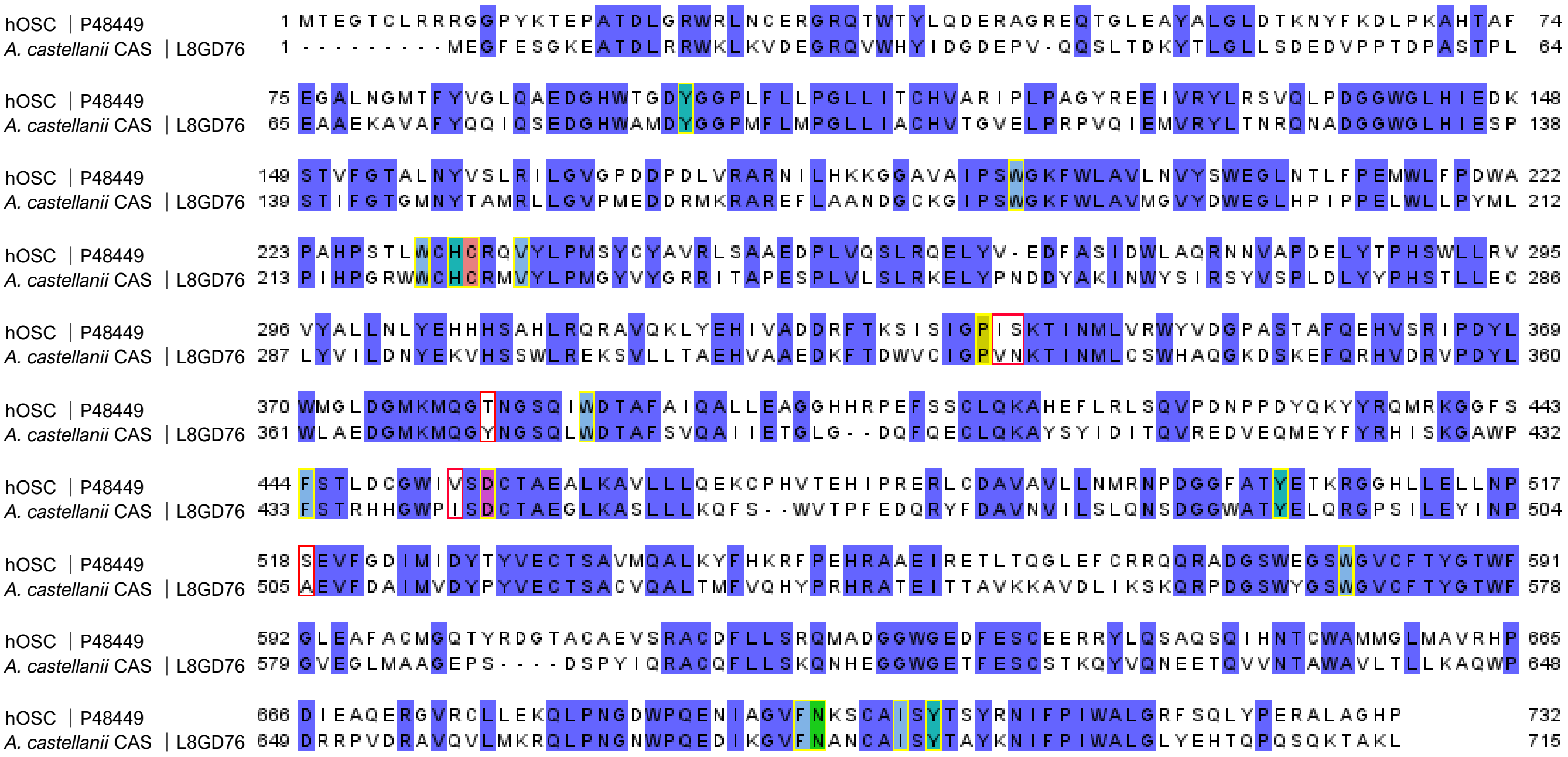

Figure S2. Molecular dynamics (MD) simulation investigating the stability of the complex of **4f** bound to a homology model of CAS from *A. castellanii*. MD simulations that were performed over a time period of 100 ns show a high stability of the protein-ligand complex. The Root Mean Square Deviation (RMSD) for the protein is shown in turquoise and the RMSD for the ligand is shown in magenta.

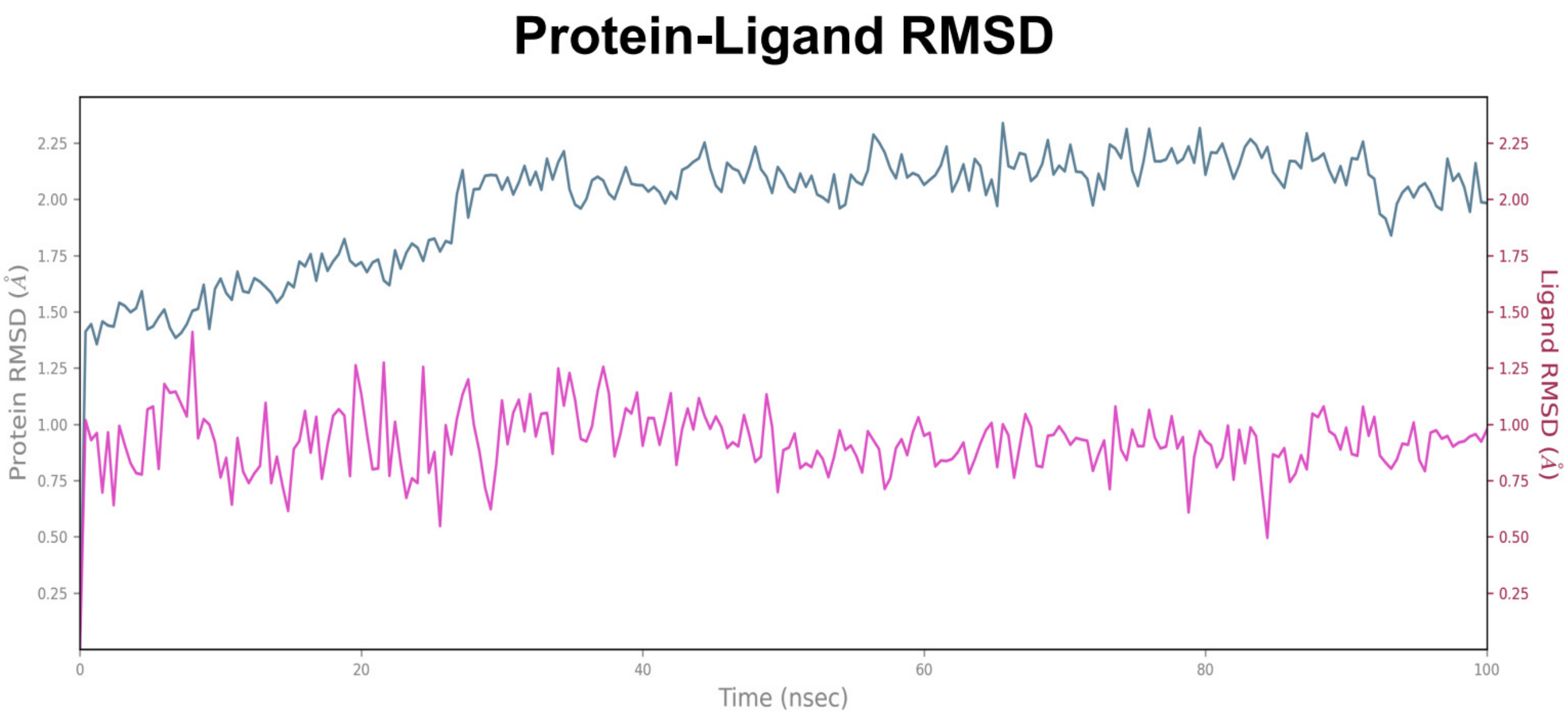

Figure S3. Molecular dynamics (MD) simulation investigating the protein-ligand contacts within the complex of **4f** bound to a homology model of CAS from *A. castellanii*. A) Protein-ligand contacts analyzed by the interaction fractions of each amino acid engaging with **4f**. B) Schematic illustration of detailed protein-ligand contacts with the individual subunits of **4f**. Interactions that occur more than 0.0% of the simulation time in the selected trajectory (0.00 through 100.00 ns), are shown. Note: The Schrödinger software used does not support visualization of π–π interactions between aromatic amino acid residues and unconjugated double or triple bonds of the ligand.

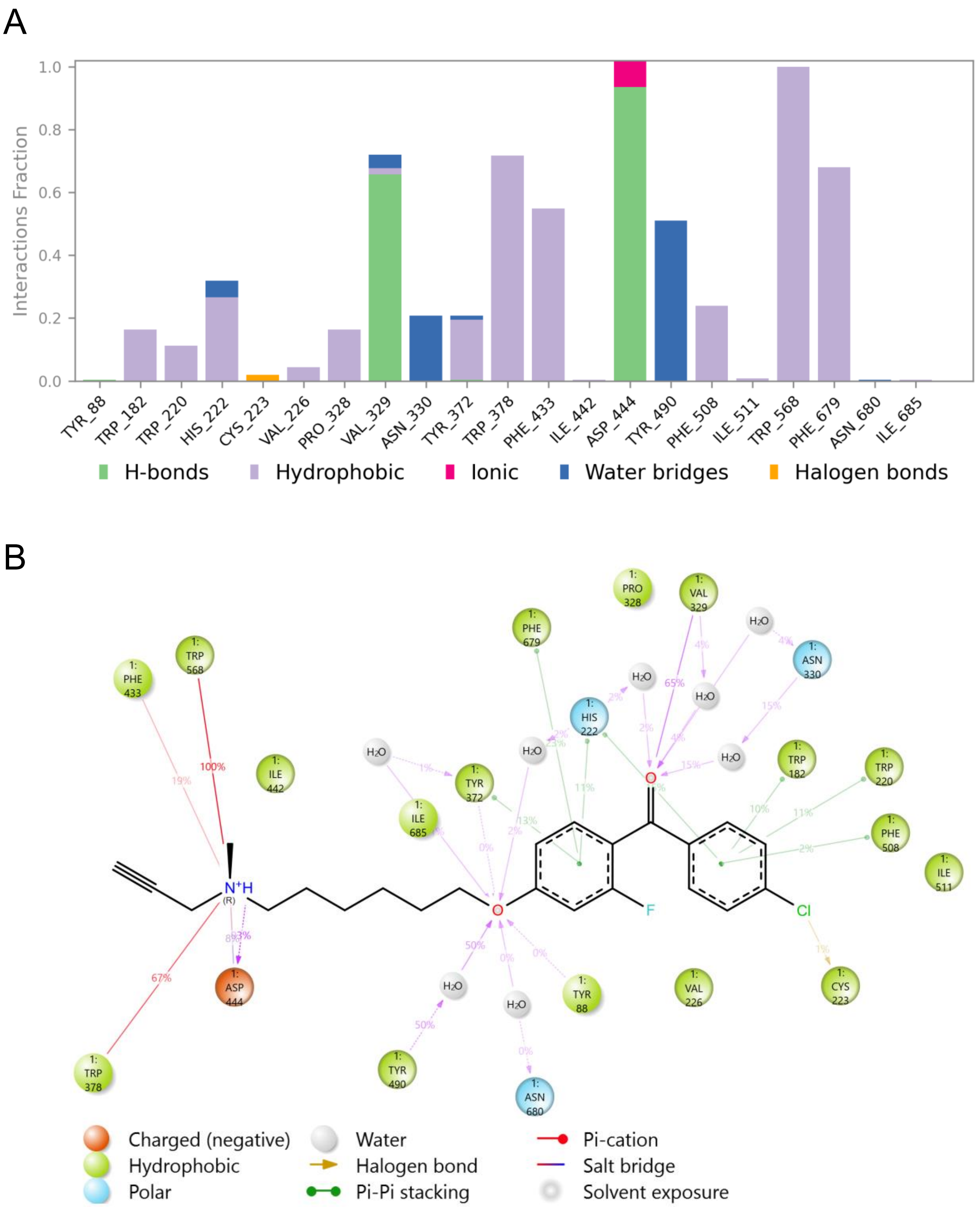

Supplementary Microscopic Images

Part A: Enlarged representation of the images already included in the manuscript

A: PAS (negative control), 7 d, scale bar = 100 µm

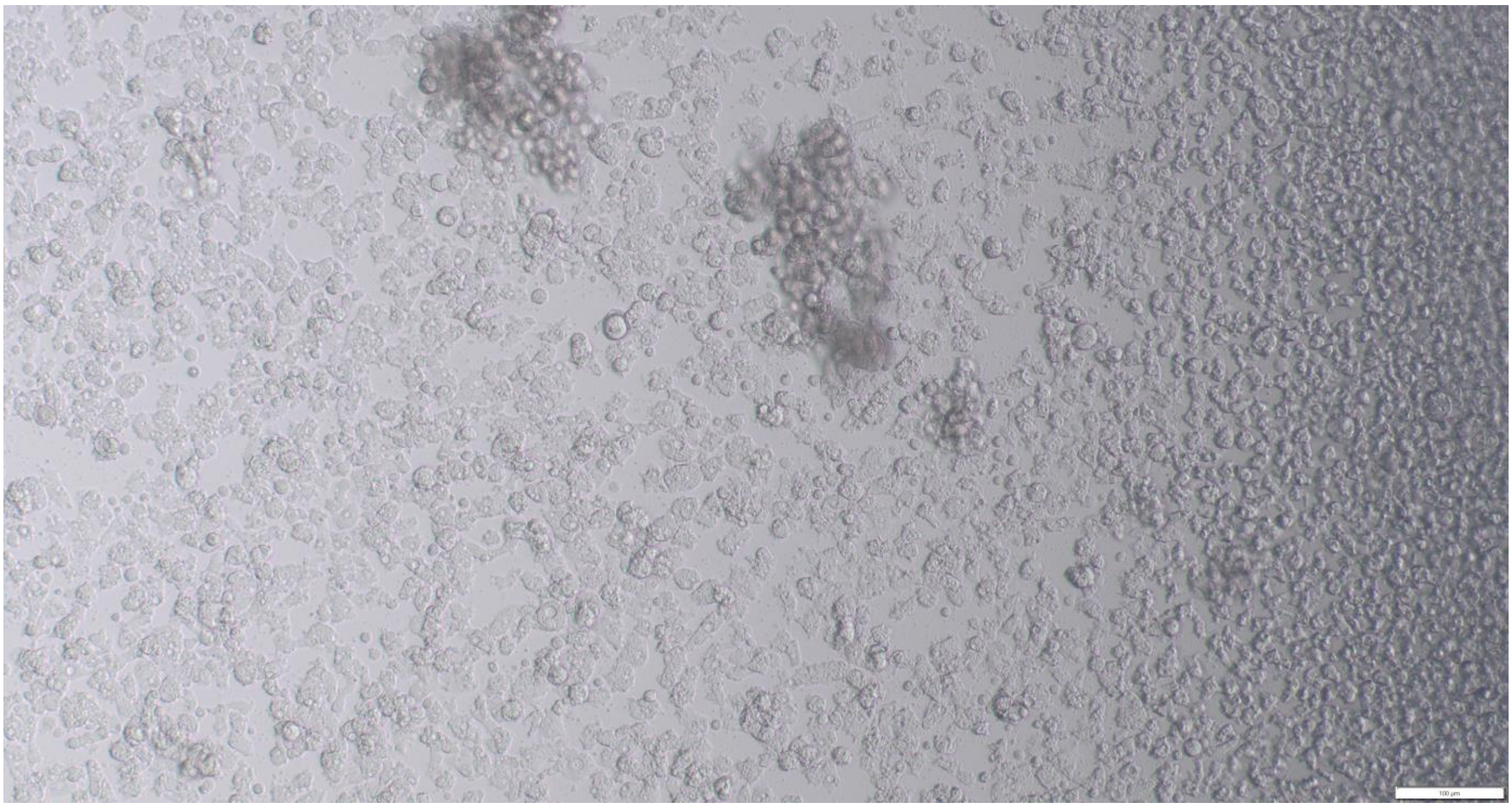

B: 3, 78.1 µM, 14 d, scale bar = 100 µm

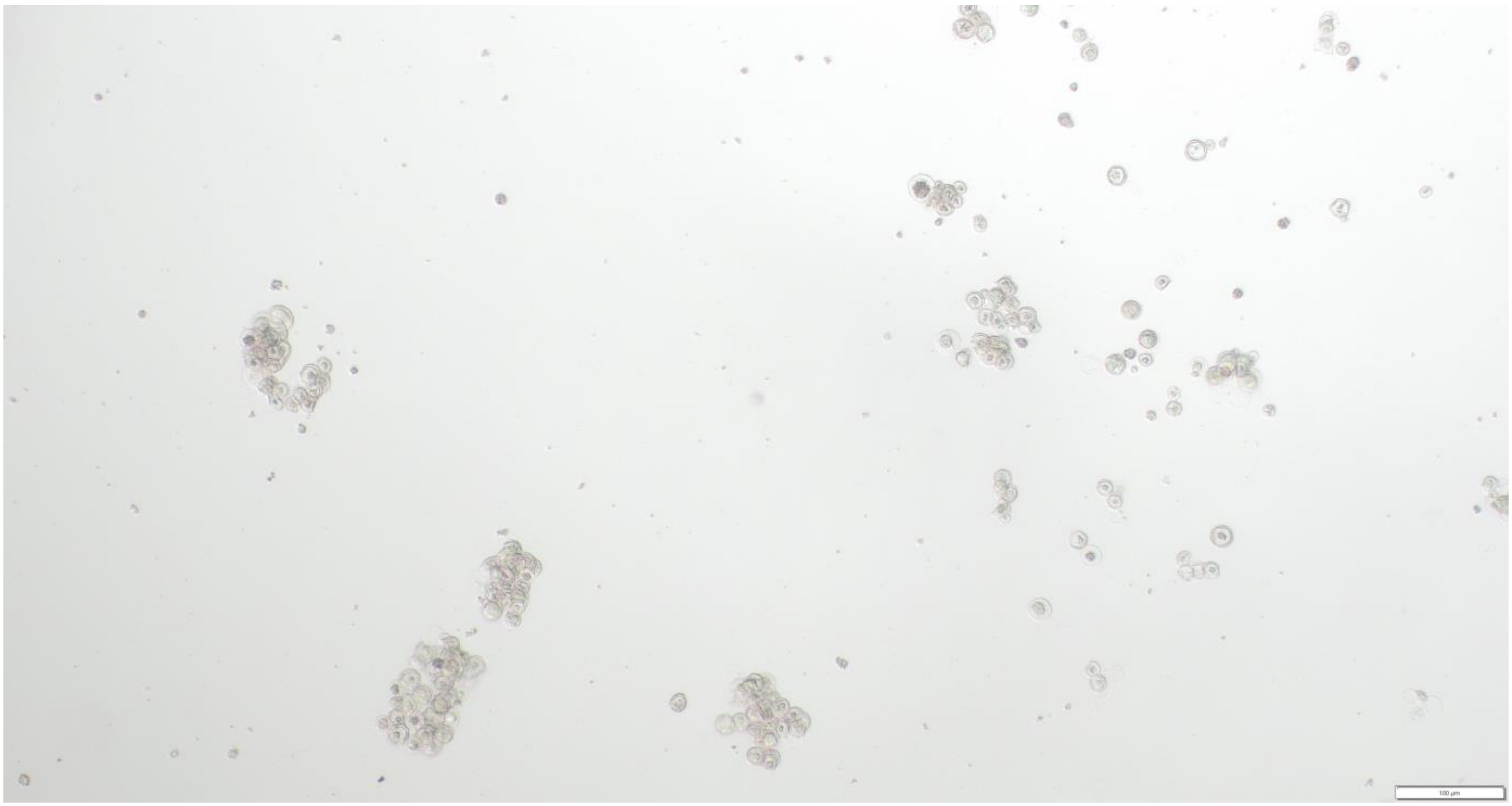

C: 3, 39.1 µM, 7 d, scale bar = 100 µm

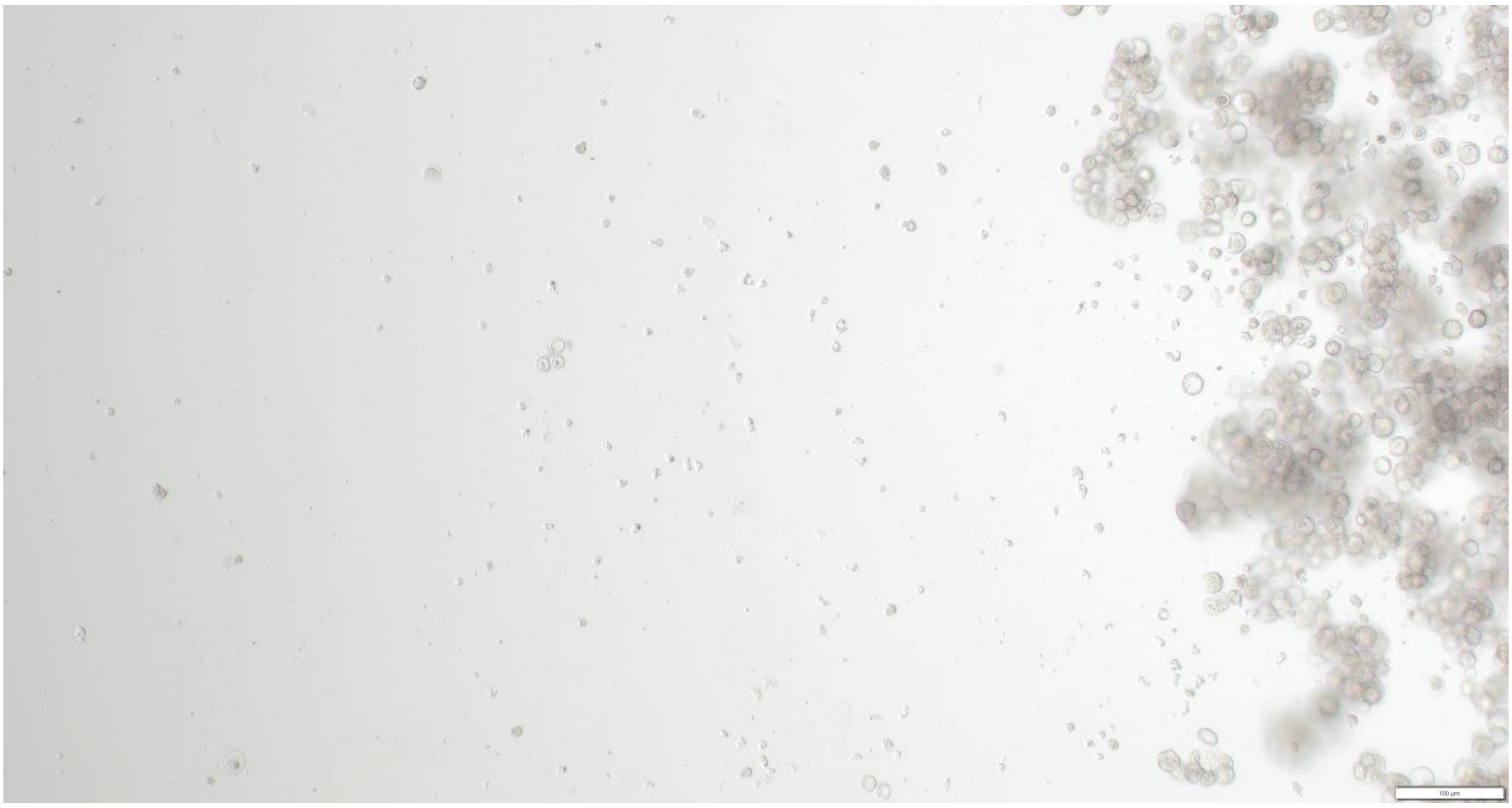

D: PAS (negative control), 14 d, scale bar = 200 µm

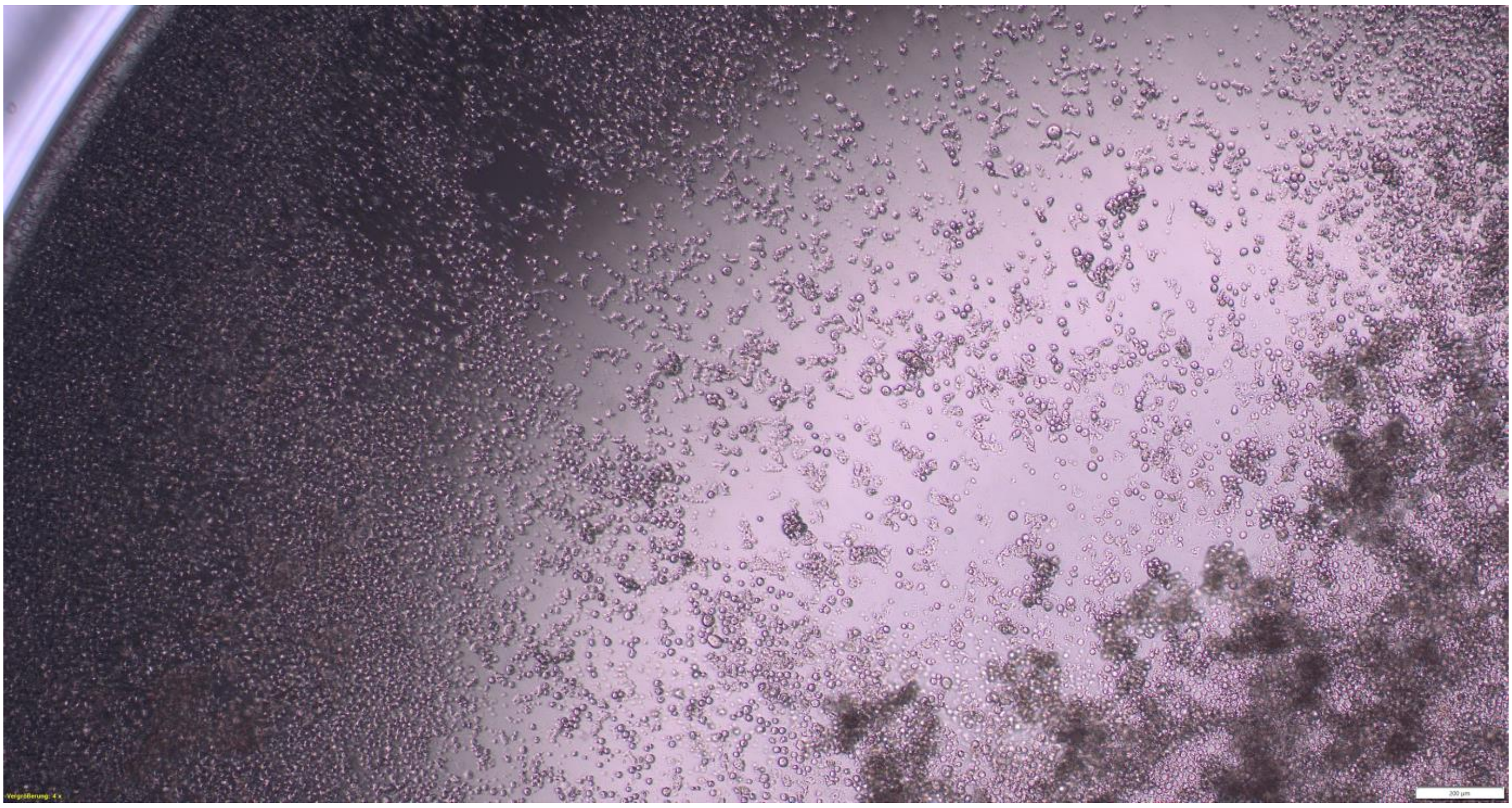

E: 3, 39.1 µM, 14 d, scale bar = 100 µm

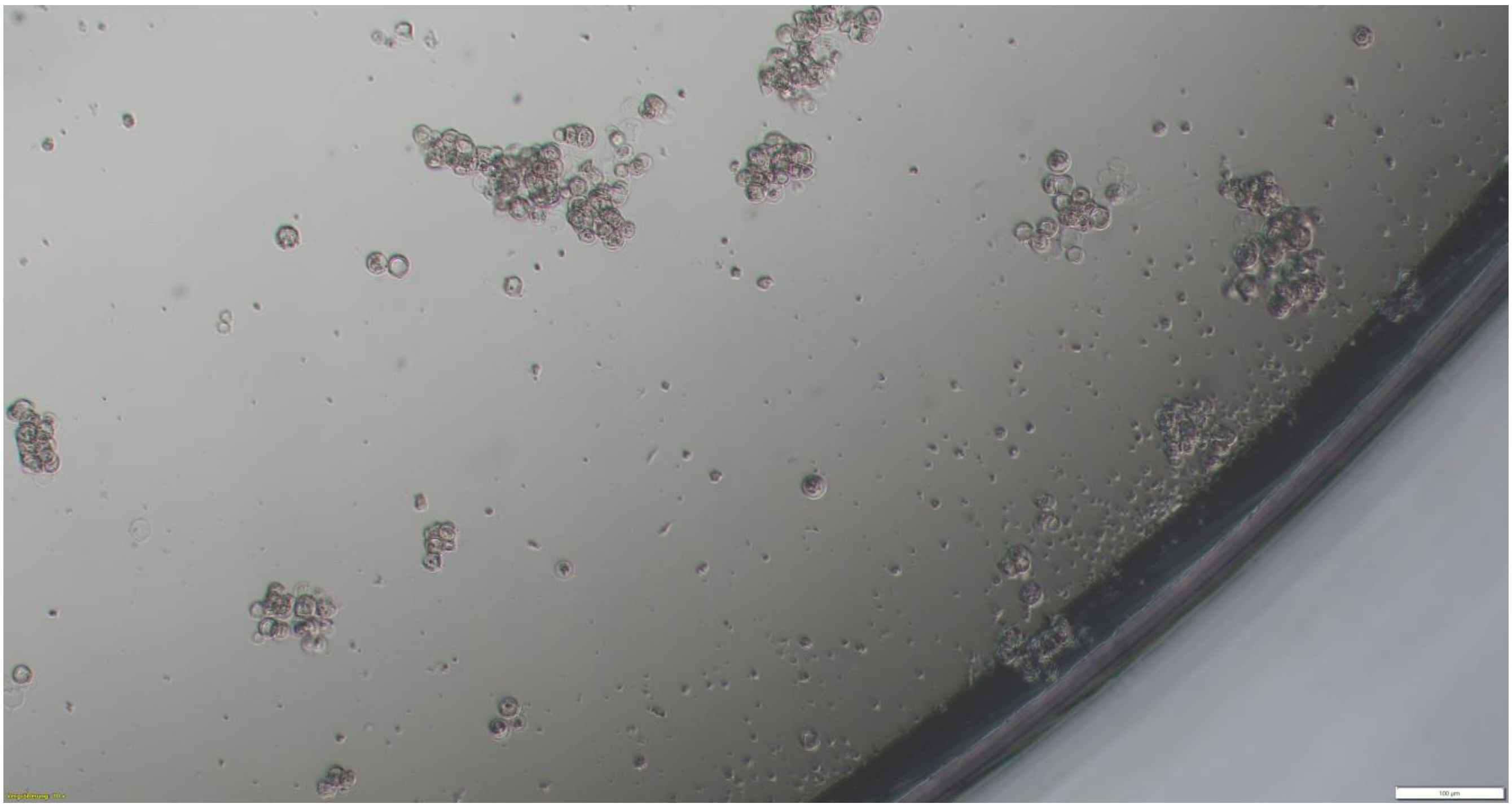

F: 3, 19.5 µM, 14 d, scale bar = 100 µm

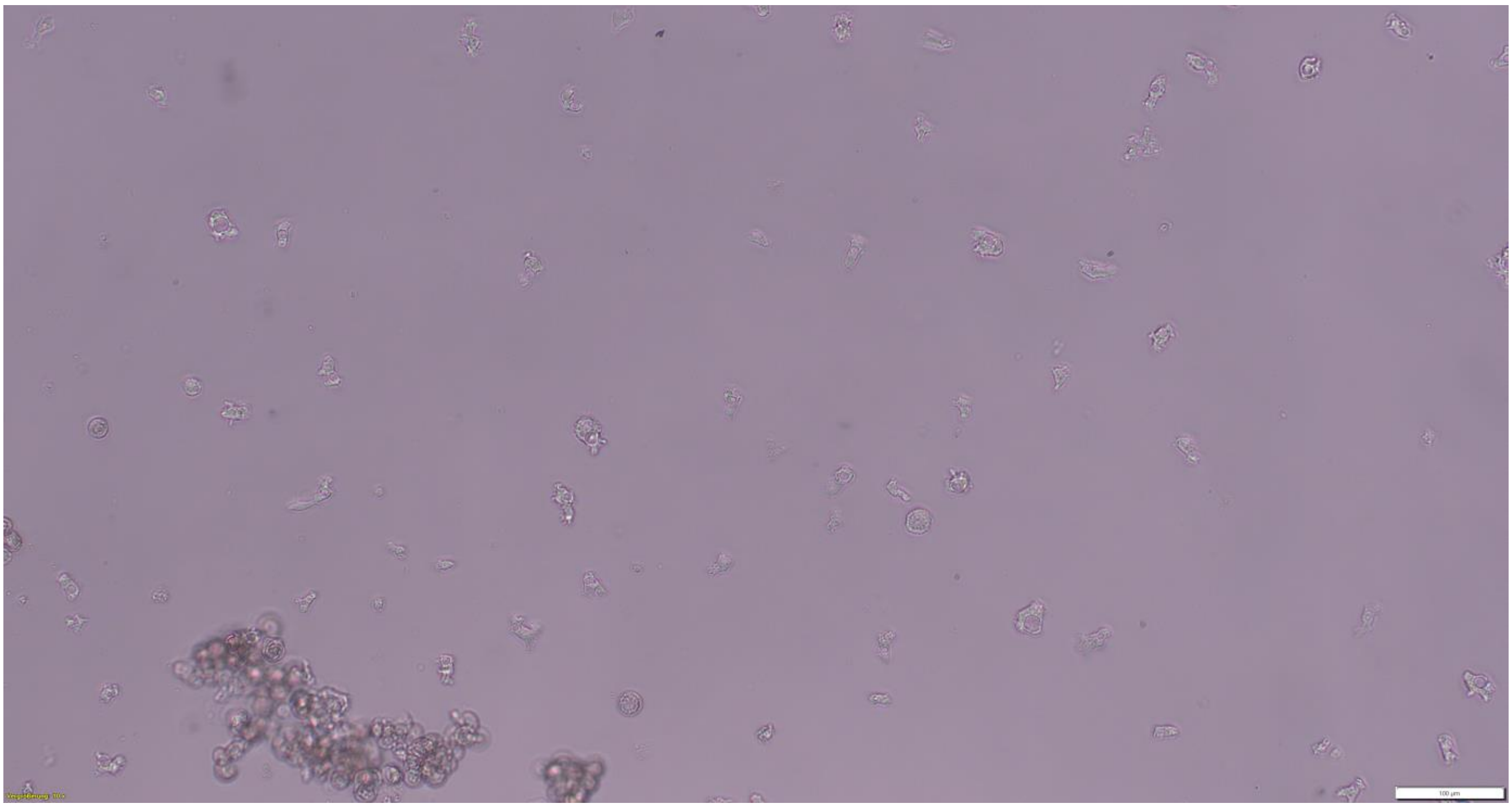

G: 3, 4.88 μM, 14 d, scale bar = 200 μm

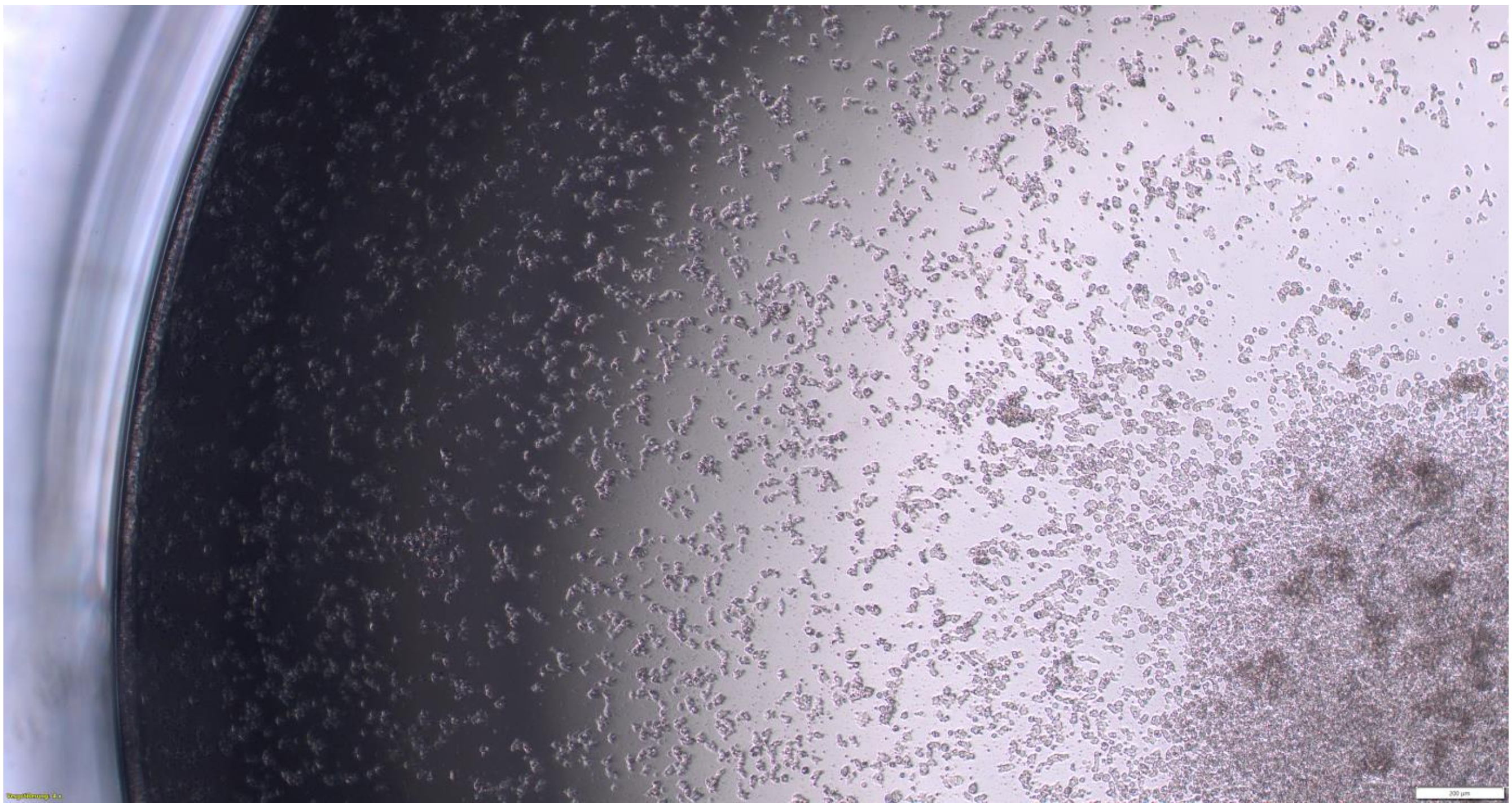

H: 4f, 39.1 μM, 14 d, scale bar = 100 μm

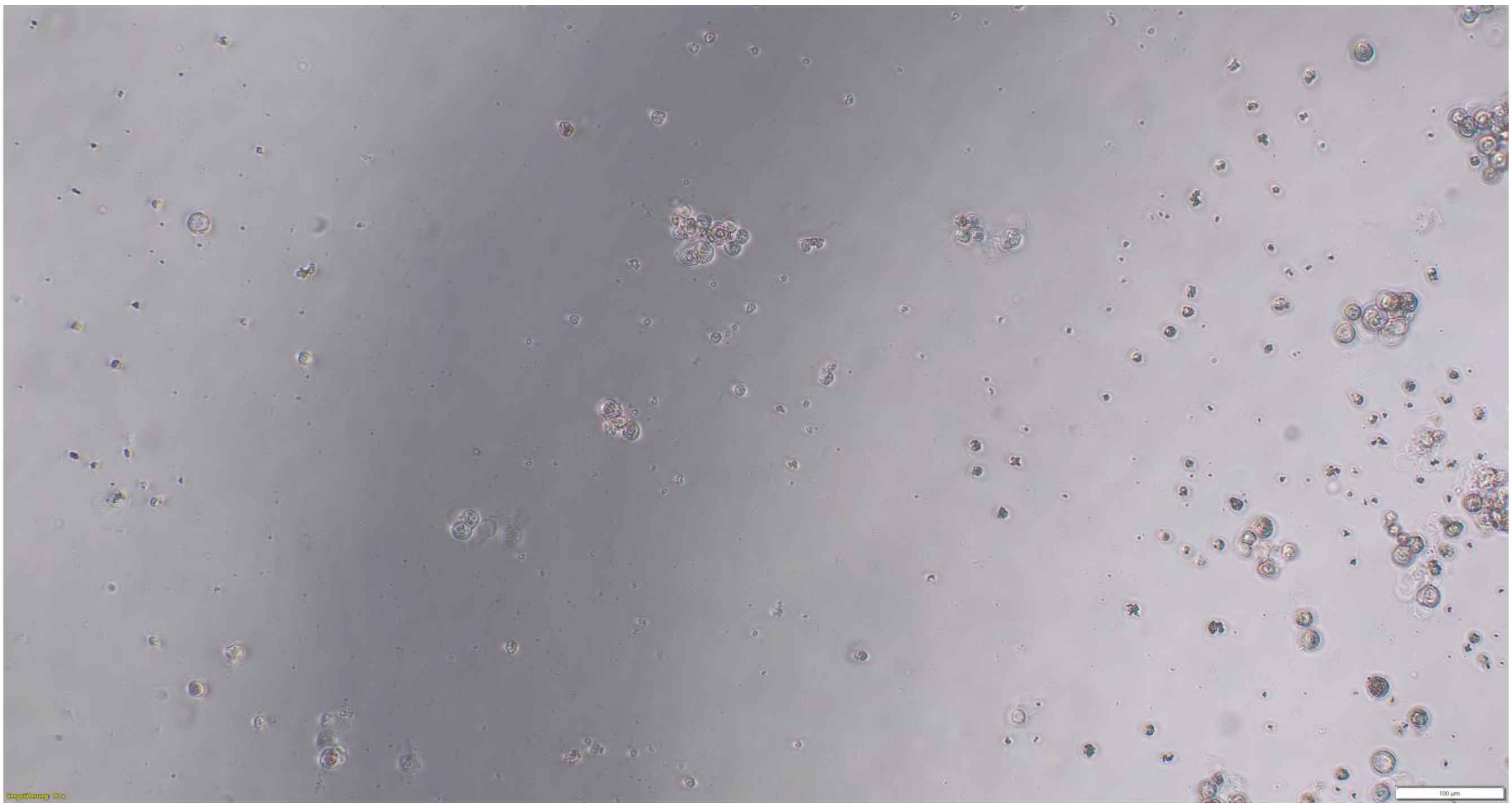

I: 4f, 4.88 µM, 14 d, scale bar = 20 µm

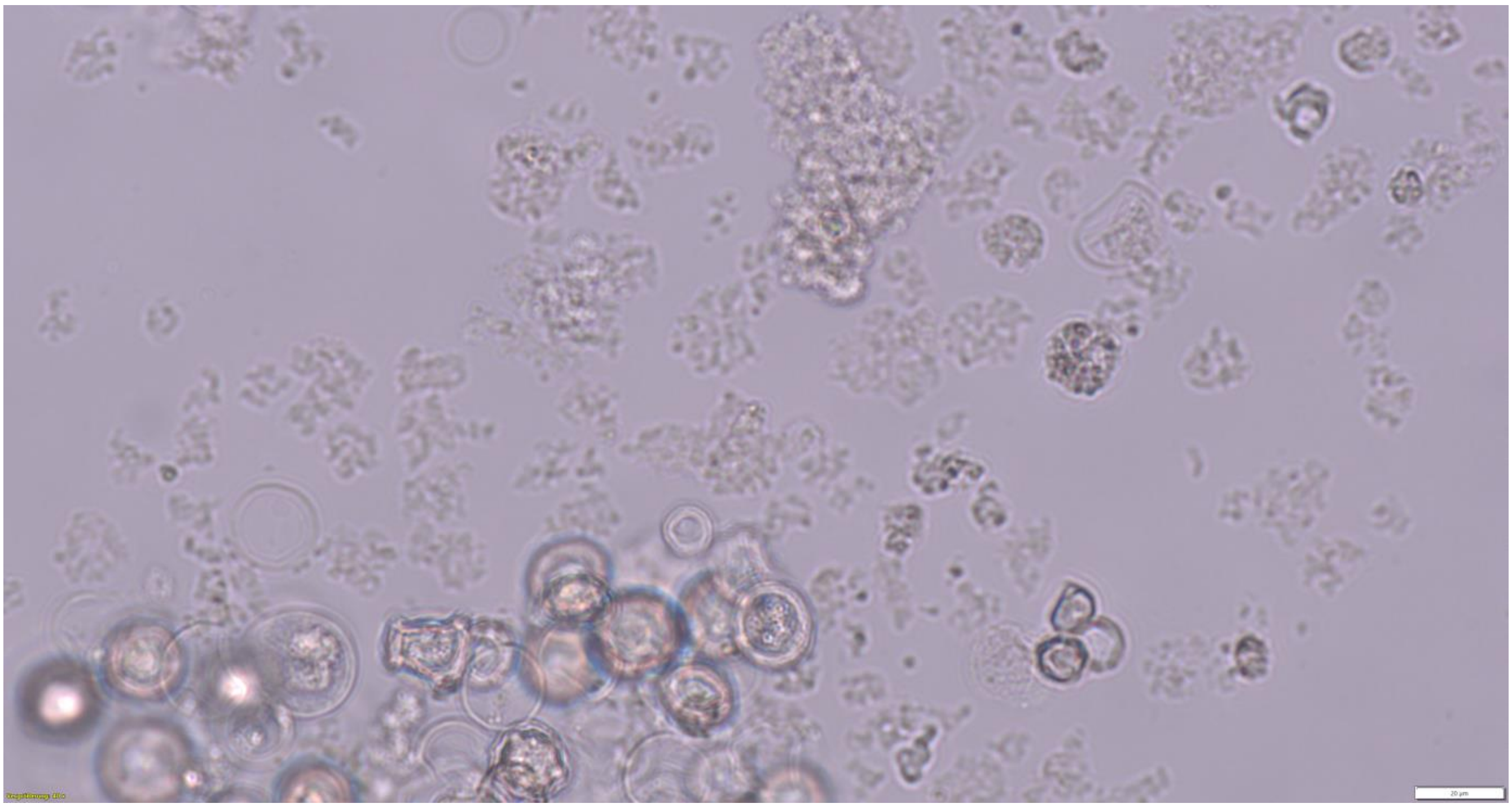

J: 4f, 9.88 µM, 7 d, scale bar = 50 µm

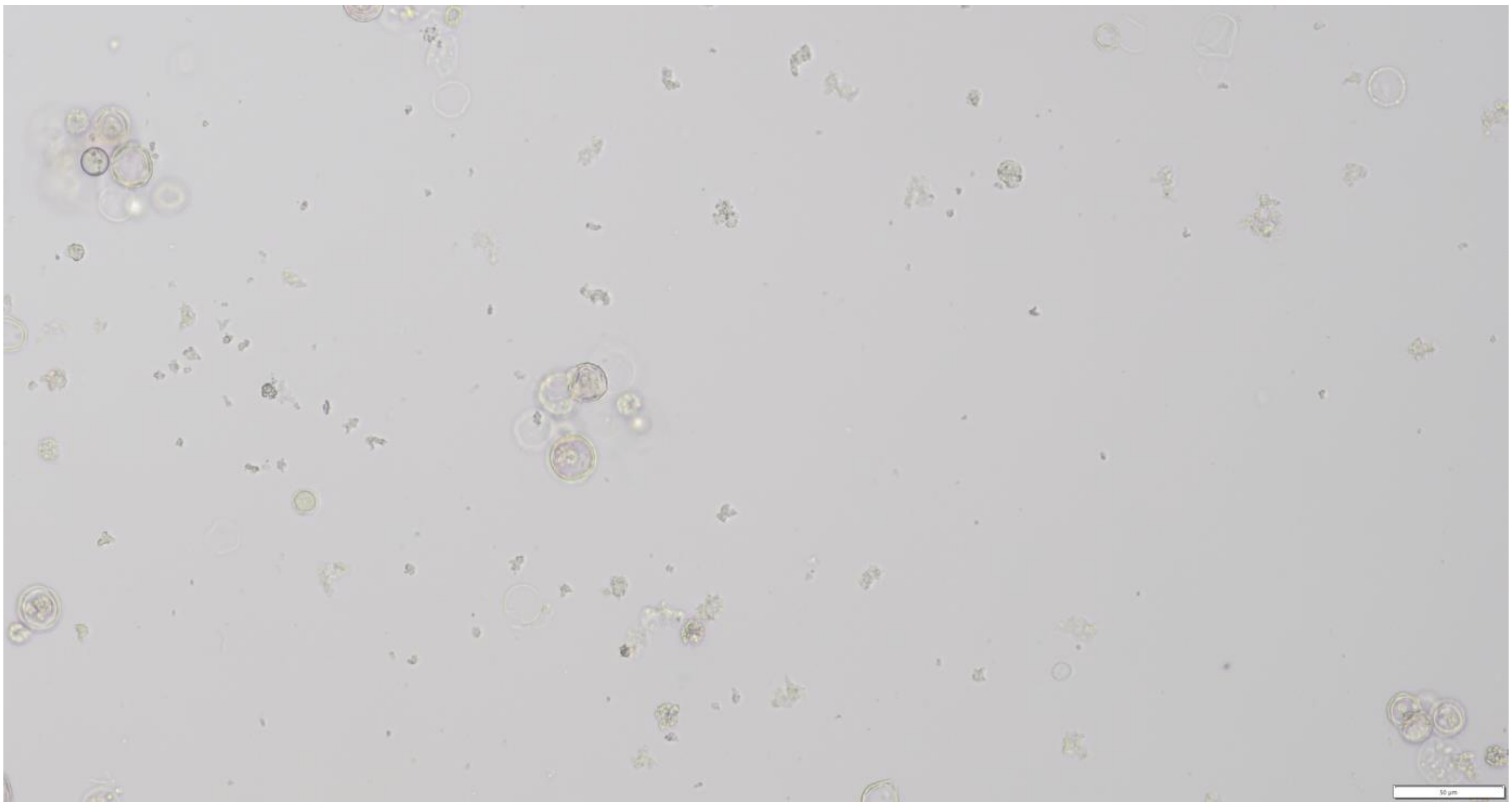

Part B: Additional Microscopic Images

A: 3, 156 µM, 7 d, scale bar = 100 µm

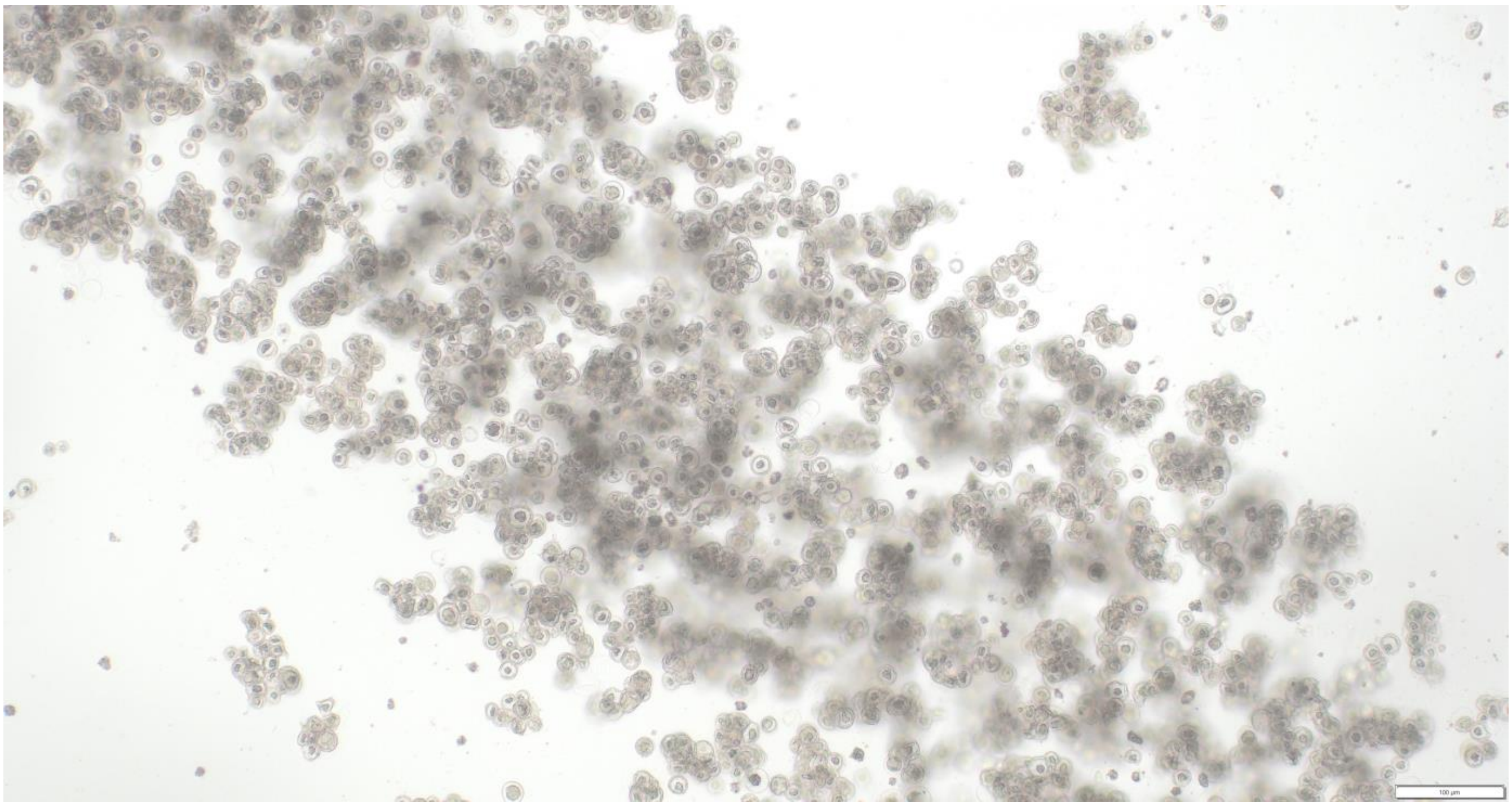

B: 3, 39.1 µM, 7 d, scale bar = 50 µm

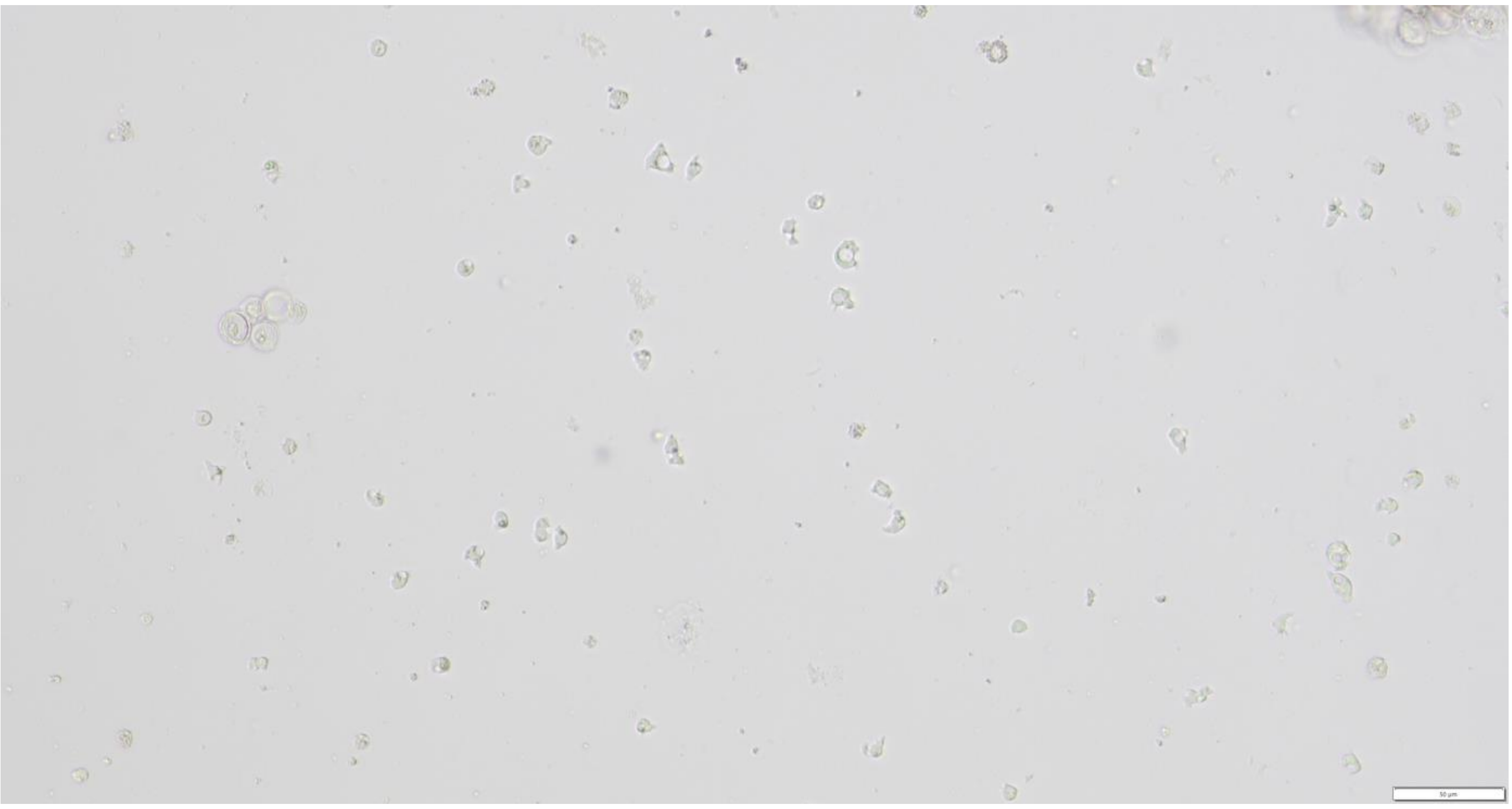

C: 4f, 39.1 µM, 14 d, scale bar = 100 µm

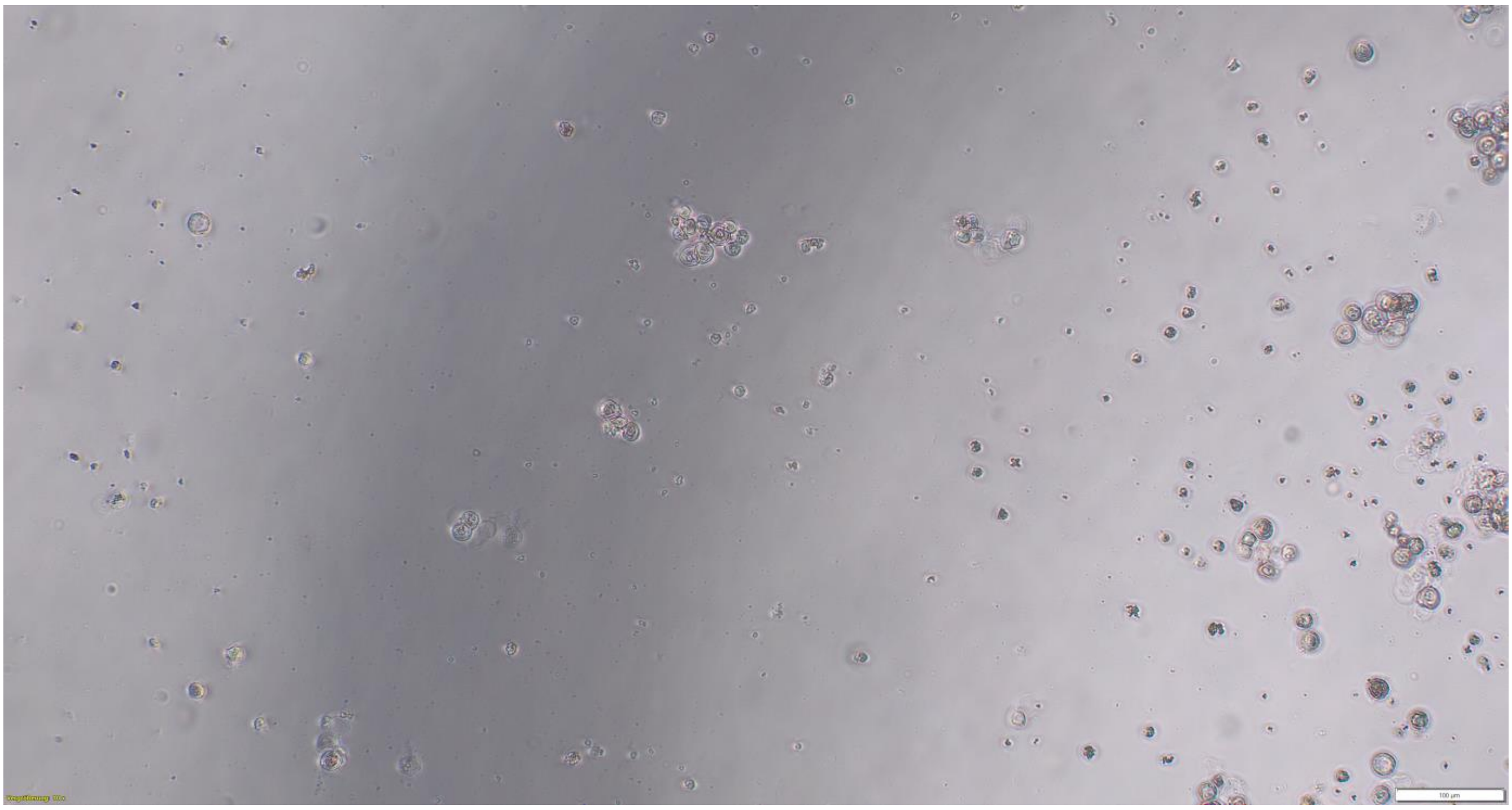

D: 4f, 4.88 µM, 14 d, scale bar = 50 µm

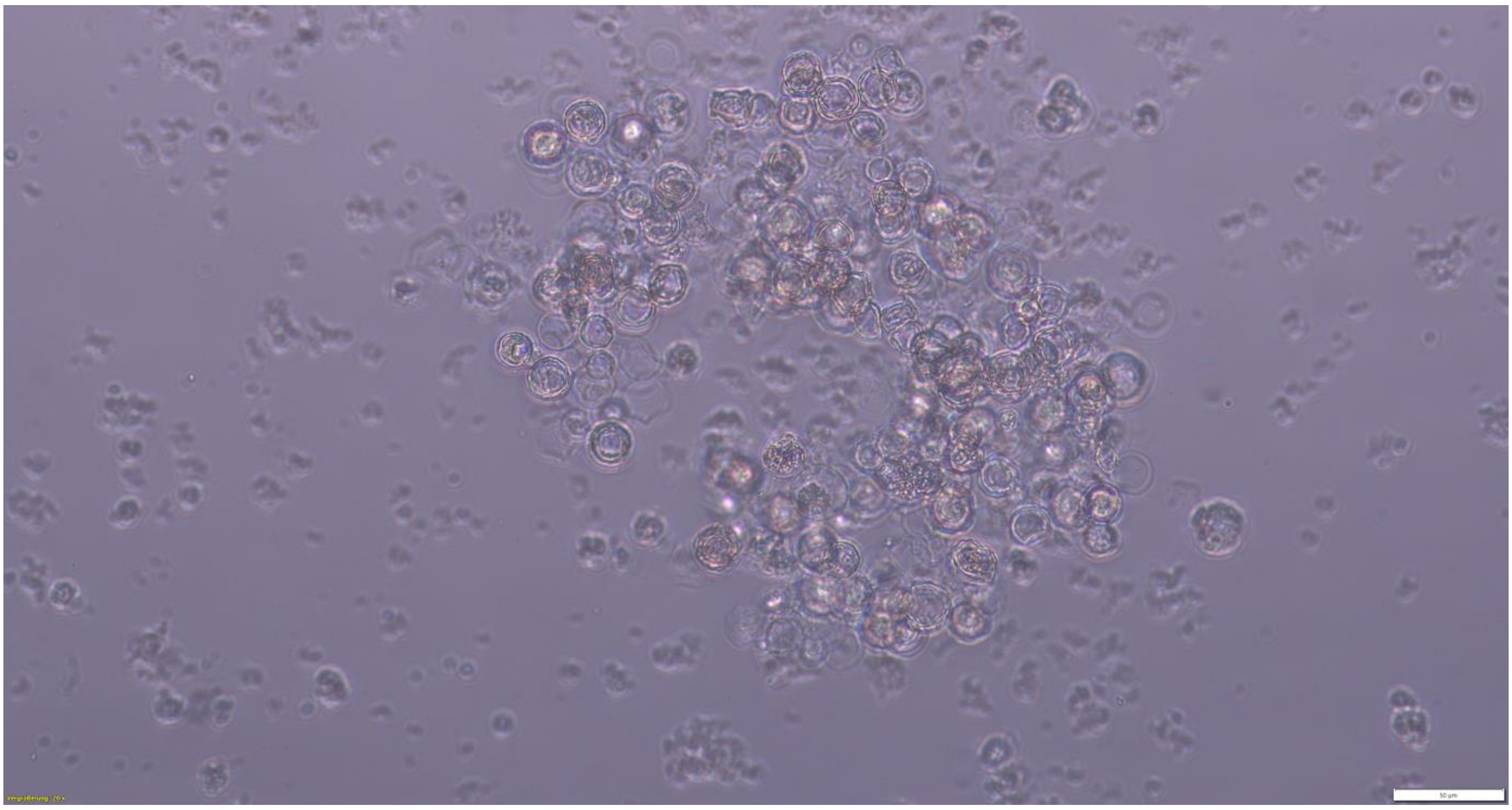

Statistical Evaluation

A one-way ANOVA test using OriginPro 2019b (Northampton, USA) was used for the statistical analysis. The tested level of significance was $P < 0.05$. The Post-Hoc test according to Bonferroni was employed.

Table S1 Statistical significances for the MTT-test after 10 min of incubation calculated with the post-hoc test according to Bonferroni, $\alpha = 0.05$; [1] – **3** (Ro 48-8071), [2] – **4b**, [3] – **4a**, [4] – **4c**, [5] – **4f**, 1 = significant, 0 = not significant

MTT 10 min		
**Group**	**P**	**Significance**
[2] 156.3 [1] 156.3	1	0
[2] 78.13 [1] 78.13	1	0
[2] 39.06 [1] 39.06	1	0
[2] 19.53 [1] 19.53	1	0
[2] 9.766 [1] 9.766	1	0
[2] 4.883 [1] 4.883	1	0
[3] 156.3 [1] 156.3	9.17E-08	1
[3] 156.3 [2] 156.3	1.45E-04	1
[3] 78.13 [1] 78.13	0.02548	1
[3] 78.13 [2] 78.13	1	0
[3] 39.06 [1] 39.06	1	0
[3] 39.06 [2] 39.06	1	0
[3] 19.53 [1] 19.53	1	0
[3] 19.53 [2] 19.53	1	0
[3] 9.766 [1] 9.766	1	0
[3] 9.766 [2] 9.766	1	0
[3] 4.883 [1] 4.883	1	0
[3] 4.883 [2] 4.883	1	0
[4] 156.3 [1] 156.3	1	0
[4] 156.3 [2] 156.3	1	0
[4] 156.3 [3] 156.3	0.06314	1
[4] 78.13 [1] 78.13	1	0
[4] 78.13 [2] 78.13	1	0
[4] 78.13 [3] 78.13	1	0
[4] 39.06 [1] 39.06	1	0
[4] 39.06 [2] 39.06	1	0
[4] 39.06 [3] 39.06	1	0
[4] 19.53 [1] 19.53	1	0
[4] 19.53 [2] 19.53	1	0
[4] 19.53 [3] 19.53	1	0
[4] 9.766 [1] 9.766	1	0
[4] 9.766 [2] 9.766	1	0
[4] 9.766 [3] 9.766	1	0
[4] 4.883 [1] 4.883	1	0
[4] 4.883 [2] 4.883	1	0
[4] 4.883 [3] 4.883	0.00907	1
[5] 156.3 [1] 156.3	1	0
[5] 156.3 [2] 156.3	0.0658	0
[5] 156.3 [3] 156.3	1.04E-13	1

[5] 156.3	[4] 156.3	1.53E-04	1
[5] 78.13	[1] 78.13	1	0
[5] 78.13	[2] 78.13	0.2321	0
[5] 78.13	[3] 78.13	6.91E-07	1
[5] 78.13	[4] 78.13	4.56E-04	1
[5] 39.06	[1] 39.06	1	0
[5] 39.06	[2] 39.06	1	0
[5] 39.06	[3] 39.06	0.65174	0
[5] 39.06	[4] 39.06	1	0
[5] 19.53	[1] 19.53	1	0
[5] 19.53	[2] 19.53	1	0
[5] 19.53	[3] 19.53	1	0
[5] 19.53	[4] 19.53	1	0
[5] 9.766	[1] 9.766	1	0
[5] 9.766	[2] 9.766	1	0
[5] 9.766	[3] 9.766	1	0
[5] 9.766	[4] 9.766	1	0
[5] 4.883	[1] 4.883	1	0
[5] 4.883	[2] 4.883	1	0
[5] 4.883	[3] 4.883	1	0
[5] 4.883	[4] 4.883	1	0

Table S2 Statistical significances for the MTT-test after 120 min of incubation calculated with the post-hoc test according to Bonferroni, α = 0.05; [1] – **3** (Ro 48-8071), [2] – **4b**, [3] – **4a**, [4] – **4c**, [5] – **4f**, 1 = significant, 0 = not significant

MTT 120 min			
**Group**		**P**	**Significance**
[2] 156.3	[1] 156.3	1.59E-08	1
[2] 78.13	[1] 78.13	2.83E-09	1
[2] 39.06	[1] 39.06	1	0
[2] 19.53	[1] 19.53	1	0
[2] 9.766	[1] 9.766	1	0
[2] 4.883	[1] 4.883	1	0
[3] 156.3	[1] 156.3	1.00E+00	0
[3] 156.3	[2] 156.3	3.25E-08	1
[3] 78.13	[1] 78.13	1	0
[3] 78.13	[2] 78.13	2.60E-09	1
[3] 39.06	[1] 39.06	7.68E-04	1
[3] 39.06	[2] 39.06	1.16E-08	1
[3] 19.53	[1] 19.53	1	0
[3] 19.53	[2] 19.53	0.0681	0
[3] 9.766	[1] 9.766	1	0
[3] 9.766	[2] 9.766	1	0
[3] 4.883	[1] 4.883	1	0
[3] 4.883	[2] 4.883	1	0
[4] 156.3	[1] 156.3	1	0
[4] 156.3	[2] 156.3	9.03E-09	1
[4] 156.3	[3] 156.3	1	0

[4] 78.13	[1] 78.13	1	0
[4] 78.13	[2] 78.13	1.07E-10	1
[4] 78.13	[3] 78.13	1	0
[4] 39.06	[1] 39.06	4.60E-07	1
[4] 39.06	[2] 39.06	1.90E-12	1
[4] 39.06	[3] 39.06	1	0
[4] 19.53	[1] 19.53	0.15376	0
[4] 19.53	[2] 19.53	0.00269	1
[4] 19.53	[3] 19.53	1	0
[4] 9.766	[1] 9.766	1	0
[4] 9.766	[2] 9.766	1	0
[4] 9.766	[3] 9.766	1	0
[4] 4.883	[1] 4.883	1	0
[4] 4.883	[2] 4.883	1	0
[4] 4.883	[3] 4.883	1	0
[5] 156.3	[1] 156.3	1.13E-06	1
[5] 156.3	[2] 156.3	1	0
[5] 156.3	[3] 156.3	2.22E-06	1
[5] 156.3	[4] 156.3	6.64E-07	1
[5] 78.13	[1] 78.13	2.54E-11	1
[5] 78.13	[2] 78.13	1	0
[5] 78.13	[3] 78.13	2.32E-11	1
[5] 78.13	[4] 78.13	8.51E-13	1
[5] 39.06	[1] 39.06	0.16274	0
[5] 39.06	[2] 39.06	1	0
[5] 39.06	[3] 39.06	3.90E-12	1
[5] 39.06	[4] 39.06	4.06E-16	1
[5] 19.53	[1] 19.53	1	0
[5] 19.53	[2] 19.53	1	0
[5] 19.53	[3] 19.53	3.53E-04	1
[5] 19.53	[4] 19.53	7.68E-06	1
[5] 9.766	[1] 9.766	1	0
[5] 9.766	[2] 9.766	1	0
[5] 9.766	[3] 9.766	0.93588	0
[5] 9.766	[4] 9.766	1	0
[5] 4.883	[1] 4.883	1	0
[5] 4.883	[2] 4.883	1	0
[5] 4.883	[3] 4.883	1	0
[5] 4.883	[4] 4.883	1	0

Table S3 Statistical significances for the minimal trophicidal concentration test (MTC) calculated with the post-hoc test according to Bonferroni, α = 0.05; [1] – **3** (Ro 48-8071), [2] – **4b**, [3] – **4a**, [4] – **4c**, [5] – **4f**, 1 = significant, 0 = not significant

MTC			
Group		P	Significance
[2] 2	[1] 2	0.0671	0
[2] 4	[1] 4	5.71E-06	1
[2] 7	[1] 7	0.00115	1

[2] 14 [1] 14	1	0
[3] 2 [1] 2	1	0
[3] 2 [2] 2	4.47E-05	1
[3] 4 [1] 4	1	0
[3] 4 [2] 4	5.71E-06	1
[3] 7 [1] 7	0.00707	1
[3] 7 [2] 7	1.80E-12	1
[3] 14 [1] 14	1.59E-07	1
[3] 14 [2] 14	3.90E-12	1
[4] 2 [1] 2	1	0
[4] 2 [2] 2	4.47E-05	1
[4] 2 [3] 2	1	0
[4] 4 [1] 4	0.06683	0
[4] 4 [2] 4	8.01E-14	1
[4] 4 [3] 4	0.06683	0
[4] 7 [1] 7	1	0
[4] 7 [2] 7	8.74E-05	1
[4] 7 [3] 7	0.06683	0
[4] 14 [1] 14	0.50291	0
[4] 14 [2] 14	1.69E-04	1
[4] 14 [3] 14	0.06683	0
[5] 2 [1] 2	0.0071	1
[5] 2 [2] 2	1	0
[5] 2 [3] 2	2.83E-06	1
[5] 2 [4] 2	2.83E-06	1
[5] 4 [1] 4	6.80E-07	1
[5] 4 [2] 4	1	0
[5] 4 [3] 4	6.80E-07	1
[5] 4 [4] 4	7.76E-15	1
[5] 7 [1] 7	1.14E-05	1
[5] 7 [2] 7	1	0
[5] 7 [3] 7	7.76E-15	1
[5] 7 [4] 7	6.80E-07	1
[5] 14 [1] 14	0.31113	0
[5] 14 [2] 14	1	0
[5] 14 [3] 14	1.69E-14	1
[5] 14 [4] 14	1.39E-06	1

Table S4 Statistical significances for the minimal cysticidal concentration test (MCC) calculated with the post-hoc test according to Bonferroni, α = 0.05; [1] – **3** (Ro 48-8071), [2] – **4b**, [3] – **4a**, [4] – **4c**, [5] – **4f**, 1 = significant, 0 = not significant

MCC		
**Group**	**P**	**Significance**
[2] 2 [1] 2	2.80E-10	1
[2] 4 [1] 4	7.65E-10	1
[2] 7 [1] 7	9.83E-10	1
[2] 14 [1] 14	1	0
[3] 2 [1] 2	1	0

[3] 2 [2] 2	2.80E-10	1
[3] 4 [1] 4	1	0
[3] 4 [2] 4	7.65E-10	1
[3] 7 [1] 7	0.07538	0
[3] 7 [2] 7	5.87E-19	1
[3] 14 [1] 14	2.86E-09	1
[3] 14 [2] 14	2.20E-12	1
[4] 2 [1] 2	0.26064	0
[4] 2 [2] 2	0.00279	1
[4] 2 [3] 2	0.26064	0
[4] 4 [1] 4	1	0
[4] 4 [2] 4	2.72E-04	1
[4] 4 [3] 4	1	0
[4] 7 [1] 7	1	0
[4] 7 [2] 7	3.32E-04	1
[4] 7 [3] 7	1.26E-06	1
[4] 14 [1] 14	0.8537	0
[4] 14 [2] 14	0.01021	1
[4] 14 [3] 14	0.00334	1
[5] 2 [1] 2	2.80E-10	1
[5] 2 [2] 2	1	0
[5] 2 [3] 2	2.80E-10	1
[5] 2 [4] 2	0.00279	1
[5] 4 [1] 4	2.65E-09	1
[5] 4 [2] 4	1	0
[5] 4 [3] 4	2.65E-09	1
[5] 4 [4] 4	7.33E-04	1
[5] 7 [1] 7	1.69E-10	1
[5] 7 [2] 7	1	0
[5] 7 [3] 7	8.61E-20	1
[5] 7 [4] 7	7.97E-05	1
[5] 14 [1] 14	1	0
[5] 14 [2] 14	1	0
[5] 14 [3] 14	6.27E-14	1
[5] 14 [4] 14	8.34E-04	1

Table S5 Statistical significances for the comparison of Ro 48-8071 minimal trophicidal concentration (MTC) and minimal cysticidal concentration (MCC) testing calculated with the post-hoc test according to Bonferroni, α = 0.05; [1] – **3** (Ro 48-8071, MTC), [10] – **3** (Ro 48-8071, MCC)1 = significant, 0 = not significant

Ro 48-8071 MTC/MCC		
**Group**	**P**	**Significance**
[10] 2 [1] 2	1.59E-04	1
[10] 4 [1] 4	0.0015	1
[10] 7 [1] 7	7.17E-04	1
[10] 14 [1] 14	1	0

Supplementary NMR spectra

NMR spectra for compounds **3**, **4a**-**4f**, **5**, **6a**-**6b**, **7a**-**7c**, **8a**-**8d**, and **9** can be found on the following pages.

^{1}H NMR (500 MHz, DMSO-d_6) for compound **3**

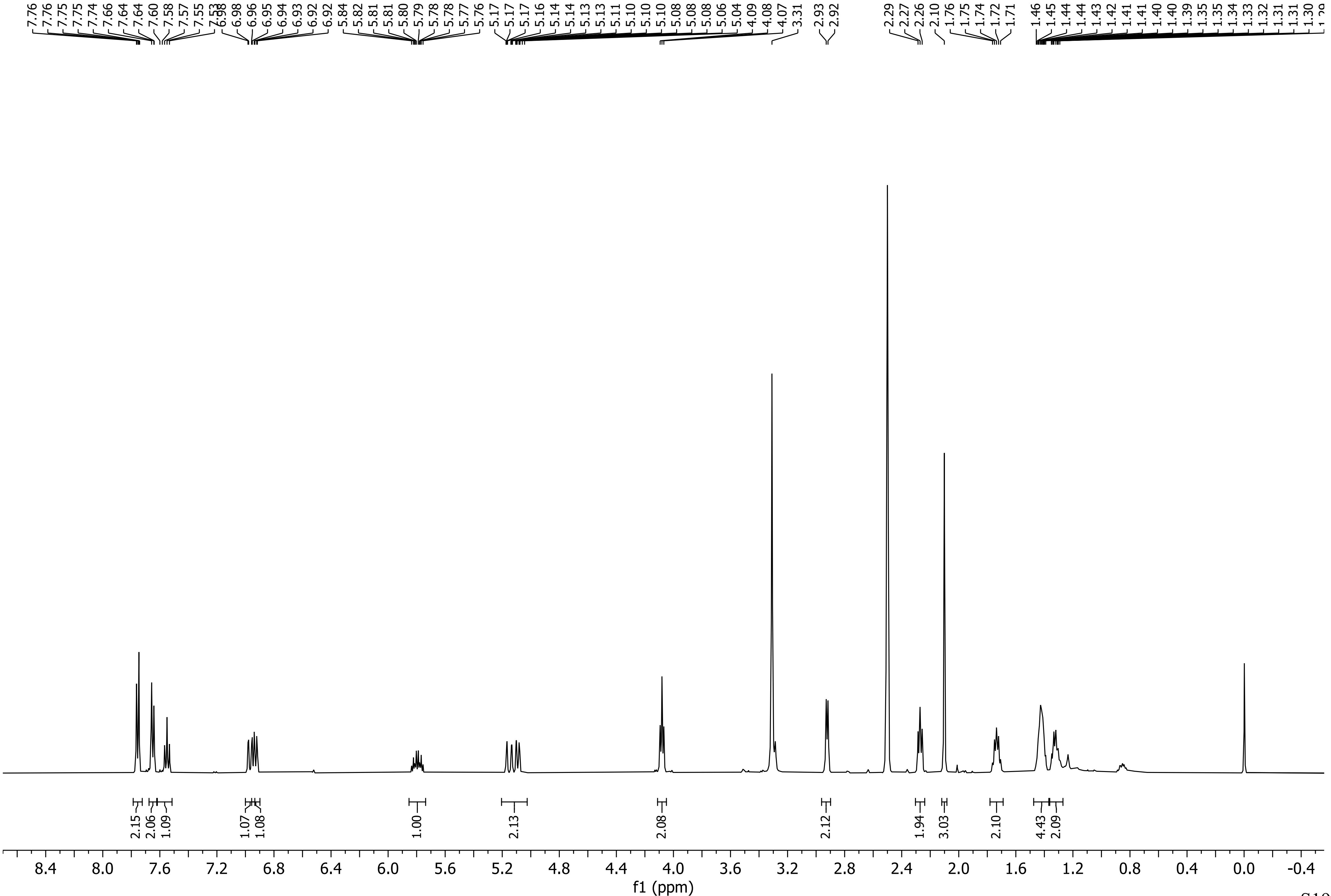

^{13}C NMR (126 MHz, DMSO-d_6) for compound **3**

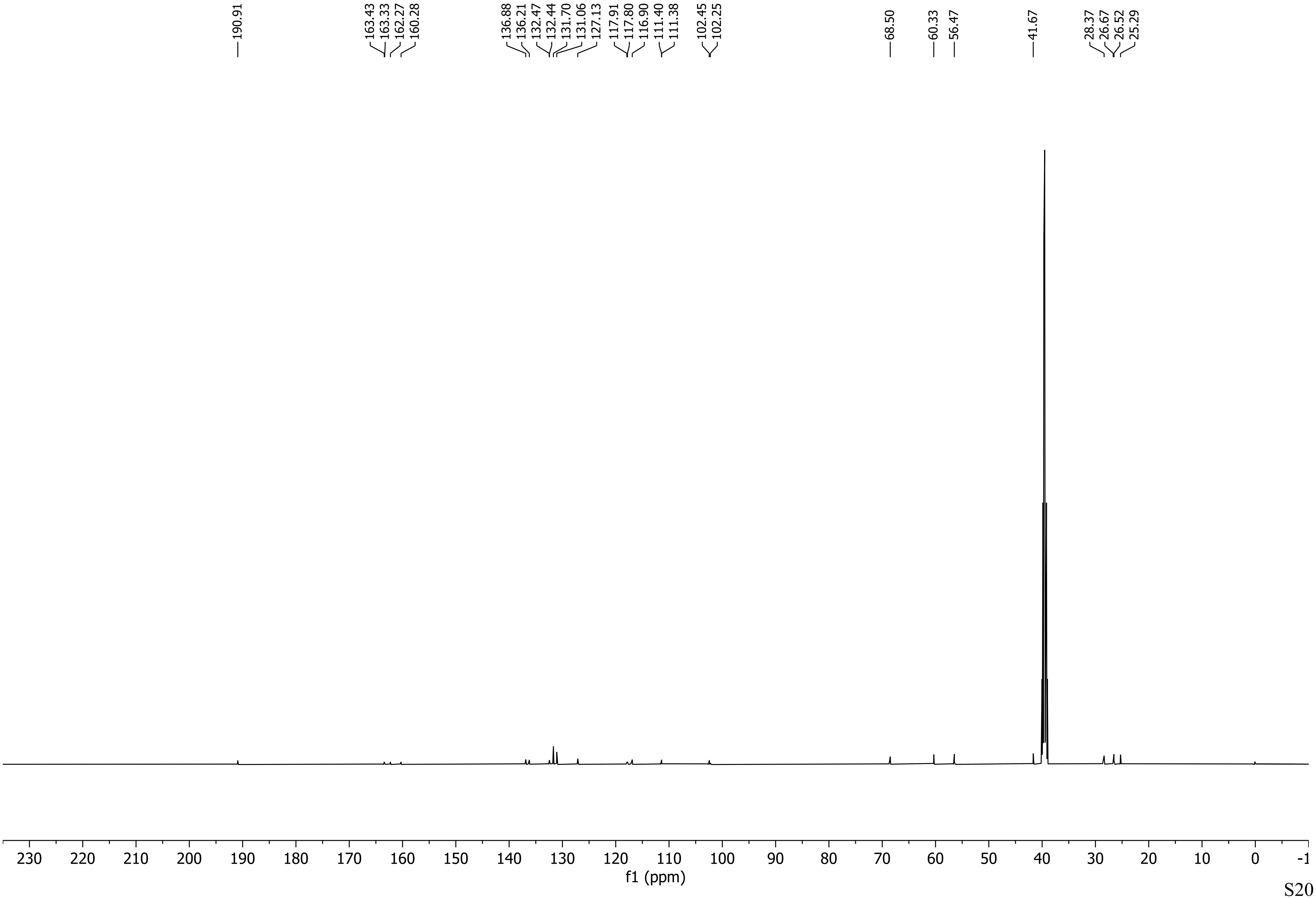

¹³C NMR (126 MHz, DMSO-d_6) for compound **3** (zoomed-in view)

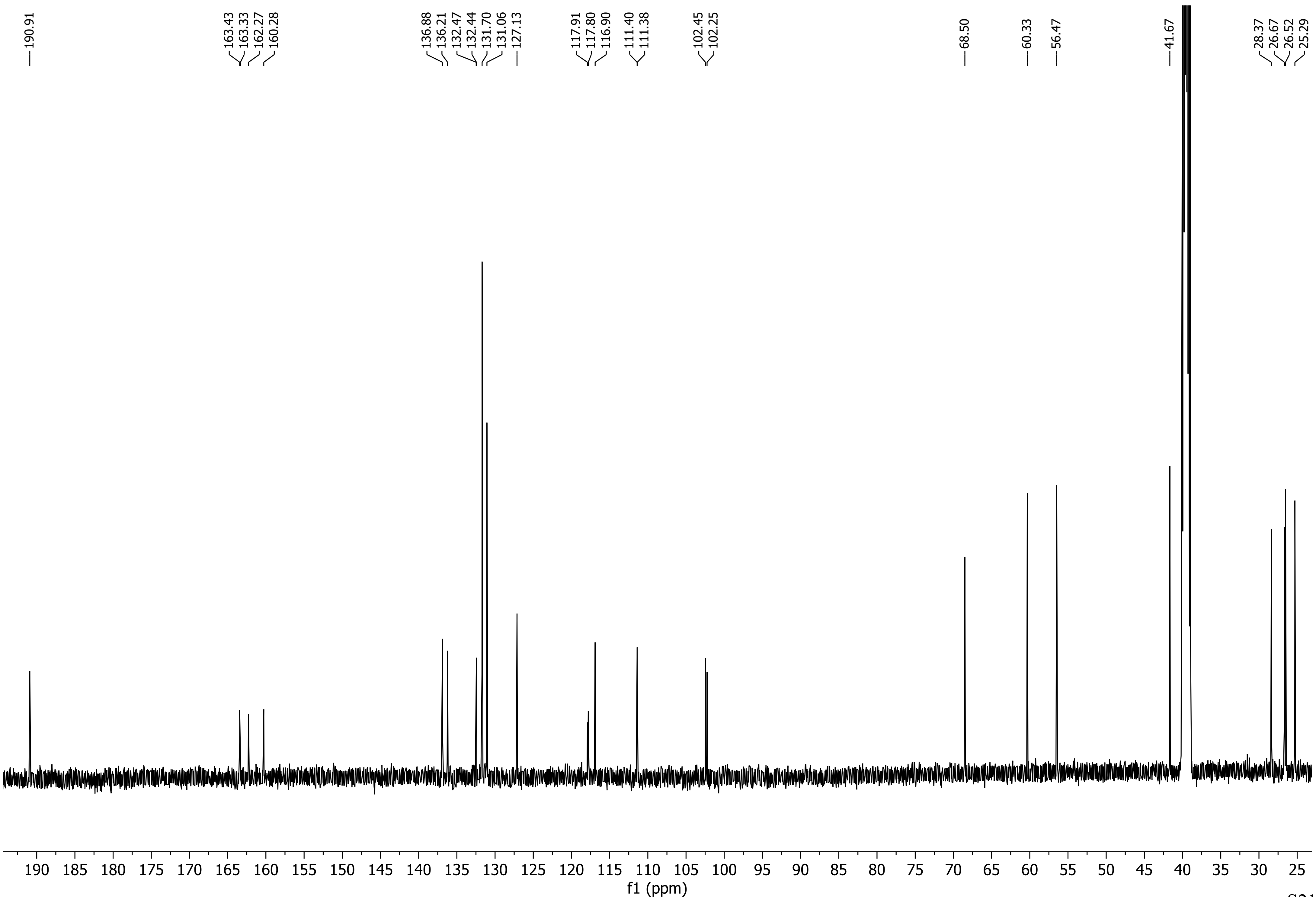

DEPT ^{13}C NMR (126 MHz, DMSO-d_6) for compound **3**

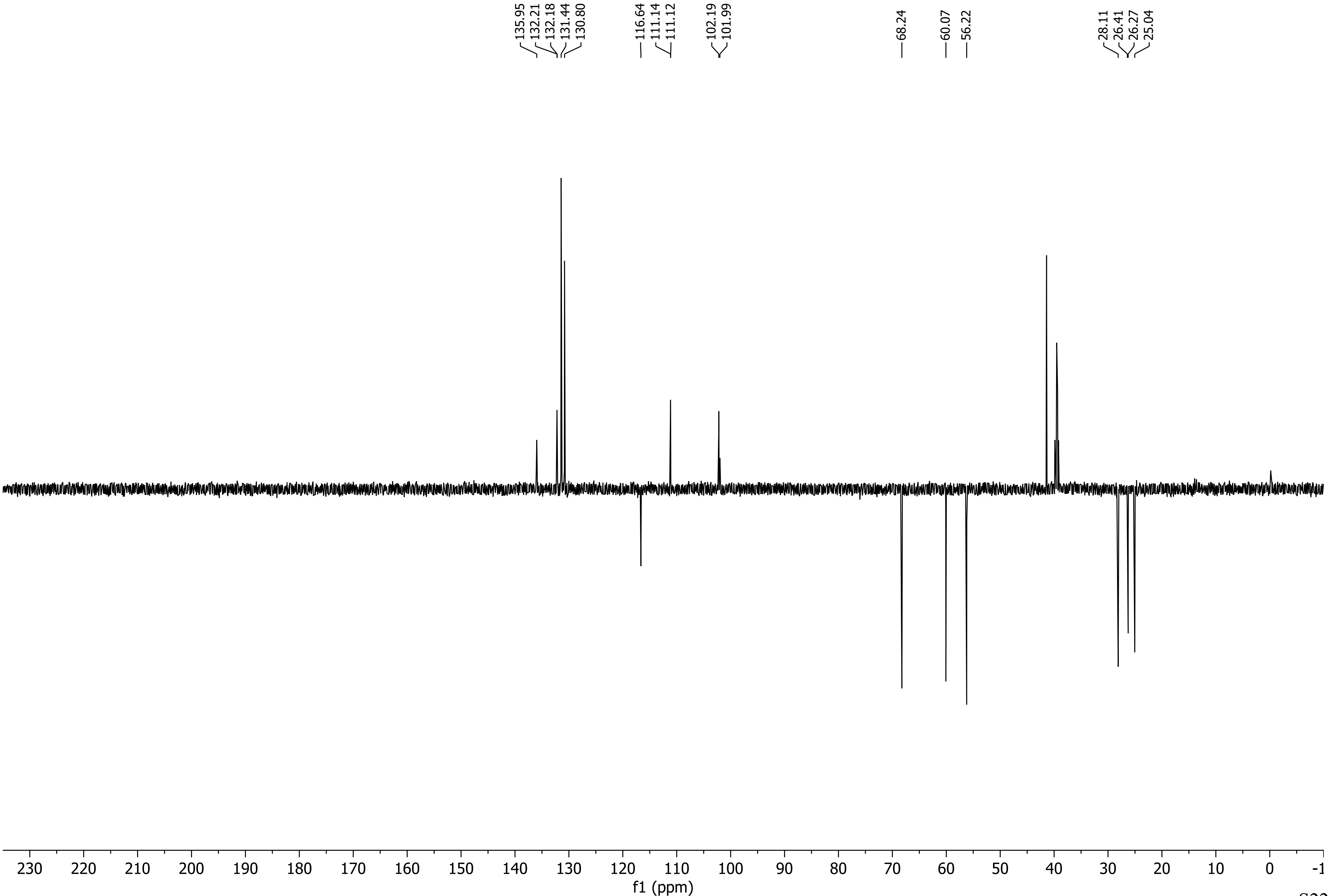

^{1}H NMR (600 MHz, CD_3OD-d_4) for compound **4a** (zoomed-in view)

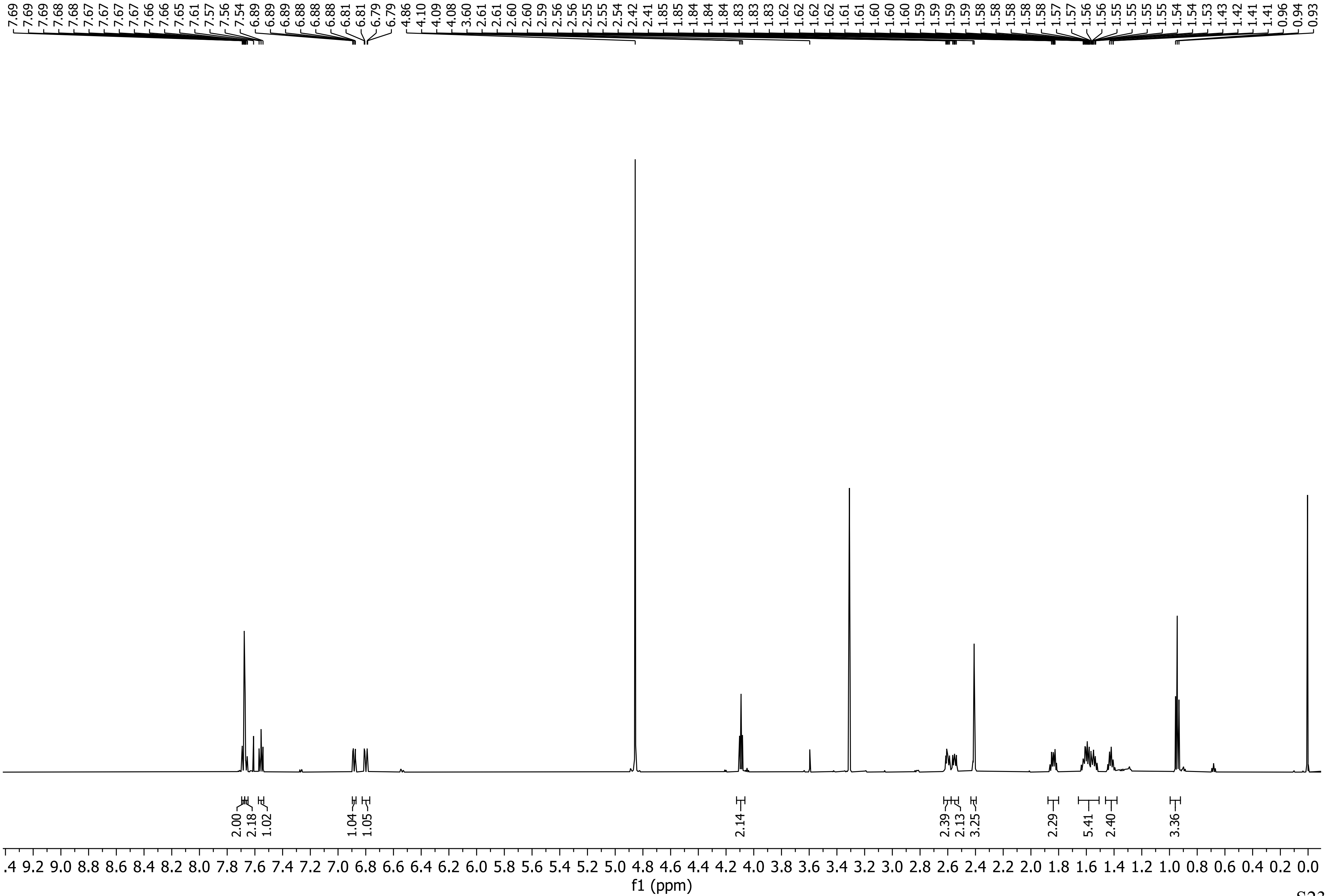

^{13}C NMR (151 MHz, CD_3OD) for compound **4a**

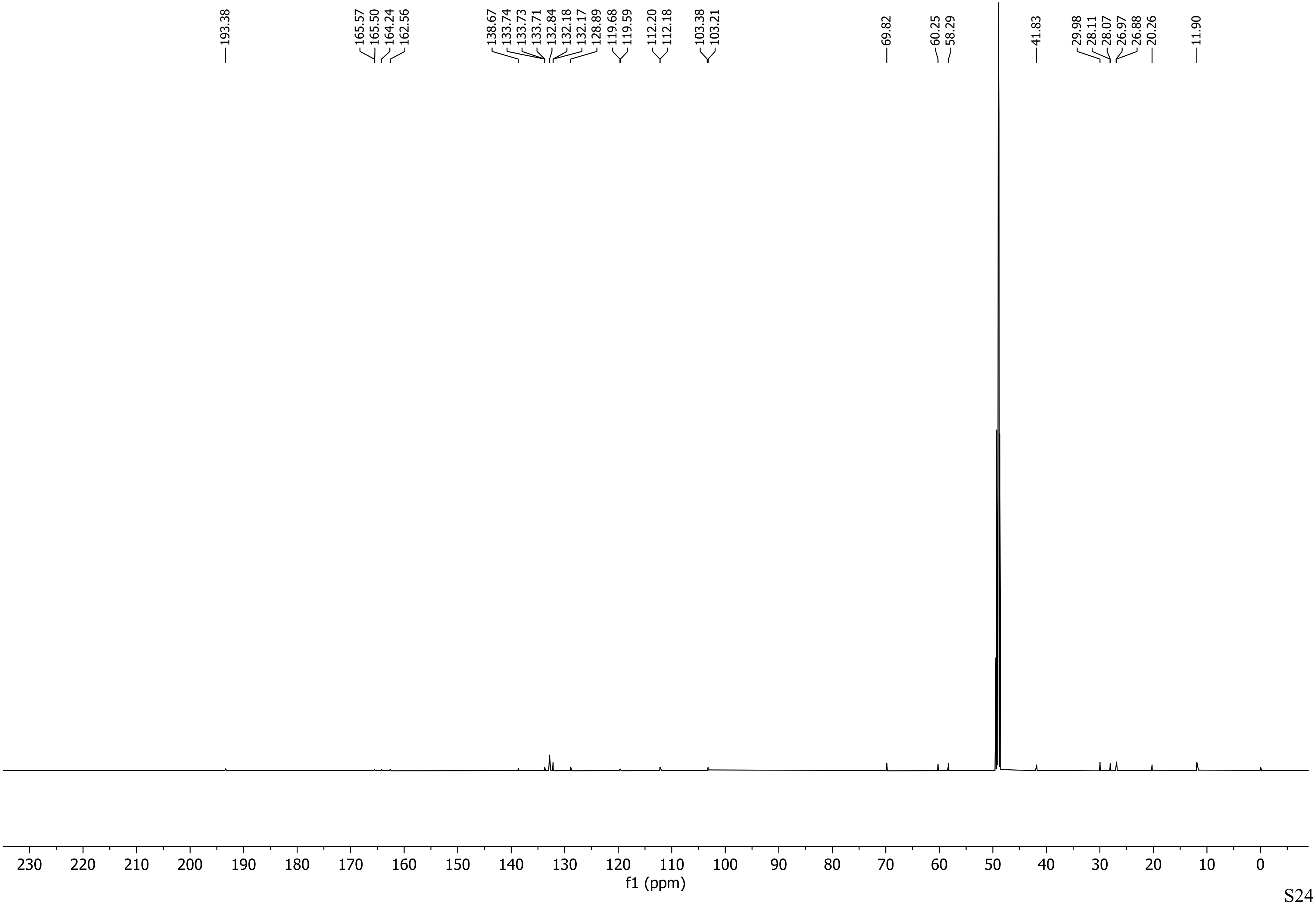

^{13}C NMR (151 MHz, CD_3OD) for compound **4a** (zoomed-in view)

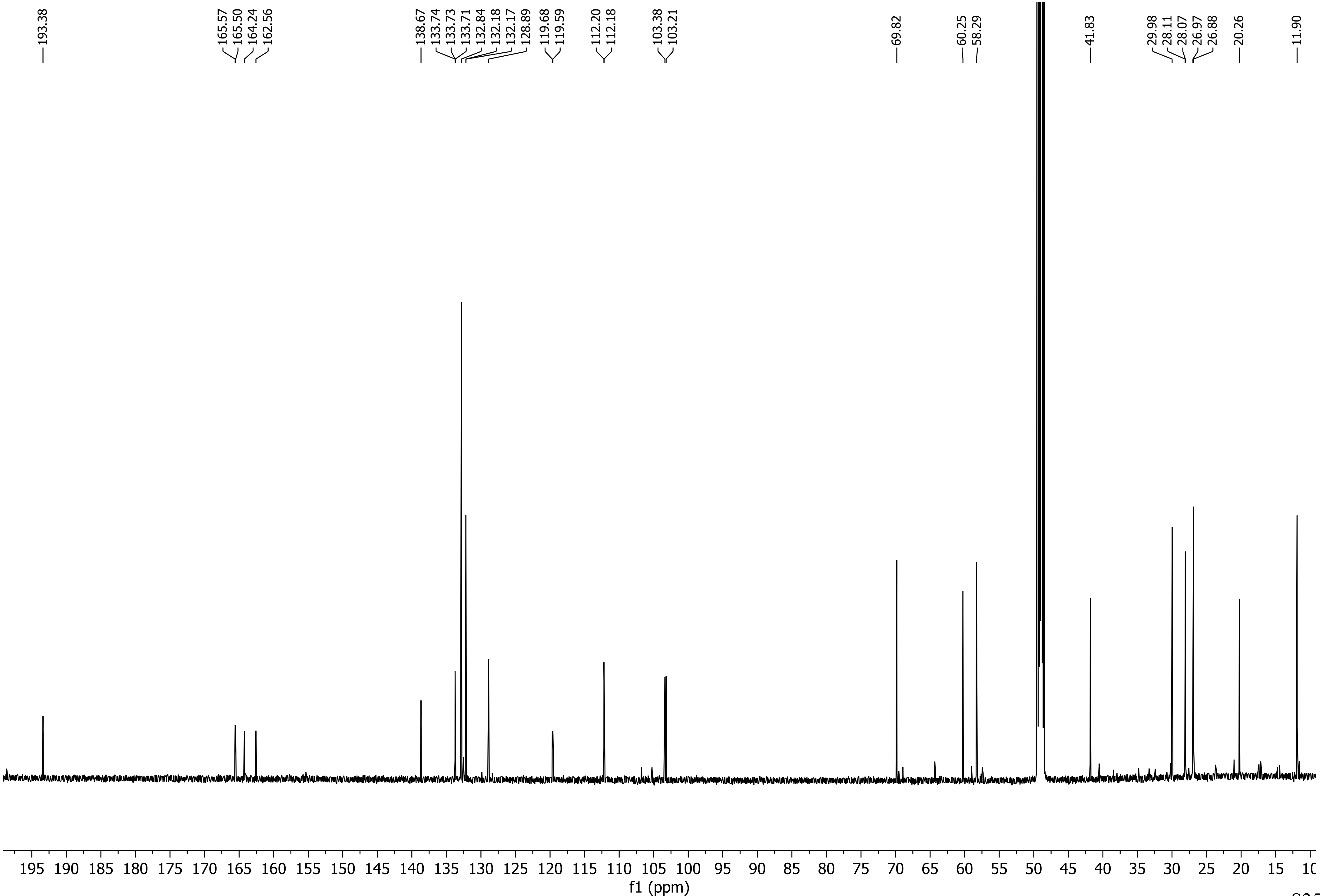

DEPT ^{13}C NMR (151 MHz, CD_3OD) for compound **4a**

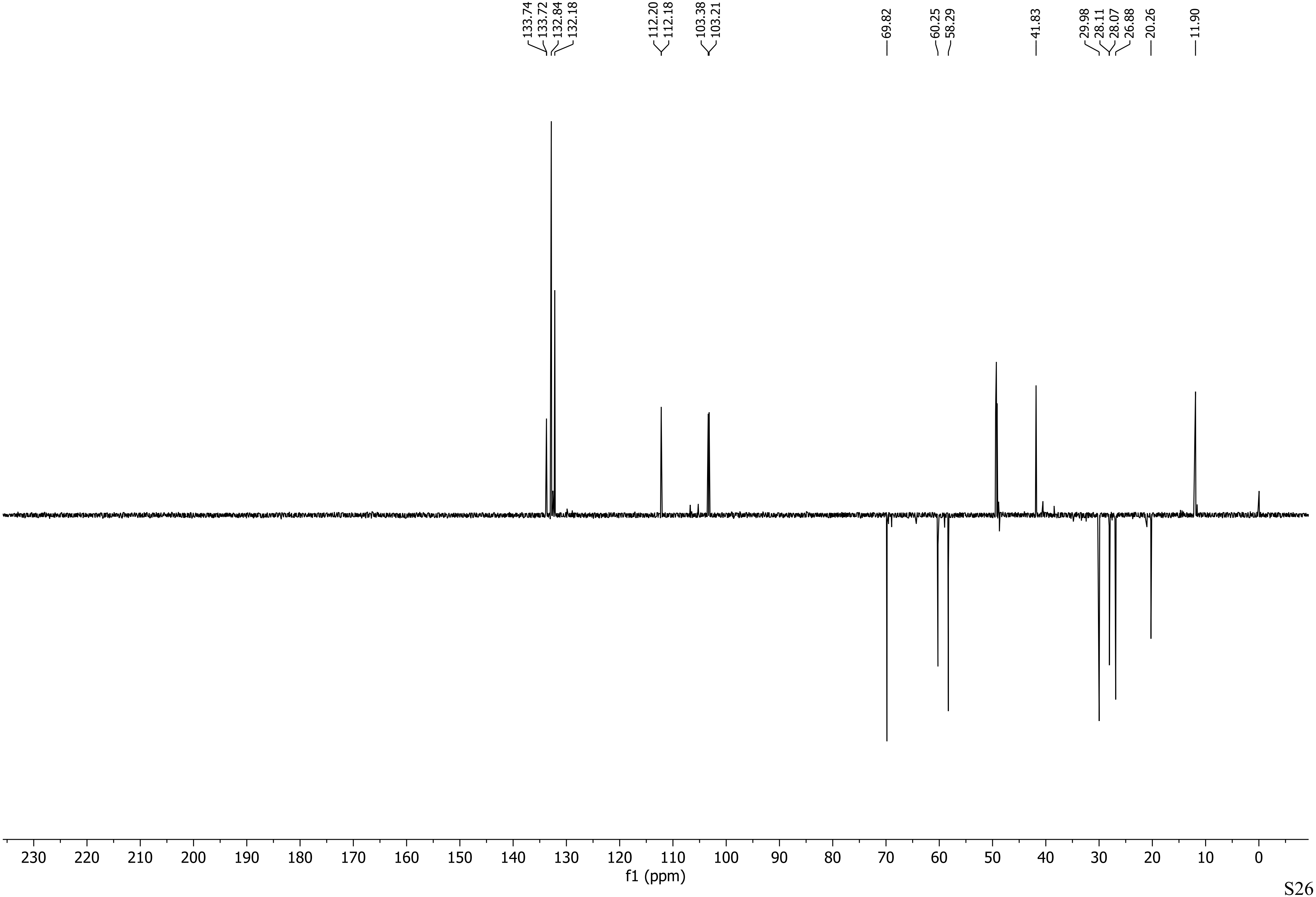

^{1}H NMR (500 MHz, DMSO-d_6) for compound **4b** (zoomed-in view)

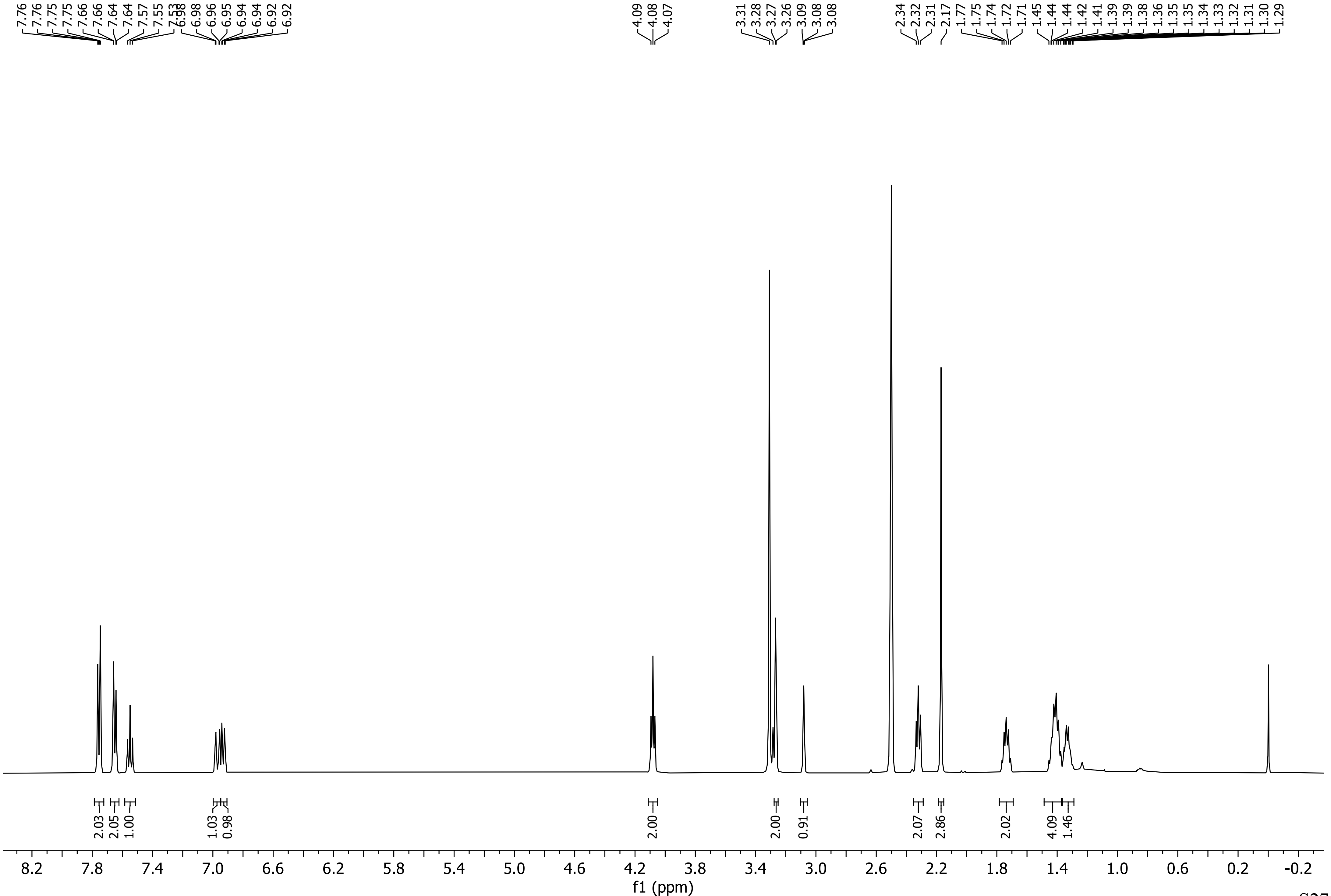

^{13}C NMR (126 MHz, DMSO-d_6) for compound **4b**

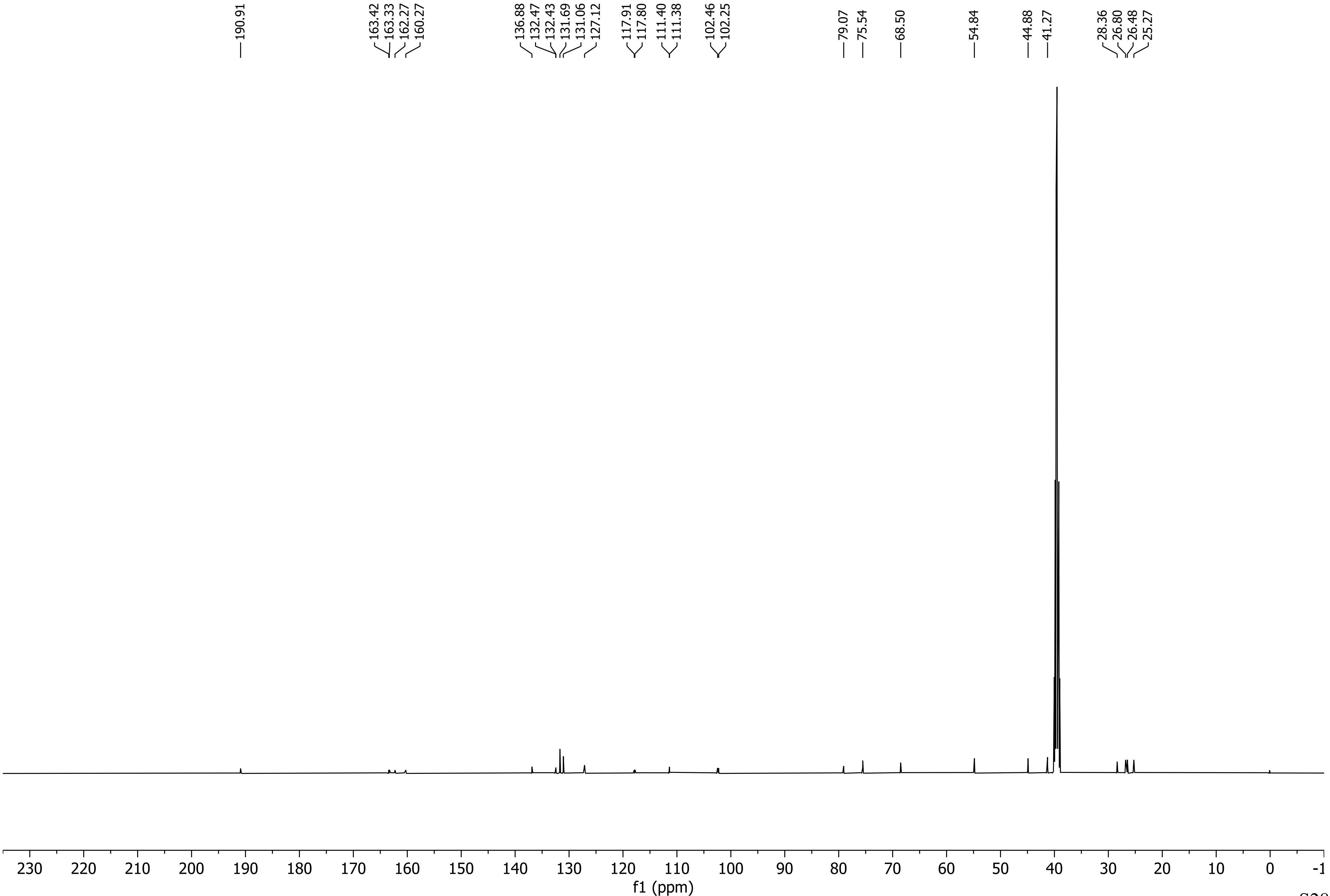

^{13}C NMR (126 MHz, DMSO-d_6) for compound **4b** (zoomed-in view)

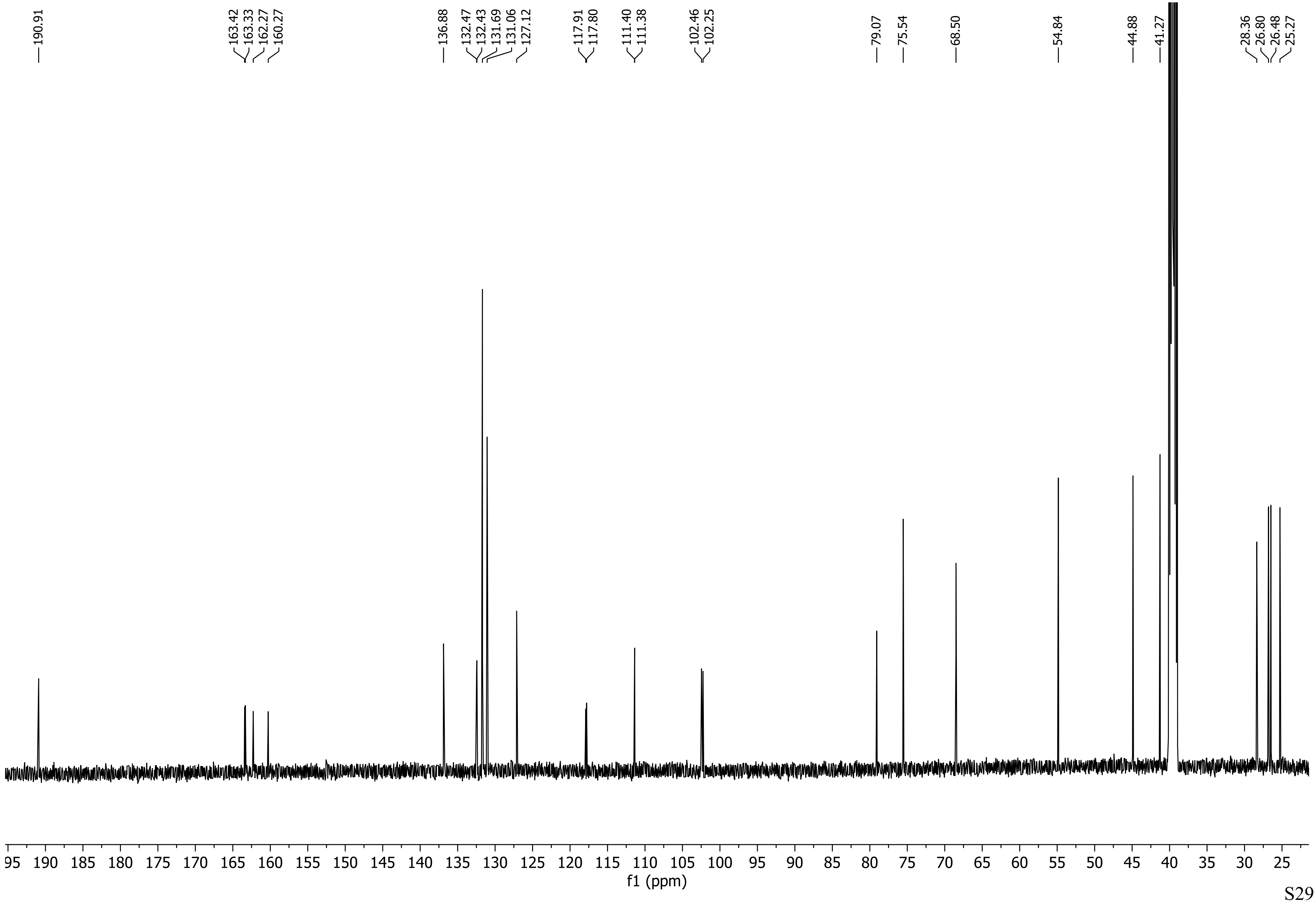

DEPT ¹³C NMR (126 MHz, DMSO-d_6) for compound **4b**

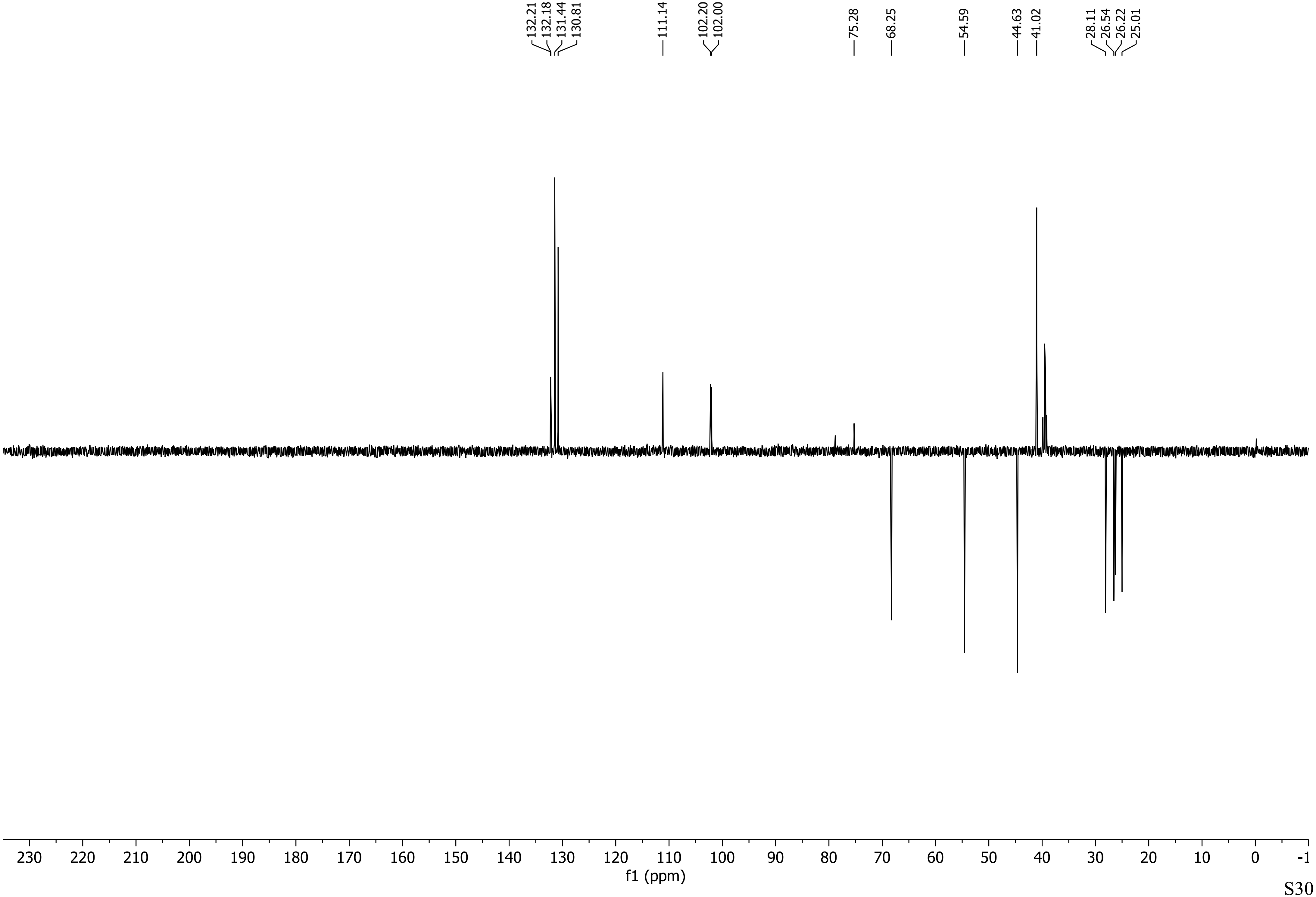

^{1}H NMR (500 MHz, DMSO-d_6) for compound **4c** (zoomed-in view)

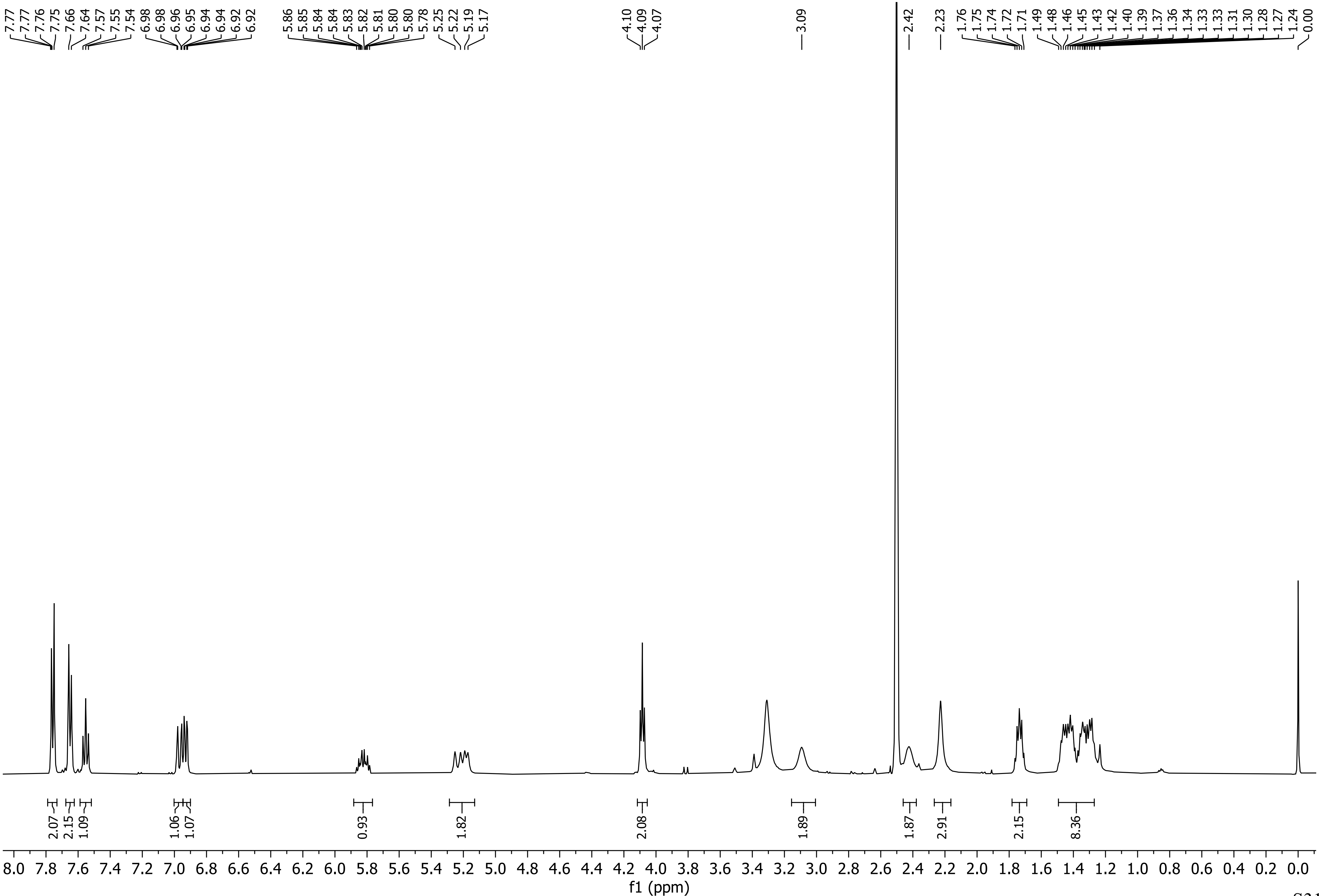

^{13}C NMR (126 MHz, DMSO-d_6) for compound **4c**

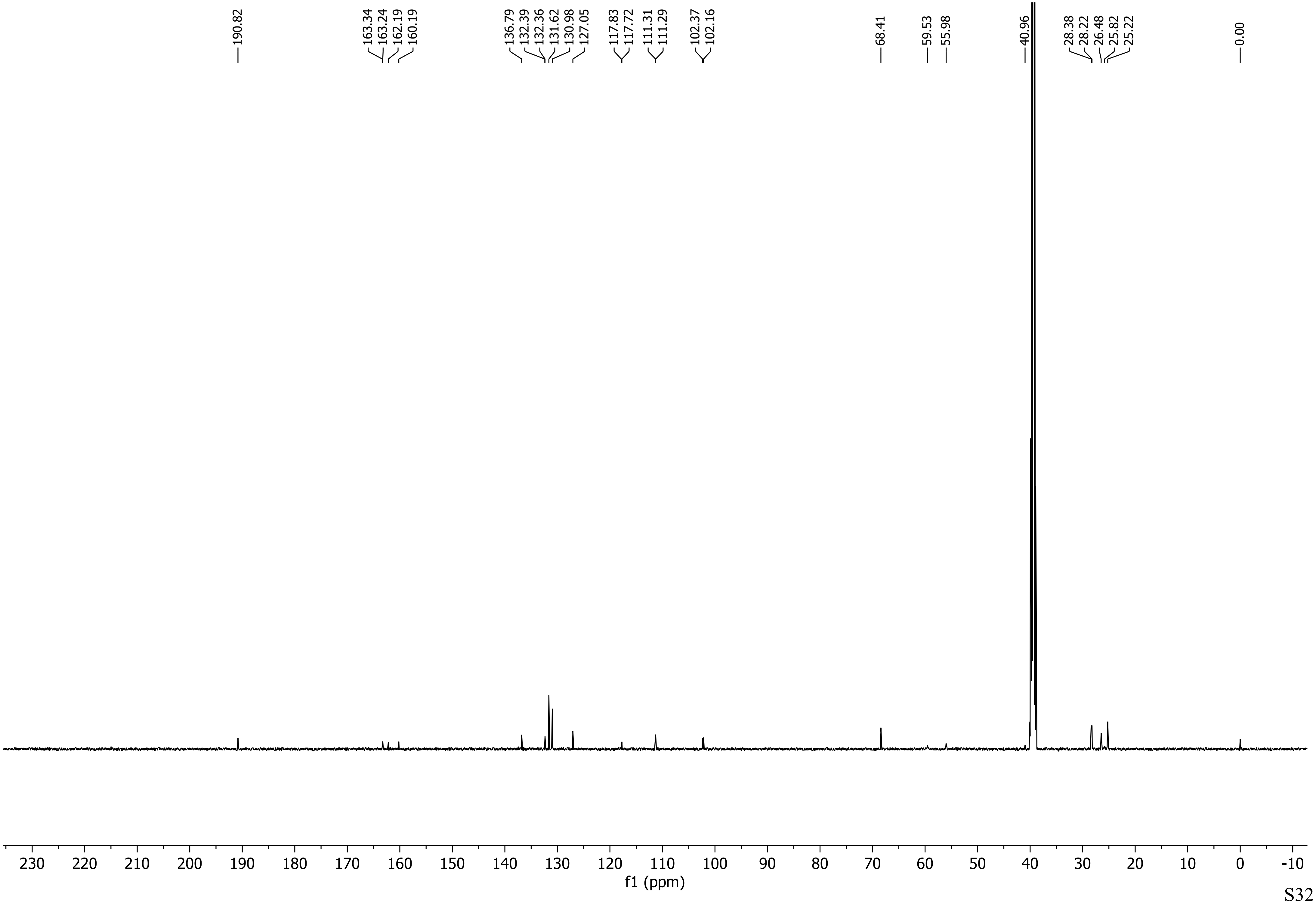

^{13}C NMR (126 MHz, DMSO-d_6) for compound **4c** (zoomed-in view)

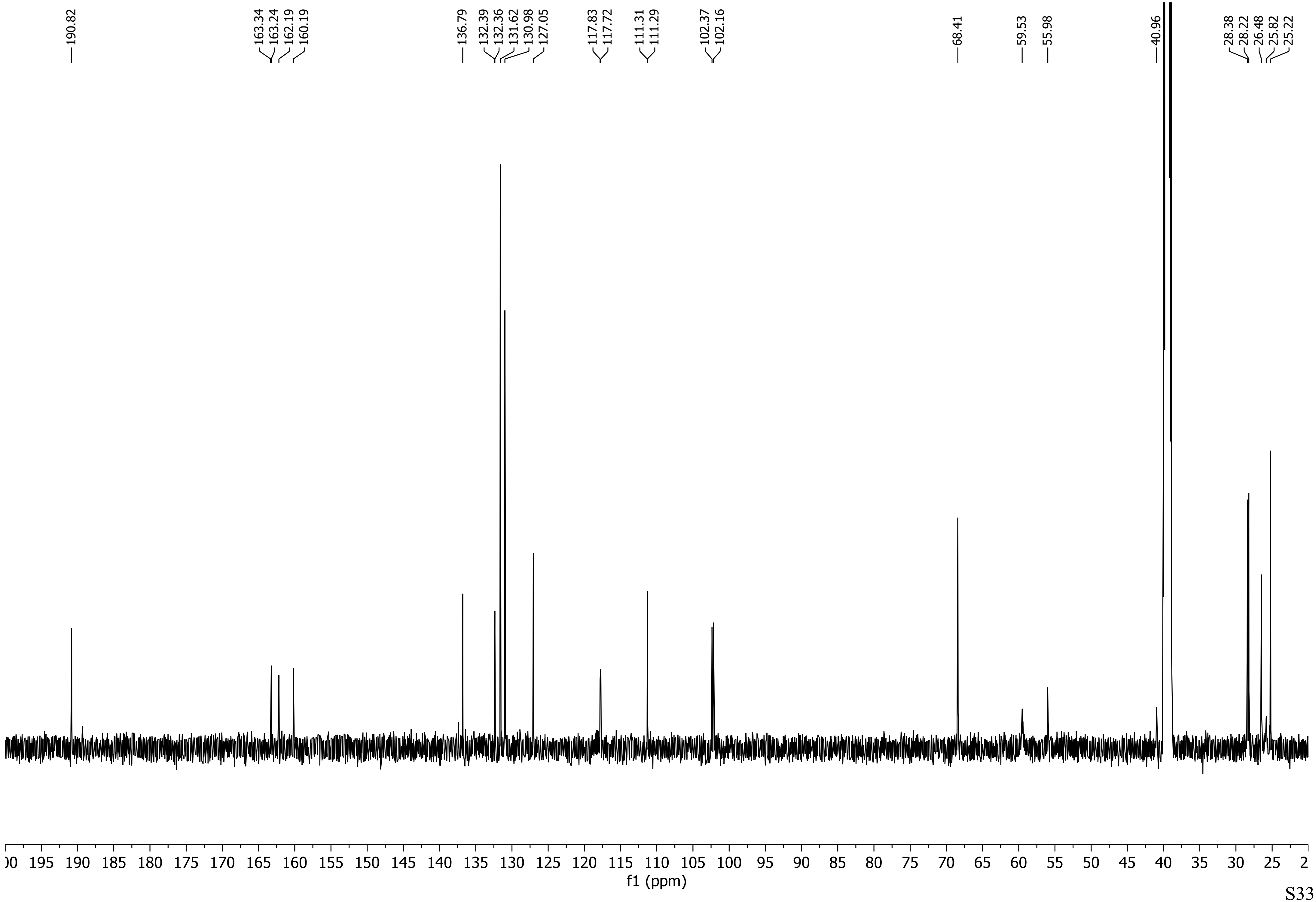

DEPT ^{13}C NMR (126 MHz, DMSO-d_6) for compound **4c** (zoomed-in view)

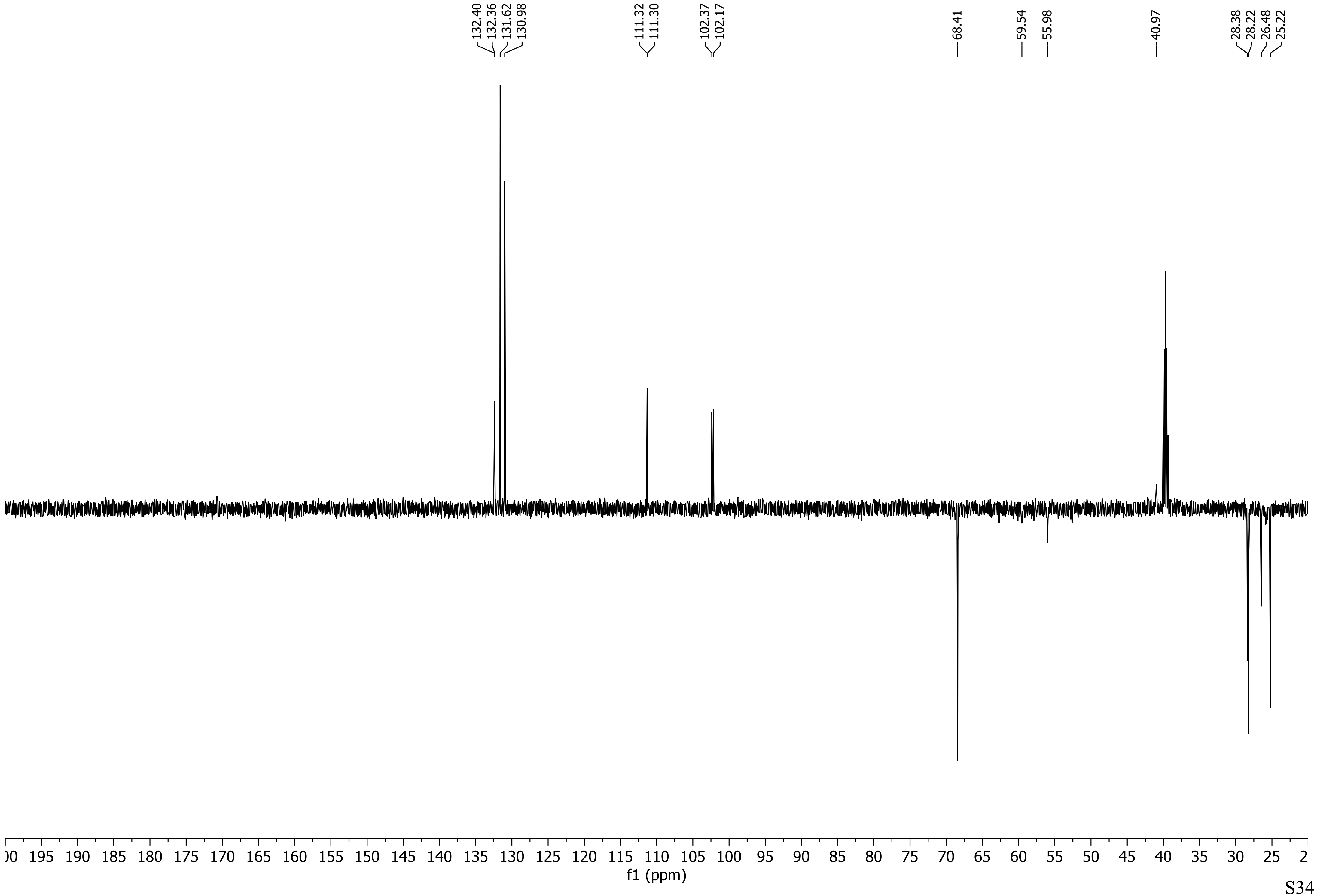

^{1}H NMR (500 MHz, DMSO-d_6) for compound **4d** (zoomed-in view)

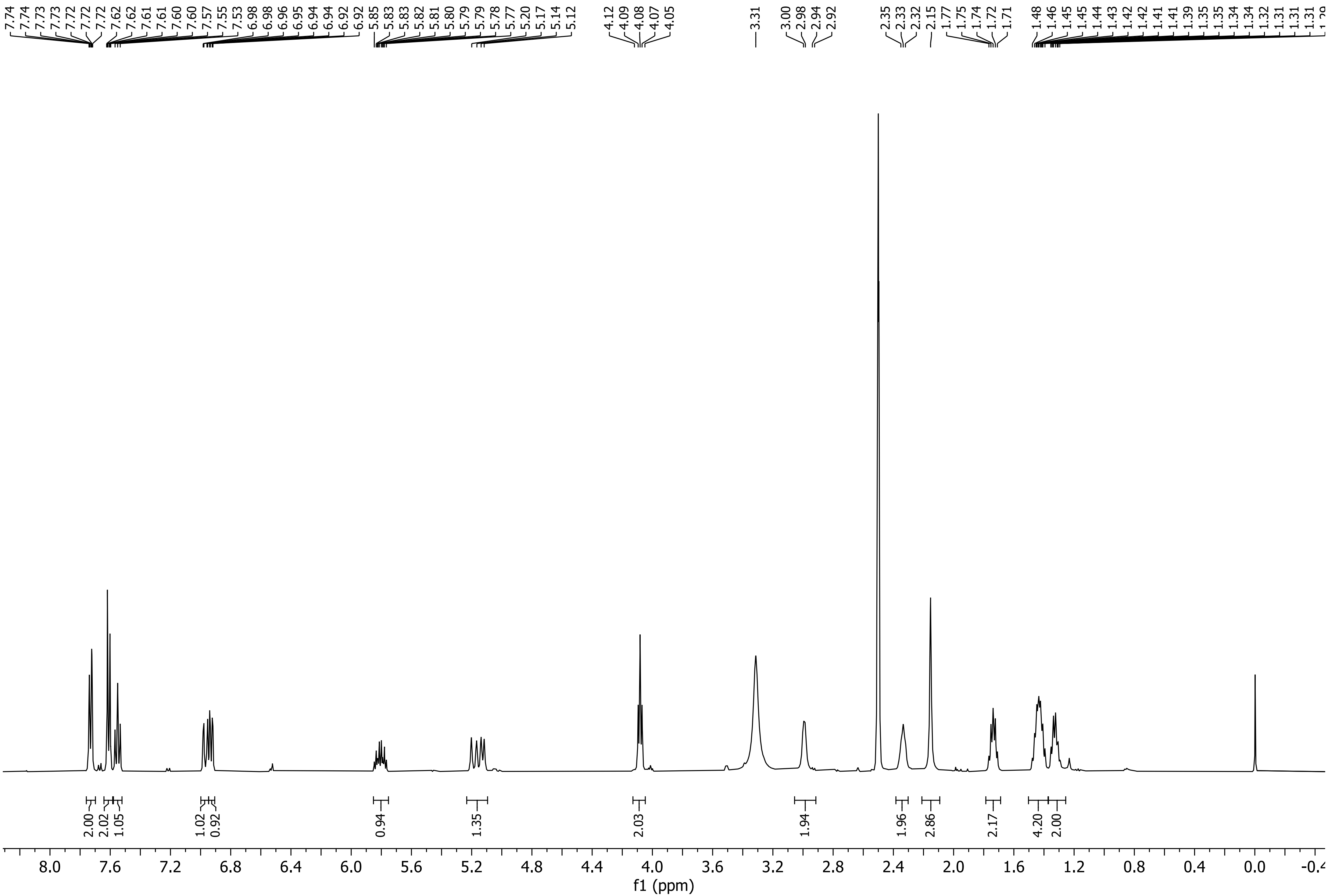

^{13}C NMR (126 MHz, DMSO-d_6) for compound **4d**

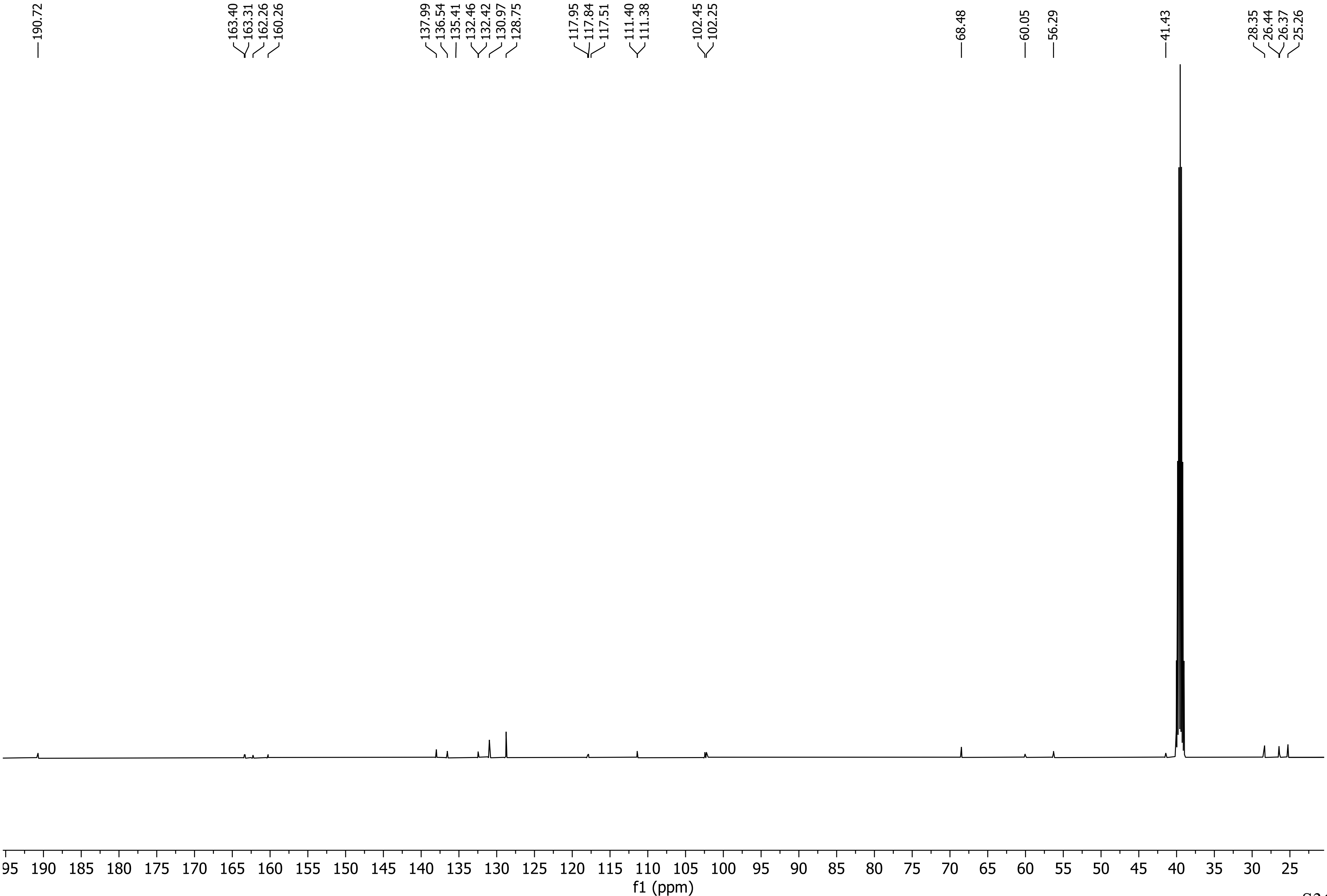

^{13}C NMR (126 MHz, DMSO-d_6) for compound **4d** (zoomed-in view)

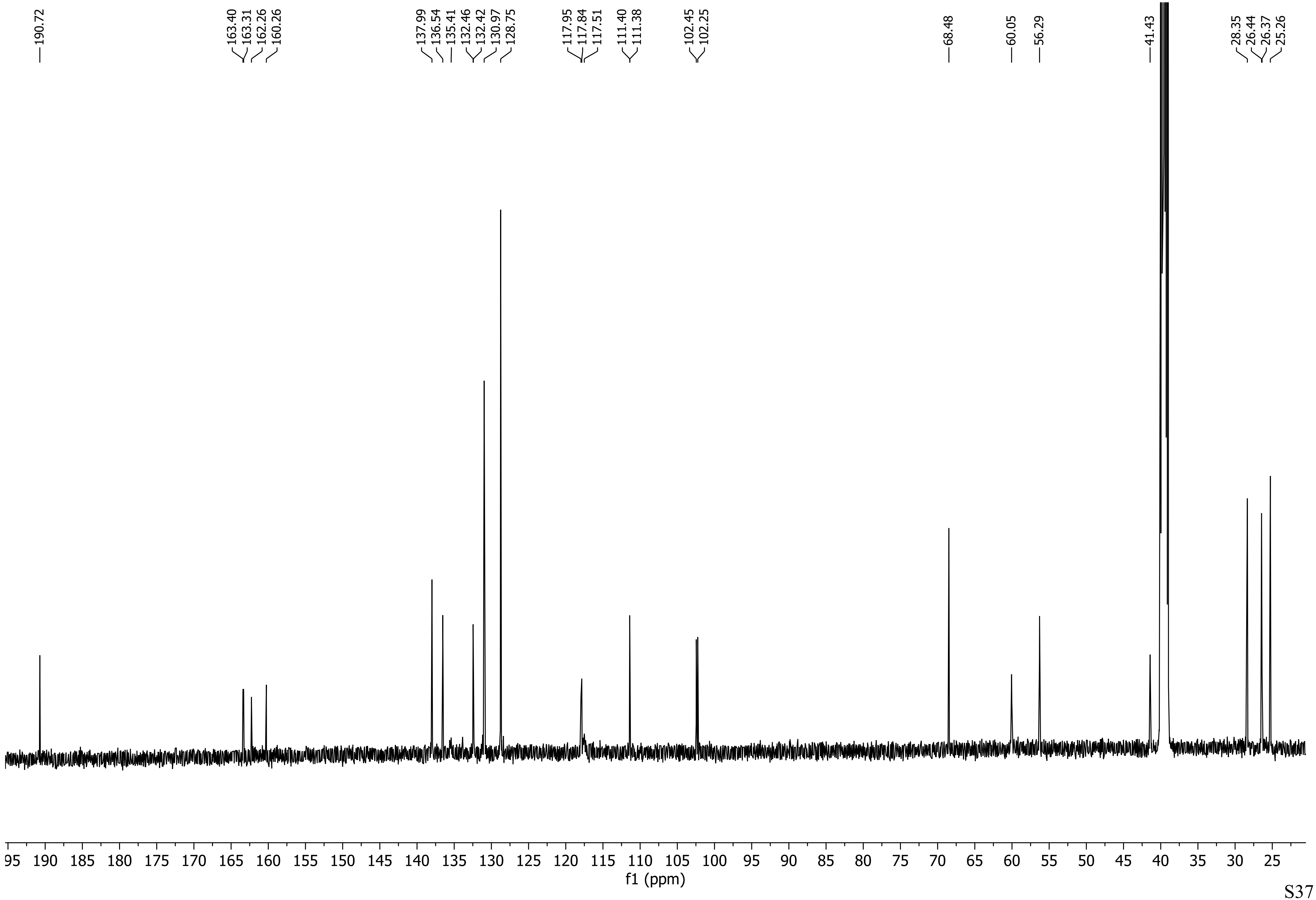

DEPT ^{13}C NMR (126 MHz, DMSO-d_6) for compound **4d**

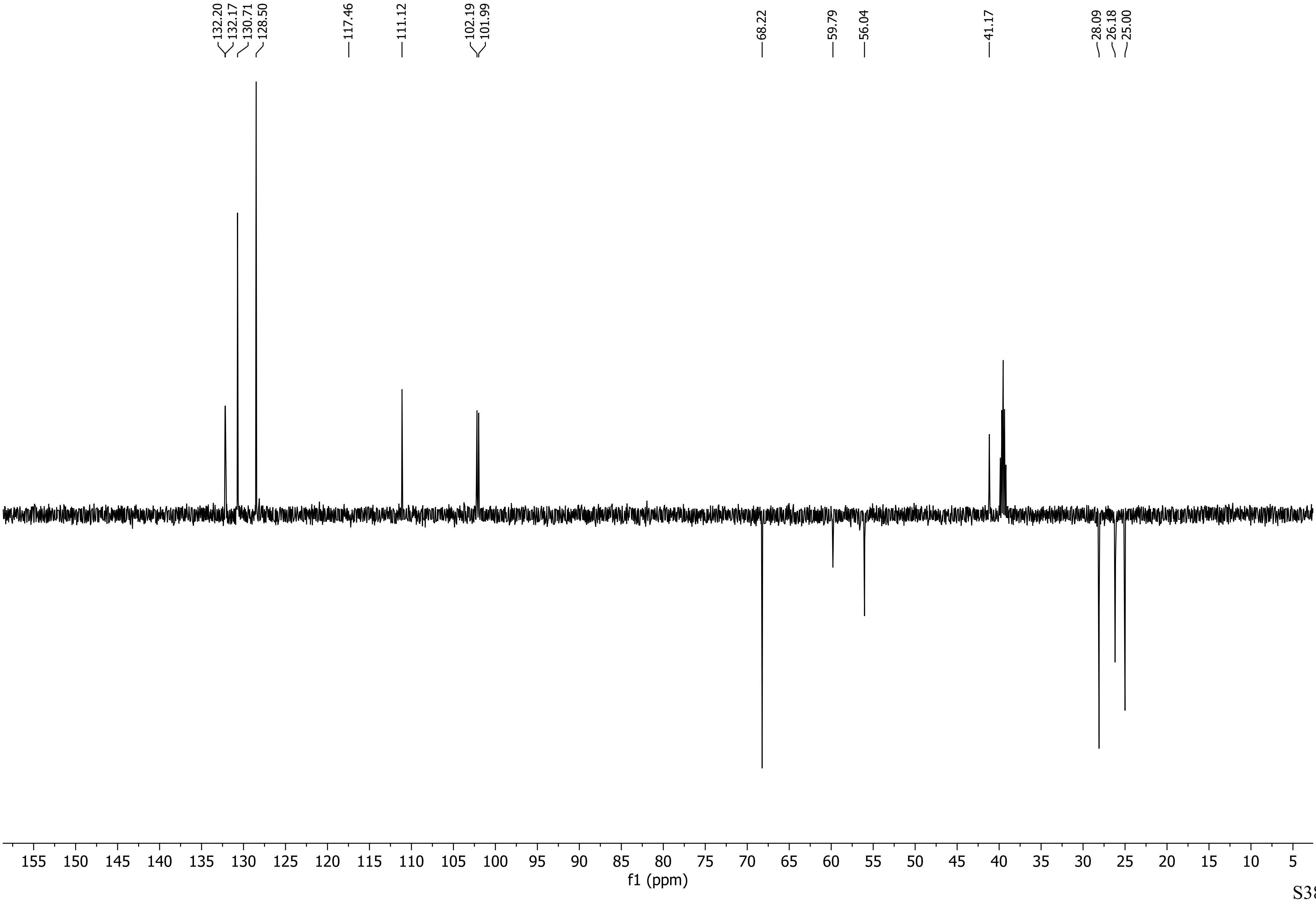

^{1}H NMR (500 MHz, DMSO-d_6) for compound **4e** (zoomed-in view)

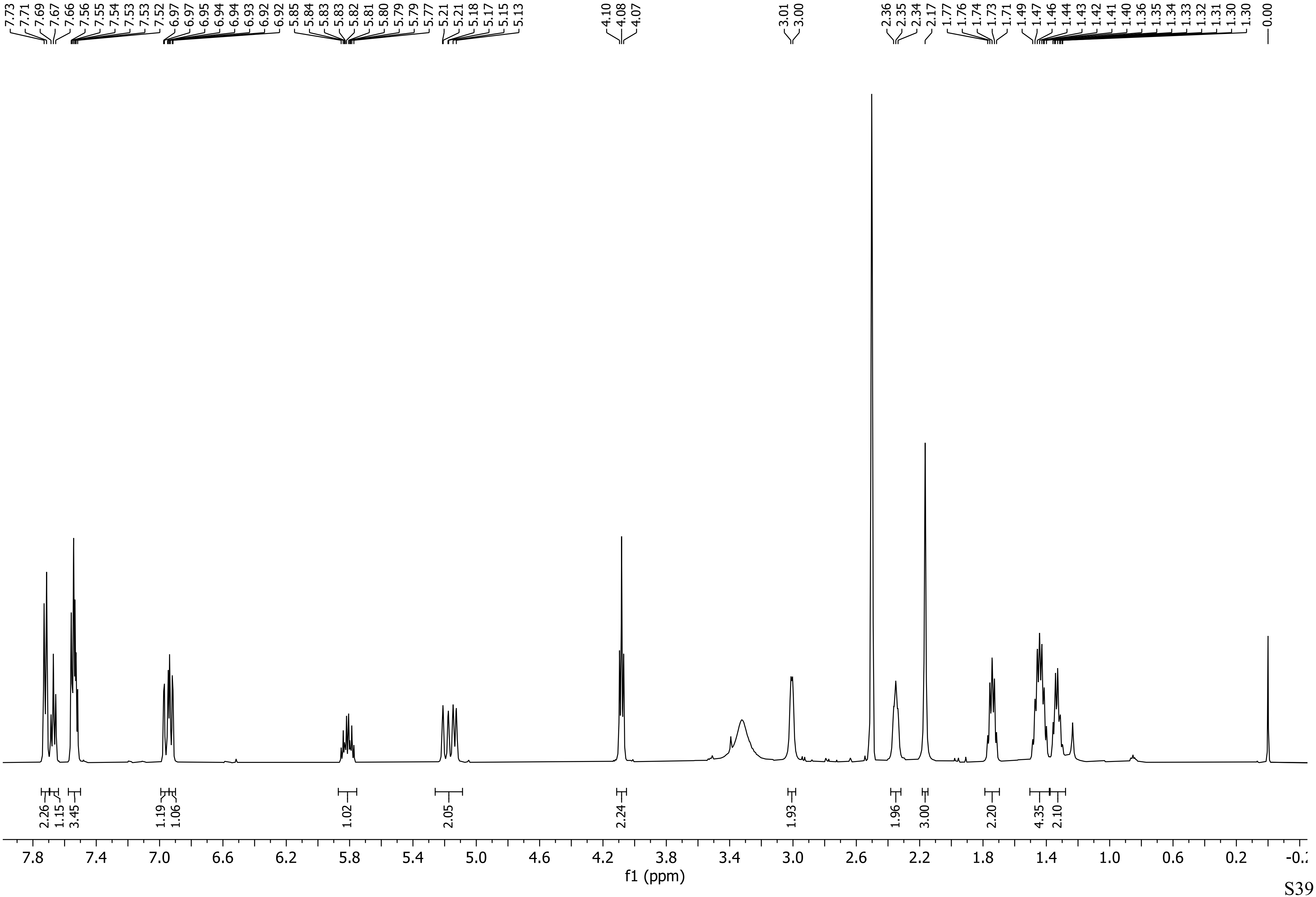

^{13}C NMR (126 MHz, DMSO-d_6) for compound **4e**

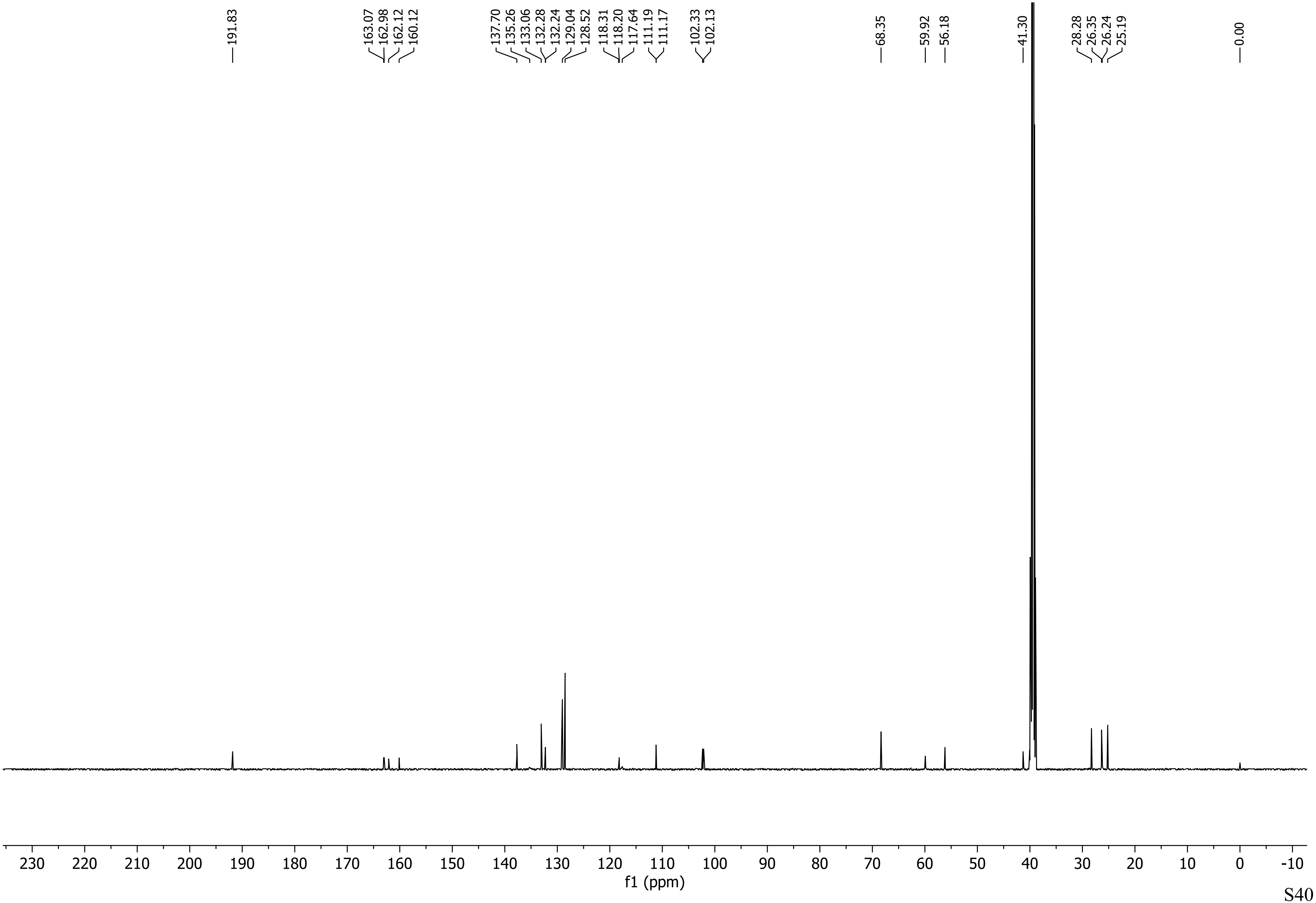

^{13}C NMR (126 MHz, DMSO-d_6) for compound **4e** (zoomed-in view)

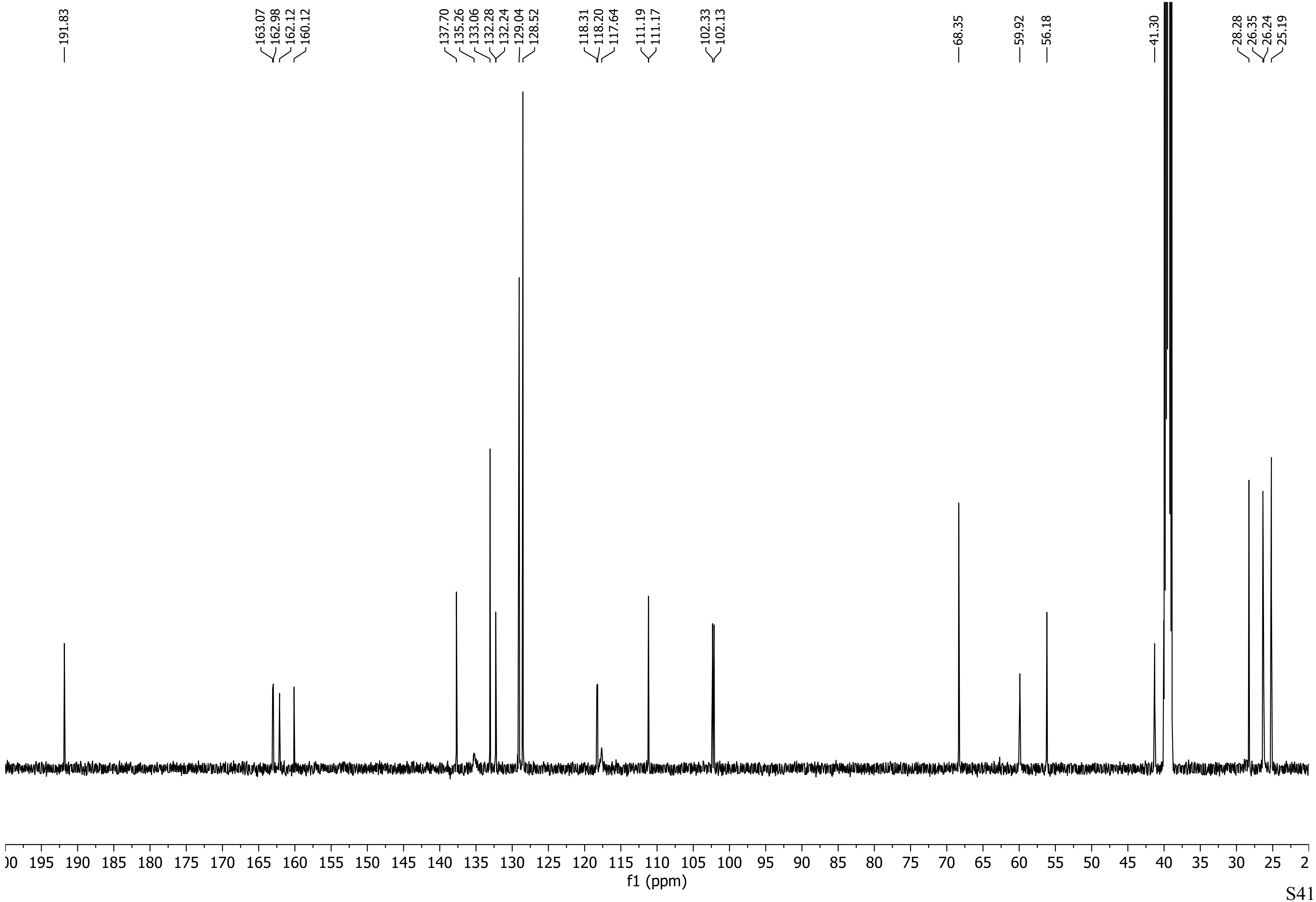

DEPT ^{13}C NMR (126 MHz, DMSO-d_6) for compound **4e** (zoomed-in view)

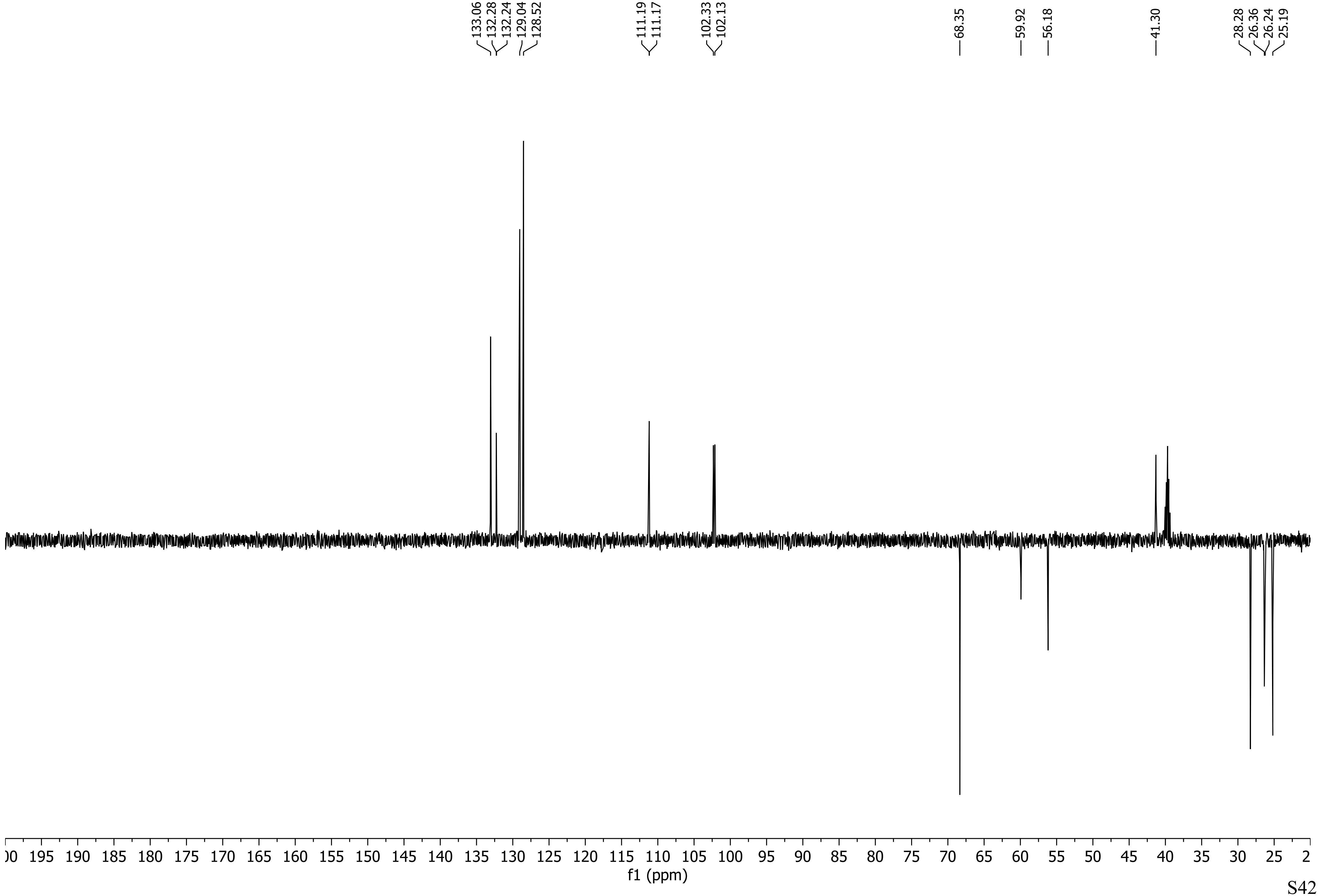

^{1}H NMR (500 MHz, DMSO-d_6) for compound **4f** (zoomed-in view)

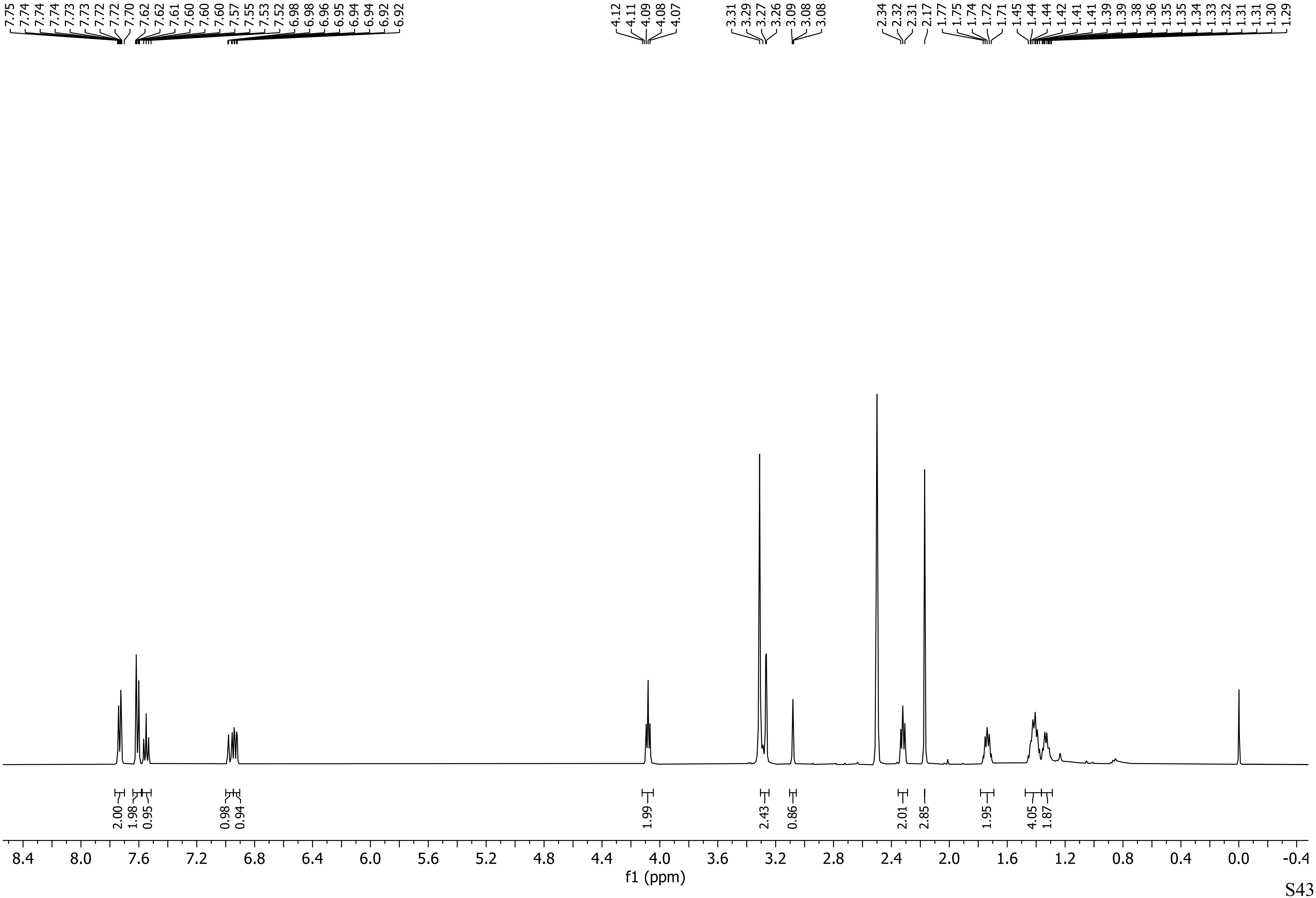

^{13}C NMR NMR (126 MHz, DMSO-d_6) for compound **4f**

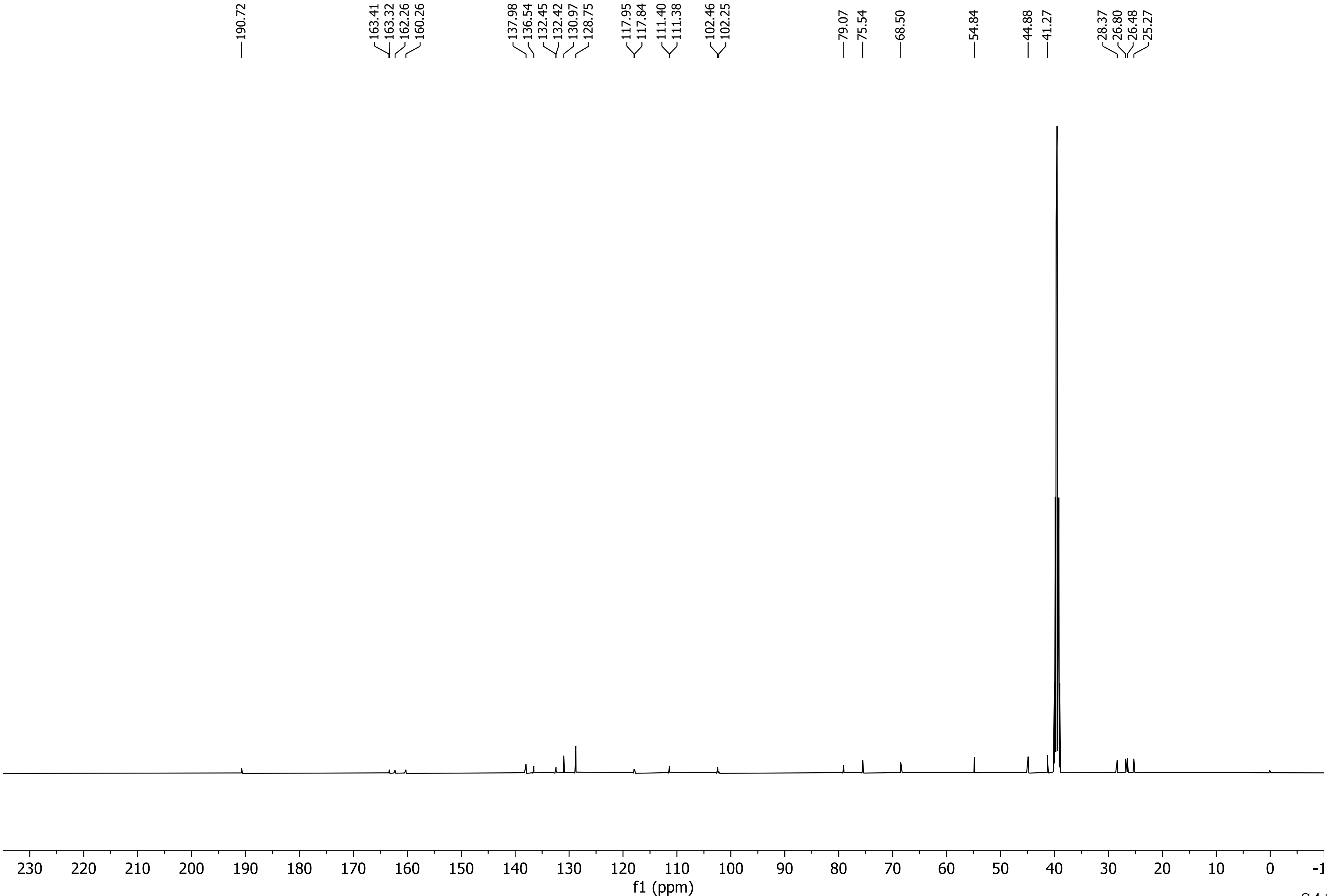

^{13}C NMR NMR (126 MHz, DMSO-d_6) for compound **4f** (zoomed-in view)

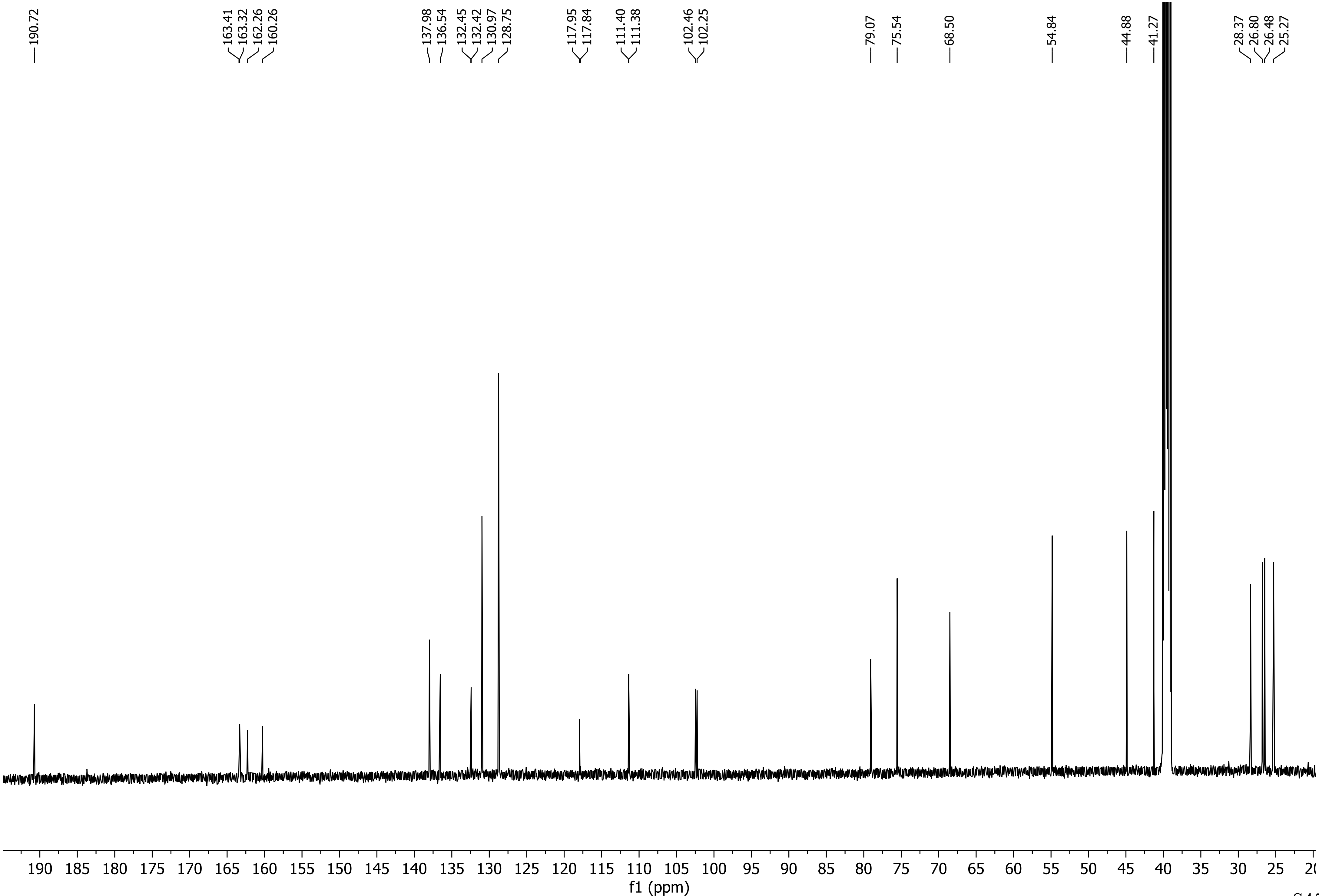

DEPT ^{13}C NMR (126 MHz, DMSO-d_6) for compound **4f**

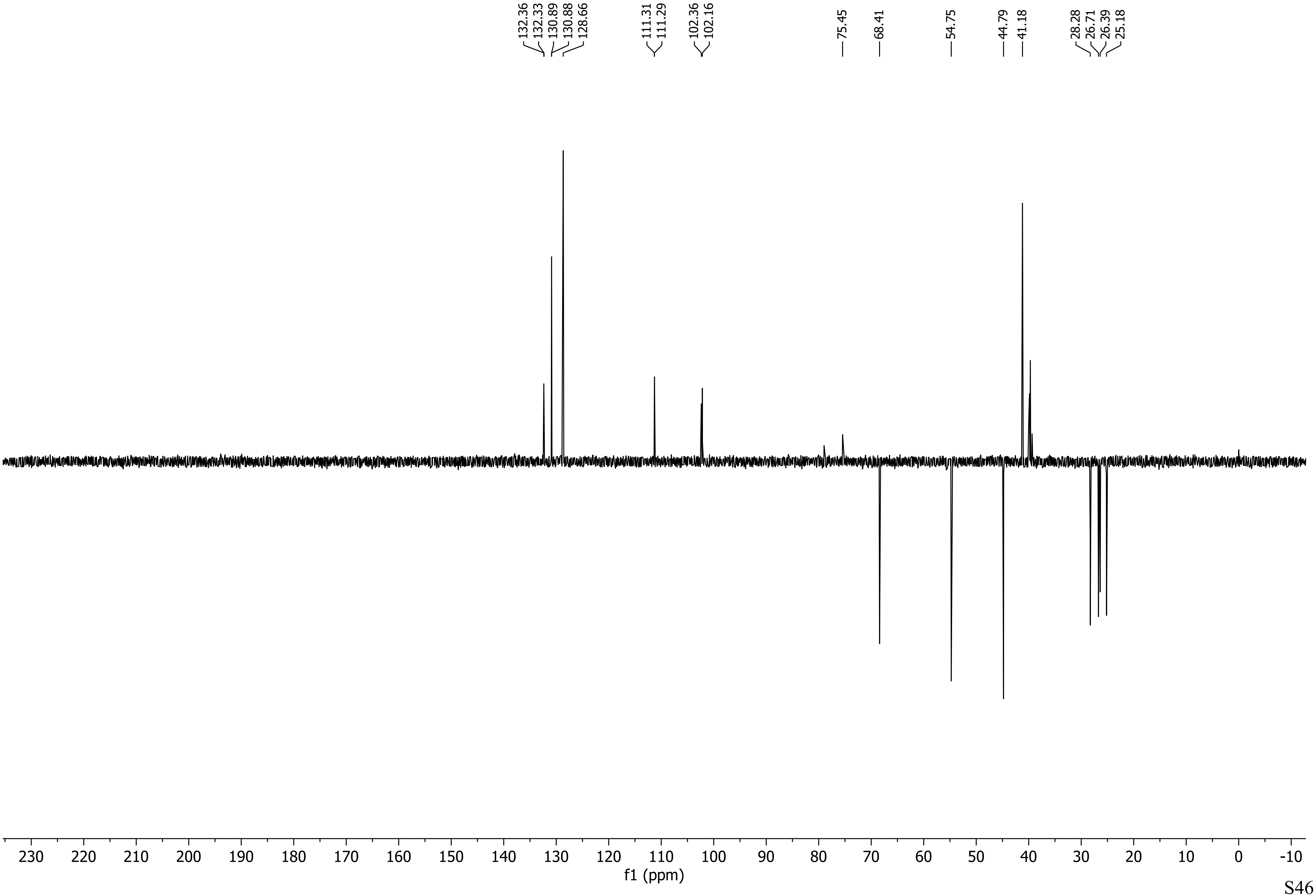

^{1}H NMR (500 MHz, DMSO-d_6) for compound **5** (zoomed-in view)

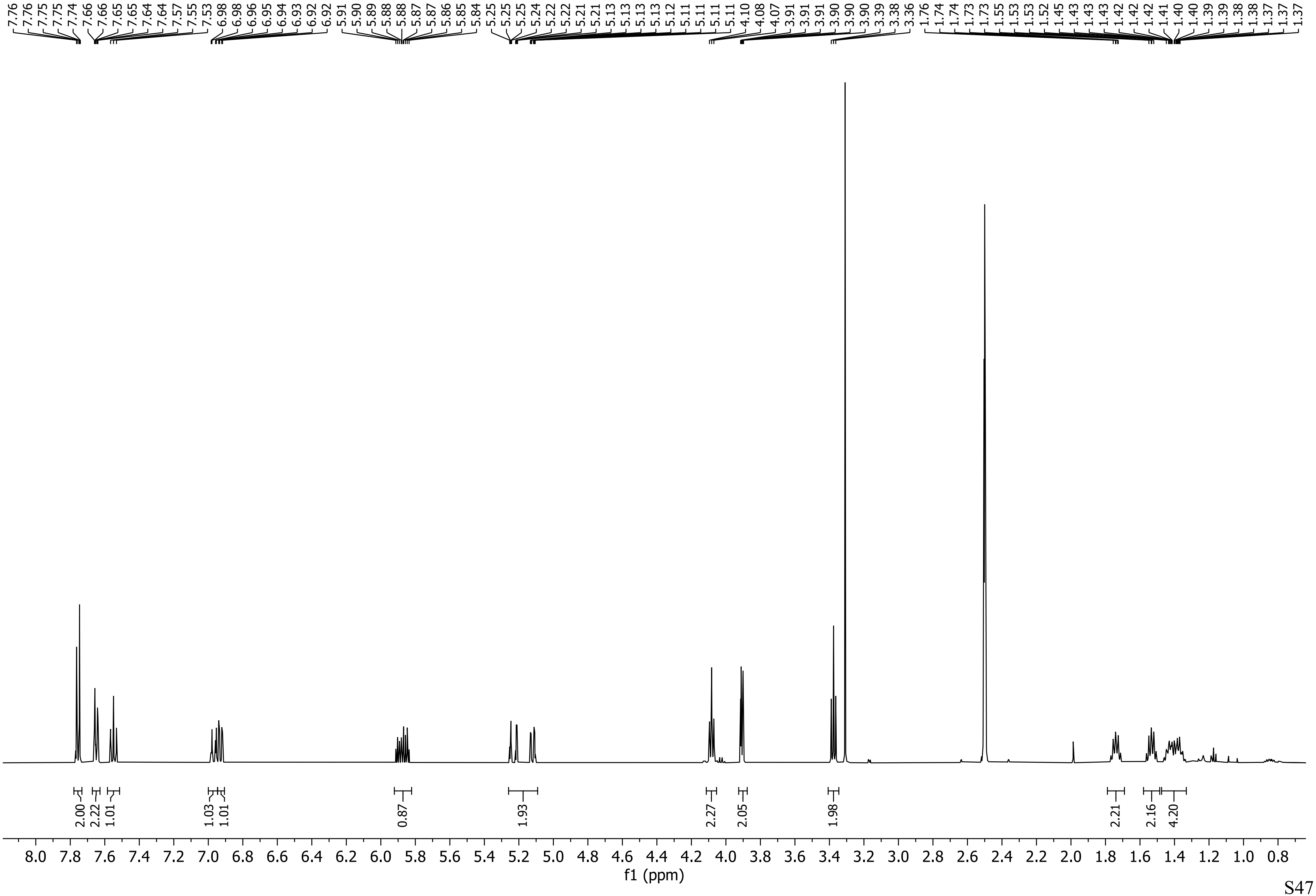

^{13}C NMR (126 MHz, DMSO-d_6) for compound **5**

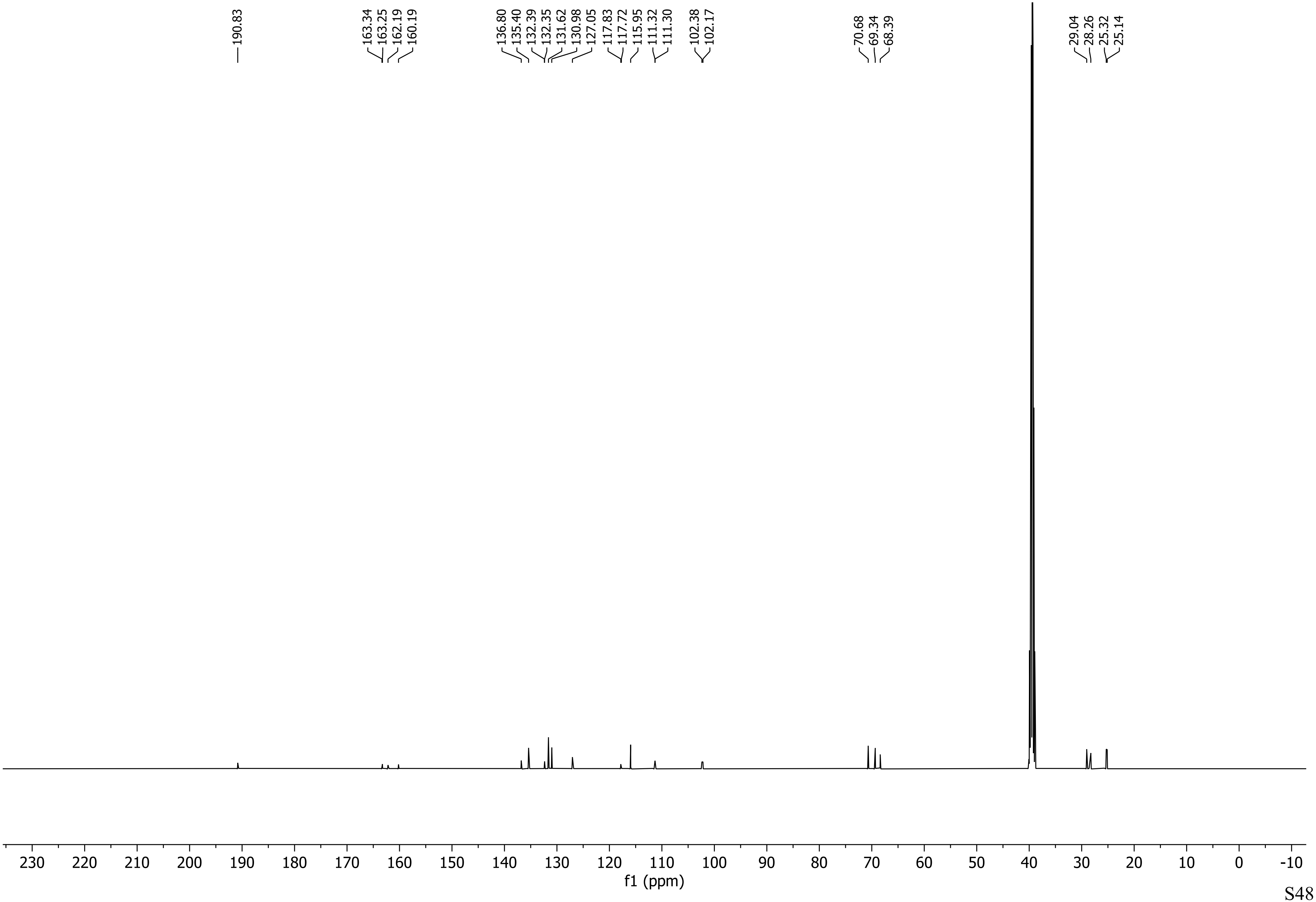

^{13}C NMR (126 MHz, DMSO-d_6) for compound **5** (zoomed-in view)

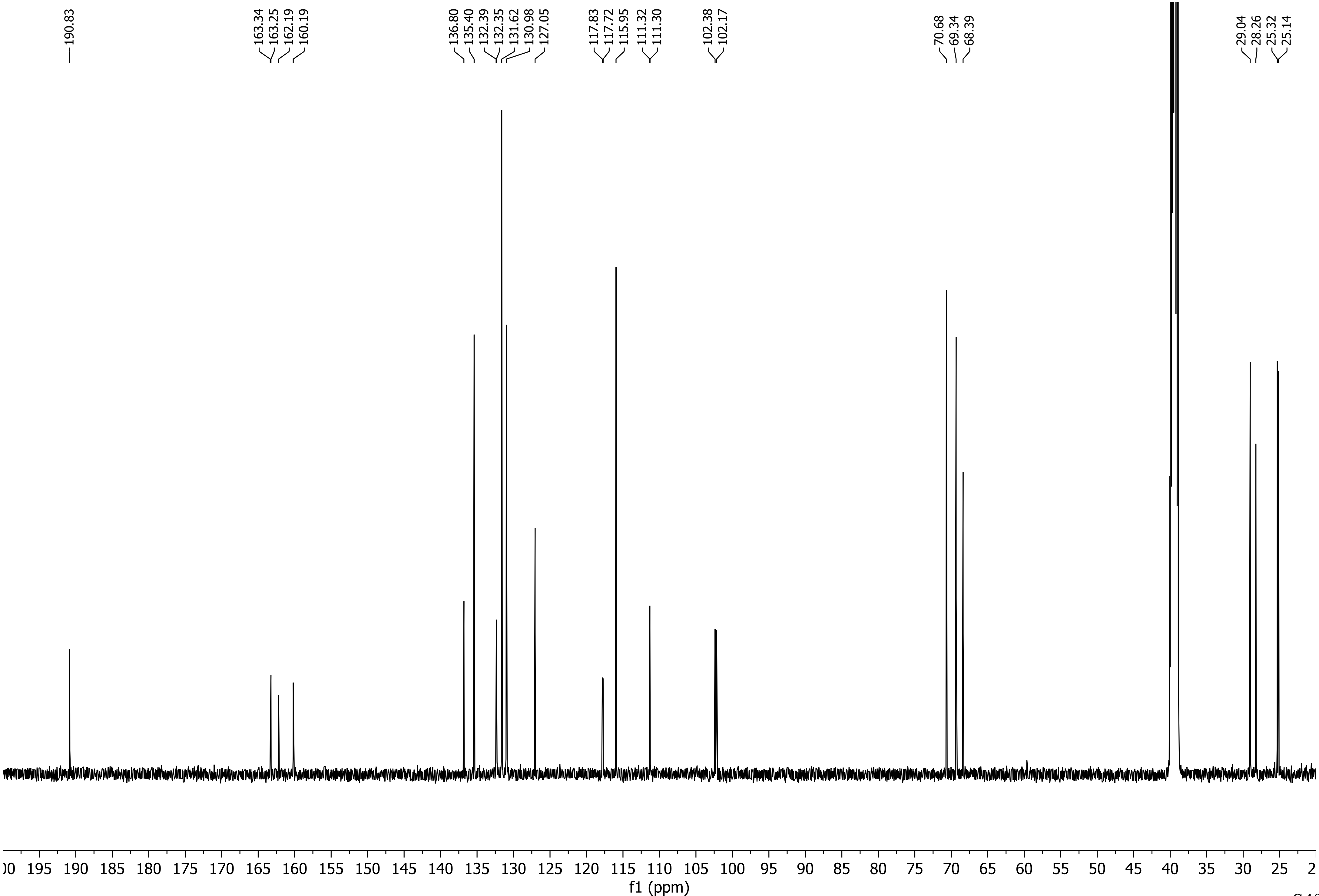

DEPT ^{13}C NMR (126 MHz, DMSO-d_6) for compound **5** (zoomed-in view)

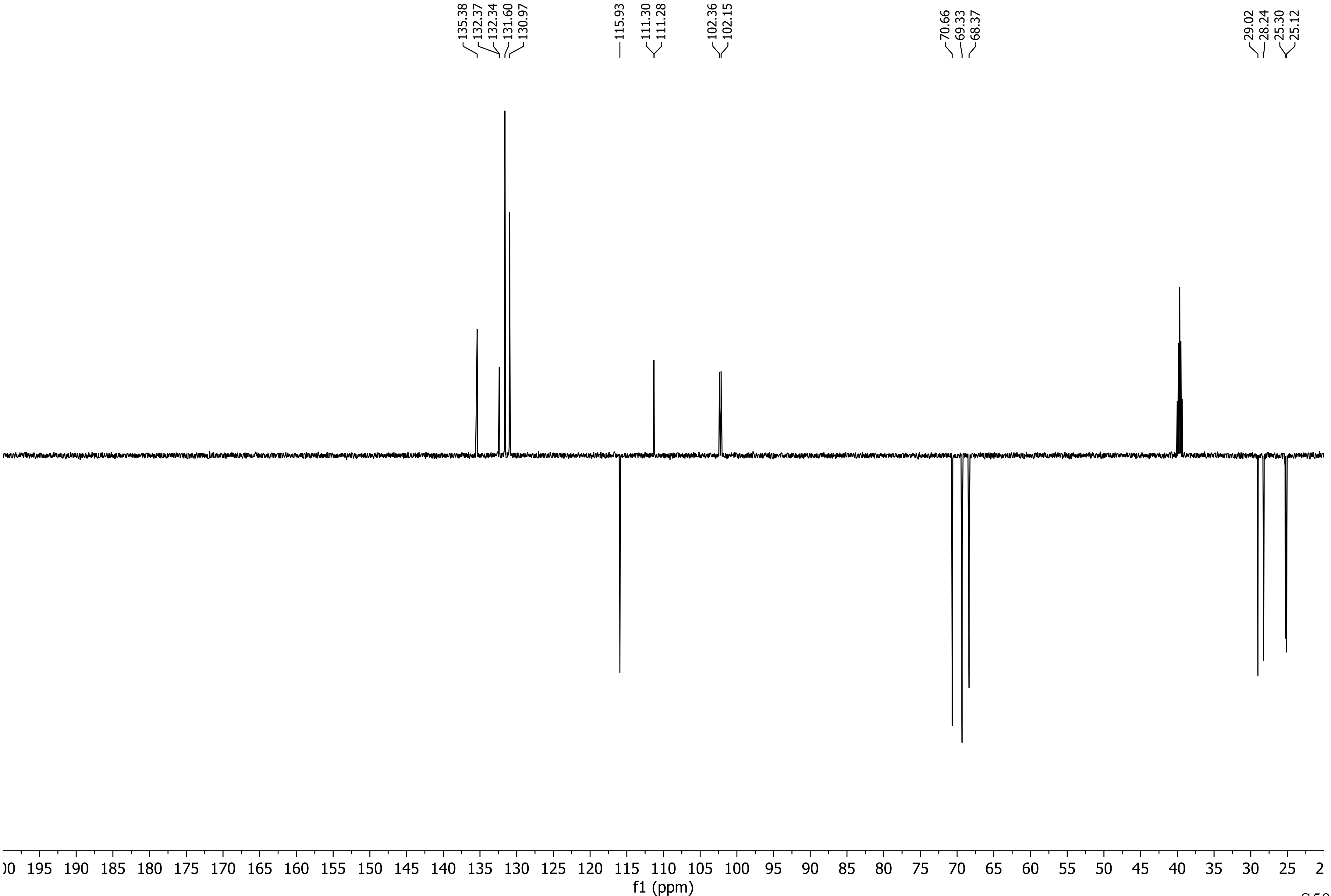

¹H NMR (500 MHz, DMSO-d_6) for compound **6a** (zoomed-in view)

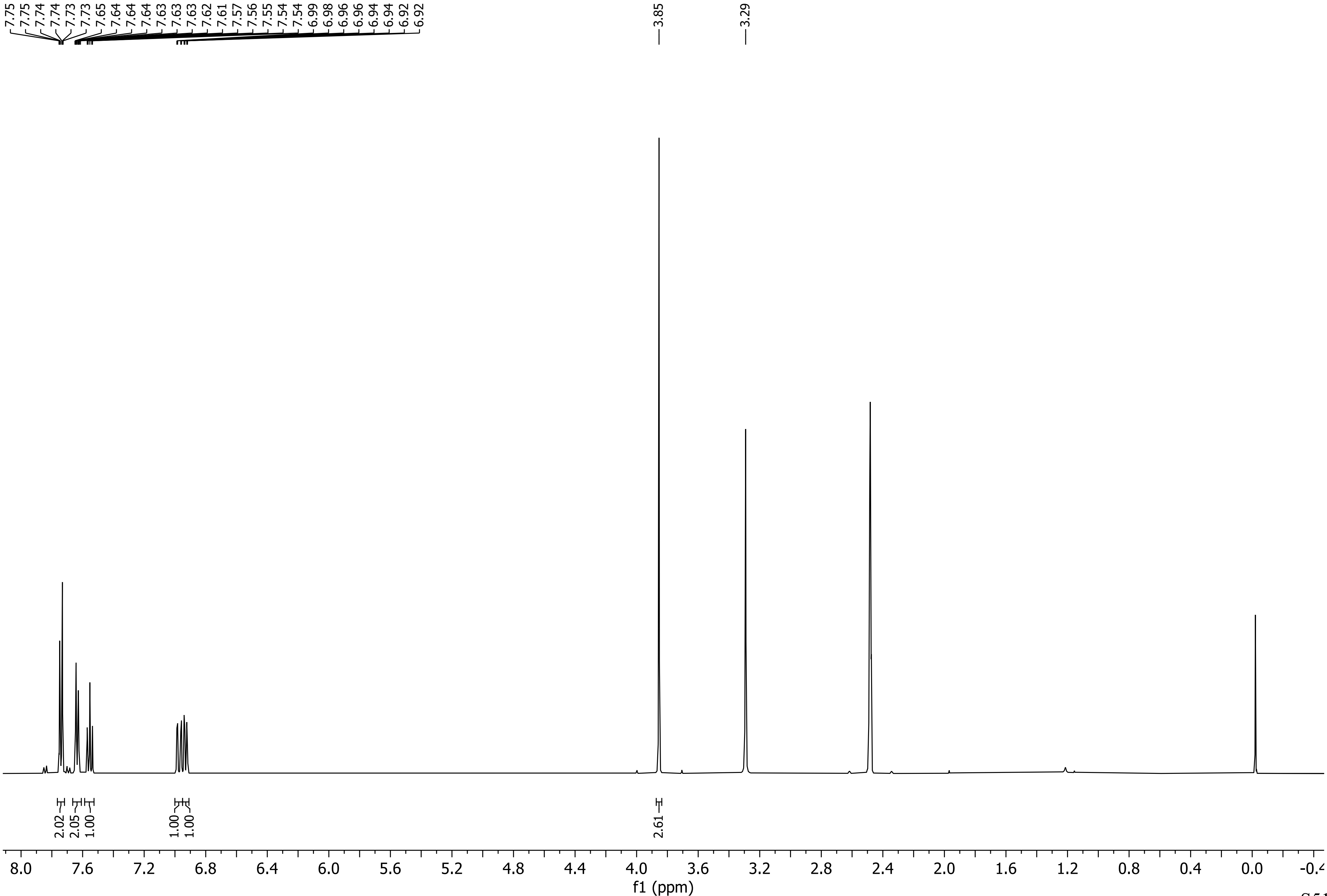

^{13}C NMR (126 MHz, DMSO-d_6) for compound **6a**

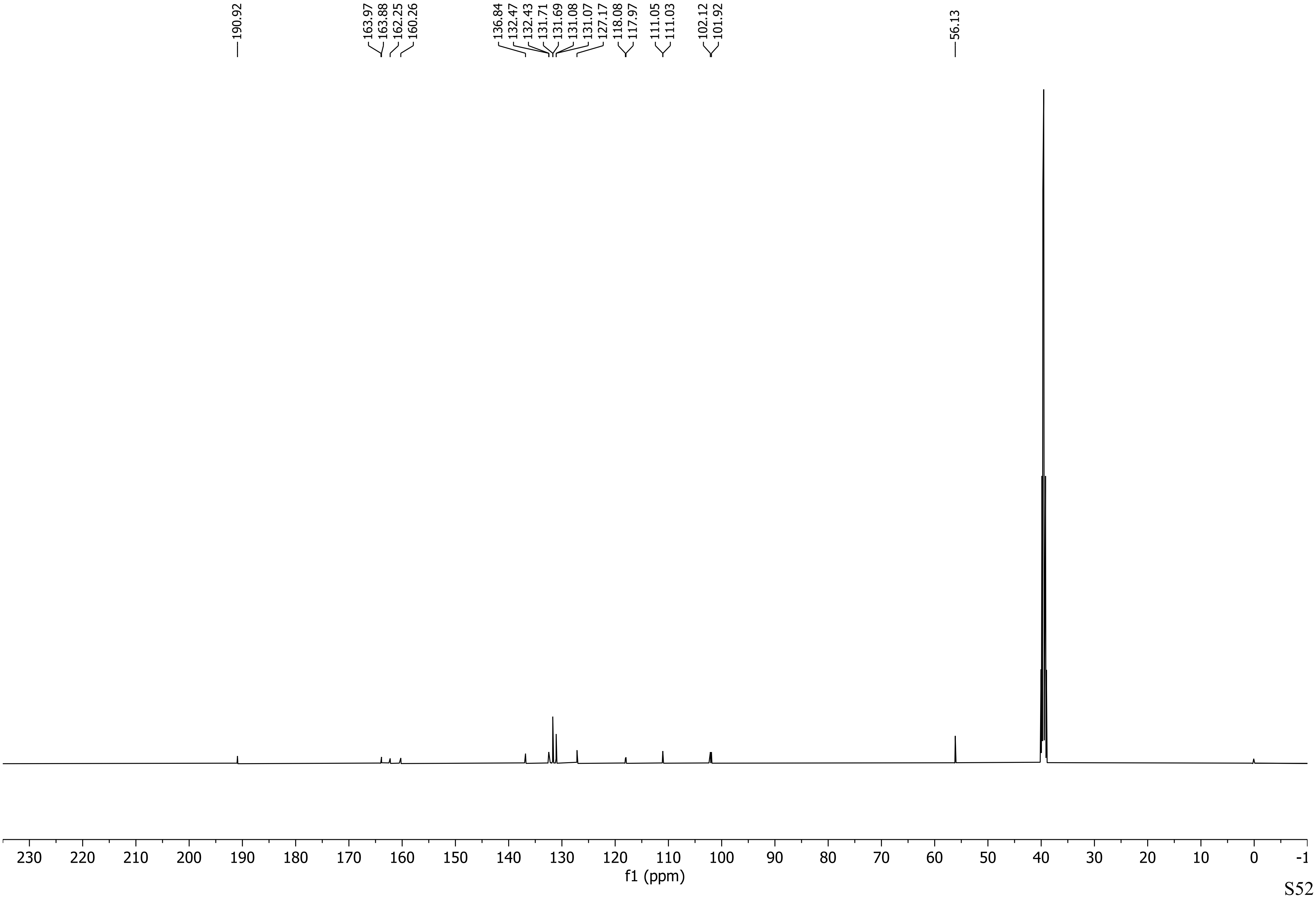

^{13}C NMR (126 MHz, DMSO-d_6) for compound **6a** (zoomed-in view)

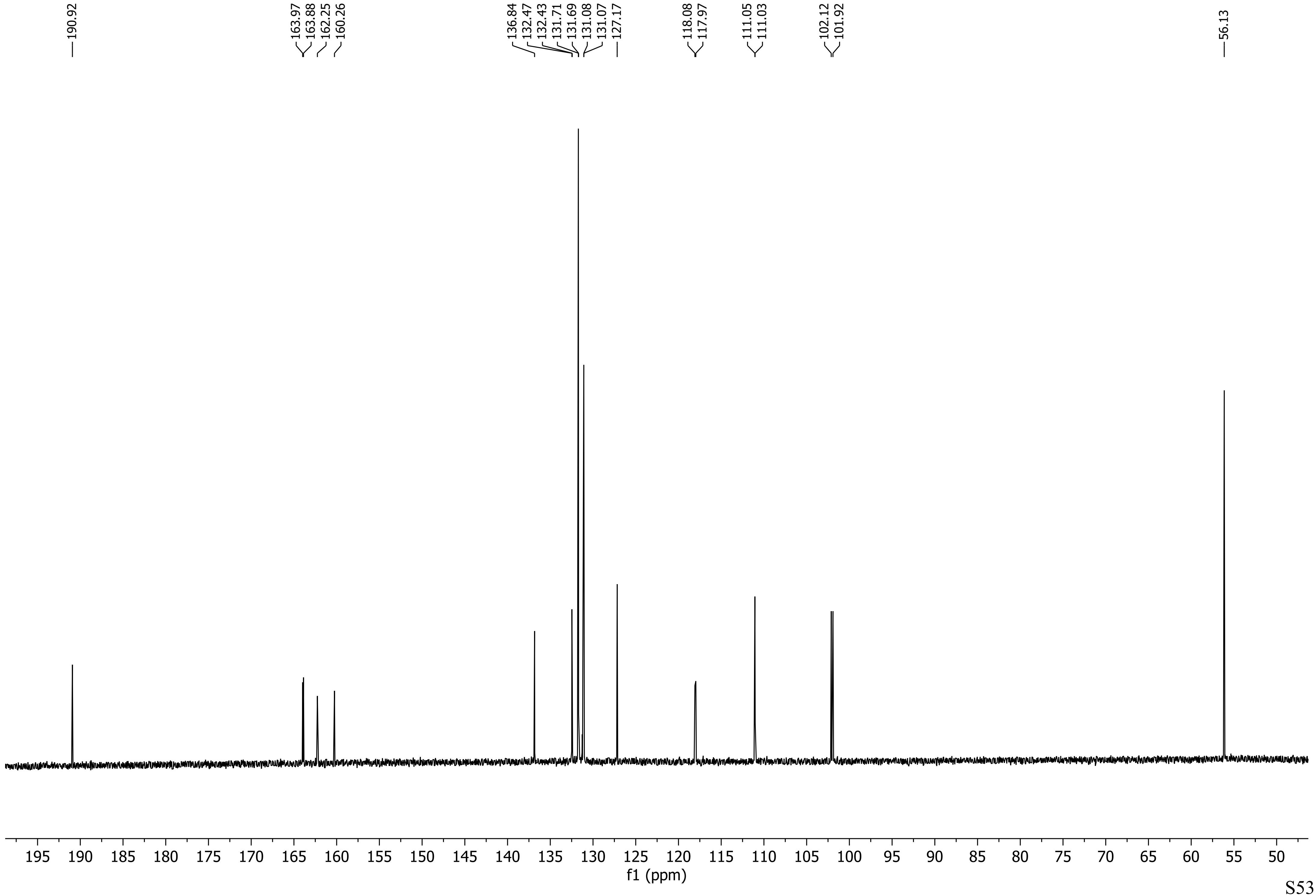

DEPT ^{13}C NMR (126 MHz, DMSO-d_6) for compound **6a**

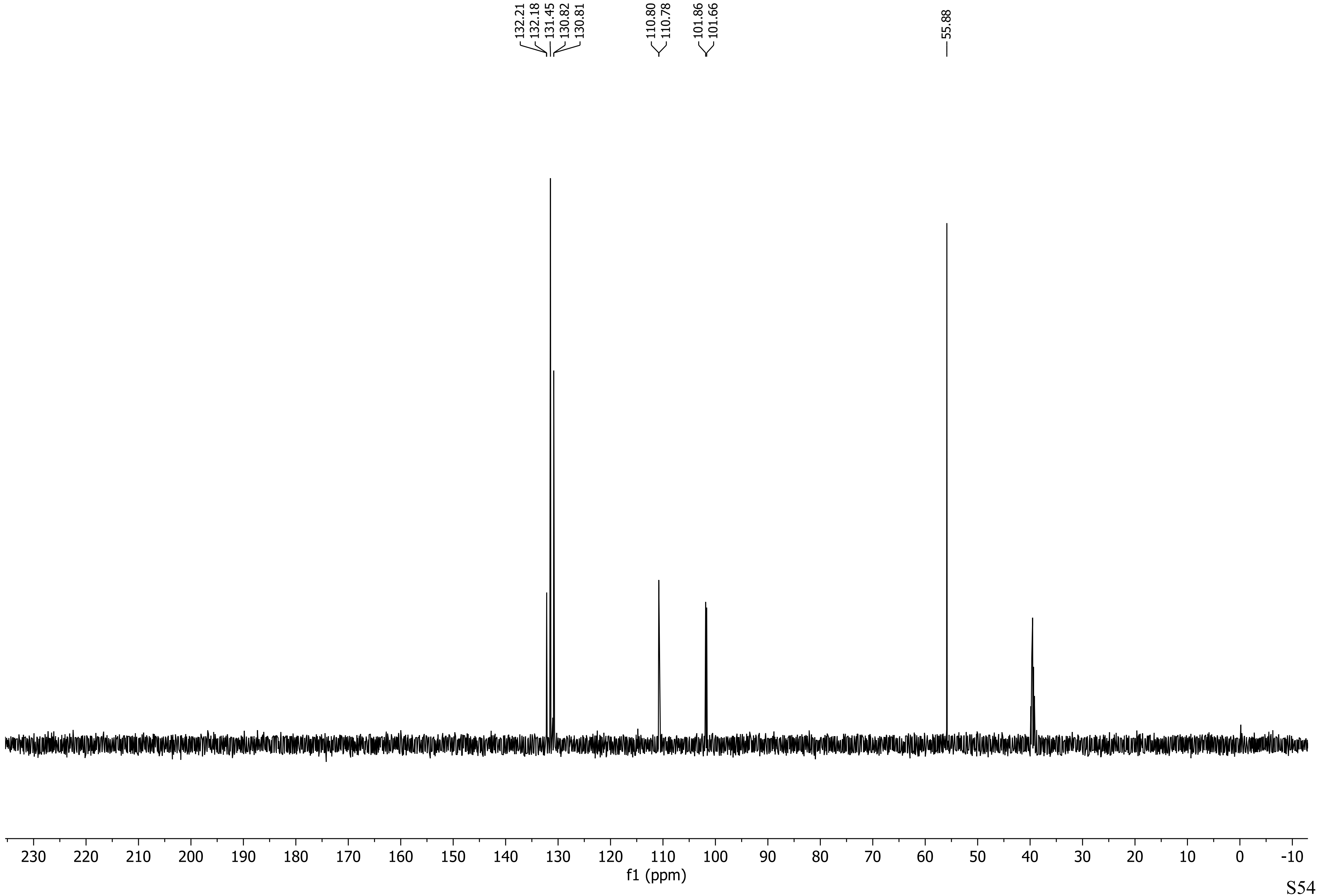

^{1}H NMR (500 MHz, DMSO-d_6) for compound **6b** (zoomed-in view)

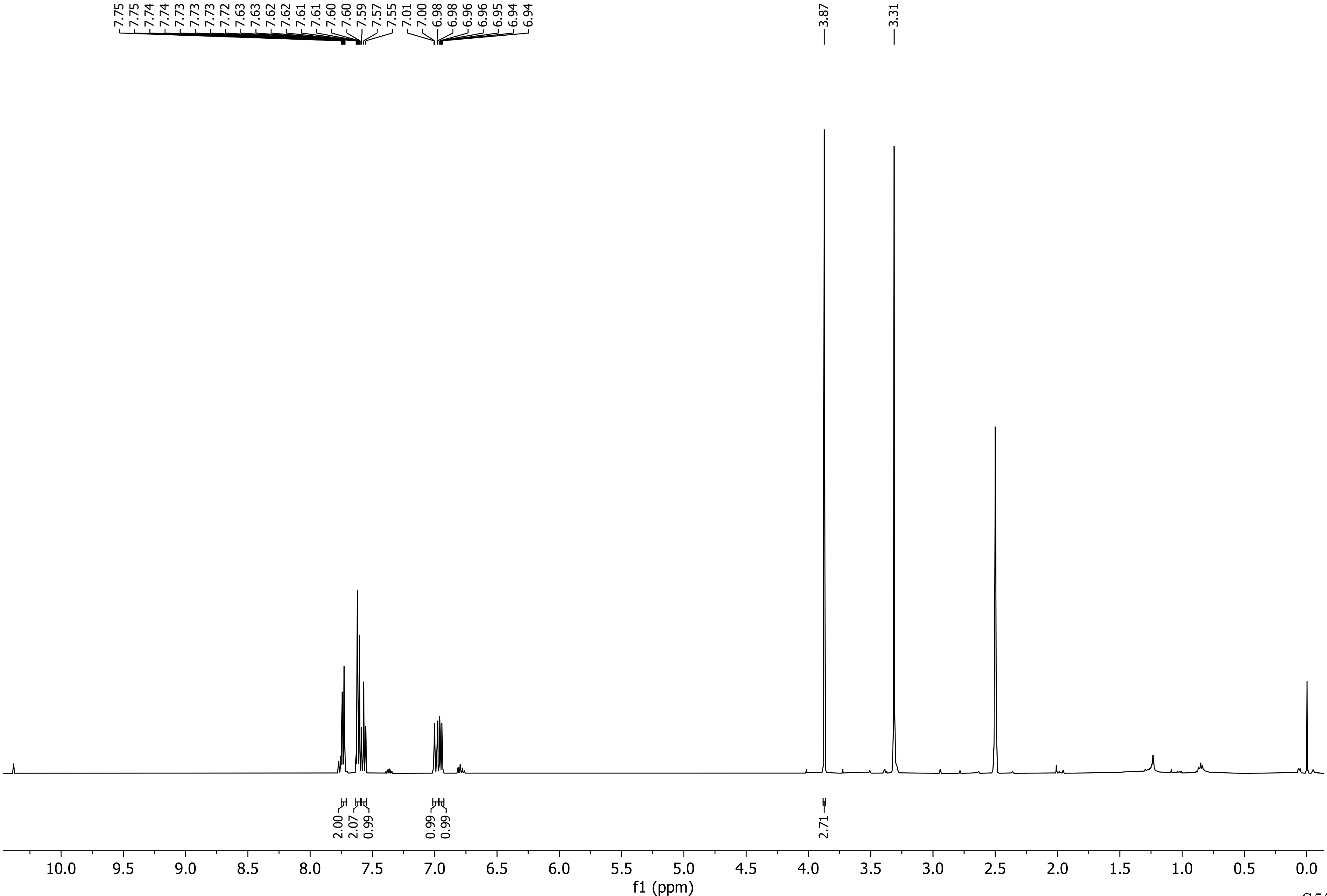

^{13}CNMR (126 MHz, DMSO-d_6) for compound **6b**

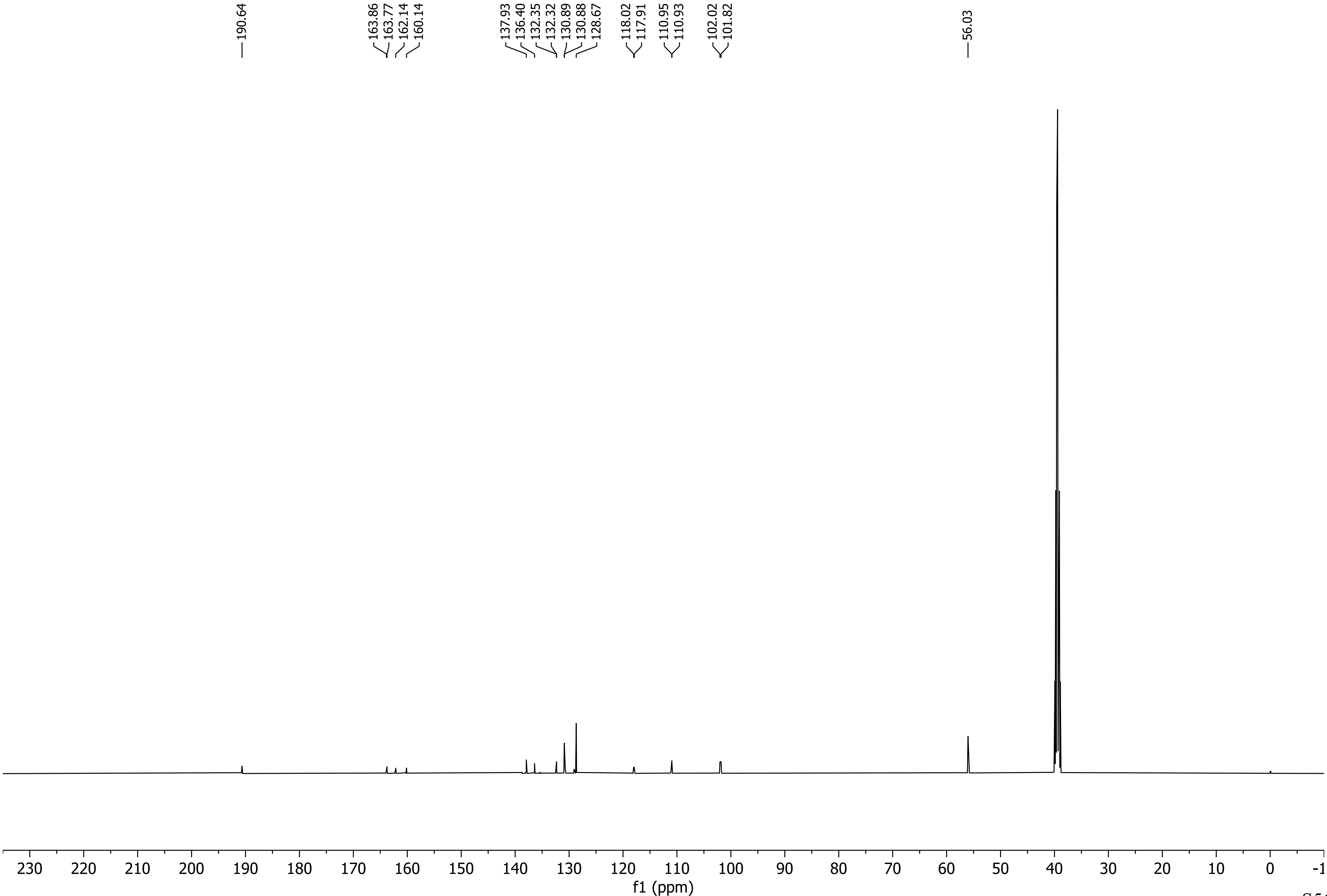

^{13}CNMR (126 MHz, DMSO-d_6) for compound **6b** (zoomed-in view)

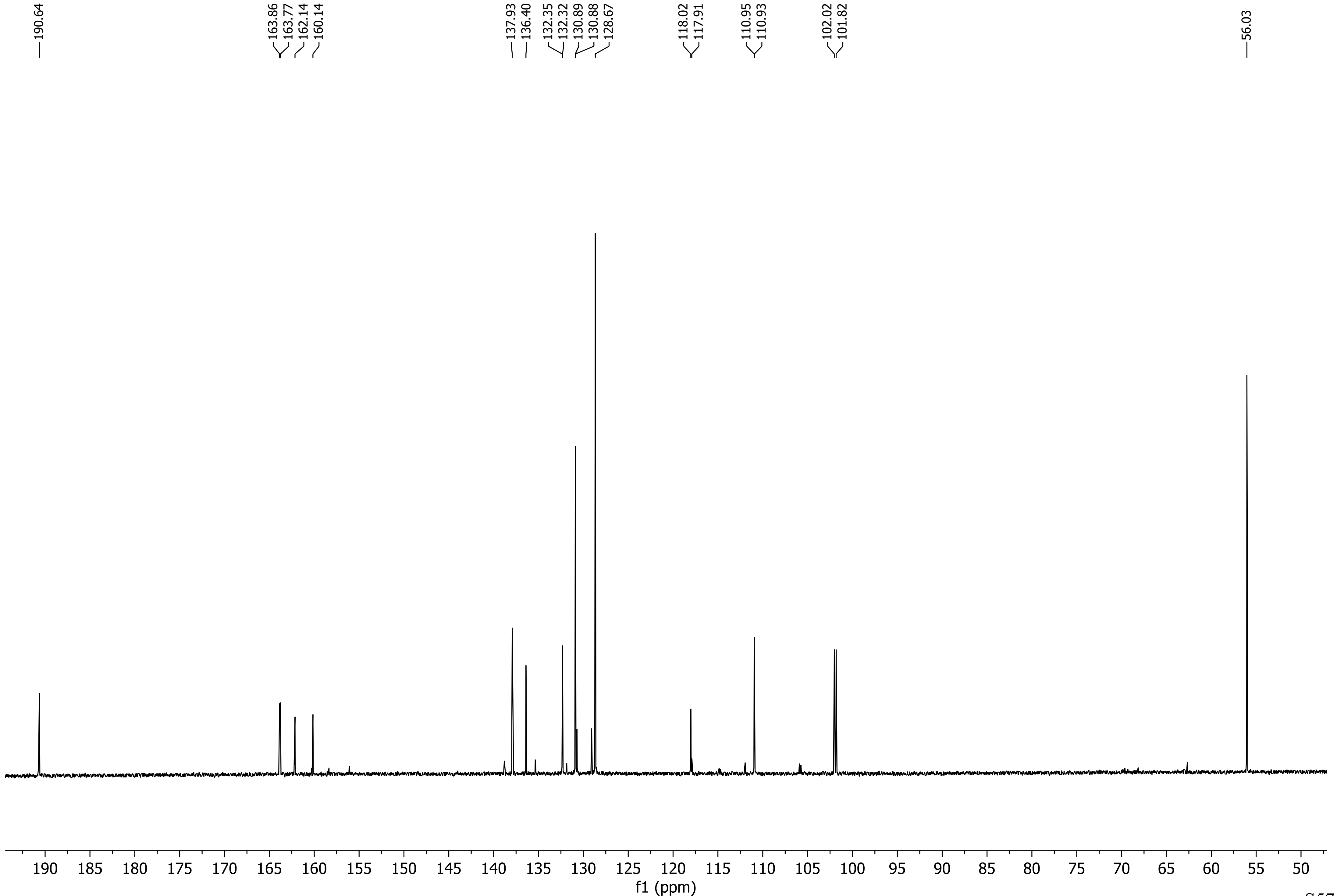

DEPT ^{13}C NMR (126 MHz, DMSO-d_6) for compound **6b**

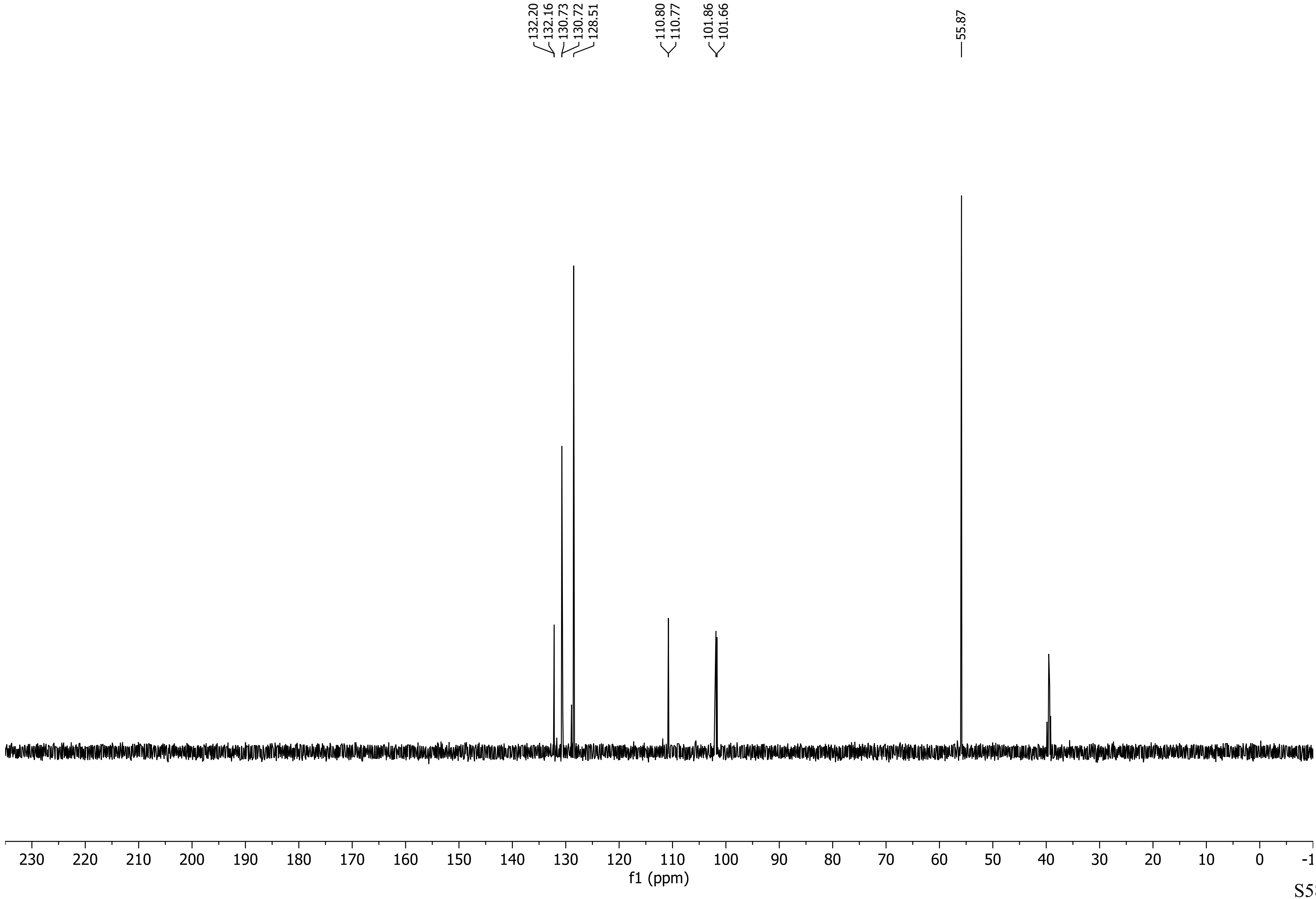

^{1}H NMR (500 MHz, DMSO-d_6) for compound **7a** (zoomed-in view)

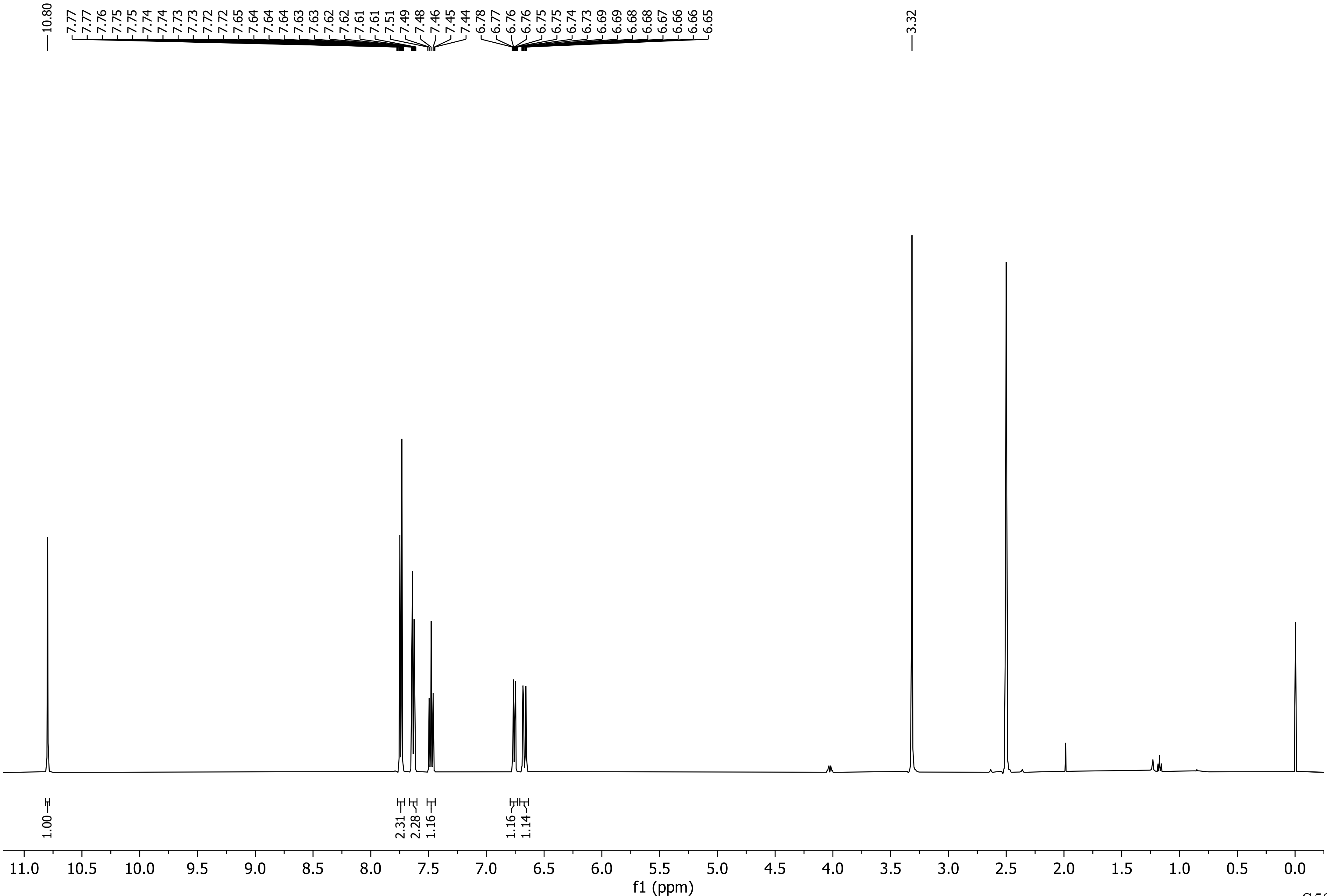

^{13}C NMR (126 MHz, DMSO-d_6) for compound **7a**

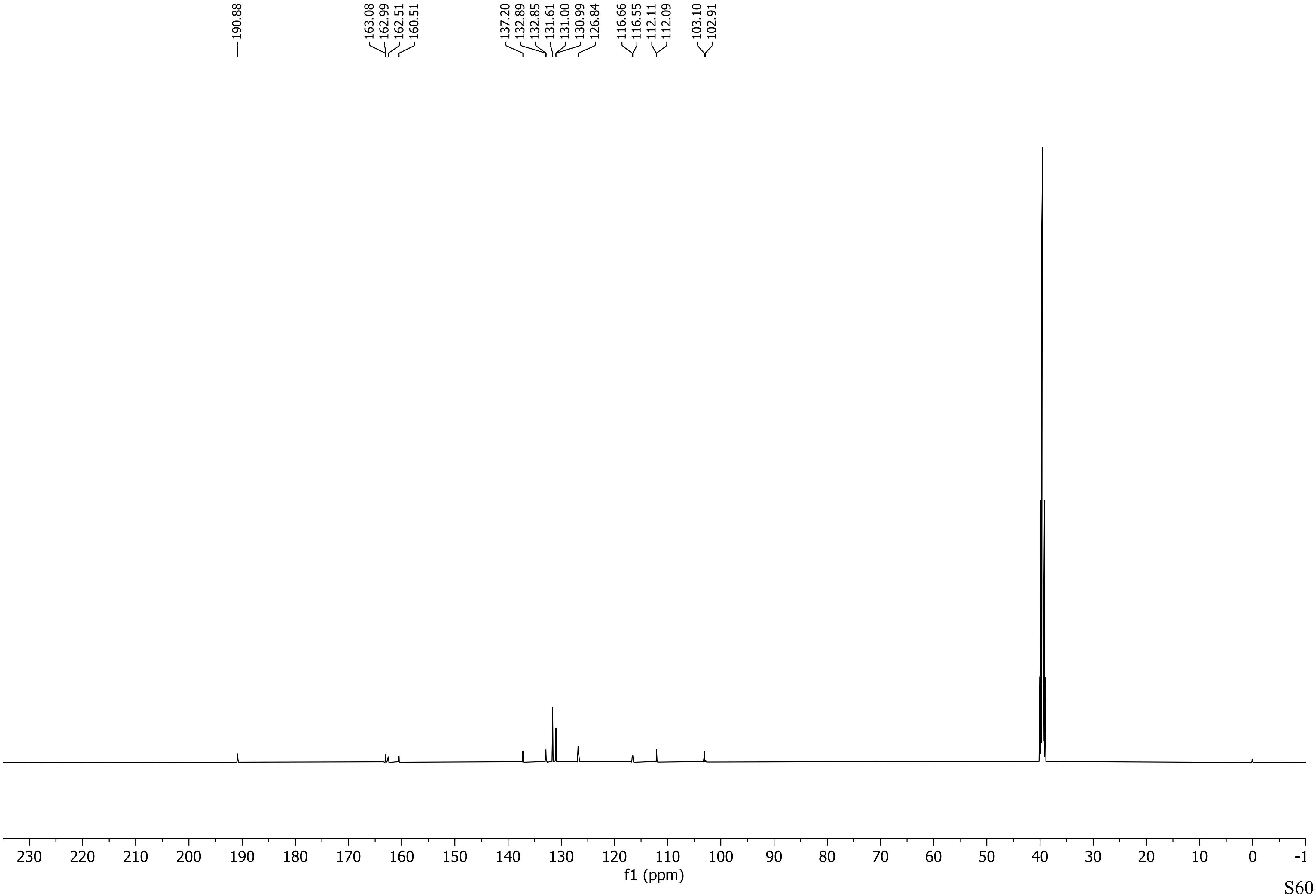

^{13}C NMR (126 MHz, DMSO-d_6) for compound **7a** (zoomed-in view)

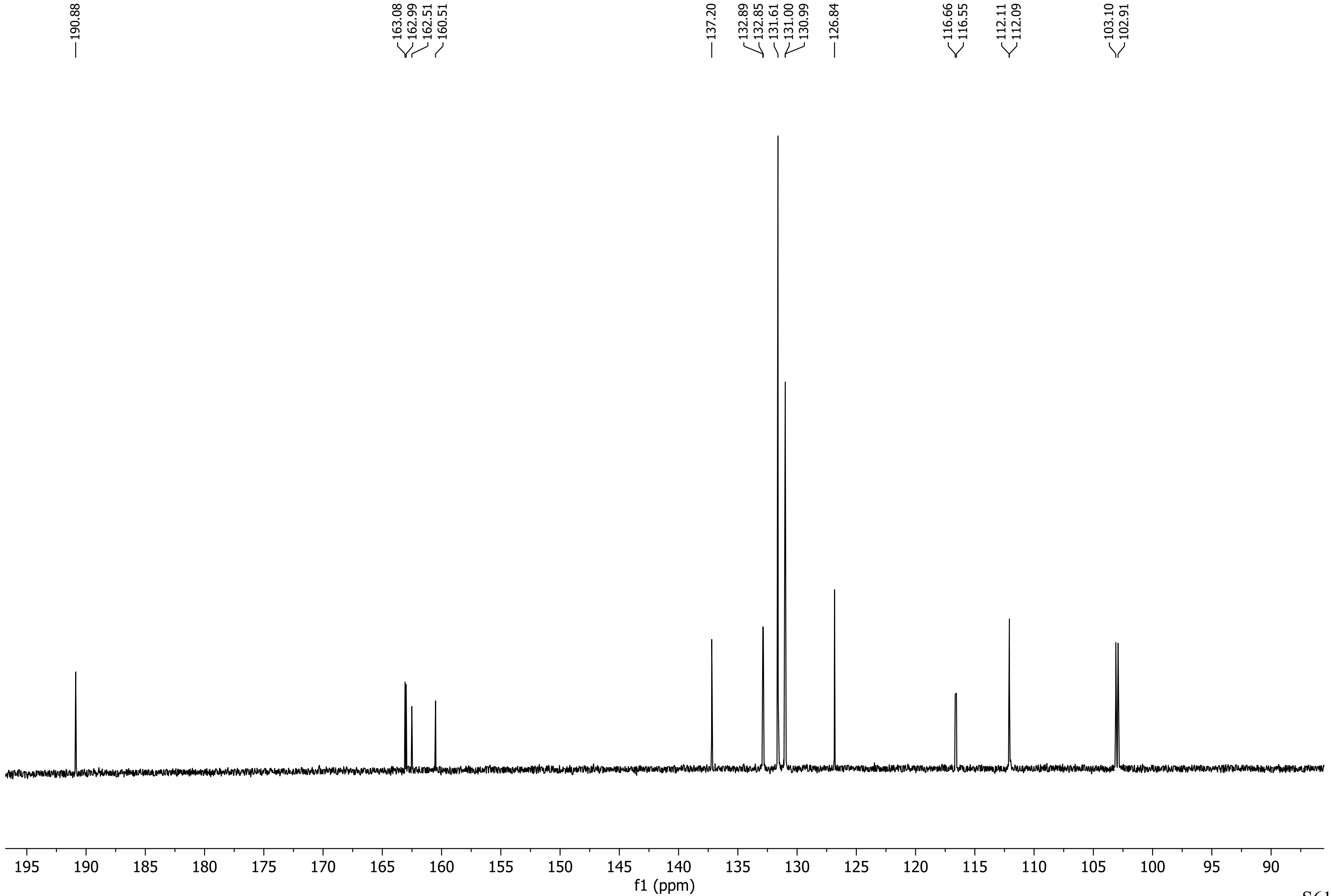

DEPT ^{13}C NMR (126 MHz, DMSO-d_6) for compound **7a**

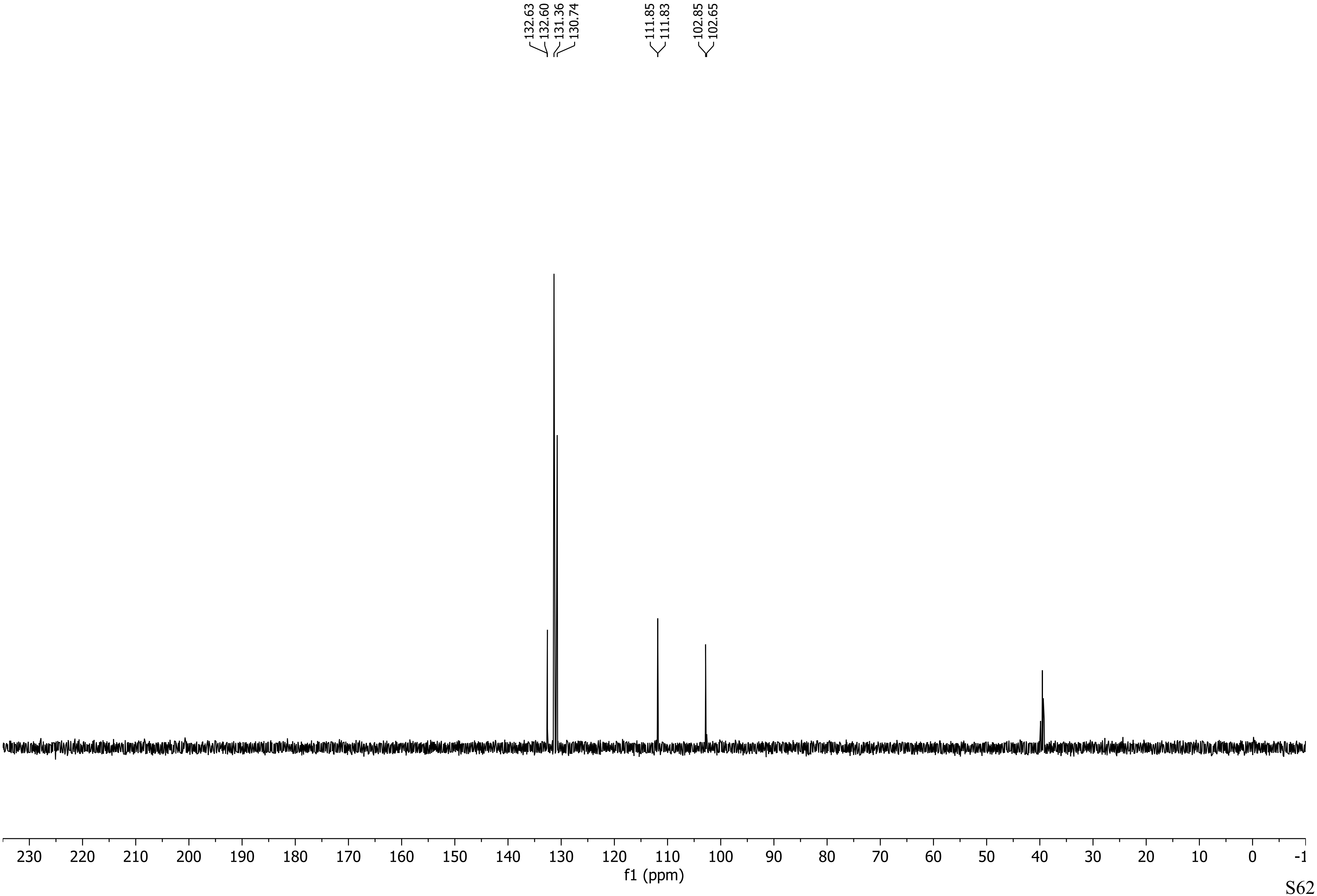

^{1}H NMR (500 MHz, DMSO-d_6) for compound **7b** (zoomed-in view)

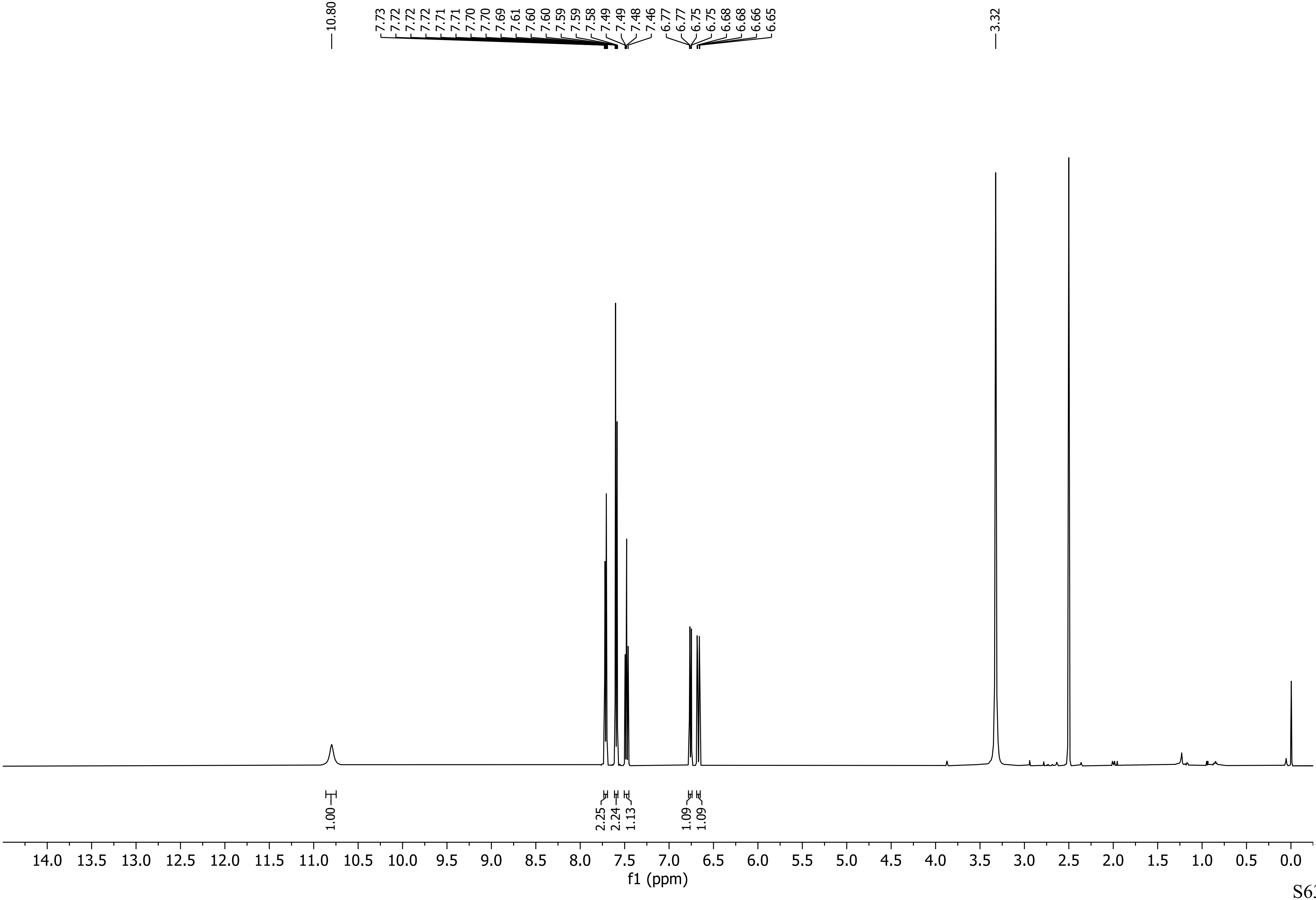

^{13}C NMR (126 MHz, DMSO-d_6) for compound **7b**

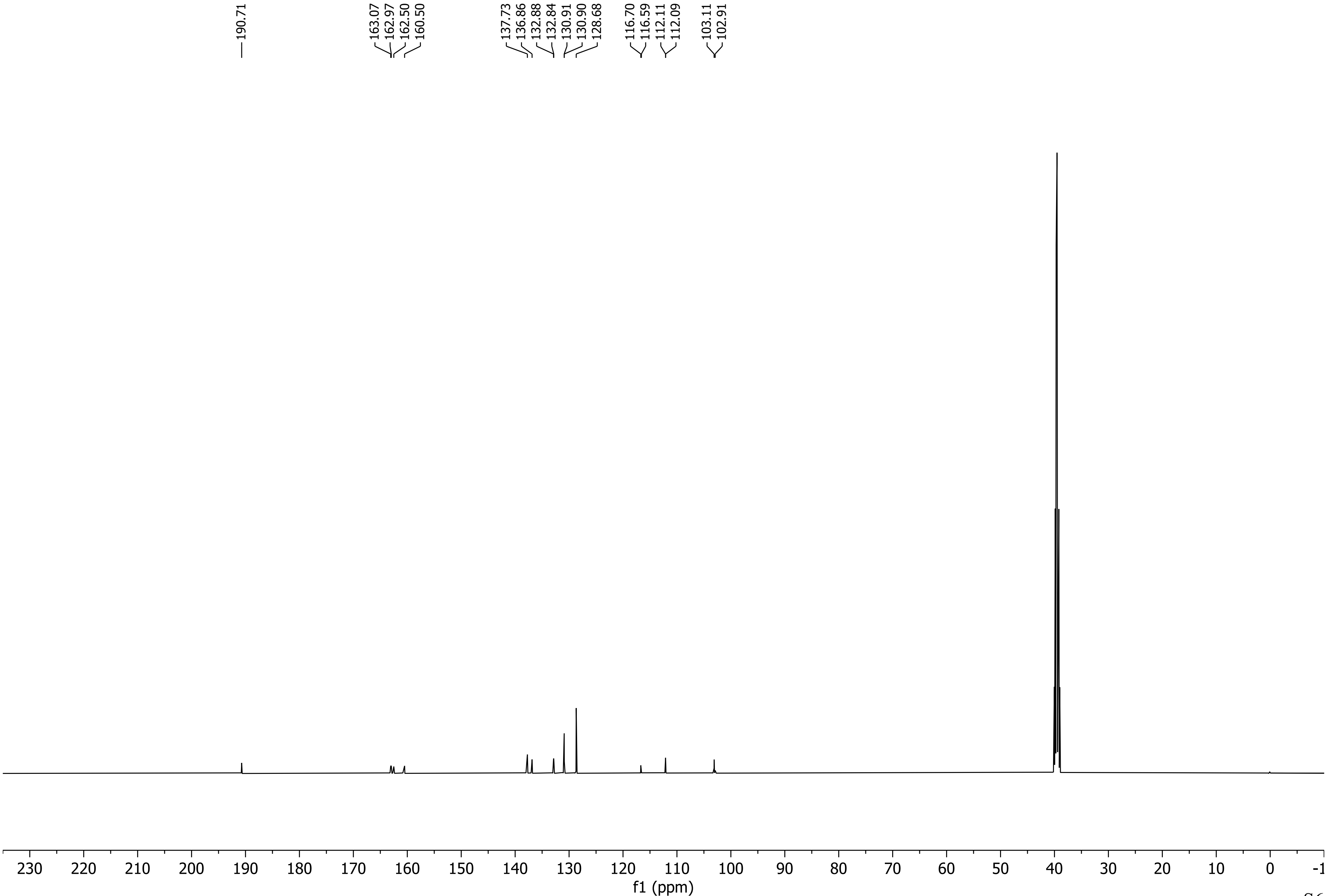

^{13}C NMR (126 MHz, DMSO-d_6) for compound **7b** (zoomed-in view)

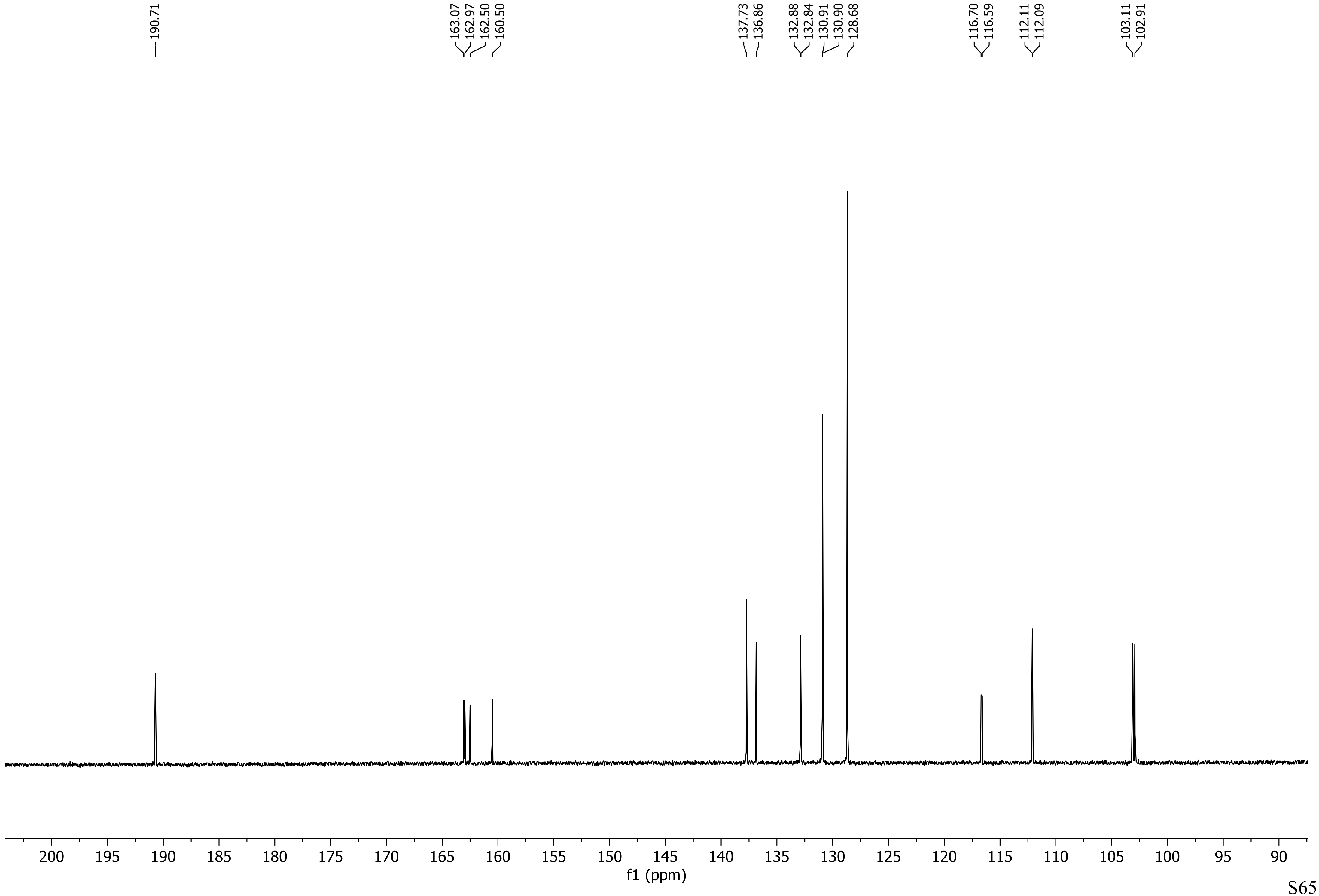

DEPT ^{13}C NMR (126 MHz, DMSO-d_6) for compound **7b**

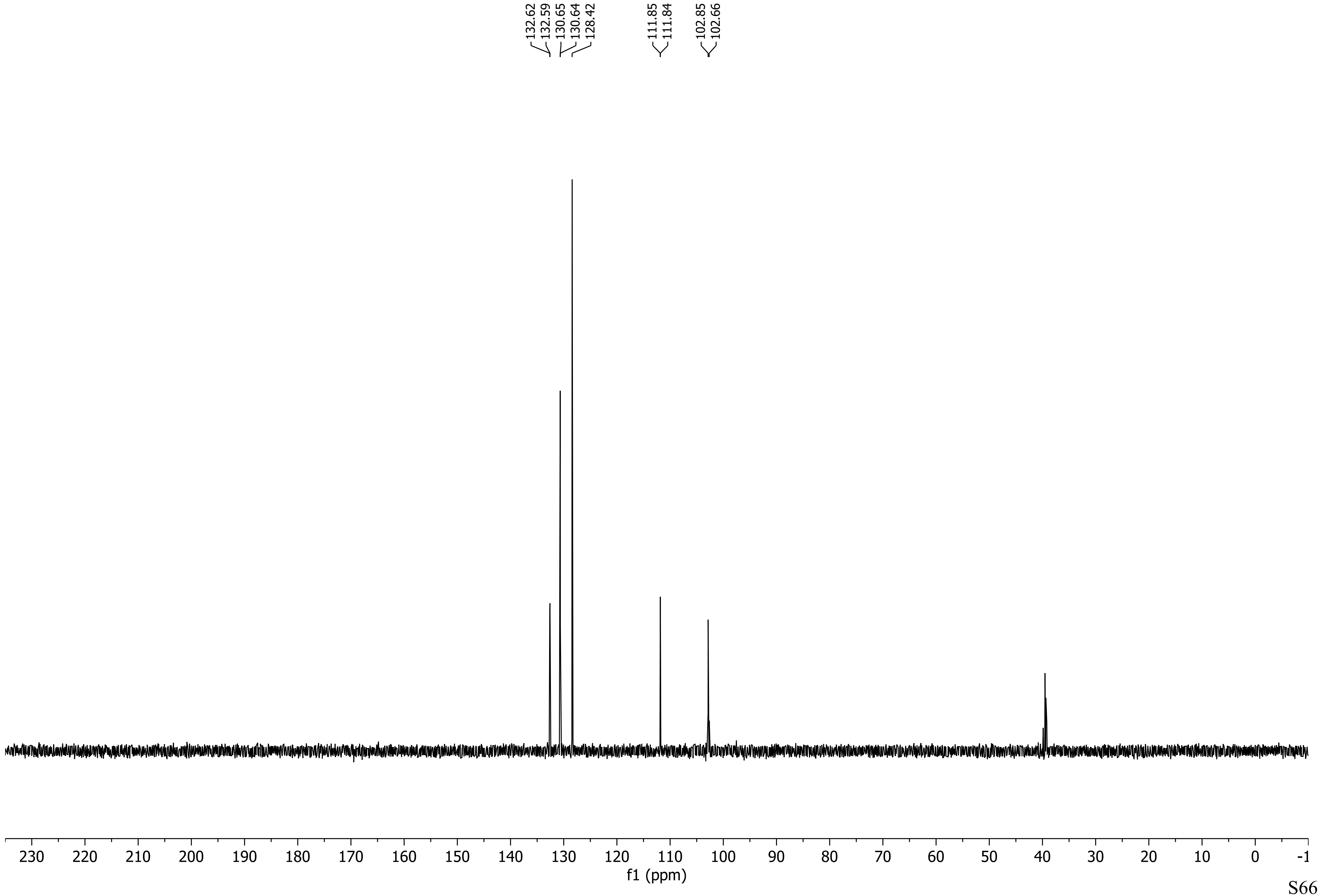

^1H NMR (600 MHz, DMSO-d_6) for compound **7c** (zoomed-in view)

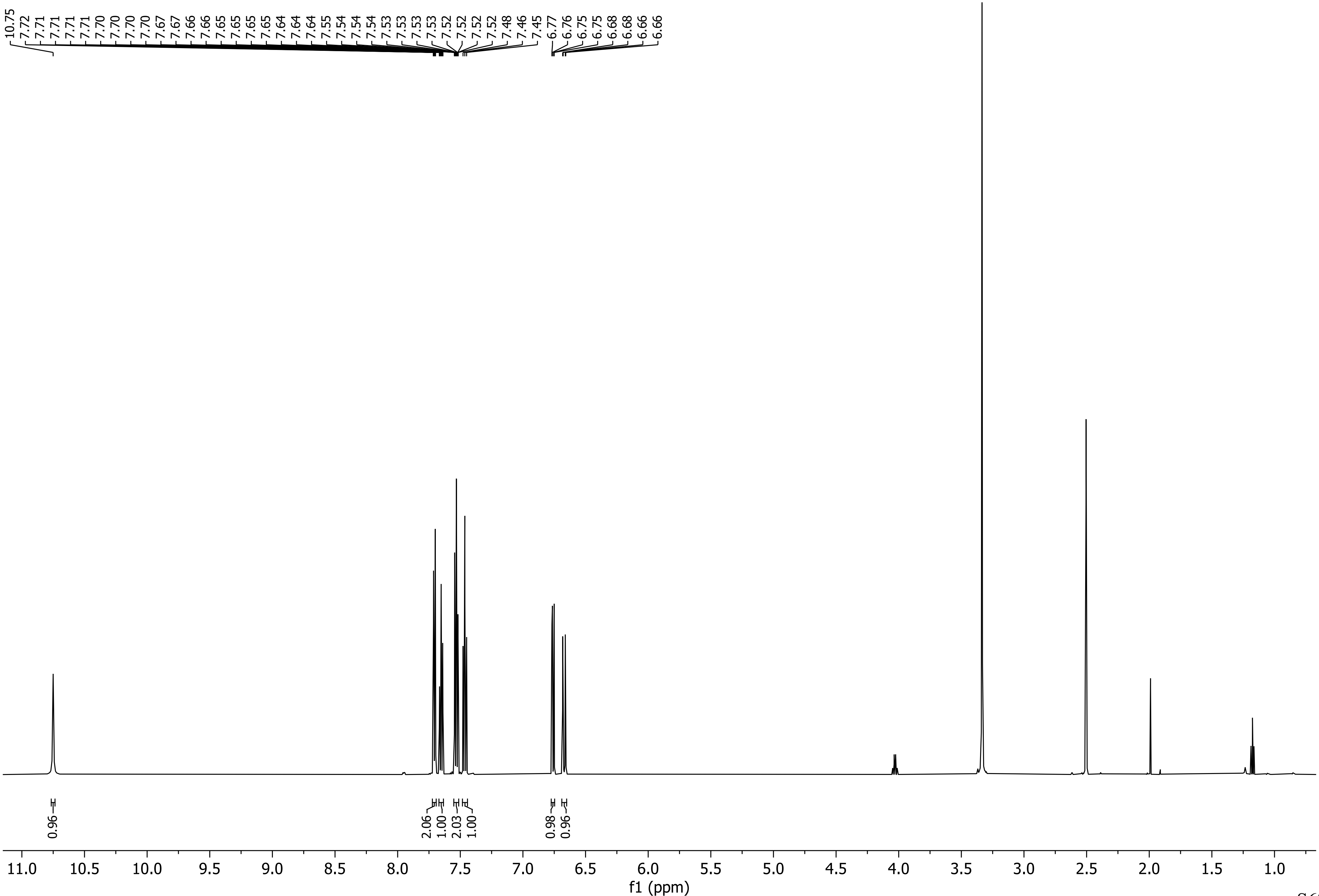

^{13}C NMR (151 MHz, DMSO-d_6) for compound **7c**

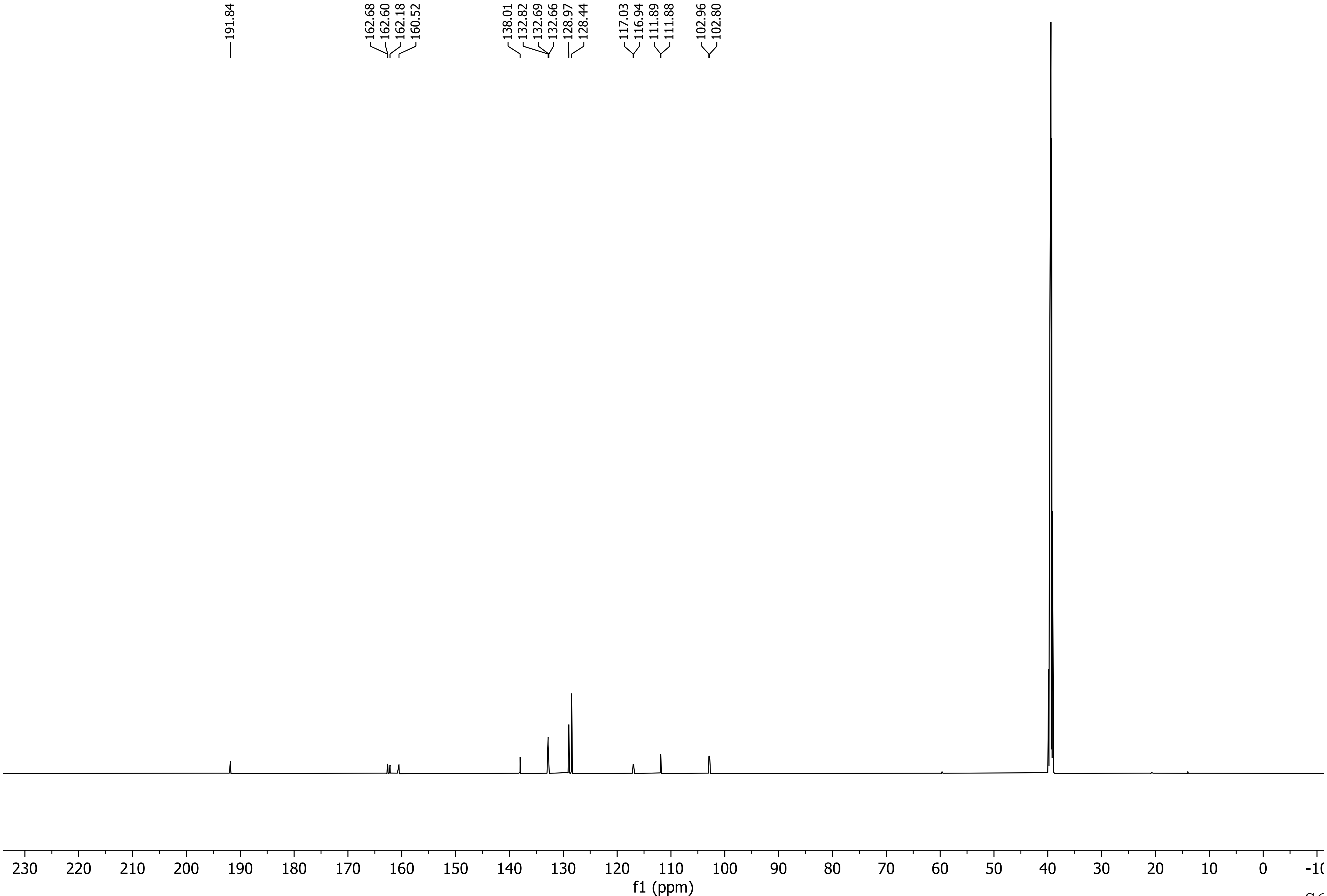

^{13}C NMR (151 MHz, DMSO-d_6) for compound **7c** (zoomed-in view)

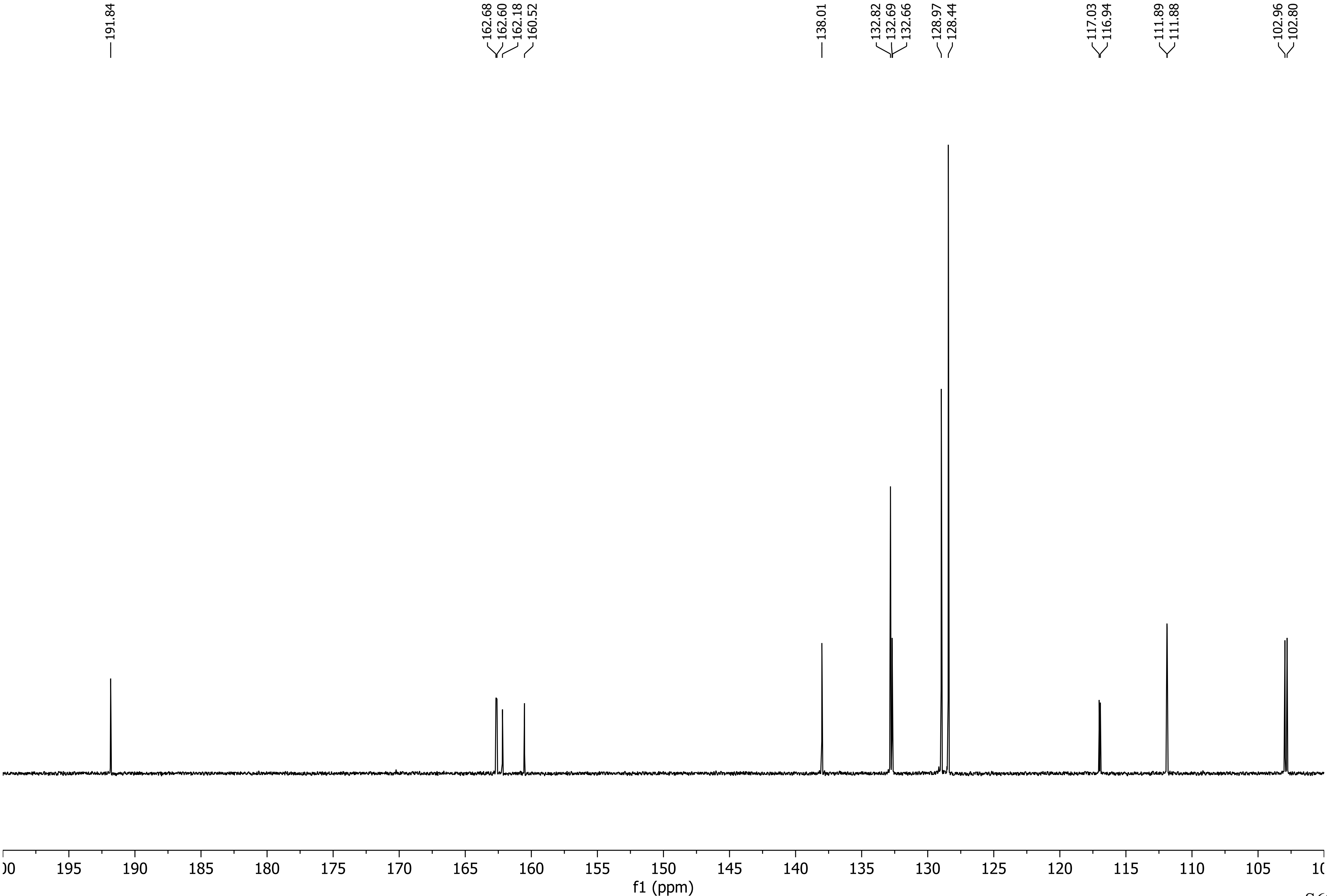

DEPT ^{13}C NMR (151 MHz, DMSO-d_6) for compound **7c** (zoomed-in view)

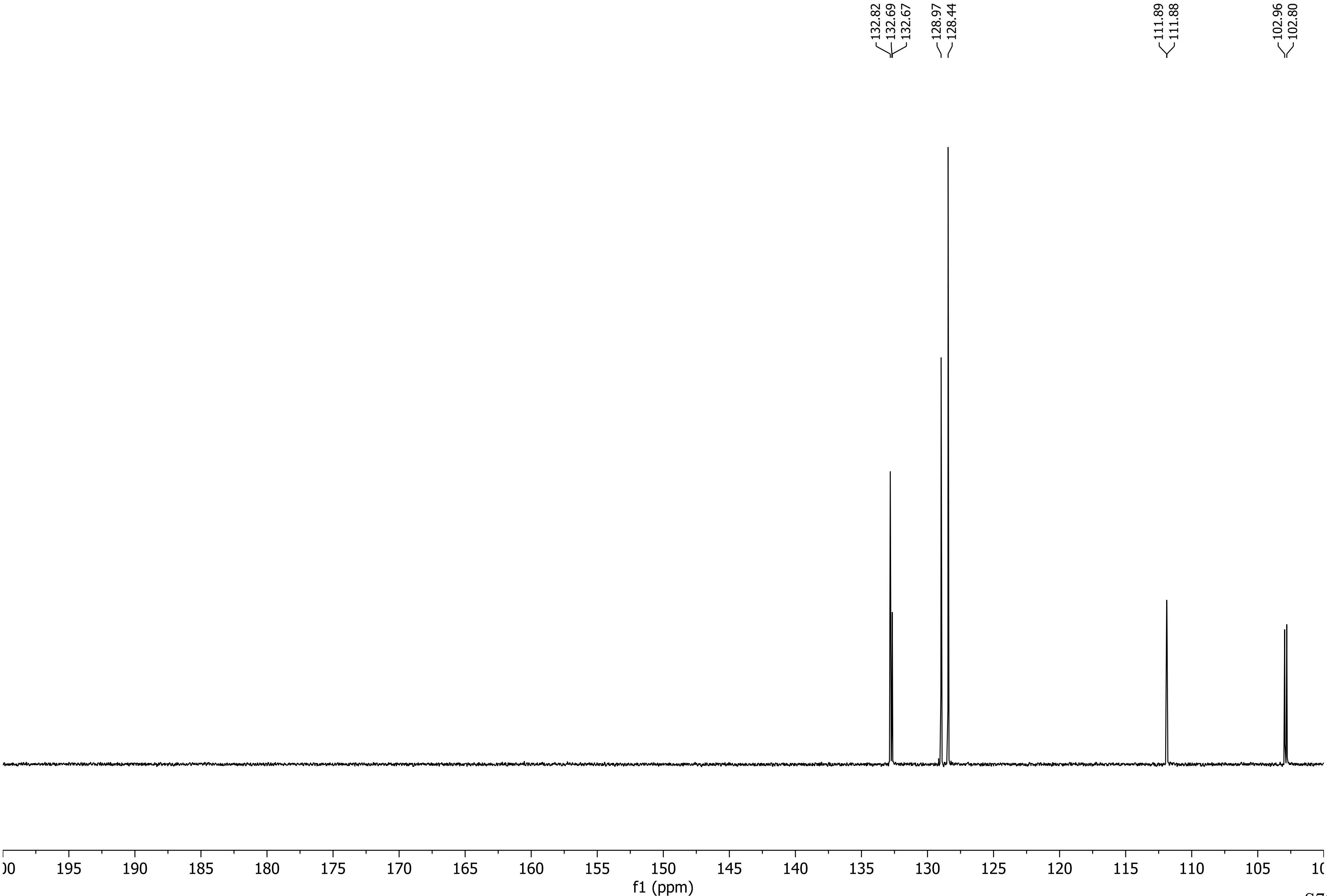

^{1}H NMR (600 MHz, DMSO-d_6) for compound **8a** (zoomed-in view)

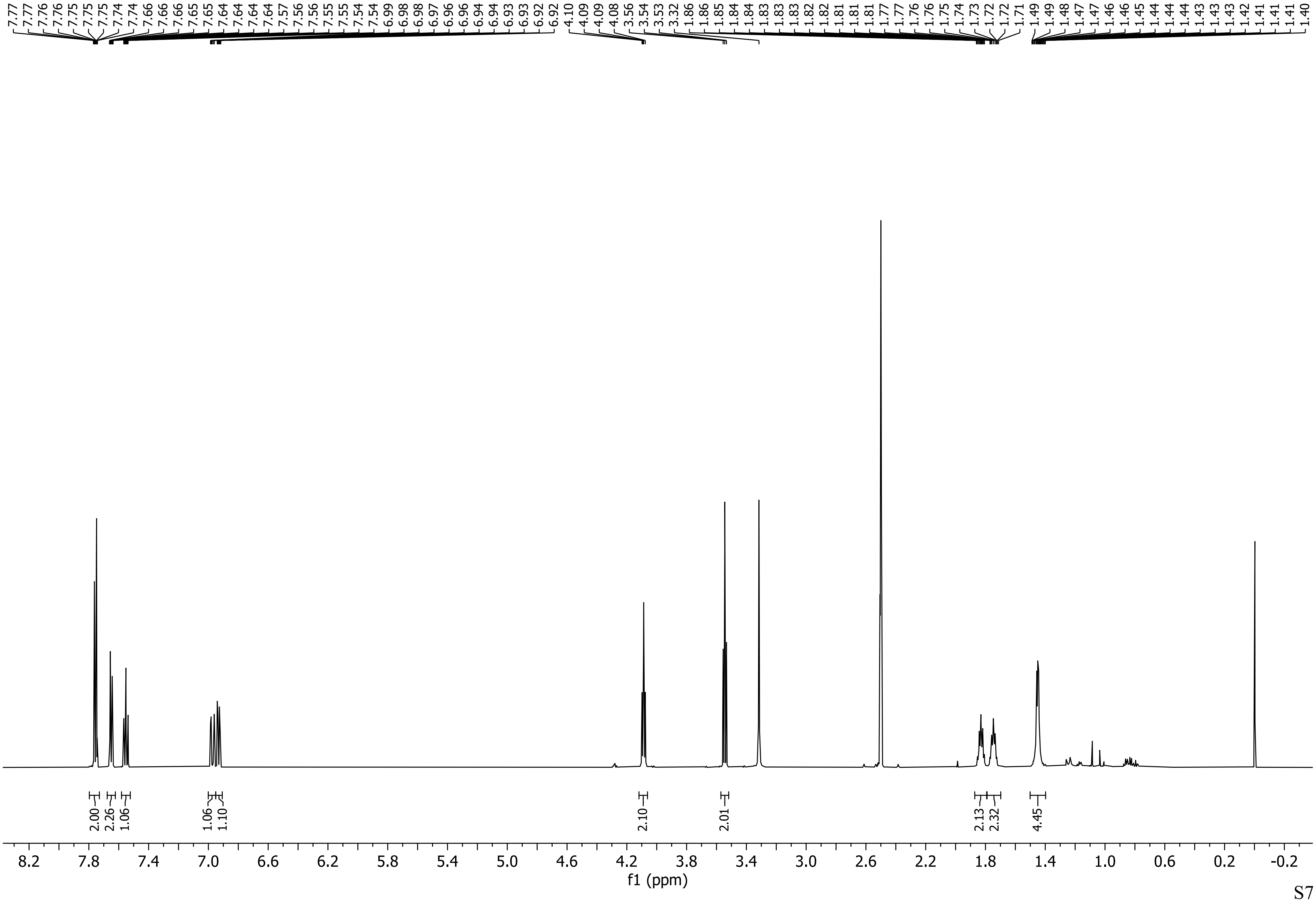

^{13}C NMR (151 MHz, DMSO-d_6) for compound **8a**

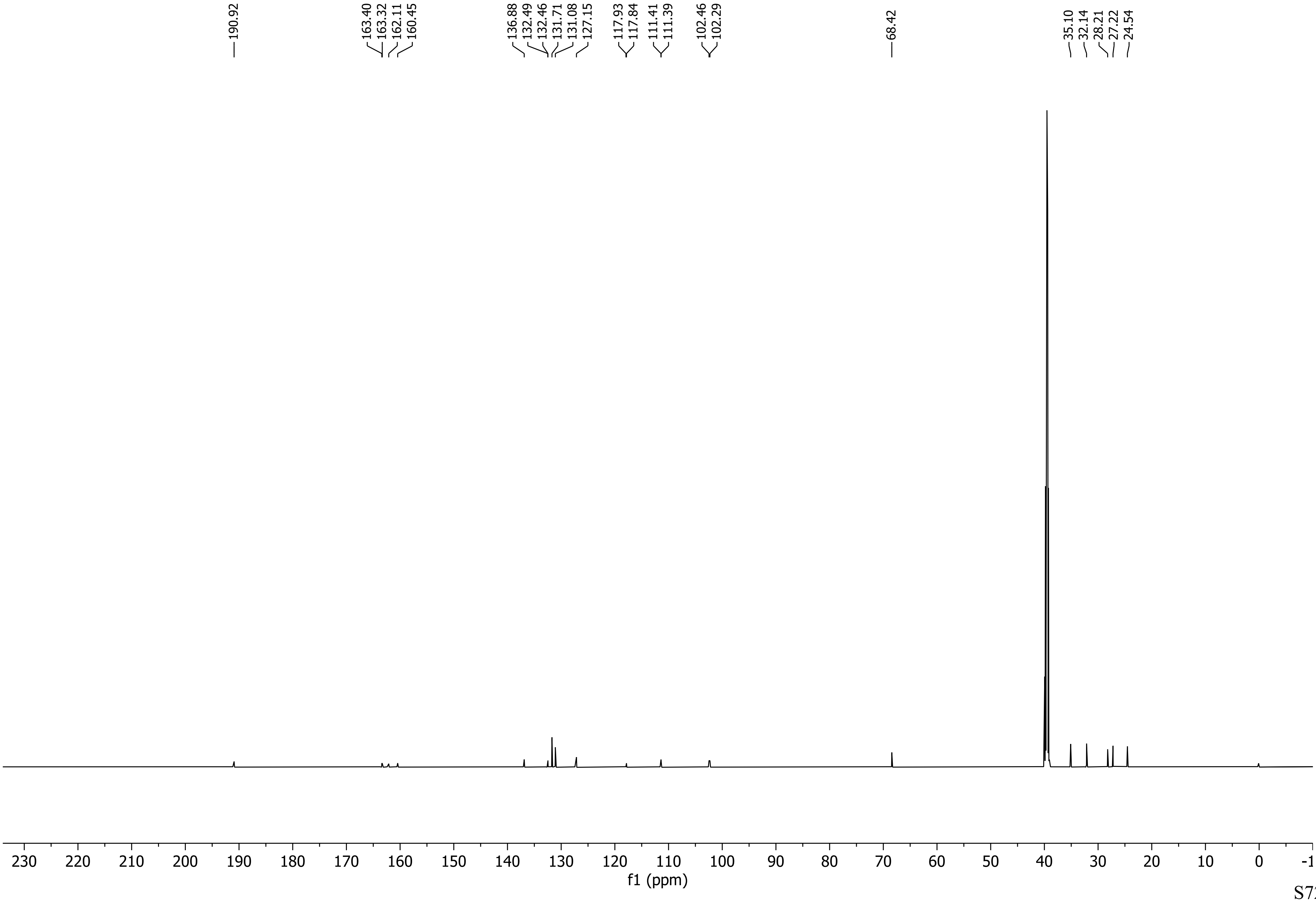

^{13}C NMR (151 MHz, DMSO-d_6) for compound **8a** (zoomed-in view)

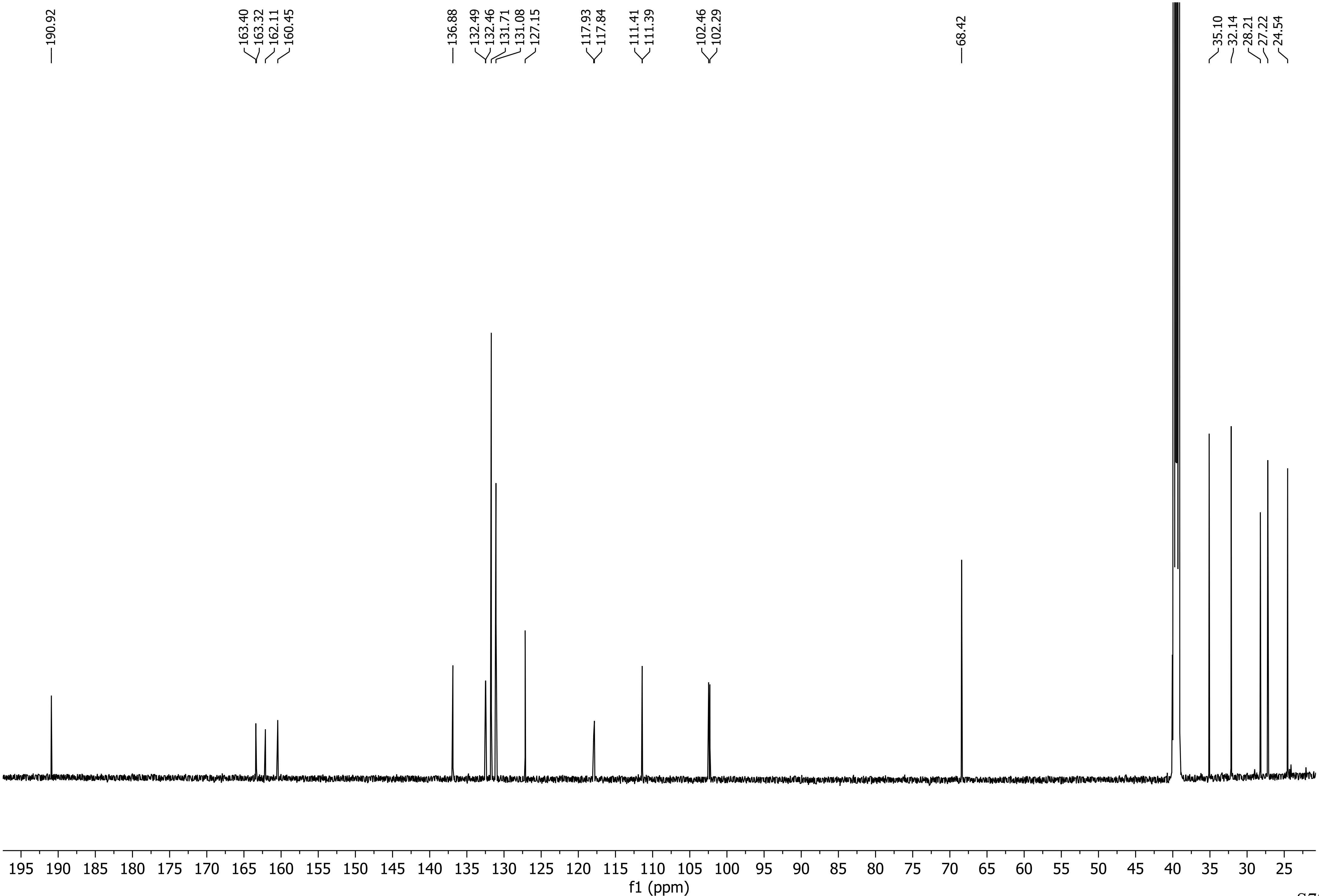

DEPT ^{13}C-NMR (151 MHz, DMSO-d_6) for compound **8a**

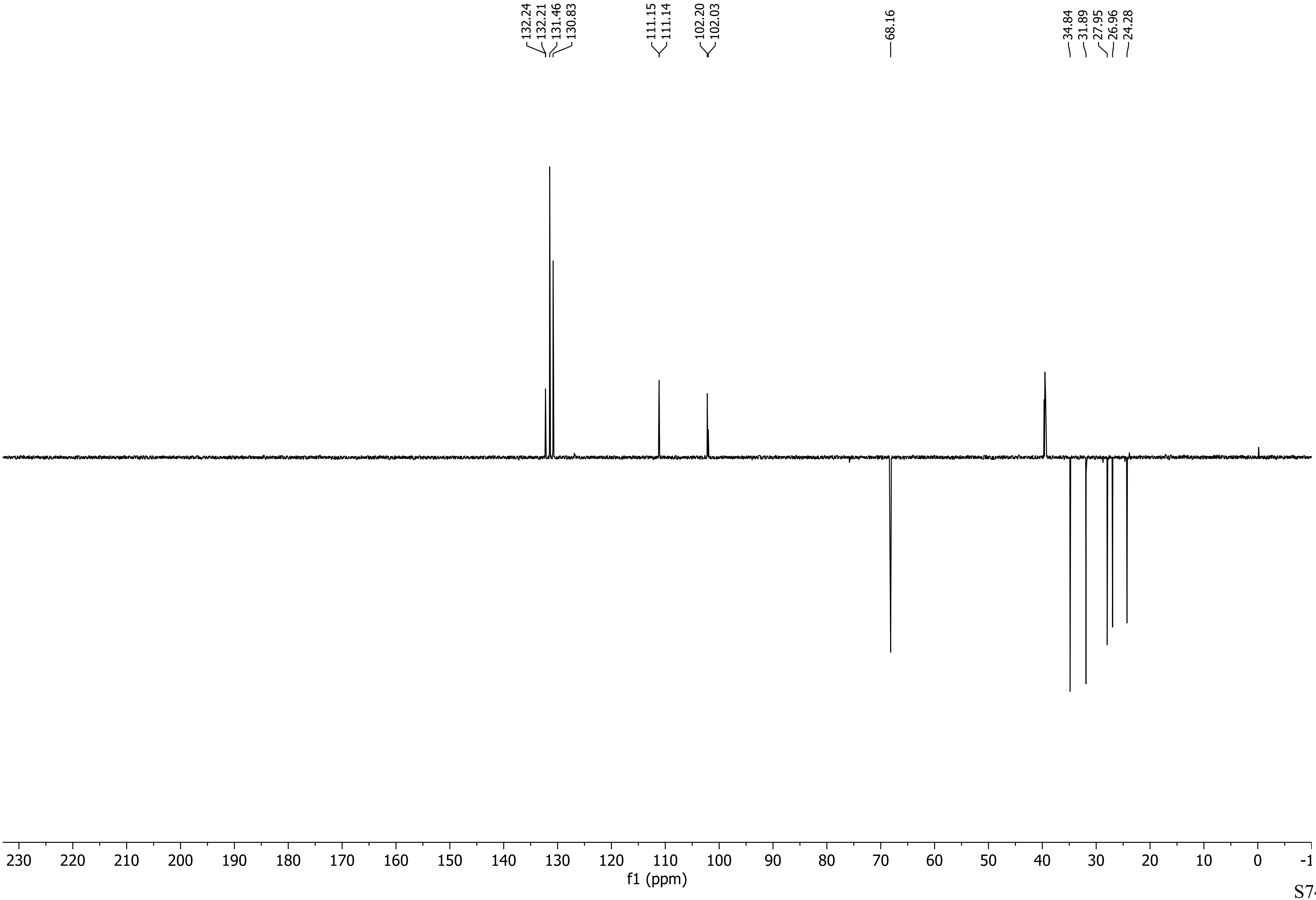

^{1}H NMR (500 MHz, DMSO-d_6) for compound **8b** (zoomed-in view)

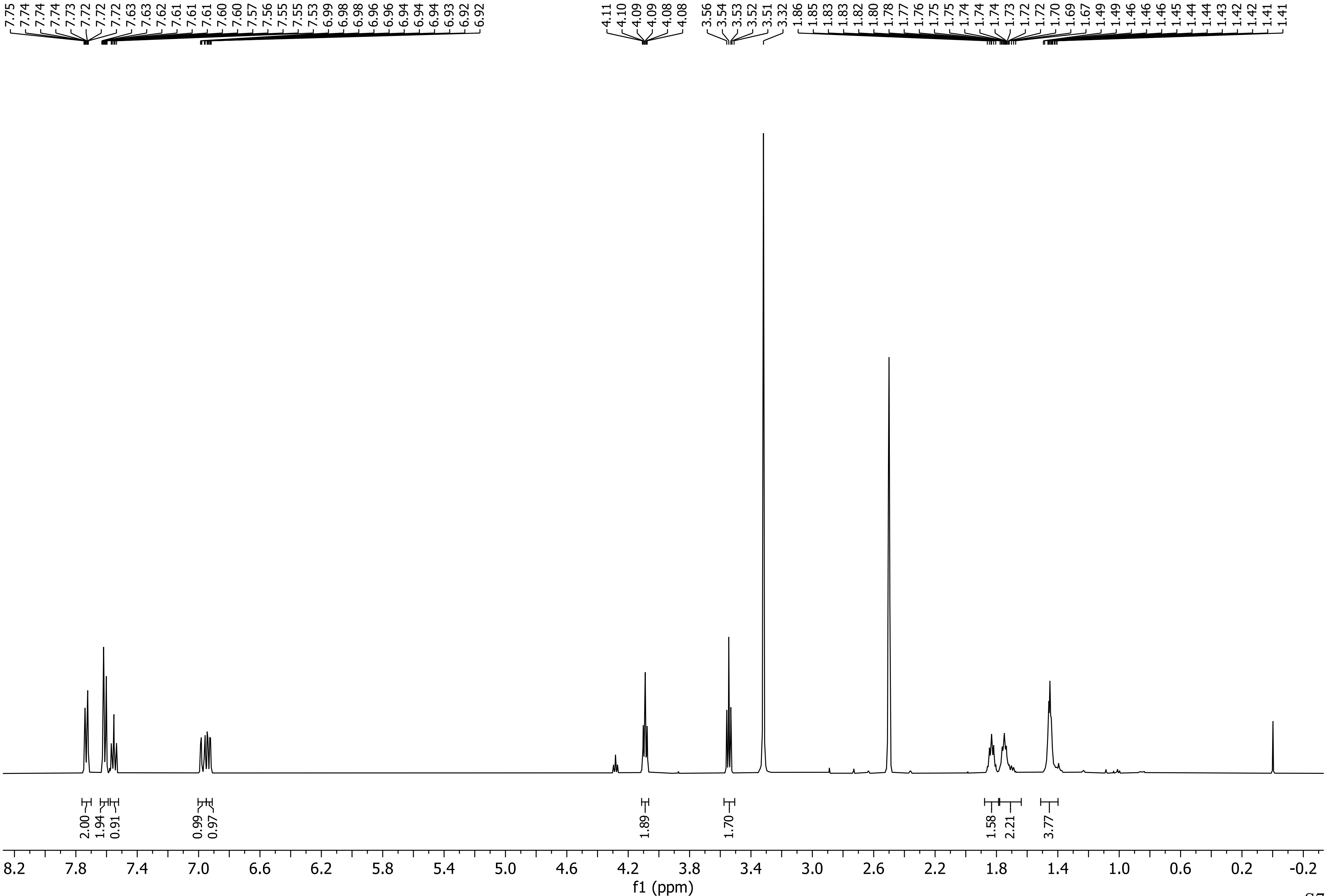

^{13}C NMR (126 MHz, DMSO-d_6) for compound **8b**

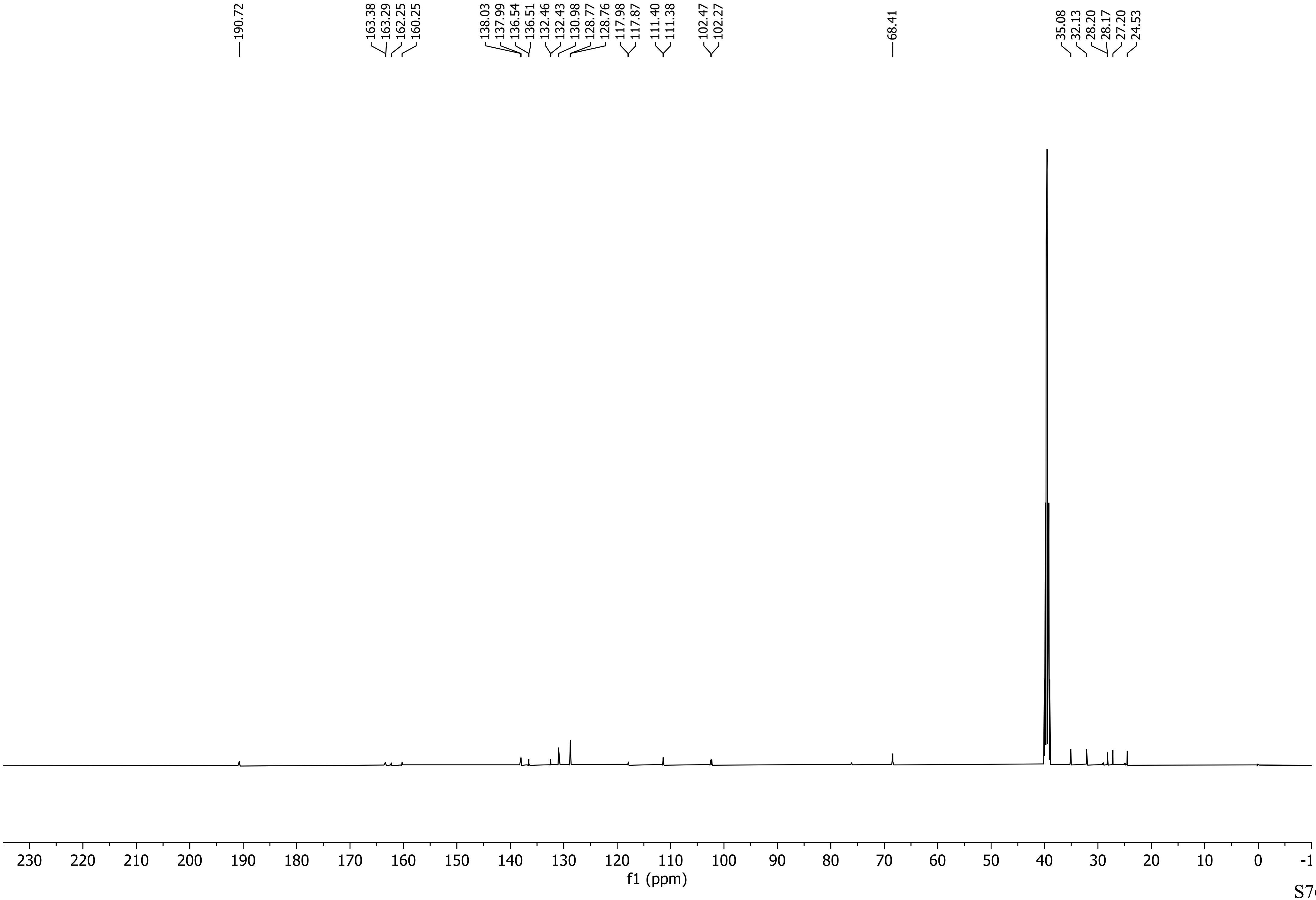

^{13}C NMR (126 MHz, DMSO-d_6) for compound **8b** (zoomed-in view)

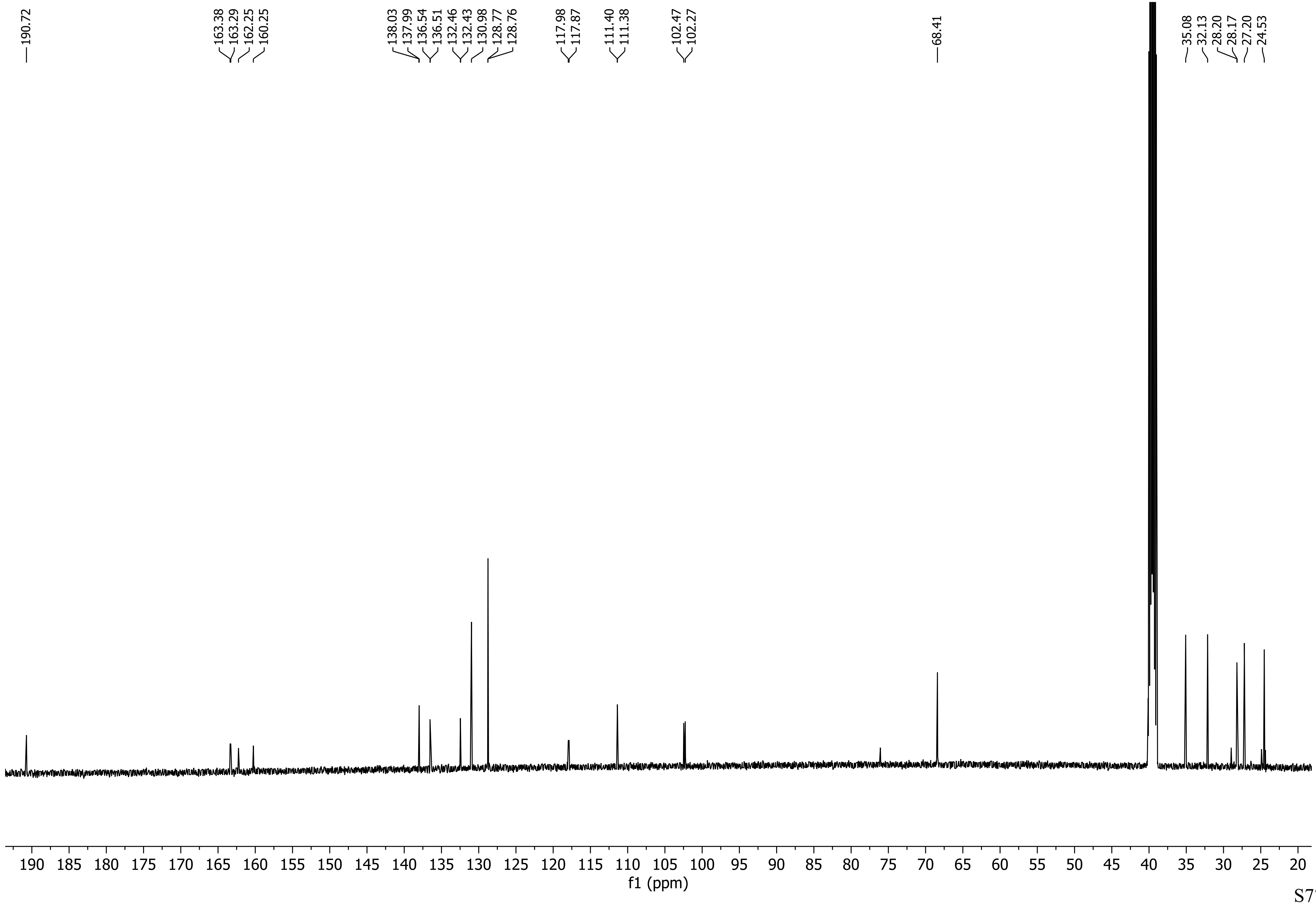

DEPT ^{13}C NMR (126 MHz, DMSO-d_6) for compound **8b**

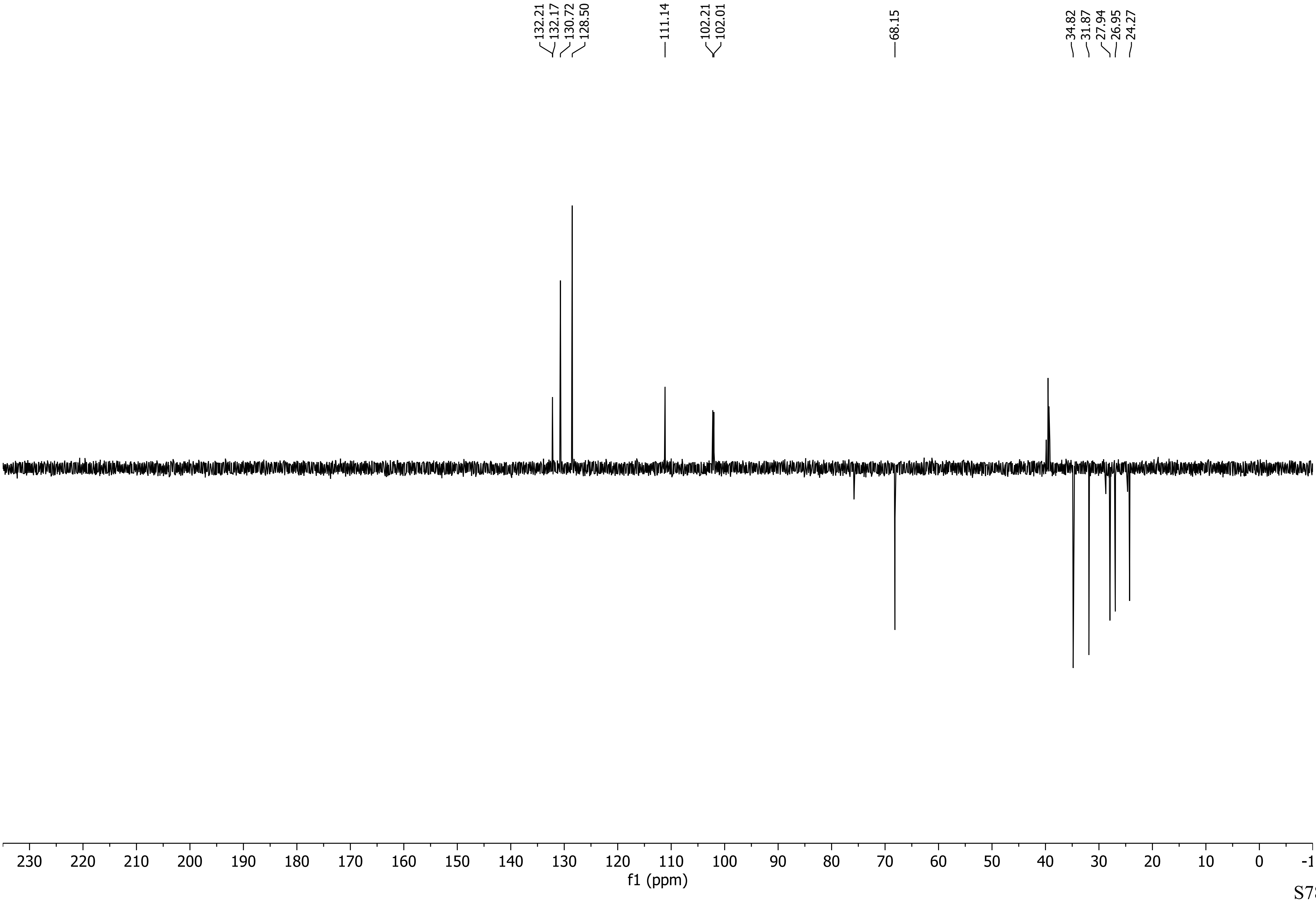

^{1}H NMR (500 MHz, DMSO-d_6) for compound **8c** (zoomed-in view)

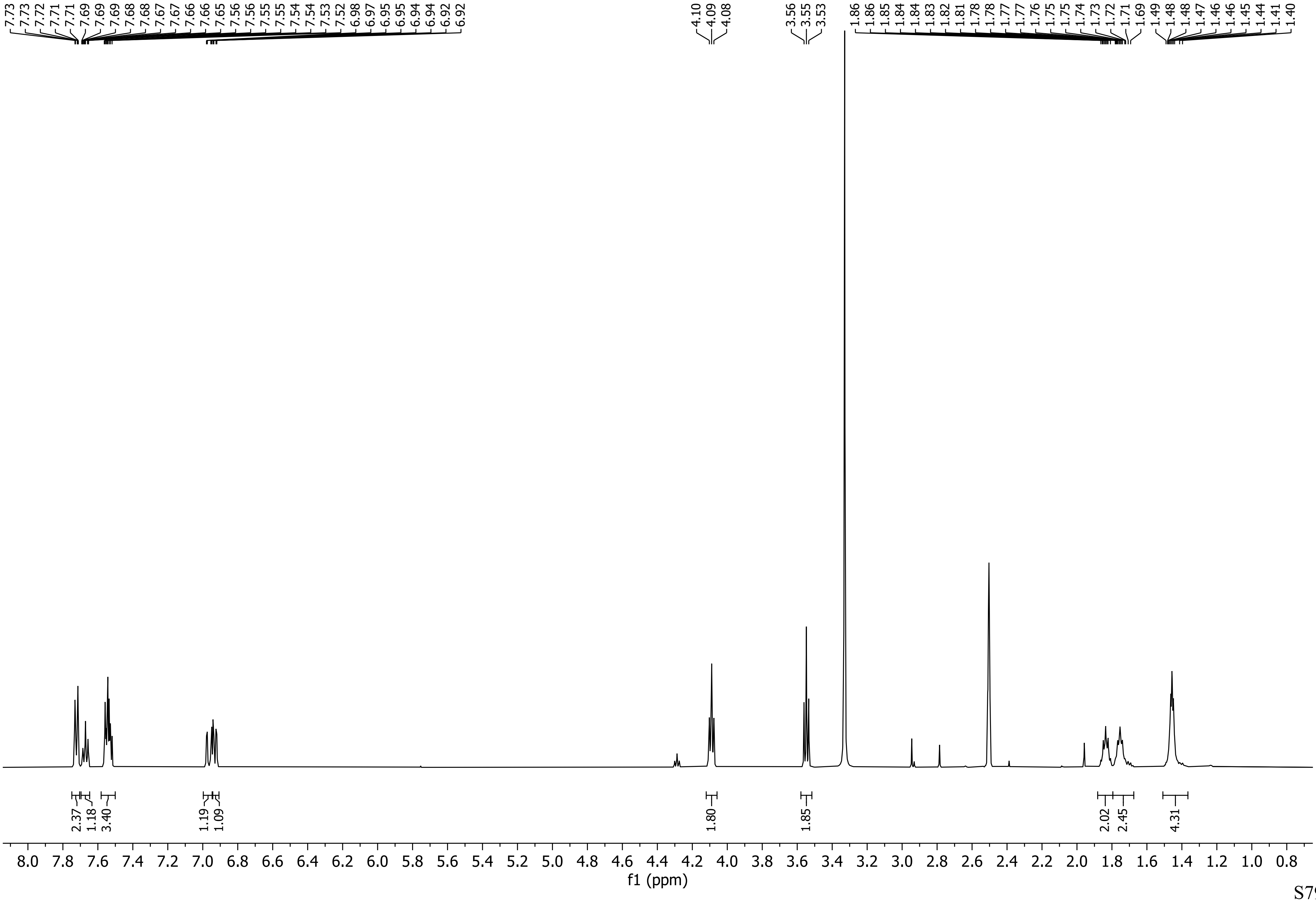

^{13}C NMR (126 MHz, DMSO-d_6) for compound **8c**

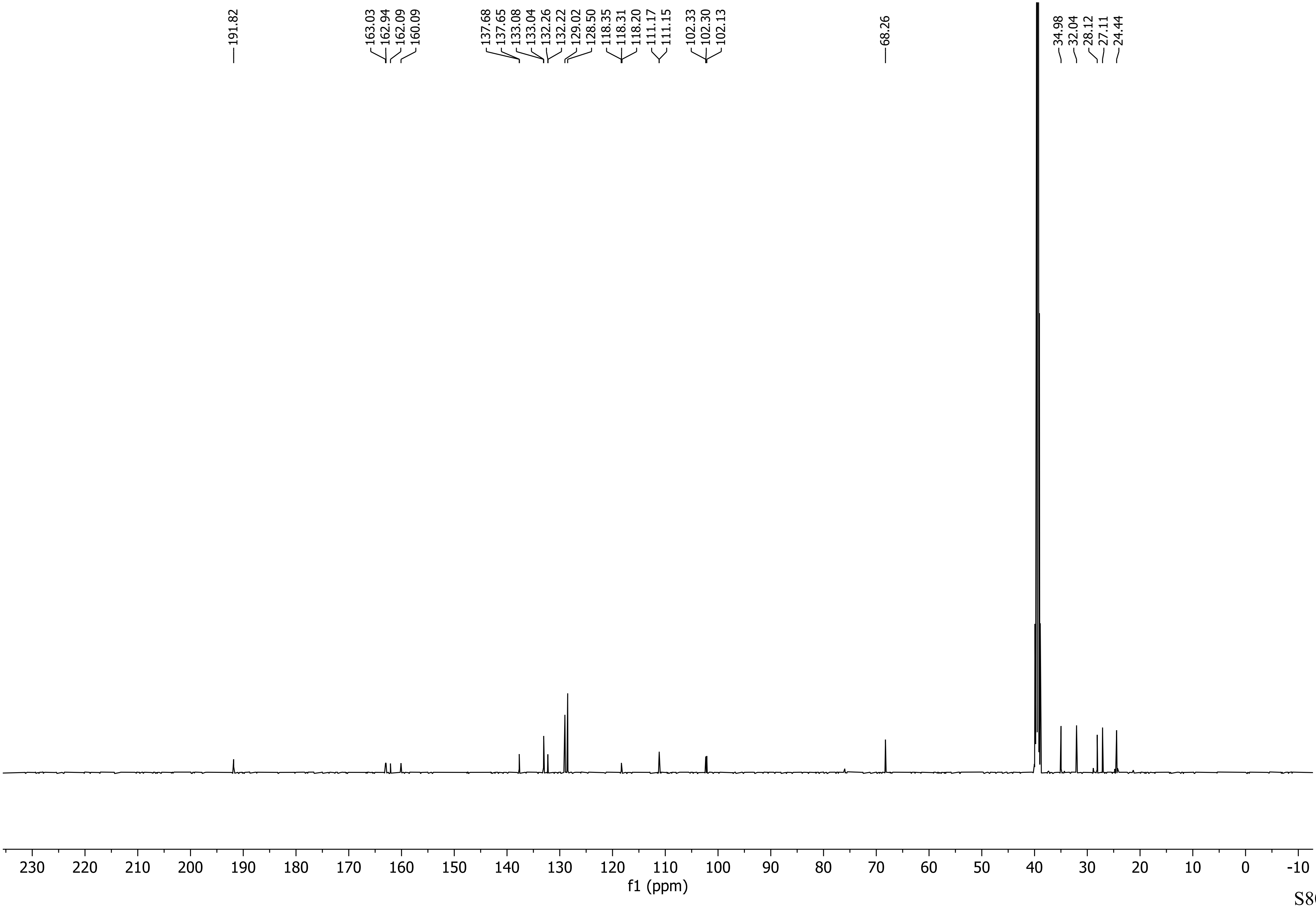

^{13}C NMR (126 MHz, DMSO-d_6) for compound **8c** (zoomed-in view)

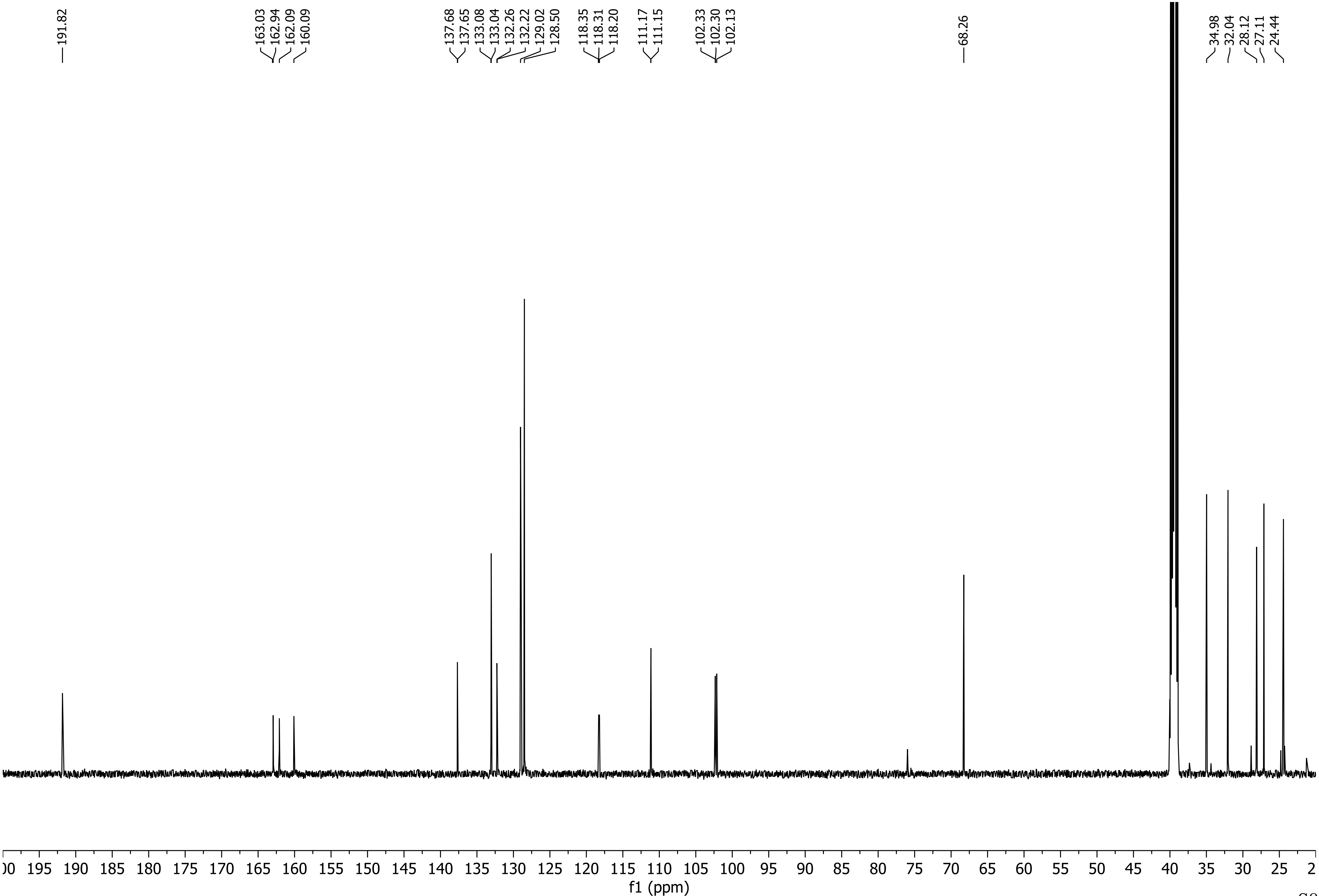

DEPT ^{13}C NMR (126 MHz, DMSO-d_6) for compound **8c** (zoomed-in view)

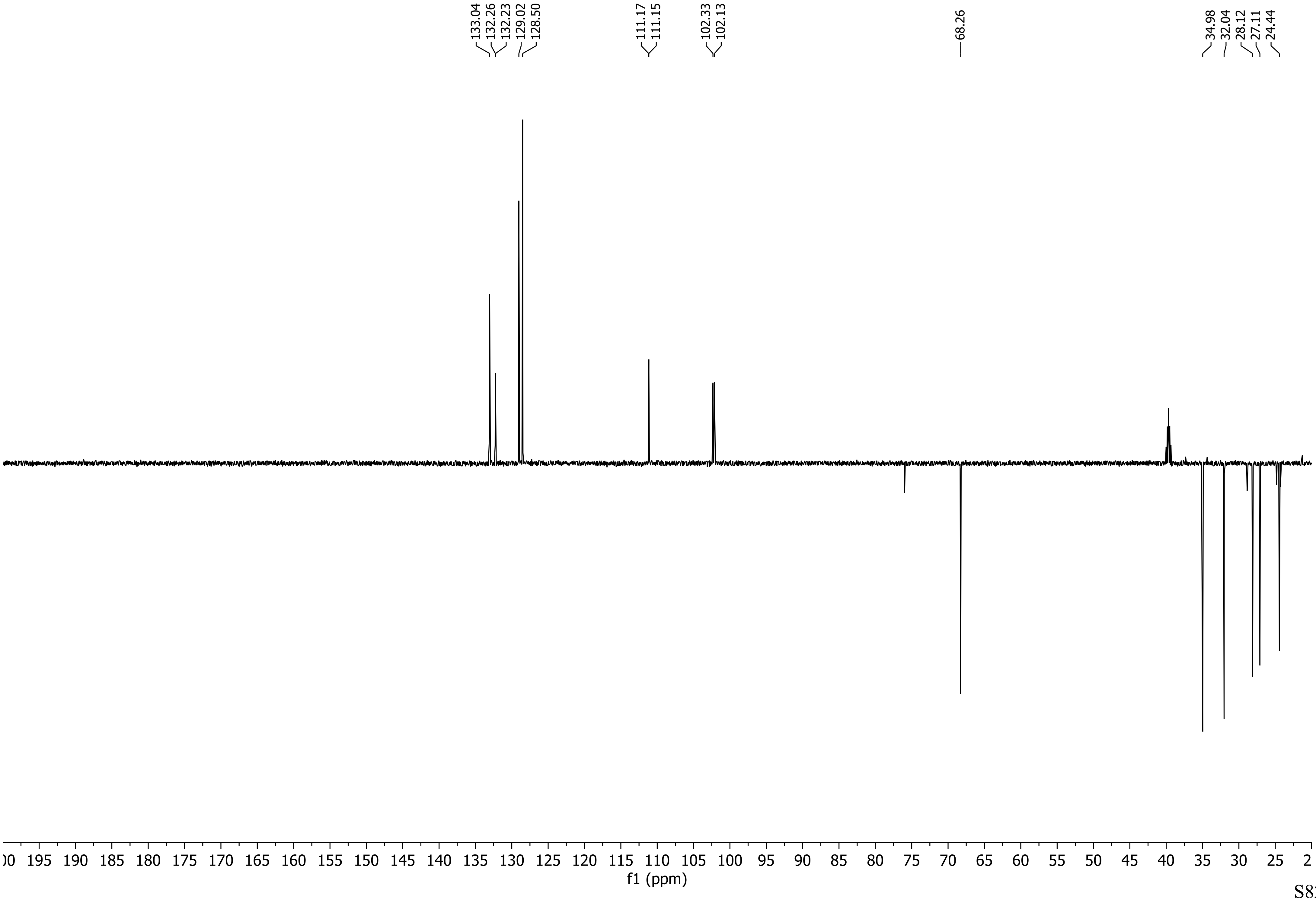

^{1}H NMR (500 MHz, DMSO-d_6) for compound **8d** (zoomed-in view)

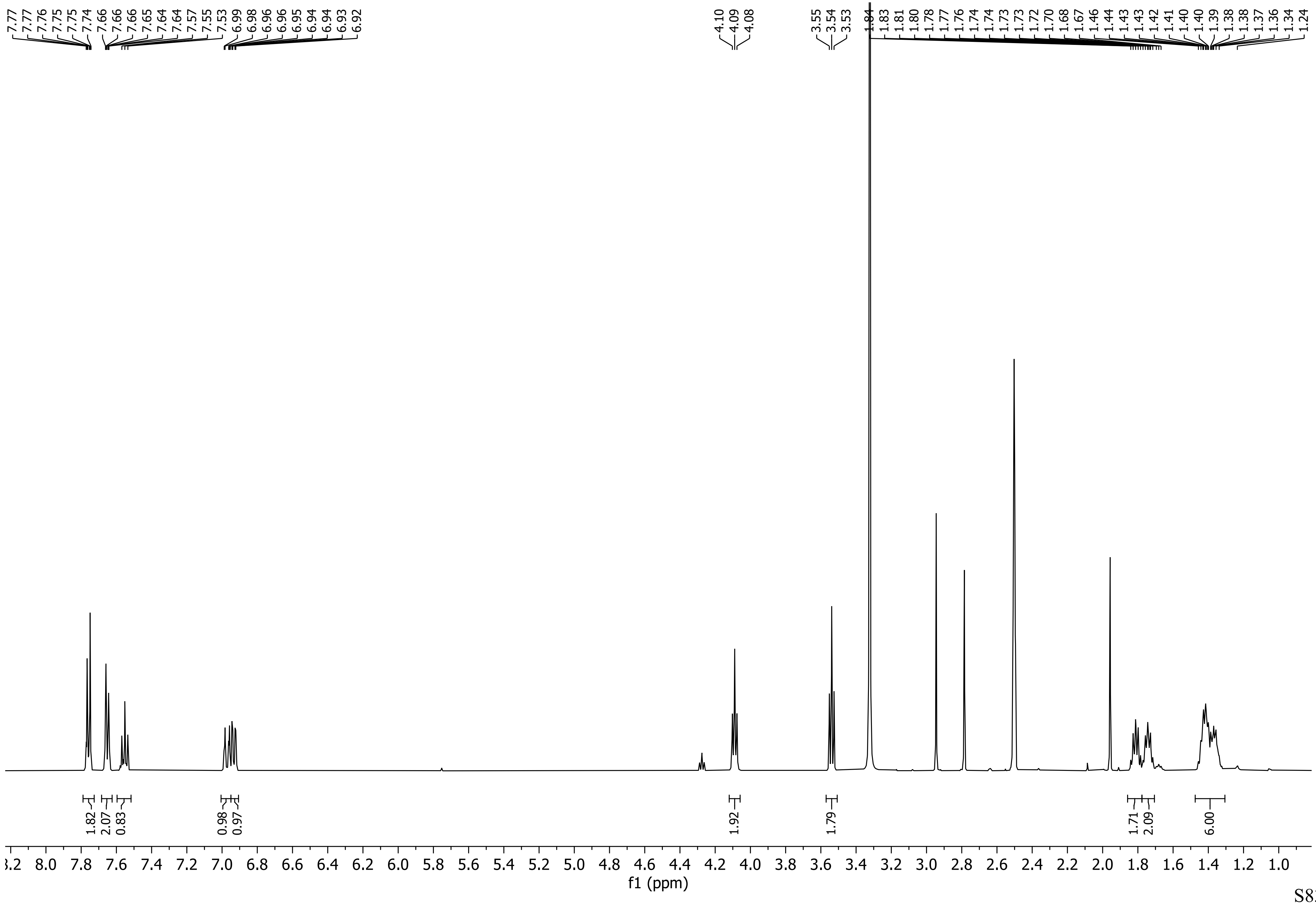

^{13}C NMR (126 MHz, DMSO-d_6) for compound **8d**

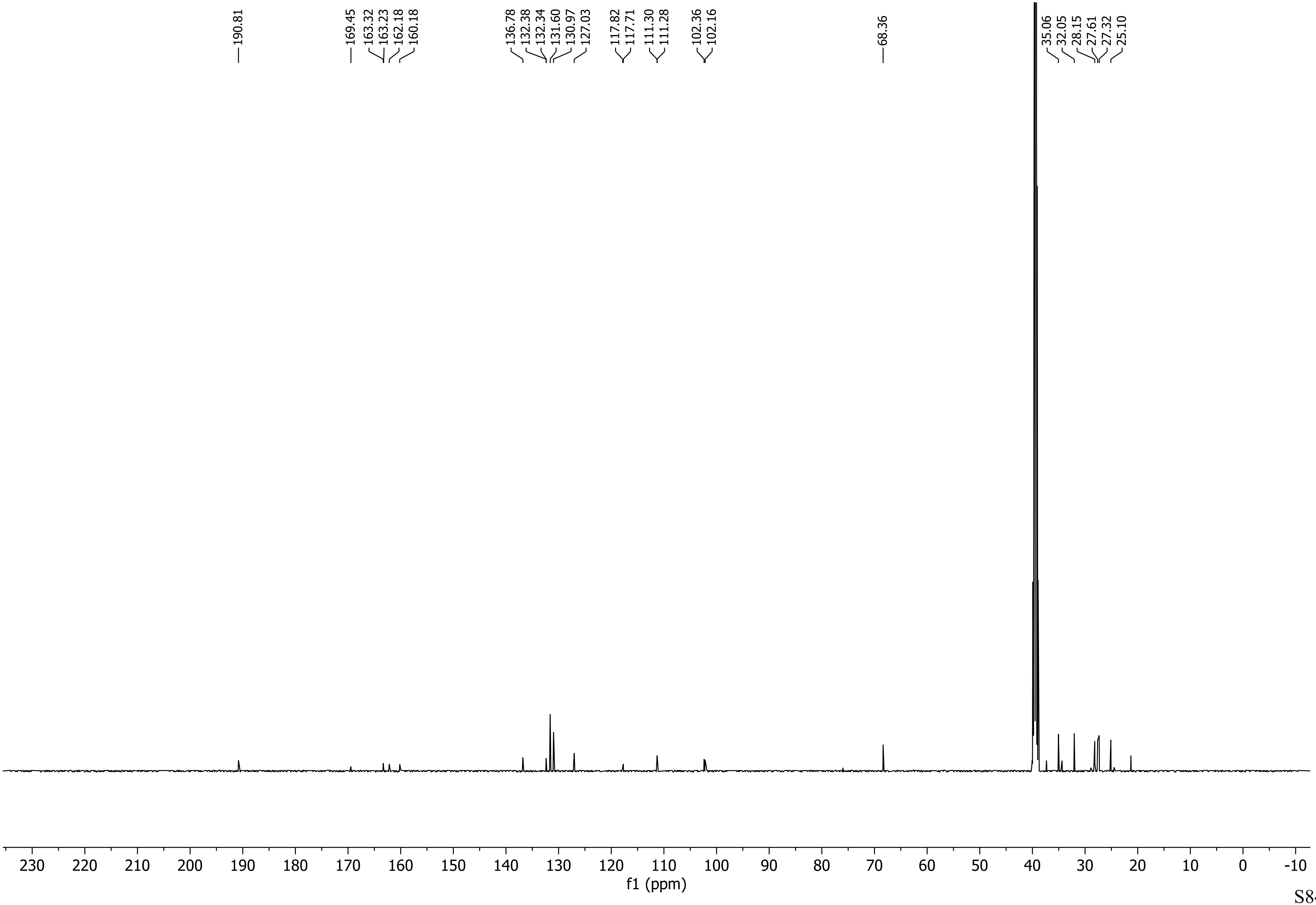

^{13}C NMR (126 MHz, DMSO-d_6) for compound **8d** (zoomed-in view)

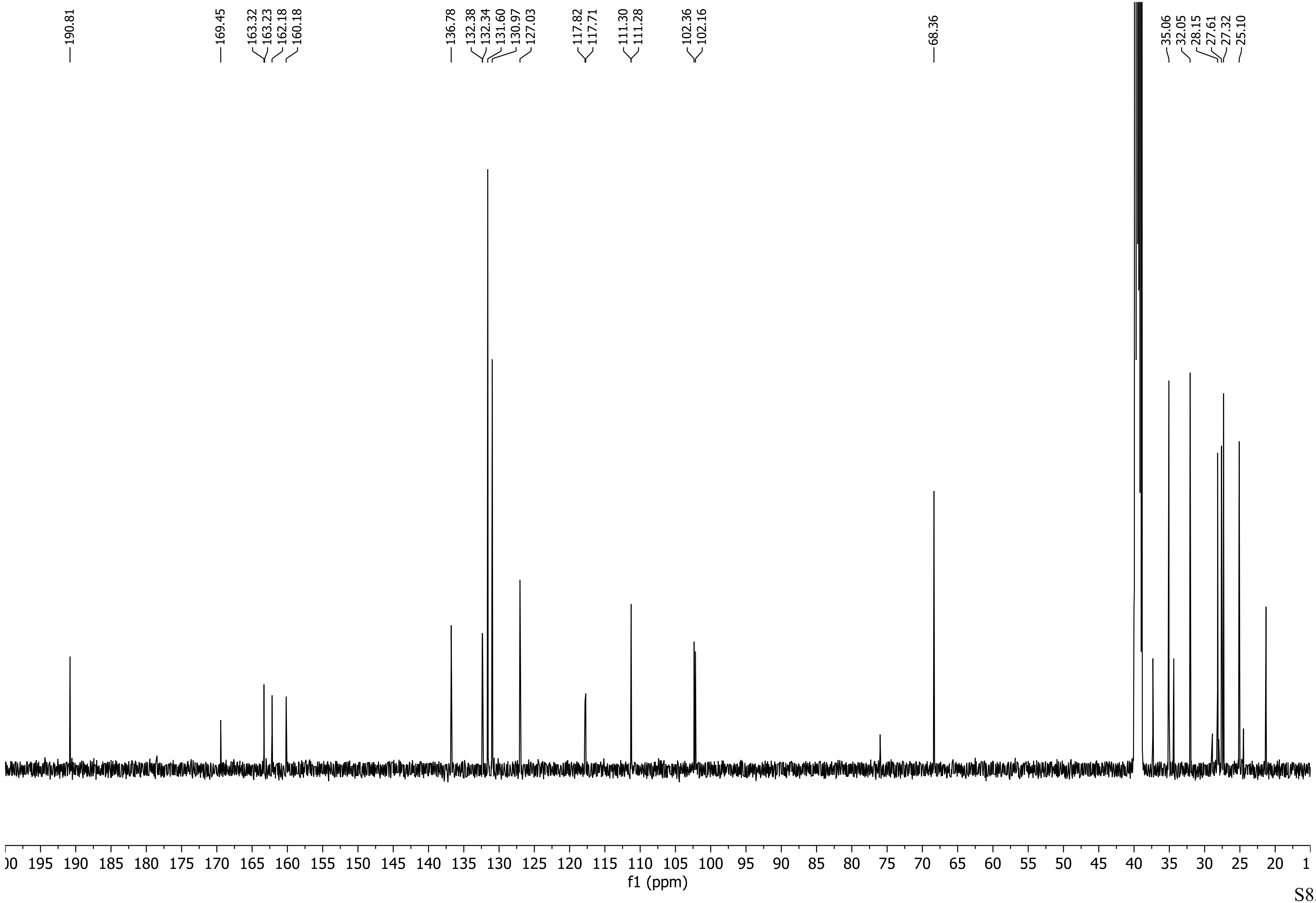

DEPT ^{13}C NMR (126 MHz, DMSO-d_6) for compound **8d** (zoomed-in view)

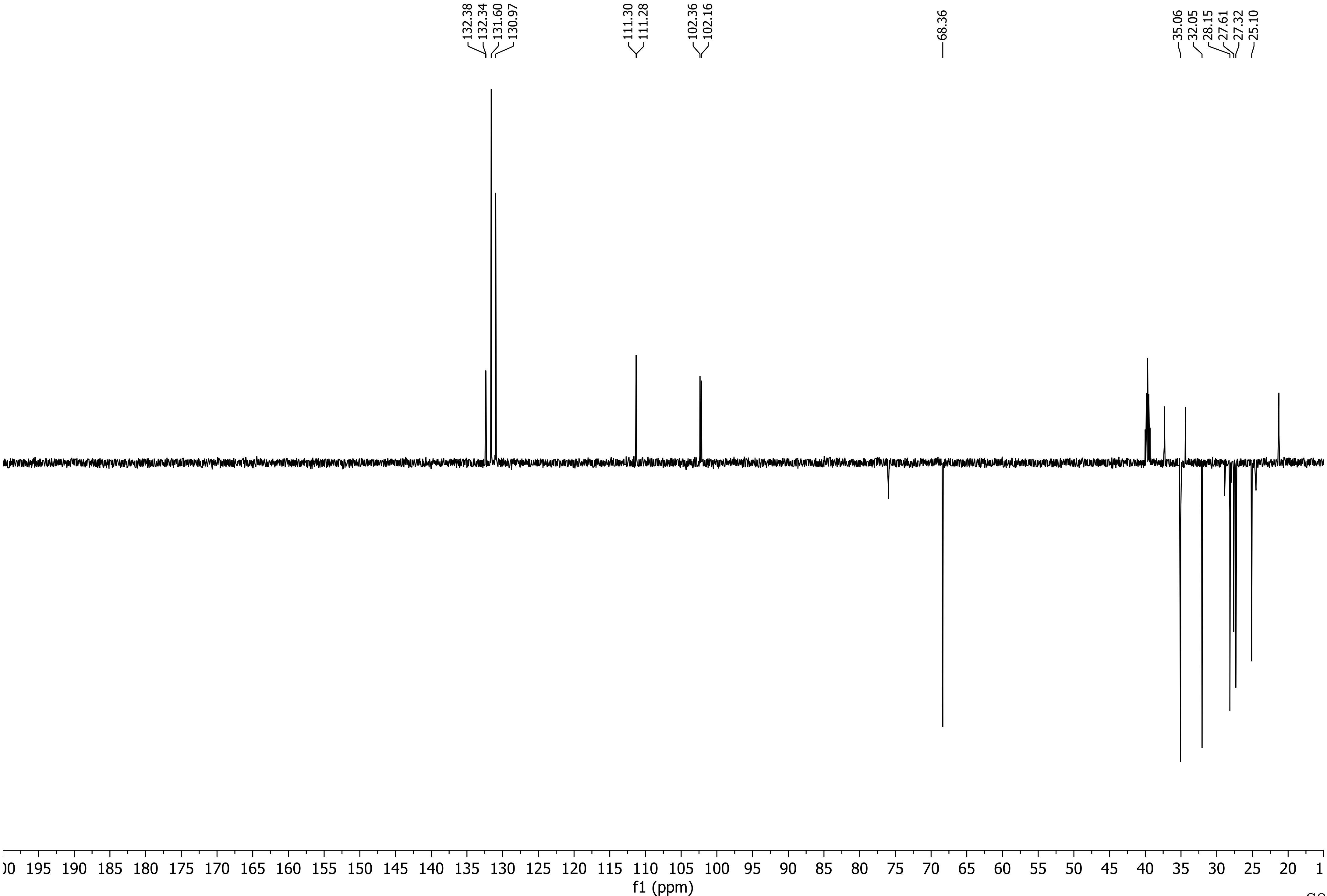

^{1}H NMR (500 MHz, DMSO-d_6) for compound **9** (zoomed-in view)

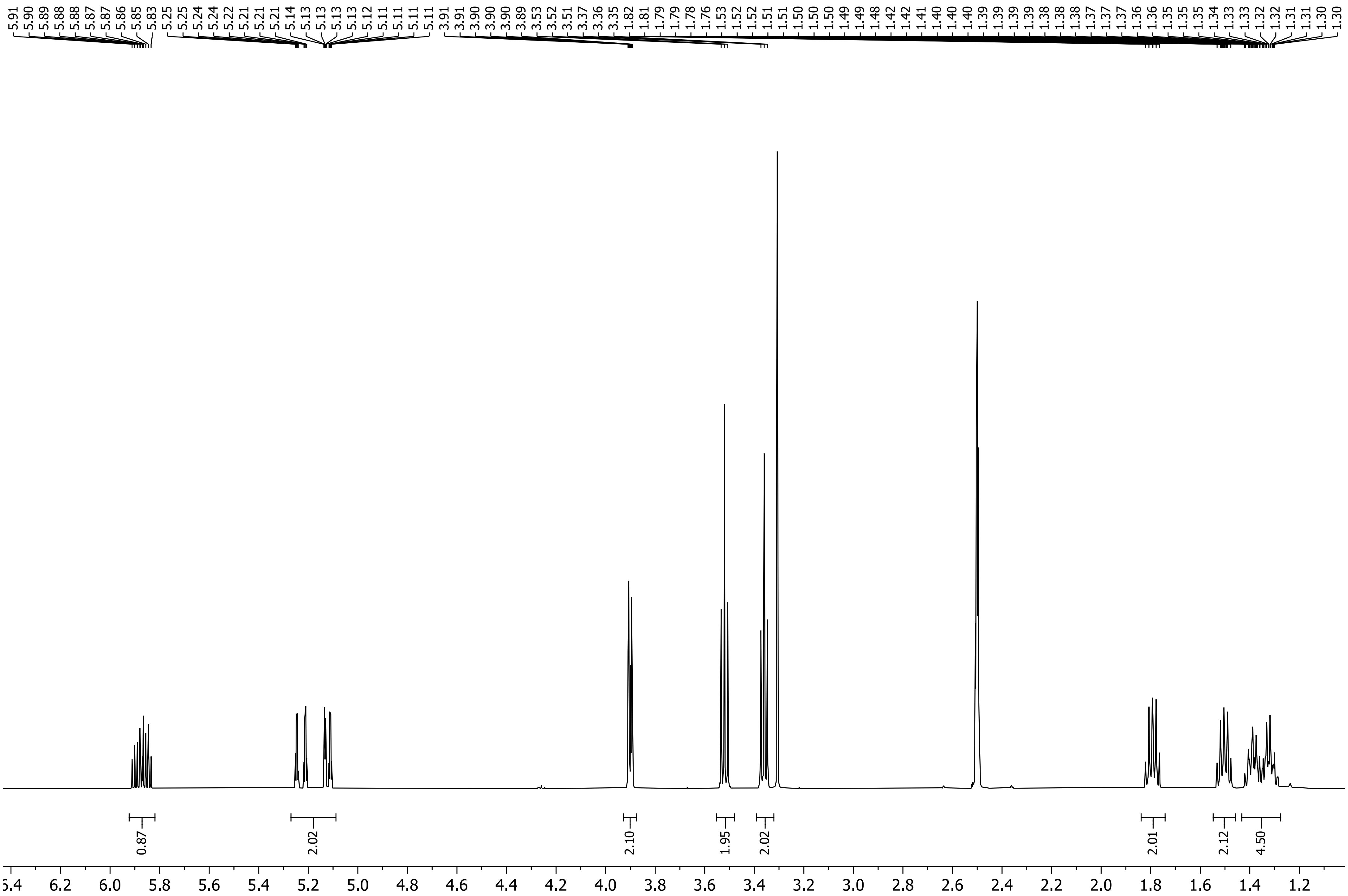

^{13}C NMR (126 MHz, DMSO-d_6) for compound **9**

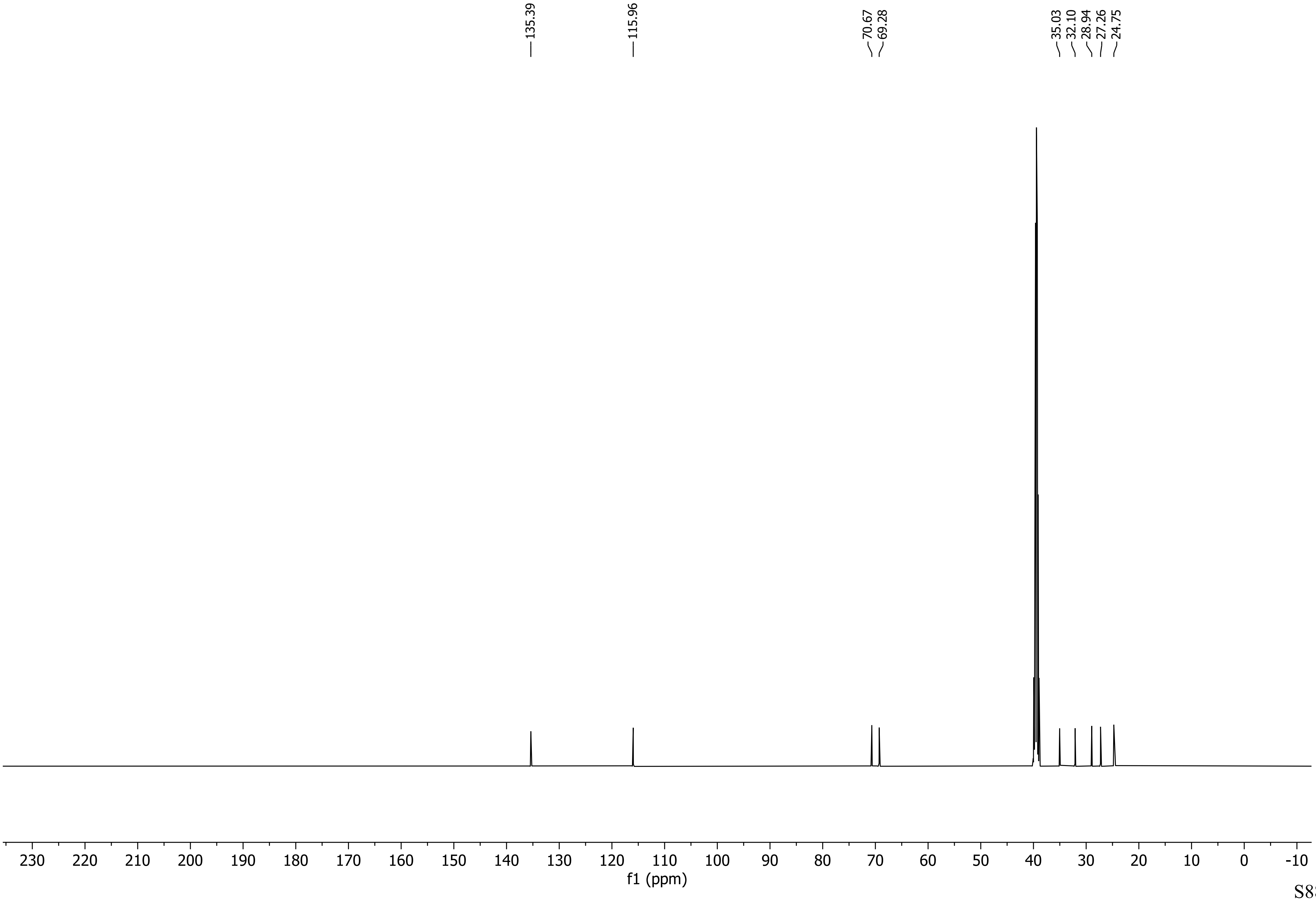

^{13}C NMR (126 MHz, DMSO-d_6) for compound **9** (zoomed-in view)

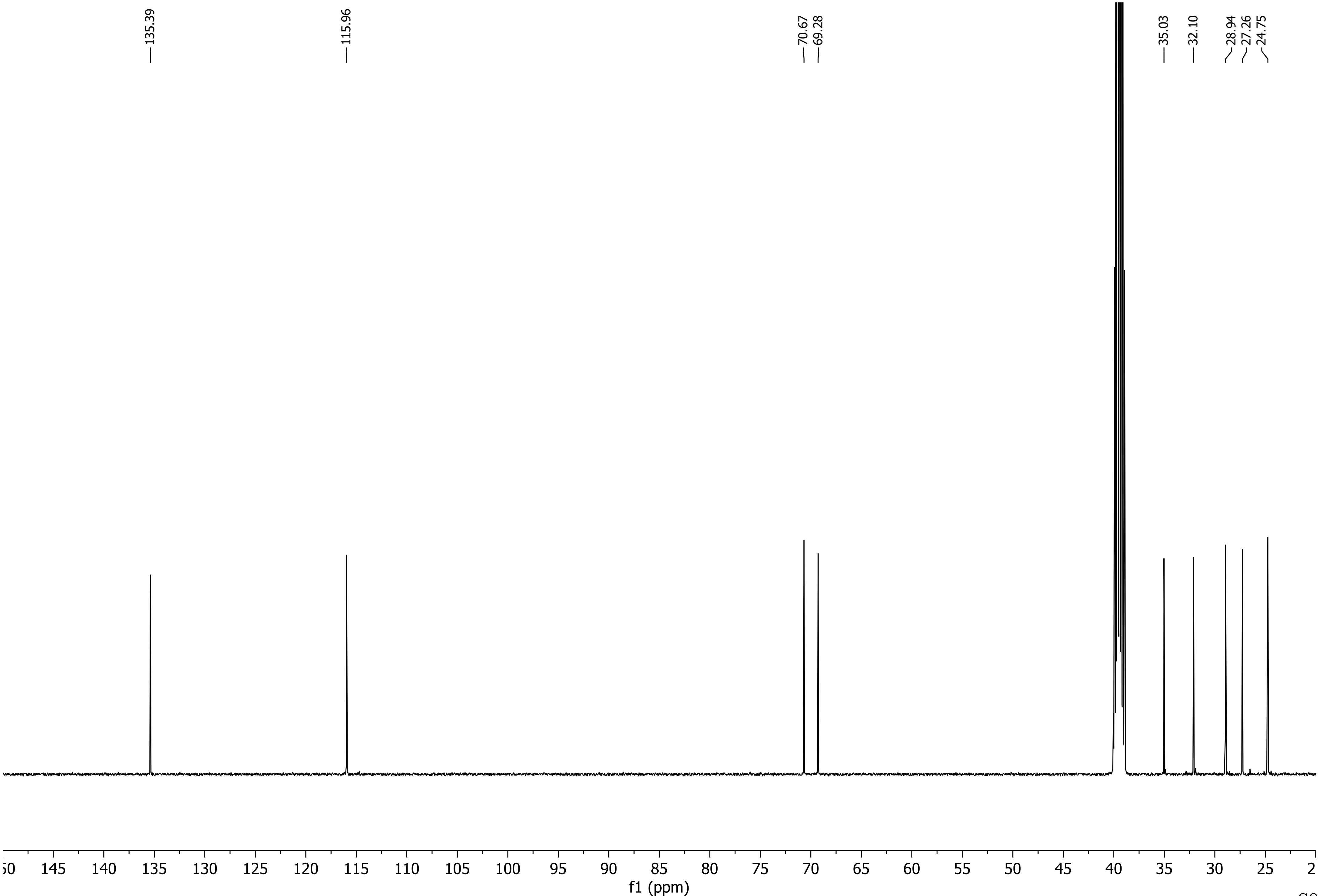

DEPT ^{13}C NMR (126 MHz, DMSO-d_6) for compound **9** (zoomed-in view)

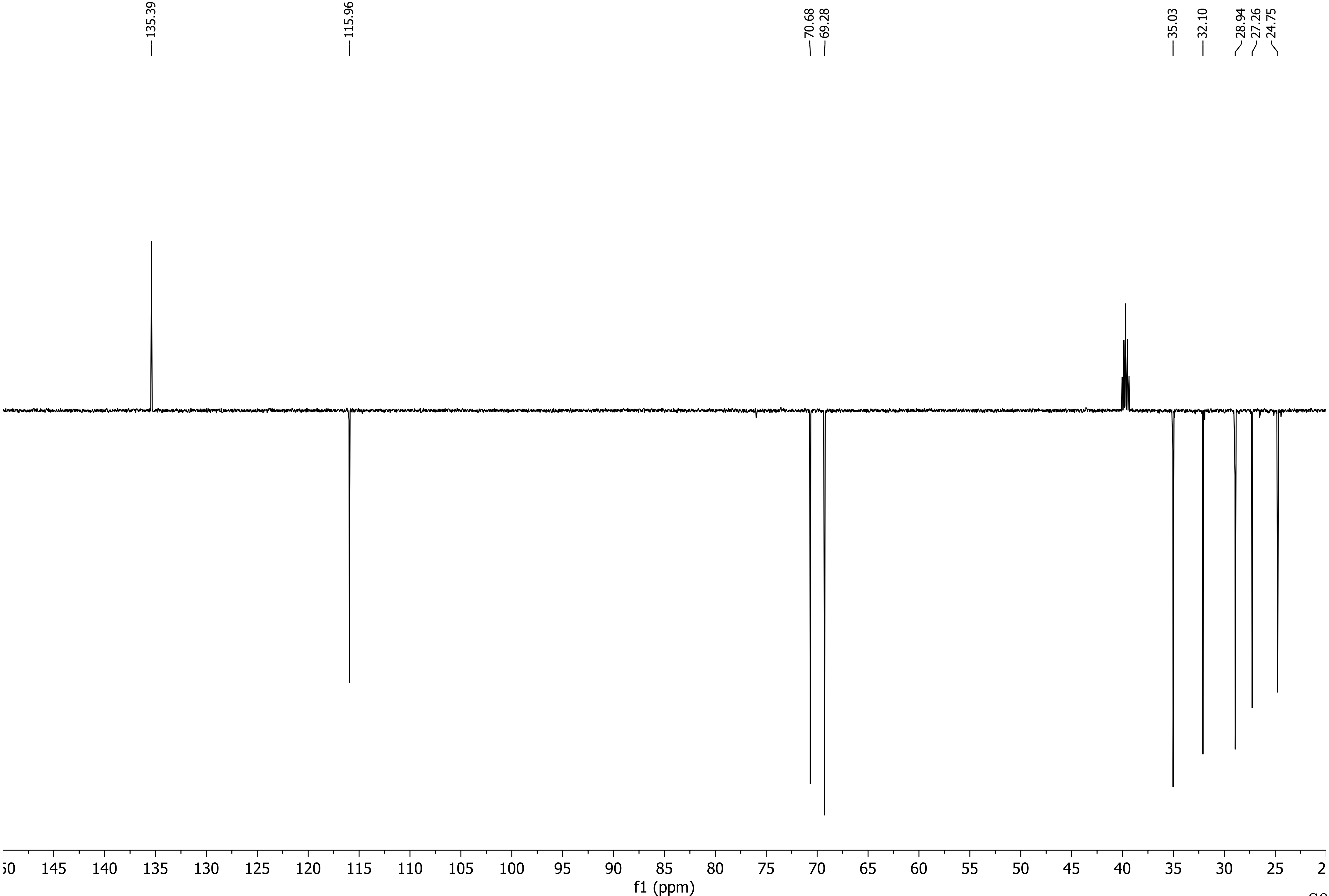

Supplementary HPLC chromatograms

HPLC chromatogram of Ro 48-8071 (**3**):

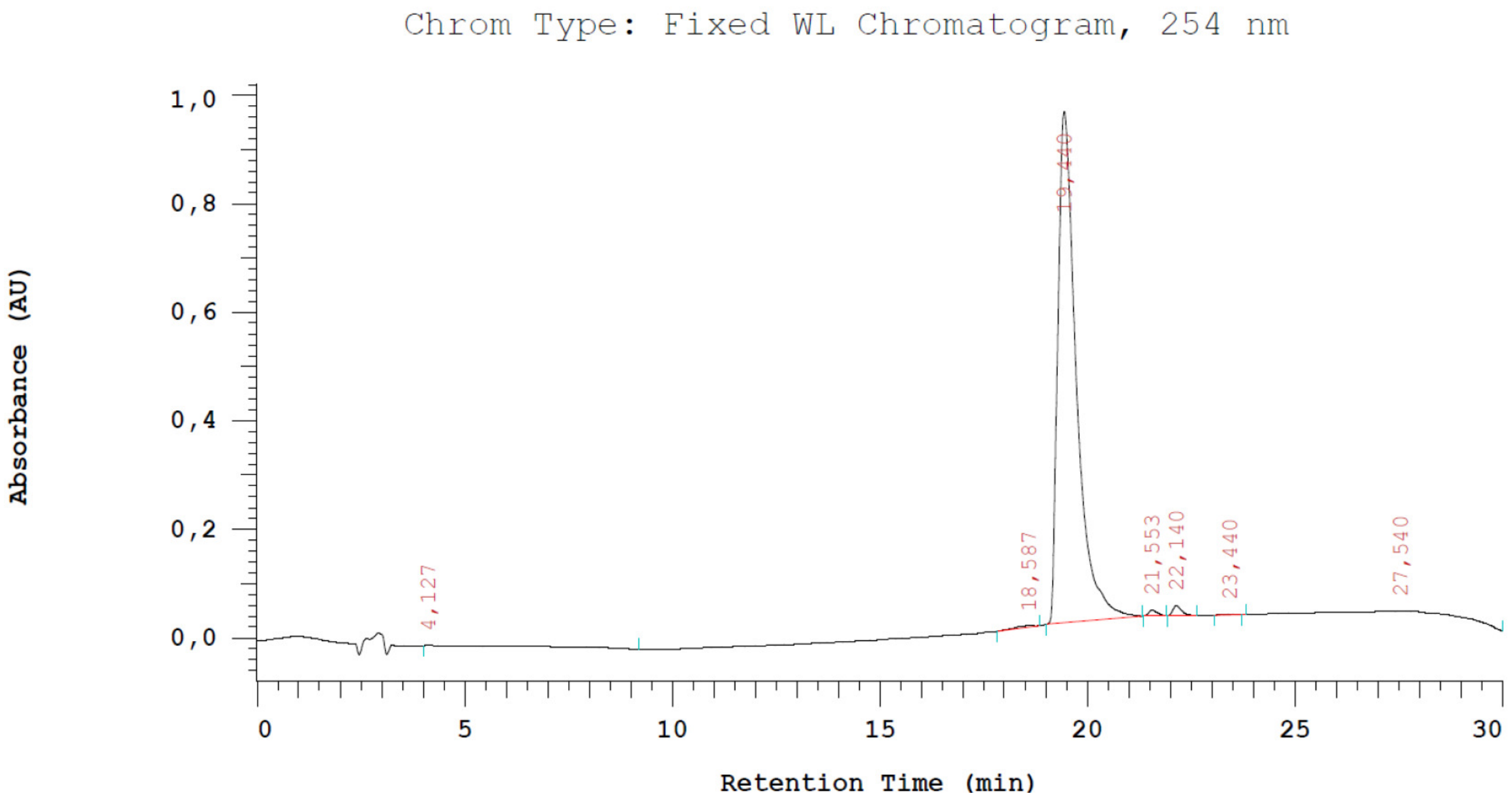

No.	RT	Area	Conc 1	BC
1	4,127	0	0,000	
2	18,587	50165	0,341	MC
3	19,440	14466735	98,200	MC
4	21,553	72854	0,495	MC
5	22,140	133111	0,904	MC
6	23,440	9023	0,061	MC
7	27,540	0	0,000	
		14731888	100,000	

HPLC chromatogram of **4a**:

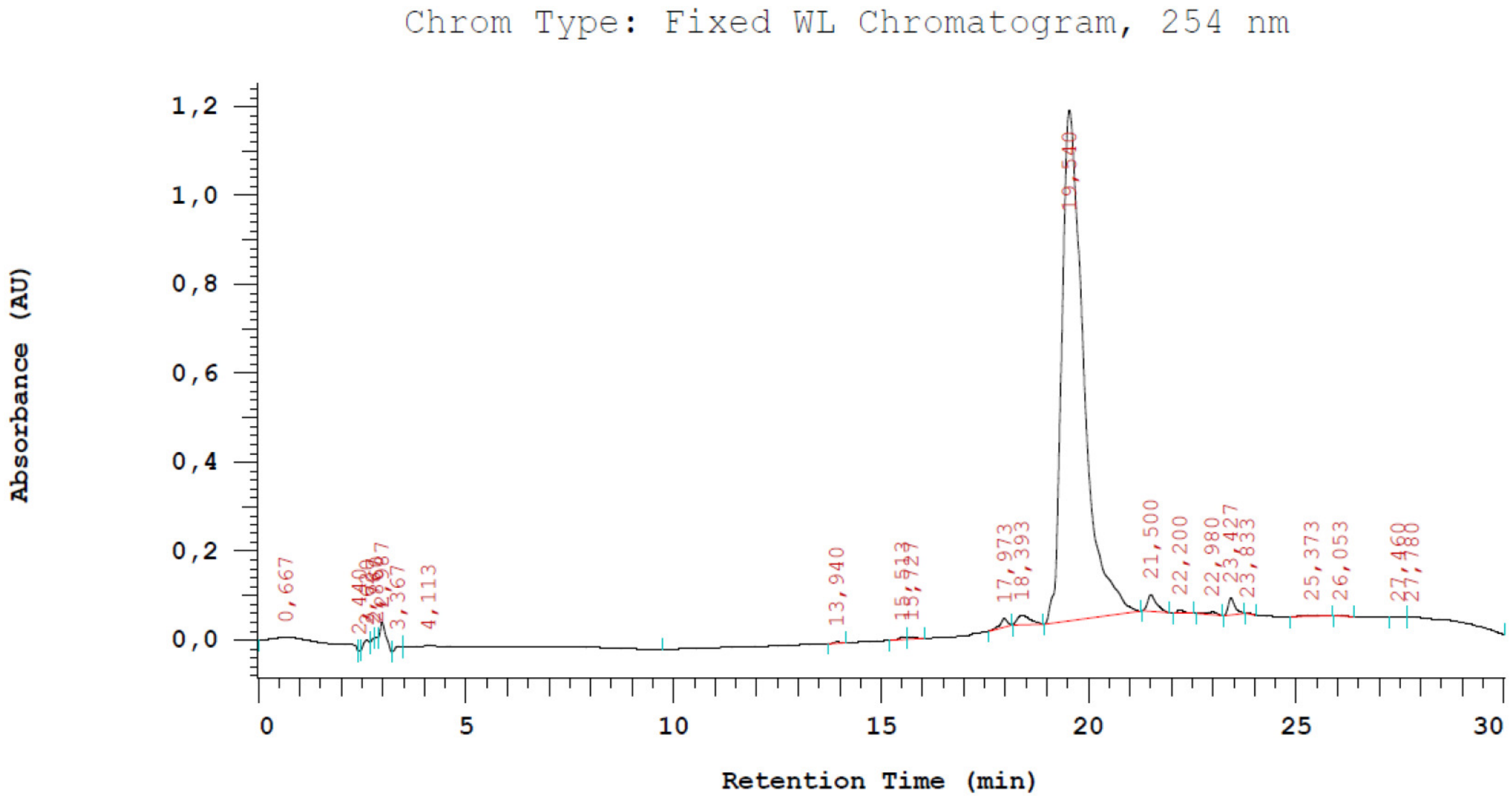

No.	RT	Area	Conc 1	BC
1	0,667	0	0,000	
2	2,440	0	0,000	
3	2,620	0	0,000	
4	2,787	0	0,000	
5	2,860	0	0,000	
6	2,987	0	0,000	
7	3,367	0	0,000	
8	4,113	0	0,000	
9	13,940	15494	0,070	MC
10	15,513	31640	0,143	MC
11	15,727	35822	0,162	MC
12	17,973	110503	0,501	MC
13	18,393	223406	1,013	MC
14	19,540	20991073	95,159	MC
15	21,500	284574	1,290	MC
16	22,200	38824	0,176	MC
17	22,980	56718	0,257	MC
18	23,427	208783	0,946	MC
19	23,833	5214	0,024	MC
20	25,373	41368	0,188	MC
21	26,053	15581	0,071	MC
22	27,460	0	0,000	
23	27,780	0	0,000	
		22059000	100,000	

HPLC chromatogram of **4b**:

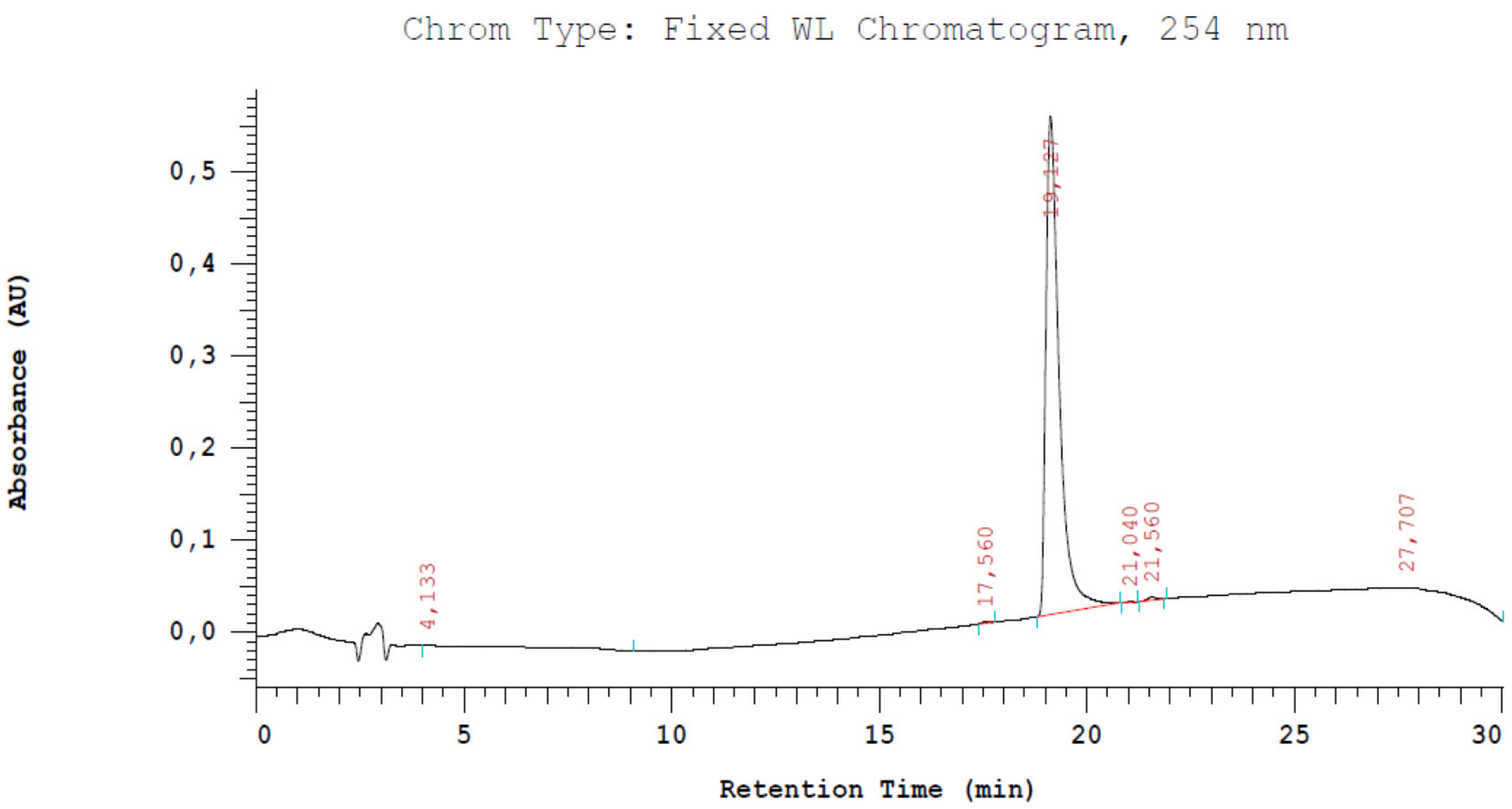

No.	RT	Area	Conc 1	BC
1	4,133	0	0,000	
2	17,560	10423	0,173	MC
3	19,127	5973839	99,372	MC
4	21,040	3160	0,053	MC
5	21,560	24156	0,402	MC
6	27,707	0	0,000	
		6011578	100,000	

HPLC chromatogram of **4c**:

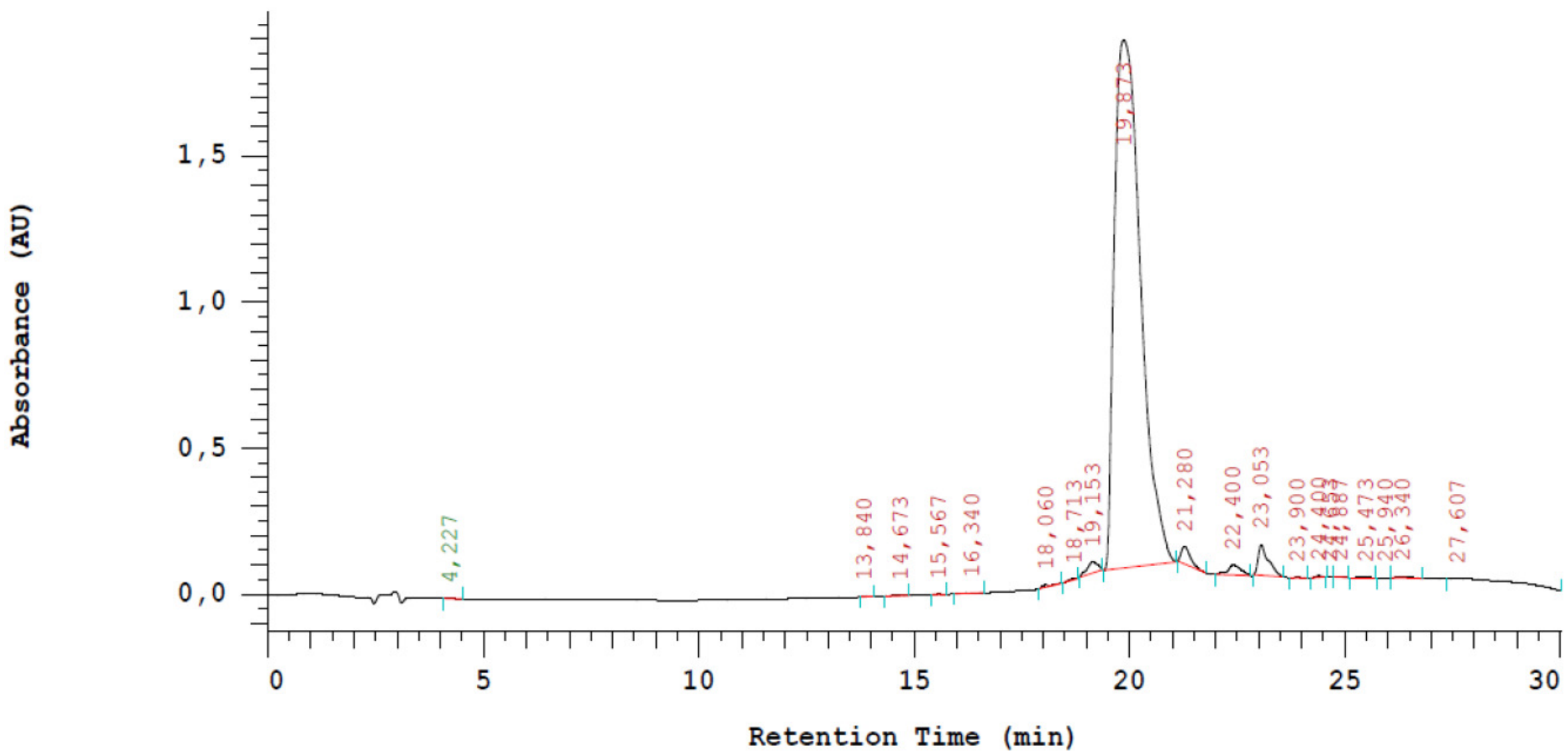

No.	RT	Area	Conc 1	BC
1	4,227	1607	0,004	BB
2	13,840	12688	0,032	MC
3	14,673	17619	0,044	MC
4	15,567	7091	0,018	MC
5	16,340	18971	0,047	MC
6	18,060	49470	0,123	MC
7	18,713	24459	0,061	MC
8	19,153	316590	0,788	MC
9	19,873	37992384	94,508	MC
10	21,280	438060	1,090	MC
11	22,400	350691	0,872	MC
12	23,053	847126	2,107	MC
13	23,900	10040	0,025	MC
14	24,400	29090	0,072	MC
15	24,653	0	0,000	
16	24,887	15938	0,040	MC
17	25,473	15031	0,037	MC
18	25,940	0	0,000	
19	26,340	53179	0,132	MC
20	27,607	0	0,000	
		40200034	100,000	

HPLC chromatogram of **4d**:

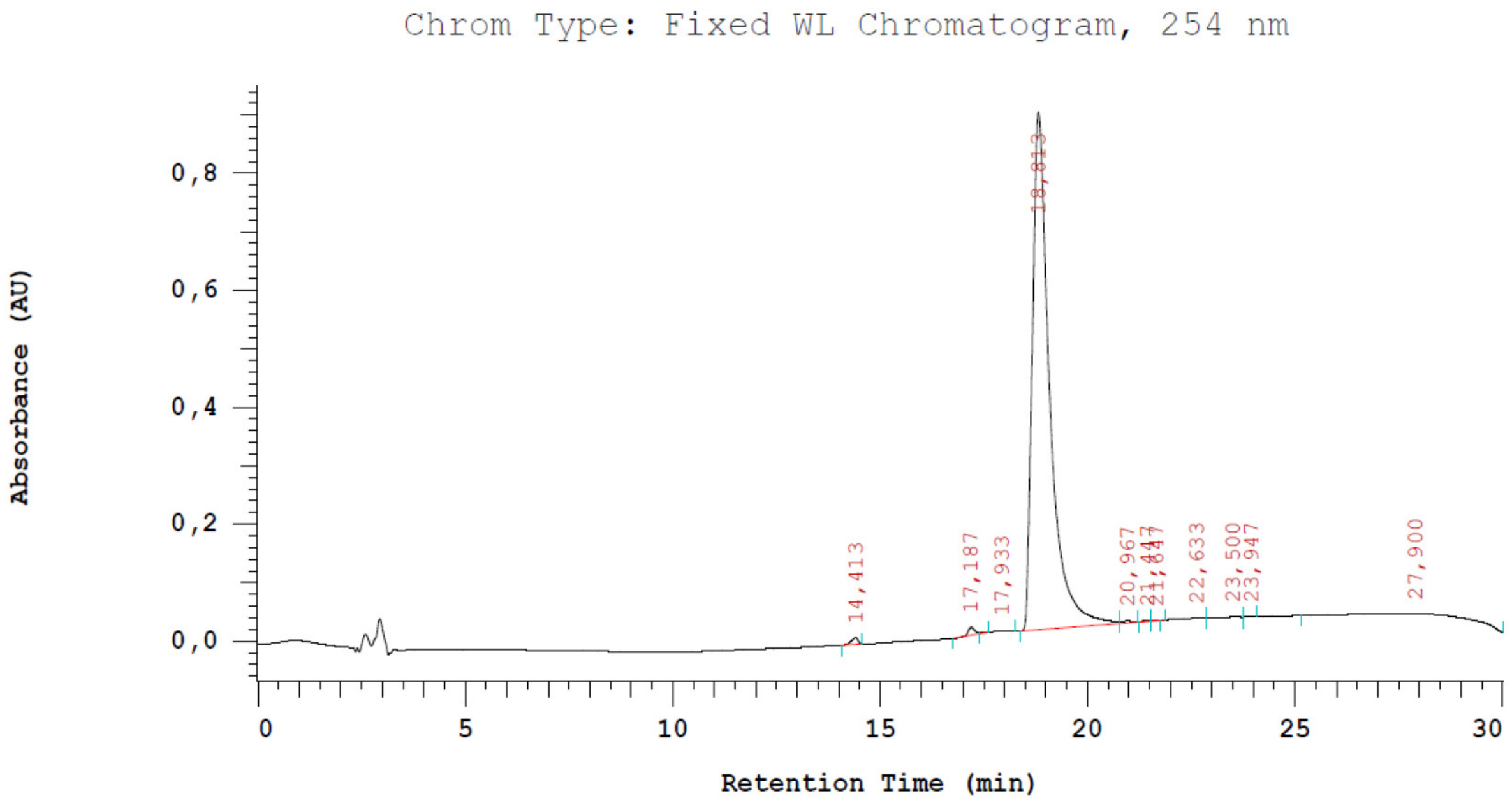

No.	RT	Area	Conc 1	BC
1	14,413	62437	0,482	MC
2	17,187	76157	0,588	MC
3	17,933	0	0,000	
4	18,813	12757788	98,498	MC
5	20,967	36706	0,283	MC
6	21,447	13478	0,104	MC
7	21,647	5721	0,044	MC
8	22,633	0	0,000	
9	23,500	0	0,000	
10	23,947	0	0,000	
11	27,900	0	0,000	
		12952287	100,000	

HPLC chromatogram of **4e**:

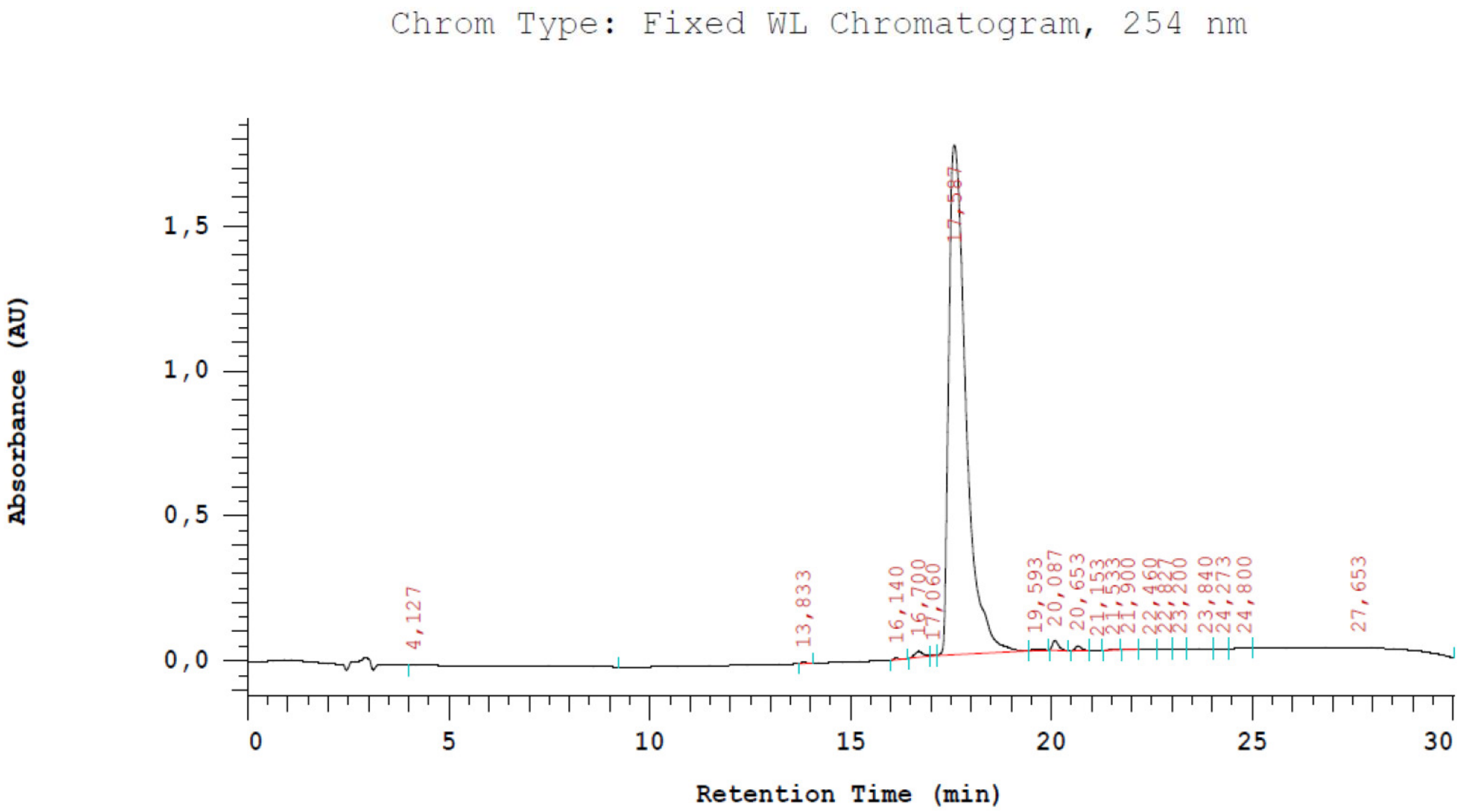

No.	RT	Area	Conc 1	BC
1	4,127	0	0,000	
2	13,833	17147	0,064	MC
3	16,140	23490	0,087	MC
4	16,700	133251	0,494	MC
5	17,060	3069	0,011	MC
6	17,587	26438059	98,015	MC
7	19,593	47856	0,177	MC
8	20,087	182153	0,675	MC
9	20,653	91317	0,339	MC
10	21,153	0	0,000	
11	21,533	20059	0,074	MC
12	21,900	17024	0,063	MC
13	22,460	0	0,000	
14	22,827	0	0,000	
15	23,200	0	0,000	
16	23,840	0	0,000	
17	24,273	0	0,000	
18	24,800	0	0,000	
19	27,653	0	0,000	
		26973425	100,000	

SUPPORTING INFORMATION

HPLC chromatogram of **4f**:

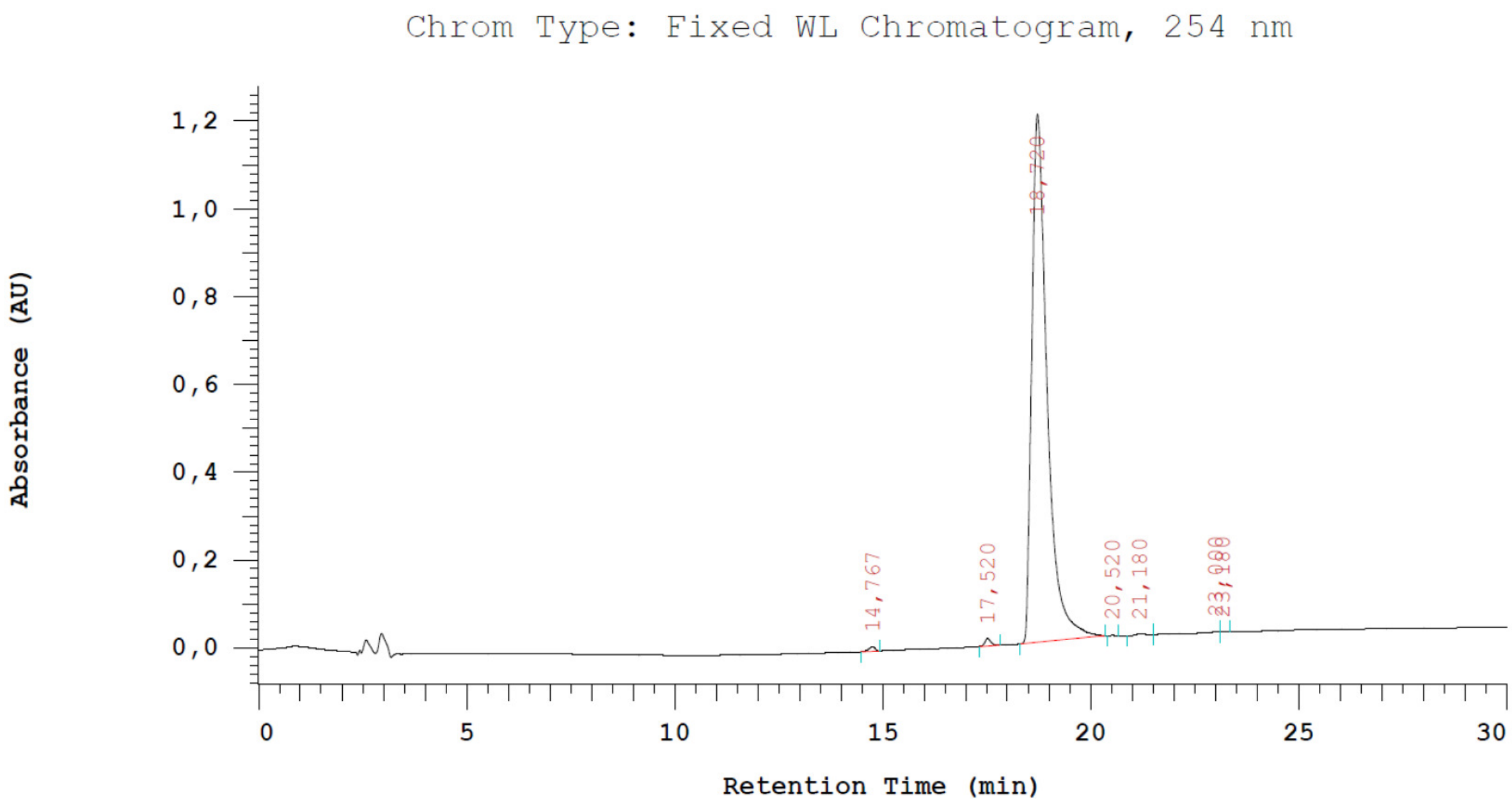

No.	RT	Area	Conc 1
1	14,767	54966	0,358
2	17,520	88105	0,574
3	18,720	15217376	99,069
4	20,520	0	0,000
5	21,180	0	0,000
6	23,000	0	0,000
7	23,180	0	0,000
		15360447	100,000

HPLC chromatogram of **5**:

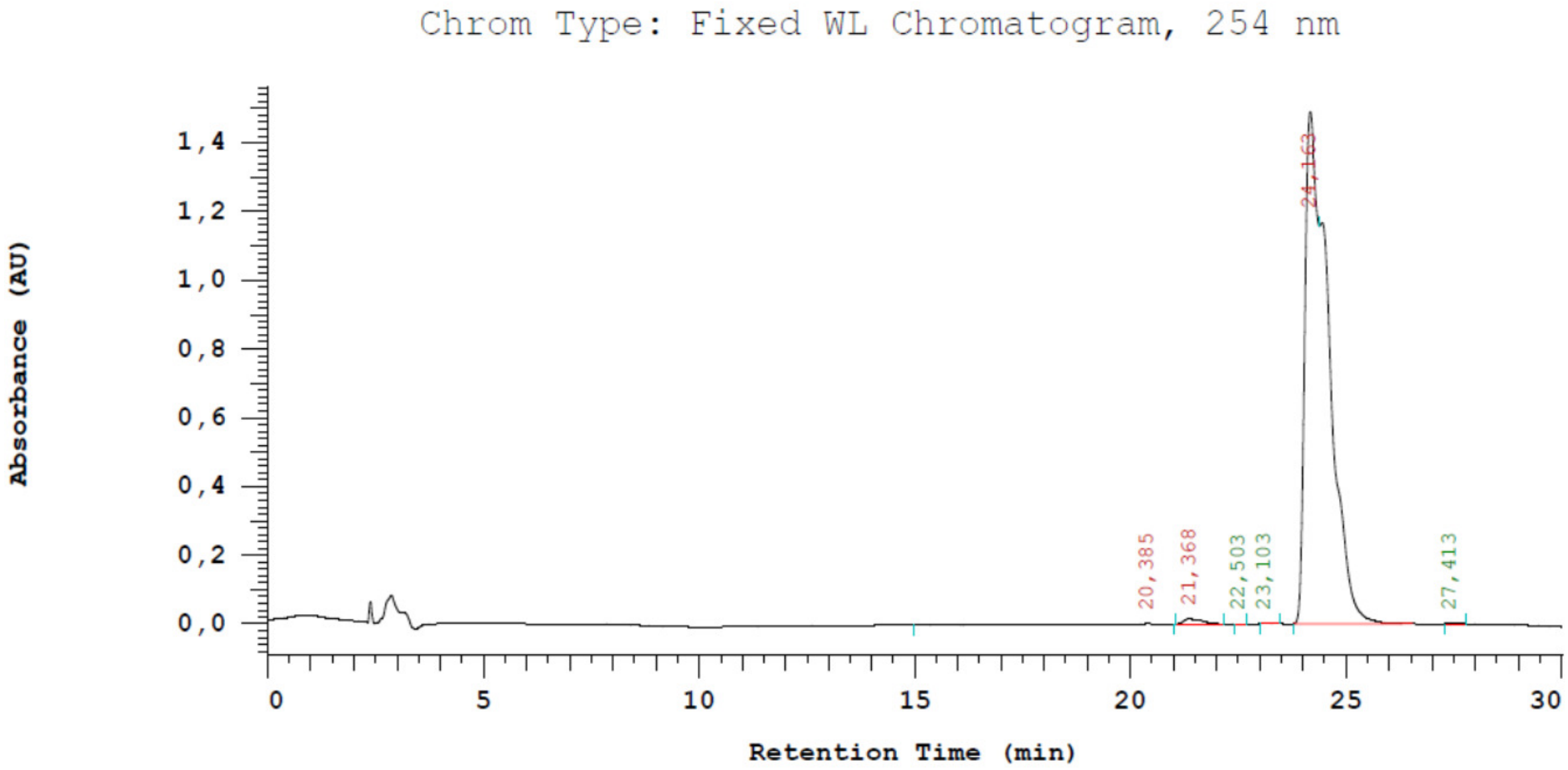

No.	Name	RT	Area	Area %
1		20,385	0	0,000
2		21,368	241608	1,591
3		22,503	2734	0,018
4		23,103	11093	0,073
5		24,163	14920503	98,280
6		27,413	5659	0,037
			15181597	100,000

Supplementary Compound Table (InChI format)

Table S6 Novel compounds with corresponding InChI codes and data from biological screening, asterisk: Minimal trophicidal concentrations after 14 days, n.d. – not determined; mean ± SD, n = 3-6

Compound No.	InChI	Biological Activity (MTC_{14d})*
**3**	InChI=1S/C23H27BrFNO2/c1-3-14-26(2)15-6-4-5-7-16-28-20-12-13-21(22(25)17-20)23(27)18-8-10-19(24)11-9-18/h3,8-13,17H,1,4-7,14-16H2,2H3	22.8 ± 8.0 µM
**4a**	InChI=1S/C23H29BrFNO2/c1-3-14-26(2)15-6-4-5-7-16-28-20-12-13-21(22(25)17-20)23(27)18-8-10-19(24)11-9-18/h8-13,17H,3-7,14-16H2,1-2H3	58.6 ± 21.4 µM
**4b**	InChI=1S/C23H25BrFNO2/c1-3-14-26(2)15-6-4-5-7-16-28-20-12-13-21(22(25)17-20)23(27)18-8-10-19(24)11-9-18/h1,8-13,17H,4-7,14-16H2,2H3	11.4 ± 4.0 µM
**4c**	InChI=1S/C24H29BrFNO2/c1-3-15-27(2)16-7-5-4-6-8-17-29-21-13-14-22(23(26)18-21)24(28)19-9-11-20(25)12-10-19/h3,9-14,18H,1,4-8,15-17H2,2H3	39.1 ± 0.0 µM
**4d**	InChI=1S/C23H27ClFNO2/c1-3-14-26(2)15-6-4-5-7-16-28-20-12-13-21(22(25)17-20)23(27)18-8-10-19(24)11-9-18/h3,8-13,17H,1,4-7,14-16H2,2H3	117 ± 68 µM
**4e**	InChI=1S/C23H28FNO2/c1-3-15-25(2)16-9-4-5-10-17-27-20-13-14-21(22(24)18-20)23(26)19-11-7-6-8-12-19/h3,6-8,11-14,18H,1,4-5,9-10,15-17H2,2H3	≥ 156 µM
**4f**	InChI=1S/C23H25ClFNO2/c1-3-14-26(2)15-6-4-5-7-16-28-20-12-13-21(22(25)17-20)23(27)18-8-10-19(24)11-9-18/h1,8-13,17H,4-7,14-16H2,2H3	5.70 ± 1.99 µM
**5**	InChI=1S/C22H24BrFO3/c1-2-13-26-14-5-3-4-6-15-27-19-11-12-20(21(24)16-19)22(25)17-7-9-18(23)10-8-17/h2,7-12,16H,1,3-6,13-15H2	≥ 156 µM
**6a**	InChI=1S/C14H10BrFO2/c1-18-11-6-7-12(13(16)8-11)14(17)9-2-4-10(15)5-3-9/h2-8H,1H3	n.d.
**6b**	InChI=1S/C14H10ClFO2/c1-18-11-6-7-12(13(16)8-11)14(17)9-2-4-10(15)5-3-9/h2-8H,1H3	n.d.
**6c**	InChI=1S/C14H11FO2/c1-17-11-7-8-12(13(15)9-11)14(16)10-5-3-2-4-6-10/h2-9H,1H3	n.d.
**7a**	InChI=1S/C13H8BrFO2/c14-9-3-1-8(2-4-9)13(17)11-6-5-10(16)7-12(11)15/h1-7,16H	n.d.
**7b**	InChI=1S/C13H8ClFO2/c14-9-3-1-8(2-4-9)13(17)11-6-5-10(16)7-12(11)15/h1-7,16H	n.d.
**7c**	InChI=1S/C13H9FO2/c14-12-8-10(15)6-7-11(12)13(16)9-4-2-1-3-5-9/h1-8,15H	n.d.
**8a**	InChI=1S/C19H19Br2FO2/c20-11-3-1-2-4-12-24-16-9-10-17(18(22)13-16)19(23)14-5-7-15(21)8-6-14/h5-10,13H,1-4,11-12H2	n.d.
**8b**	InChI=1S/C19H19BrClFO2/c20-11-3-1-2-4-12-24-16-9-10-17(18(22)13-16)19(23)14-5-7-15(21)8-6-14/h5-10,13H,1-4,11-12H2	n.d.
**8c**	InChI=1S/C19H20BrFO2/c20-12-6-1-2-7-13-23-16-10-11-17(18(21)14-16)19(22)15-8-4-3-5-9-15/h3-5,8-11,14H,1-2,6-7,12-13H2	n.d.
**8d**	InChI=1S/C20H21Br2FO2/c21-12-4-2-1-3-5-13-25-17-10-11-18(19(23)14-17)20(24)15-6-8-16(22)9-7-15/h6-11,14H,1-5,12-13H2	n.d.
**9**	InChI=1S/C9H17BrO/c1-2-8-11-9-6-4-3-5-7-10/h2H,1,3-9H2	n.d.

References

[1] Thoma, R.; Schulz-Gasch, T.; D'Arcy, B.; Benz, J.; Aebi, J.; Dehmlow, H.; Hennig, M.; Stihle, M.; Ruf, A. Insight into steroid scaffold formation from the structure of human oxidosqualene cyclase. *Nature* **2004**, *432*, 118–122. DOI: 10.1038/nature02993.

[2] Sievers, F.; Wilm, A.; Dineen, D.; Gibson, T. J.; Karplus, K.; Li, W.; Lopez, R.; McWilliam, H.; Remmert, M.; Söding, J.*; et al.* Fast, scalable generation of high‐quality protein multiple sequence alignments using Clustal Omega. *Molecular Systems Biology* **2011**, *7*, MSB201175. DOI: 10.1038/msb.2011.75.

[3] Goujon, M.; McWilliam, H.; Li, W.; Valentin, F.; Squizzato, S.; Paern, J.; Lopez, R. A new bioinformatics analysis tools framework at EMBL–EBI. *Nucleic Acids Res* **2010**, *38*, W695-W699. DOI: 10.1093/nar/gkq313.
